# Coligand role in the NHC nickel catalyzed C–F bond activation: investigations on the insertion of bis(NHC) nickel into the C–F bond of hexafluorobenzene†

**DOI:** 10.1039/d0sc04237d

**Published:** 2020-10-06

**Authors:** Maximilian W. Kuntze-Fechner, Hendrik Verplancke, Lukas Tendera, Martin Diefenbach, Ivo Krummenacher, Holger Braunschweig, Todd B. Marder, Max C. Holthausen, Udo Radius

**Affiliations:** Institute for Inorganic Chemistry, Julius-Maximilians-Universität Würzburg Am Hubland 97074 Würzburg Germany u.radius@uni-wuerzburg.de; Institute for Inorganic and Analytical Chemistry, Goethe-Universität Frankfurt Max-von-Laue-Strasse 7 60438 Frankfurt Germany max.holthausen@chemie.uni-frankfurt.de; Institute for Sustainable Chemistry & Catalysis with Boron, Julius-Maximilians-Universität Würzburg Am Hubland 97074 Würzburg Germany

## Abstract

The reaction of [Ni(Mes_2_Im)_2_] (**1**) (Mes_2_Im = 1,3-dimesityl-imidazolin-2-ylidene) with polyfluorinated arenes as well as mechanistic investigations concerning the insertion of **1** and [Ni(^i^Pr_2_Im)_2_] (**1ipr**) (^i^Pr_2_Im = 1,3-diisopropyl-imidazolin-2-ylidene) into the C–F bond of C_6_F_6_ is reported. The reaction of **1** with different fluoroaromatics leads to formation of the nickel fluoroaryl fluoride complexes *trans*-[Ni(Mes_2_Im)_2_(F)(Ar^F^)] (Ar^F^ = 4-CF_3_-C_6_F_4_**2**, C_6_F_5_**3**, 2,3,5,6-C_6_F_4_N **4**, 2,3,5,6-C_6_F_4_H **5**, 2,3,5-C_6_F_3_H_2_**6**, 3,5-C_6_F_2_H_3_**7**) in fair to good yields with the exception of the formation of the pentafluorophenyl complex **3** (less than 20%). Radical species and other diamagnetic side products were detected for the reaction of **1** with C_6_F_6_, in line with a radical pathway for the C–F bond activation step using **1**. The difluoride complex *trans*-[Ni(Mes_2_Im)_2_(F)_2_] (**9**), the bis(aryl) complex *trans*-[Ni(Mes_2_Im)_2_(C_6_F_5_)_2_] (**15**), the structurally characterized nickel(i) complex *trans*-[Ni^I^(Mes_2_Im)_2_(C_6_F_5_)] (**11**) and the metal radical *trans*-[Ni^I^(Mes_2_Im)_2_(F)] (**12**) were identified. Complex **11**, and related [Ni^I^(Mes_2_Im)_2_(2,3,5,6-C_6_F_4_H)] (**13**) and [Ni^I^(Mes_2_Im)_2_(2,3,5-C_6_F_3_H_2_)] (**14**), were synthesized independently by reaction of *trans*-[Ni(Mes_2_Im)_2_(F)(Ar^F^)] with PhSiH_3_. Simple electron transfer from **1** to C_6_F_6_ was excluded, as the redox potentials of the reaction partners do not match and [Ni(Mes_2_Im)_2_]^+^, which was prepared independently, was not detected. DFT calculations were performed on the insertion of [Ni(^i^Pr_2_Im)_2_] (**1ipr**) and [Ni(Mes_2_Im)_2_] (**1**) into the C–F bond of C_6_F_6_. For **1ipr**, concerted and NHC-assisted pathways were identified as having the lowest kinetic barriers, whereas for **1**, a radical mechanism with fluoride abstraction and an NHC-assisted pathway are both associated with almost the same kinetic barrier.

## Abstract 2

A combined experimental and theoretical study on the mechanism of the C–F bond activation of C_6_F_6_ with [Ni(NHC)_2_] is provided.

## Graphical abstract

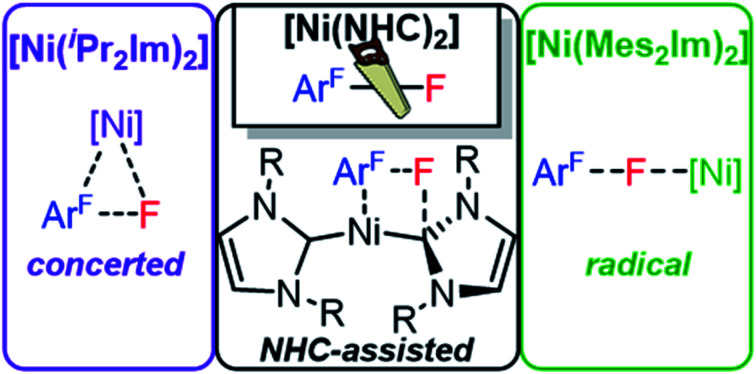

## Introduction

**Scheme 1:**
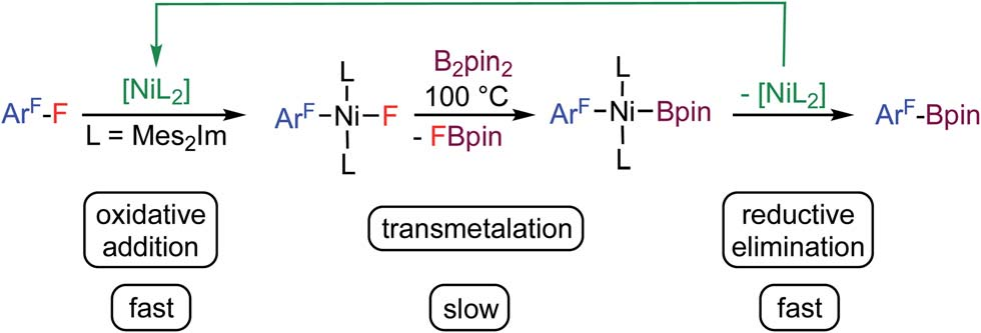
Thermal borylation of fluoroarenes with B_2_pin_2_ mediated by [Ni(Mes_2_Im)_2_] *via* the oxidative addition product *trans*-[Ni(Mes_2_Im)_2_(F)(Ar^F^)] as the resting state of the catalysis.

Fluorinated organic compounds have exceptional properties that are being exploited in many applications including materials, pharmaceuticals and agrochemicals. The development of methods to introduce fluorinated aromatic building blocks selectively into organic molecules is thus of fundamental interest in many areas of chemical research.^1^ One strategy for such transformations is the selective activation and subsequent functionalization of C–F bonds of readily available fluoroorganic compounds such as fluoroaromatics. The challenge here is the selective cleavage of very stable C–F bonds.^2^ We have recently established a protocol for the transformation of commercially available fluoroaromatics *via* a selective C–F defluoroborylation process to obtain polyfluorinated arylboronic esters,^3^ which may be further used in late stage functionalization, for example in Suzuki–Miyaura cross-coupling reactions.^4^ Defluoroborylation of polyfluoroaromatics can be achieved by a thermal [Ni(Mes_2_Im)_2_]-catalyzed (Mes_2_Im = 1,3-dimesityl-imidazolin-2-ylidene) transformation of polyfluoroarenes into fluoroaryl boronic acid pinacol esters *via* C–F bond activation and transmetalation with bis(pinacolato)diboron (B_2_pin_2_) as the boron source (see Scheme 1).^3*a*^ Various arenes with different degrees of fluorination were converted into their corresponding boronate esters in this way. One particularly interesting finding of our study was that activation of the C–F bond by the nickel(0) complex is fast at ambient temperature. This step yields the oxidative addition product *trans*-[Ni(Mes_2_Im)_2_(F)(Ar^F^)] (Ar^F^ = fluoroaryl), which represents the resting state in the catalytic cycle. The subsequent defluoroborylation step with B_2_pin_2_ is the rate determining step and requires elevated temperatures. A boryl complex *trans*-[Ni(Mes_2_Im)_2_(Bpin)(Ar^F^)], a likely intermediate, was never observed and stoichiometric reactions of *trans*-[Ni(Mes_2_Im)_2_(F)(Ar^F^)] with B_2_pin_2_ led directly to the formation of Ar^F^–Bpin. This finding implied that reductive elimination is very fast and that [Ni(Mes_2_Im)_*n*_(Bpin)(Ar^F^)], once formed, will eliminate Ar^F^–Bpin immediately (Scheme 1).^5*a*^

As an alternative to the thermally-induced C–F bond activation and subsequent borylation of fluoroarenes, we have recently developed a process that employs visible-light photocatalysis, which has emerged as a powerful tool in organic synthesis.^6^ Our highly selective and general photocatalytic C–F borylation protocol^3*b*^ employs a rhodium biphenyl complex^7^ as triplet sensitizer combined with the nickel catalyst [Ni(Mes_2_Im)_2_] (**1**) for the C–F bond activation step and the defluoroborylation process. This Rh/Ni tandem catalyst system operates with visible light (400 nm) and achieves the highly selective borylation of a wide range of polyfluoroarenes with B_2_pin_2_ at room temperature in excellent yields. Both procedures, the thermal and photochemical defluoroborylation, work well for partially fluorinated aromatics but fail, or afford only low yields, for perfluoroaromatics such as hexafluorobenzene or octafluorotoluene.

**Scheme 2:**
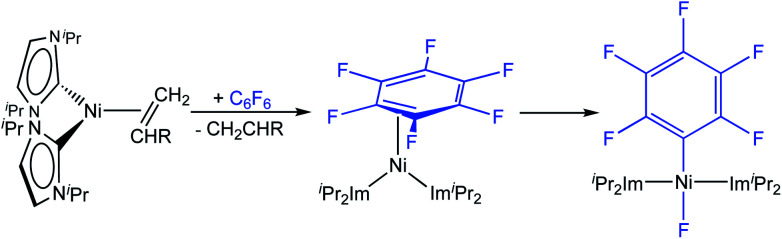
Stoichiometric C–F bond activation of C_6_F_6_ using sources of [Ni(^i^Pr_2_Im)_2_] **1iPr**.

Utilizing the dinuclear complex [Ni_2_(^i^Pr_2_Im)_4_(μ-(η^2^:η^2^)-COD)] (^i^Pr_2_Im = 1,3-diisopropyl-imidazolin-2-ylidene) or the ethylene complex [Ni(^i^Pr_2_Im)_2_(η^2^-C_2_H_4_)]^8^ as sources of [Ni(^i^Pr_2_Im)_2_] (**1iPr**), we previously found that both readily undergo C–F bond insertion with a wide variety of per- and polyfluoroaromatics on a time scale suitable for catalysis (exemplarily shown for C_6_F_6_ in Scheme 2).^4*c*,*k*,9^ Mechanistic investigations^9*a*^ of the insertion process were performed using the ethylene complex [Ni(^i^Pr_2_Im)_2_(η^2^-C_2_H_4_)] as nickel precursor. Ethylene exchange at the [Ni(^i^Pr_2_Im)_2_(η^2^-C_2_H_4_)] complex with hexafluorobenzene and octafluoronaphthalene occurs at low temperatures (−80 °C and −30 °C, respectively; Scheme 2). Subsequent insertion reactions occur at higher temperatures (0 °C and 20 °C, respectively) to form the *trans*-[Ni(^i^Pr_2_Im)_2_(F)(C_6_F_5_)] and *trans*-[Ni(^i^Pr_2_Im)_2_(F)(C_10_F_7_)] fluoroaryl fluoride complexes.^9^ We studied the C–F bond activation kinetics and, based on the decay rates of the octafluoronaphthalene complex [Ni(^i^Pr_2_Im)_2_(η^2^-C_10_F_8_)] determined by variable-temperature NMR spectroscopy, we derived an activation enthalpy of Δ*H*^‡^ = 27.7 ± 1.9 kcal mol^−1^ (Δ*S*^‡^ = 8.8 ± 6.0 cal K^−1^ mol^−1^).

We report herein on the reactivity of **1** with polyfluorinated arenes. We compare the results with those of earlier studies on C–F bond activation processes using nickel complexes with sterically less demanding NHCs, employing ^i^Pr instead of Mes substituents, *i.e.*, using [Ni(^i^Pr_2_Im)_2_] (**1ipr**) as the nickel source. We demonstrate that the complex of the small NHC ligand ^i^Pr_2_Im favors a concerted oxidative addition proceeding through an η^2^(C,C) intermediate in reactions with fluoroarenes to yield *trans*-[Ni^II^(NHC)_2_(F)(Ar^F^)] complexes, whereas the complex of the larger Mes_2_Im ligand leads to fluorine atom abstraction to yield [Ni^I^(NHC)_2_(F)] and a phenyl radical. For both mechanisms, competitive NHC-assisted pathways are found which account for the formation of diamagnetic products by a C–F bond activation step across the Ni–C_NHC_ bond. These NHC-assisted pathways play an important role for complexes of both sterically demanding and less bulky NHC ligands, and should thus be of general importance and widely applicable for the reactivity of NHC-stabilized transition metal complexes.

## Results and discussion

### C–F bond activation of fluoroaromatics

**Scheme 3:**
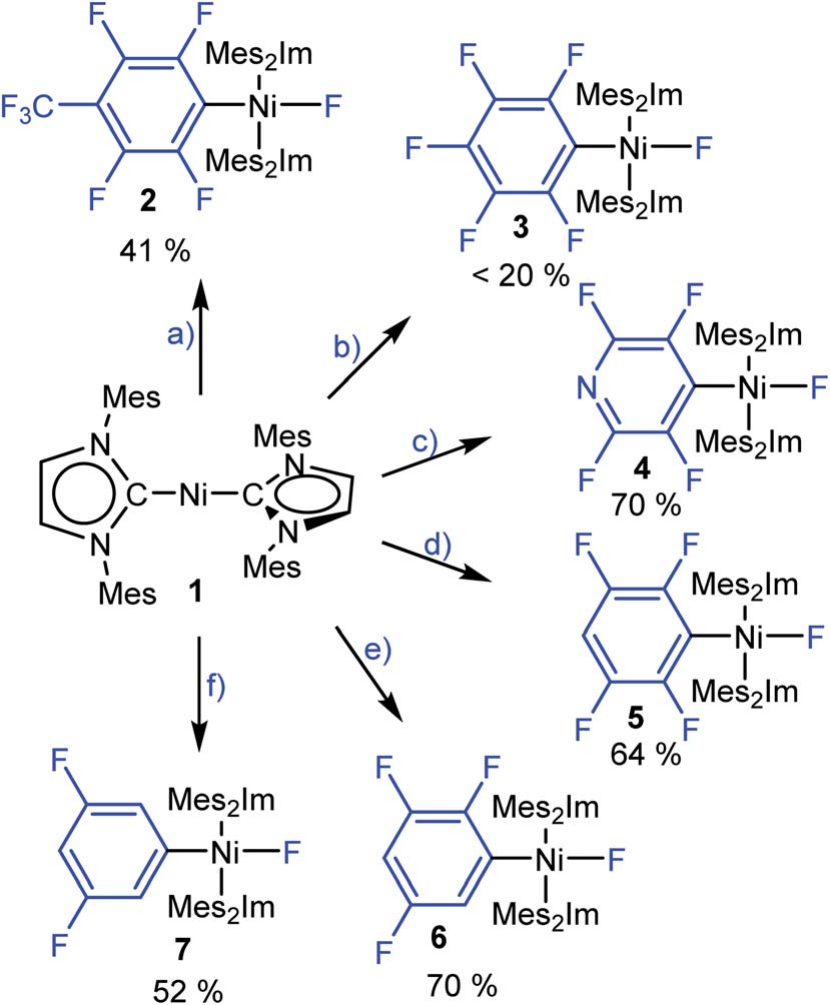
The reactions of [Ni(Mes_2_Im)_2_] (**1**) with (a) octafluorotoluene, (b) hexafluorobenzene, (c) perfluoropyridine, (d) pentafluorobenzene, (e) 1,2,3,5-tetrafluorobenzene and (f) 1,3,5-trifluorobenzene to give the complexes *trans*-[Ni(Mes_2_Im)_2_(F)(4-CF_3_-C_6_F_4_)] (**2**), *trans*-[Ni(Mes_2_Im)_2_(F)(C_6_F_5_)] (**3**), *trans*-[Ni(Mes_2_Im)_2_(F)(2,3,5,6-C_5_F_4_N)] (**4**), *trans*-[Ni(Mes_2_Im)_2_(F)(2,3,5,6-C_6_F_4_H)] (**5**), *trans*-[Ni(Mes_2_Im)_2_(F)(2,3,5-C_6_F_3_H_2_)] (**6**) and *trans*-[Ni(Mes_2_Im)_2_(F)(3,5-C_6_F_2_H_3_)] (**7**), respectively. Isolated yields are given.

To gain insight into the C–F bond activation process using [Ni(Mes_2_Im)_2_] (**1**), we first investigated stoichiometric reactions of perfluorotoluene, perfluorobenzene, perfluoropyridine and the partially fluorinated arenes pentafluorobenzene, 1,2,3,5-tetrafluorobenzene and 1,3,5-trifluorobenzene with **1** (see Scheme 3). We monitored the reactions by ^1^H and ^19^F{^1^H} NMR spectroscopy and observed a significant effect of the degree of fluorination on both reaction rate and yield. Reactions of **1** with hexafluorobenzene and octafluorotoluene proceed within seconds at room temperature, whereas the reactions with tetra- and pentafluorobenzene take minutes to complete. With 1,3,5-trifluorobenzene, full conversion of **1** takes weeks at room temperature (see ESI, Fig. S1†), but can be accelerated at 80 °C in thf to reach completion after 5 days.

These reactions can be performed in thf, toluene or hexane at room temperature and lead, in each case, to the insertion of the nickel complex into the C–F bond of the fluoroarene to form the nickel fluoroaryl fluoride complexes *trans*-[Ni(Mes_2_Im)_2_(F)(Ar^F^)] (Ar^F^ = 4-CF_3_-C_6_F_4_**2**, C_6_F_5_**3**, 2,3,5,6-C_5_F_4_N **4**, 2,3,5,6-C_6_F_4_H **5**, 2,3,5-C_6_F_3_H_2_**6**, 3,5-C_6_F_2_H_3_**7**) in fair to good isolated yields. Notably, however, the reaction with C_6_F_6_ yields less than 20% of the pentafluorophenyl complex **3**. Higher temperature, different solvents (thf, toluene, hexane) or added [NMe_4_]F does not seem to affect the yield of the insertion product **3**. Complexes **2–7** were characterized by elemental analysis, ^1^H, ^19^F{^1^H} and ^13^C{^1^H} NMR spectroscopy (see ESI†). In the ^19^F{^1^H} NMR spectra of these complexes, the resonances of the nickel-bound fluoride ligand were observed in the typical range between −361.9 and −333.1 ppm. Within the series presented (see ESI, Table S1†), the NMR shift of this resonance depends on the degree of fluorination of the fluoroaryl ligands, *i.e.*, an increase of the degree of fluorination of the aryl ligand leads to an upfield shift of the Ni–F resonance.

**Fig. 1:**
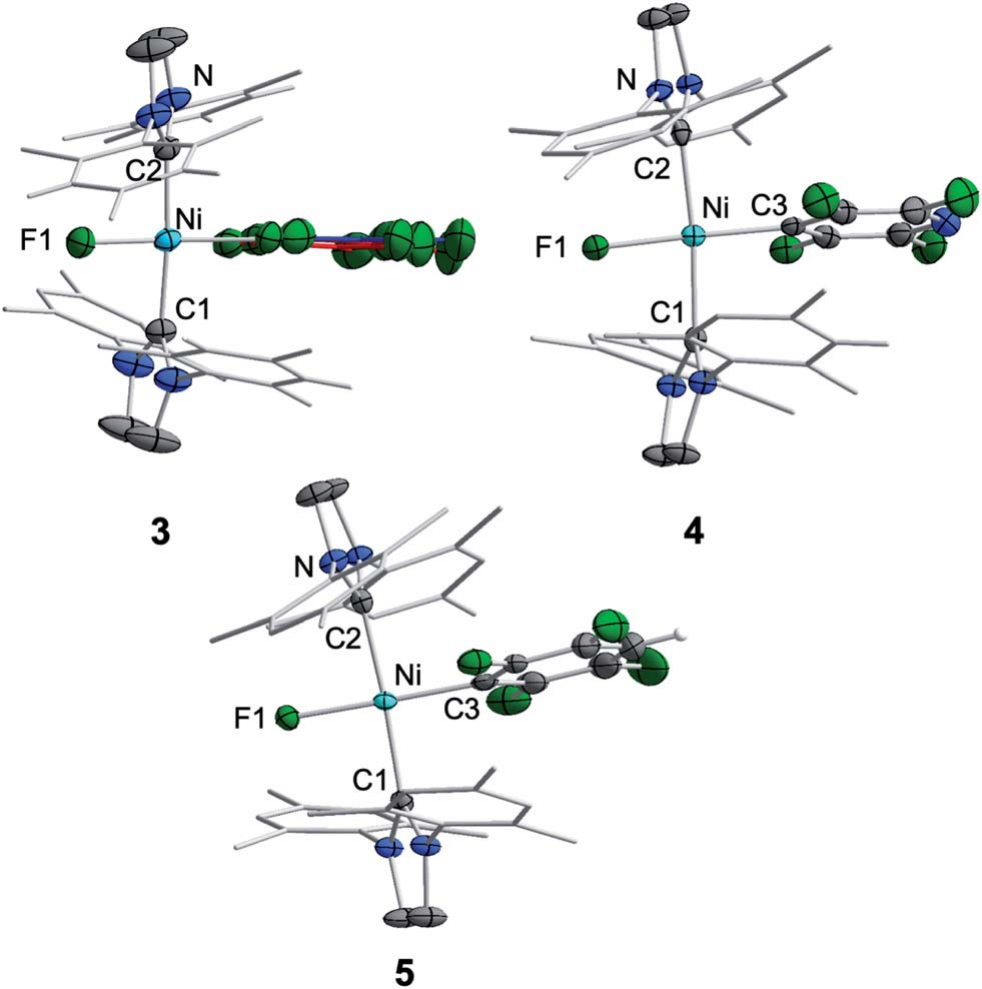
Molecular structures of *trans*-[Ni(Mes_2_Im)_2_(F)(C_6_F_5_)] (**3**) (top left), *trans*-[Ni(Mes_2_Im)_2_(F)(2,3,5,6-C_5_F_4_N)] (**4**) (top right) and *trans*-[Ni(Mes_2_Im)_2_(F)(2,3,5,6-C_6_F_4_H)] (**5**) (bottom) in the solid state (ellipsoids drawn at the 50% probability level). Hydrogens atoms, with exception of the proton at the fluoroaromatic of **5**, are omitted for clarity.

Crystals of **3**, **4**, and **5** suitable for X-ray diffraction were obtained from saturated solutions of these compounds either in pentane or hexane at −30 °C (Fig. 1, Table 1; see also ESI Fig. S34–S36 and Table S2†). The crystal structure of **6** was published previously.^3*a*^ All complexes of the type *trans*-[Ni(Mes_2_Im)_2_(F)(Ar^F^)] (Ar^F^ = C_6_F_5_**3**, 2,3,5,6-C_5_F_4_N **4**, 2,3,5,6-C_6_F_4_H **5**, 2,3,5-C_6_F_3_H_2_**6**) adopt a square planar structure with a *trans* arrangement of the NHC ligands. An increasing degree of fluorination of the fluoroaryl ligand leads to a slight shortening of the Ni–F bond lengths (Ni–F: **6**: 1.874(2) Å, **5**: 1.856(2) Å, **4**: 1.859(2) Å, **3**: 1.844(2) Å), while the distances of the nickel center to the fluoroaryl ligand become gradually longer (Ni–C3: **6**: 1.854(5) Å, **5**: 1.896(3) Å, **4**: 1.883(3) Å, **3**: 1.944(5) Å). We assume that both the upfield shift of the Ni–F ^19^F NMR resonance and the shortening of the Ni–F bond lengths with increasing degree of aryl fluorination are indications of stronger Ni–F bonding.

**Table 1:** Crystallographic data for compounds **1**, **3**, **4**, **5**, **6**, **8**, **9**, **11**, **13**, **14**, [Ni^I^(6-Mes)_2_][Br]^11*a*^ and [Ni^I^(P^i^Pr_3_)_2_(C_6_F_5_)]^22^

	*d* Ni–C1/C2	*d* Ni–C3_(ArF)_	*d* Ni–F	∠C1–Ni–C2	∠NHC(C1) : NHC(C2)
[Ni(Mes_2_Im)_2_] **1**	1.827(6)	—	—	176.4	53.0
	1.830(6)				
**3**	1.923(3)	C3: 1.882(7)	1.844(2)	175.6(1)	37.07(2)
	1.922(3)	C3′: 1.944(5)			
**4**	1.923(3)	1.883(3)	1.859(2)	174.3(1)	36.01(2)
	1.920(3)				
**5**	1.921(2)	1.896(3)	1.856(2)	176.4(1)	33.81(1)
	1.924(2)				
**6**	1.912(3)	1.854(5)	1.874(2)	176.7(1)	31.65(2)
	1.912(3)				
**8**	1.894(3)	—	—	174.5(1)	57.99(1)
	1.894(3)				
**9**	1.903(3)	—	F1: 1.845(2)	178.5(1)	53.34(1)
	1.902(3)		F2: 1.823(2)		
**11**	1.923(2)	1.984(3)	—	159.8(8)	82.37(1)
	1.923(2)				
**13**	1.930(2)	1.987(3)	—	157.3(8)	82.11(1)
	1.930(2)				
**14**	1.918(1)	C3: 1.869(1)	—	159.5(5)	82.46(8)
	1.917(1)	C3′: 2.046(1)			
[Ni^I^(6-Mes)_2_][Br]	1.939(3)	—	—	179.3(1)	57.99(1)
	1.941(3)				
[Ni^I^(P^i^Pr_3_)_2_(C_6_F_5_)]	P1: 2.243(5)	1.973(2)	—	P1–Ni–P2	—
	P2: 2.233(5)			145.2(2)	

As the low yield of *trans*-[Ni(Mes_2_Im)_2_(F)(C_6_F_5_)] (**3**) is in sharp contrast with the results we obtained previously for the reaction of [Ni_2_(^i^Pr_2_Im)_4_(μ-(η^2^:η^2^)-COD)] or [Ni(^i^Pr_2_Im)_2_(η^2^-C_2_H_4_)] with C_6_F_6_,^9^ we decided to take a closer look at the corresponding reaction using [Ni(Mes_2_Im)_2_] (**1**). Performing the stoichiometric reaction of **1** with C_6_F_6_ in an NMR tube in C_6_D_6_ led to an immediate color change from dark-violet, the color of concentrated complex **1**, to orange after addition of C_6_F_6_ at room temperature. A quantitative conversion of **1** was achieved after 5 min as monitored by ^1^H NMR spectroscopy (see ESI, Fig. S2†). However, the spectroscopic yield determined by ^19^F{^1^H} NMR spectroscopy after 5 min at room temperature, *vs.* a Ph–F containing capillary as internal standard, revealed the formation of **3** in approximately 17% yield and, in addition, the formation of small amounts of fluoride-containing side products (see ESI, Fig. S3†). Even after 72 h at room temperature, no increase in the spectroscopic yield of **3** was observed. In further control experiments, neither the use of an excess of **1** (2.85 equiv.) nor C_6_F_6_ (2.5 equiv.) increased the yield of **3** substantially. These experiments demonstrate that the low isolated yield of **3** is not a problem of the isolation process for this complex, but rather an intrinsic problem associated with its formation and the C–F bond activation step. Low temperature NMR experiments (−50 °C to +20 °C) revealed that a nickel fluoride resonance at −358 ppm appeared for this reaction in the ^19^F{^1^H} NMR spectrum already at −50 °C (see ESI, Fig. S4†), but also that, at these temperatures, all resonances are significantly broadened in the ^1^H NMR spectrum of the reaction mixture (see ESI, Fig. S5†). Although we previously observed some line broadening for the *N*-alkyl groups of the related complex *trans*-[Ni(^i^Pr_2_Im)_2_(F)(C_6_F_5_)],^9*a*^ which arose due to hindered rotation of the NHC ligand about the Ni–C axis, all resonances observed for the reaction of **1** with C_6_F_6_ are involved in the broadening. This led to the assumption that radical species are involved in the process. Subsequent EPR experiments were performed at −203 °C for the reaction of **1** with C_6_F_6_ which confirmed the presence of metal-centered radicals in the mixture.

**Fig. 2:**
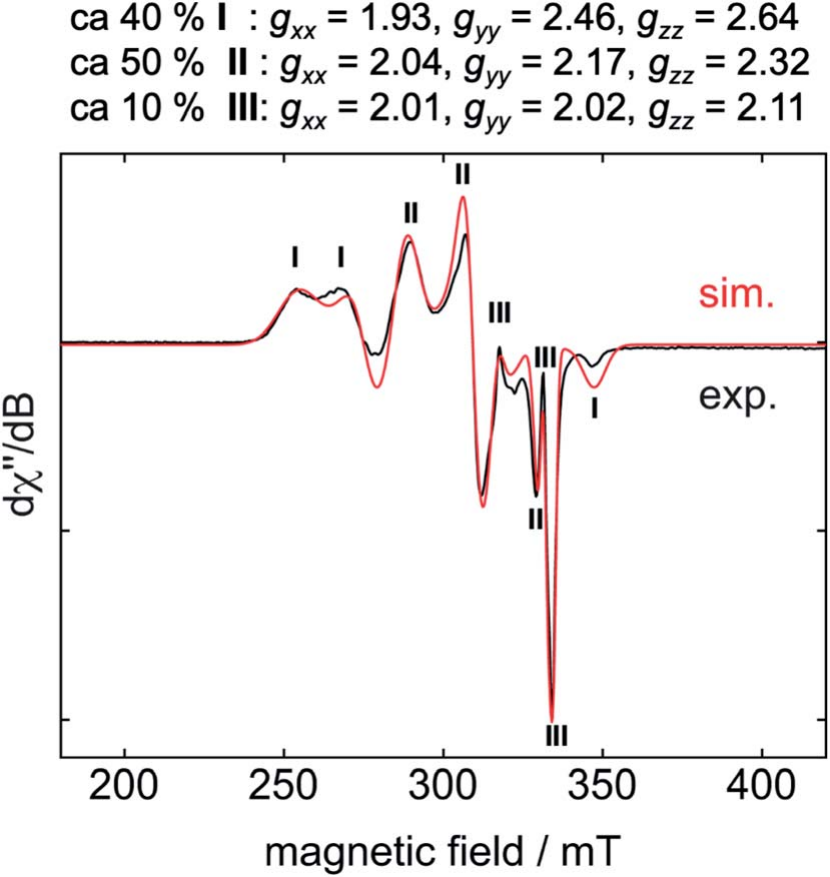
EPR spectrum (−203 °C) of the reaction mixture of **1** with C_6_F_6_ after 5 s at −78 °C in thf.

For EPR spectroscopic investigations, **1** and C_6_F_6_ were combined in an EPR tube with thf at −78 °C and the sample was frozen immediately in liquid nitrogen. The EPR tube containing the frozen reaction mixture was transferred to the cooled EPR cavity at −203 °C and a spectrum was recorded.^10^ The resulting EPR spectrum displays a superposition of resonances of three different products, of which **I** and **II** represent the two dominant species (Fig. 2, **I**: 40%, **II**: 50%, **III**: 10%).

Cyclic voltammetry results exclude a simple electron transfer from **1** to C_6_F_6_ as the origin of radical generation in the reaction mixture (see ESI, Fig. S6†), as **1** shows a reversible oxidation/reduction associated with a redox potential of −2.03 V for the redox-couple Ni^0^/Ni^I^, and an irreversible oxidation at 0.14 V for the redox-couple Ni^I^/Ni^II^. Although the reduction of C_6_F_6_ at −2.87 V is irreversible, we exclude simple one electron transfer because of the large separation of 0.84 V.

**Scheme 4:**
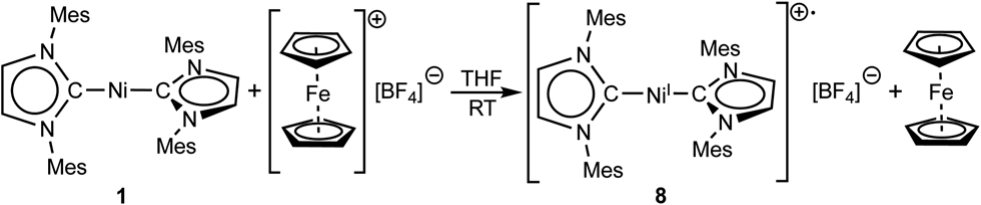
Synthesis of [Ni(Mes_2_Im)_2_][BF_4_] (**8**).

**Fig. 3:**
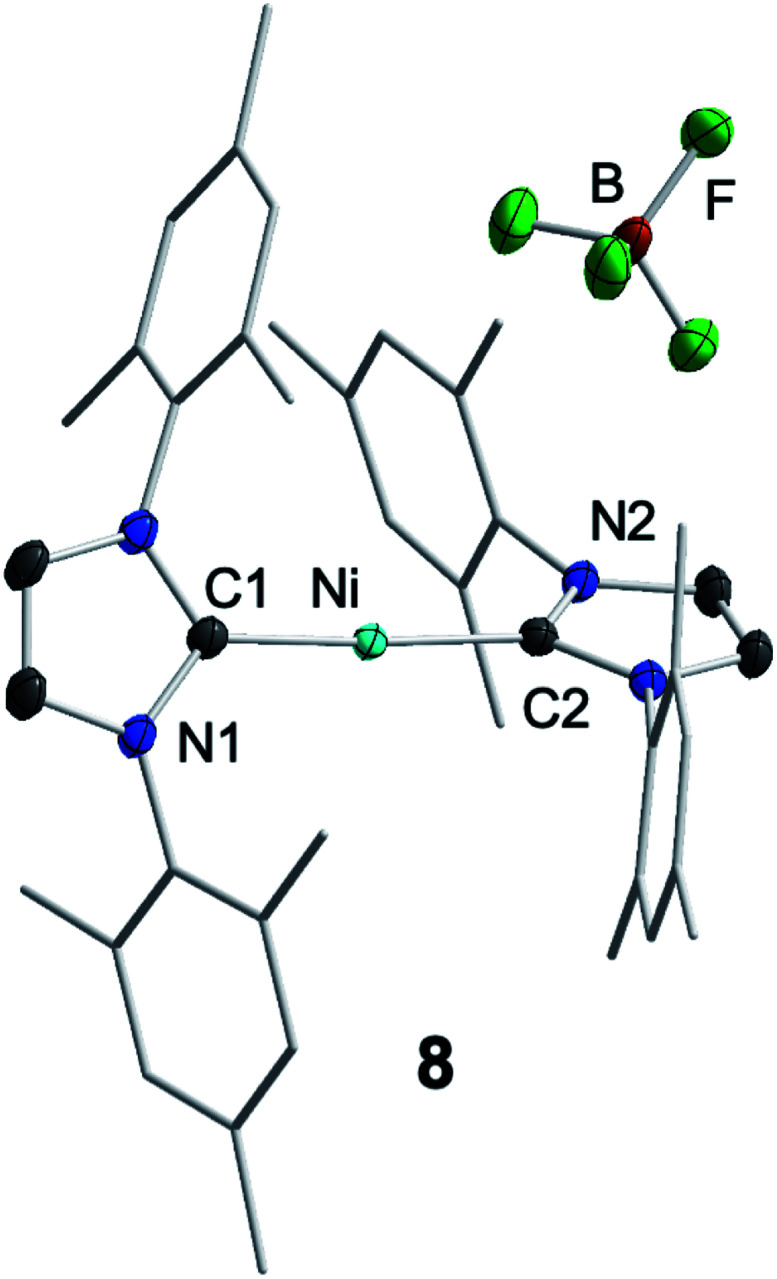
Molecular structure of [Ni(Mes_2_Im)_2_][BF_4_] (**8**) in the solid state (ellipsoids drawn at the 50% probability level). Hydrogens atoms are omitted for clarity.

For further scrutiny, complex **1** was oxidized by adding ferrocenium tetrafluoroborate in thf at room temperature to a suspension of **1** in thf. A few min after addition of the ferrocenium salt the metal-centered radical [Ni(Mes_2_Im)_2_][BF_4_] (**8**) precipitated as an off-white solid (83% isolated yield, Scheme 4), which is only sparingly soluble in common organic solvents. The Ni^I^ complex **8** was characterized by ^11^B{^1^H} and ^19^F{^1^H} NMR spectroscopy in acetonitrile (decomposition occurs after some time) and IR spectroscopy, elemental analysis and high-resolution mass spectroscopy. The ^11^B{^1^H} and ^19^F{^1^H} NMR spectra revealed an intact counter anion [BF_4_]^−^ (see ESI, Fig. S64†). Crystals of **8** suitable for X-ray diffraction (Fig. 3, Table 1; see also ESI, Table S2 and Fig. S37†) were obtained by slow evaporation of a saturated solution of **8** in a 1 : 1 toluene/ethanol mixture under an argon atmosphere at room temperature. The X-ray crystal structure reveals a nearly linear alignment of the NHC ligands with slightly elongated Ni–C distances compared to those of the starting material **1**.

**Fig. 4:**
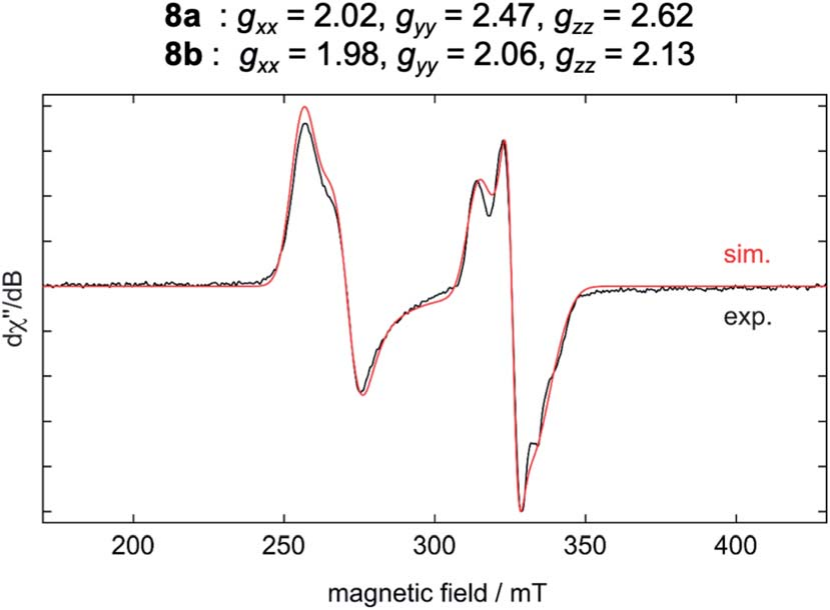
EPR spectrum of **8** in the solid state at −203 °C with NBu_4_Br.

The results of the EPR spectroscopic investigations^10^ performed on solid-state samples of [Ni(Mes_2_Im)_2_][BF_4_] (**8**) are shown in Fig. 4. The general insolubility of **8** precluded determination of its magnetic moment by the Evans method. The spectrum reveals two sets of signals, *i.e.*, **8a** (*g*_*xx*_ = 2.02, *g*_*yy*_ = 2.47, *g*_*zz*_ = 2.62; 70%) and **8b** (*g*_*xx*_ = 1.98, *g*_*yy*_ = 2.06, *g*_*zz*_ = 2.13; 30%), both in line with nickel-centered radicals. Note that in previous studies of two other homoleptic two-coordinate cationic d^9^-nickel(i) complexes, [Ni(6-Mes)_2_][Br] (6-Mes = 1,3-bis(2,4,6-trimethylphenyl)-3,4,5,6-tetrahydropyrimidin-2-ylidene) and [Ni(P^*t*^Bu_3_)_2_][Al(OC(CF_3_)_3_)_4_], no EPR signals were observed.^11^ To obtain further insight, we performed density functional theory (DFT) calculations on the molecular geometries, electronic structures and EPR parameters (*g* tensors) of a variety of potential candidates for **8** (PBE0-D/pcSseg-2, see the ESI for details†).^12^

**Fig. 5:**
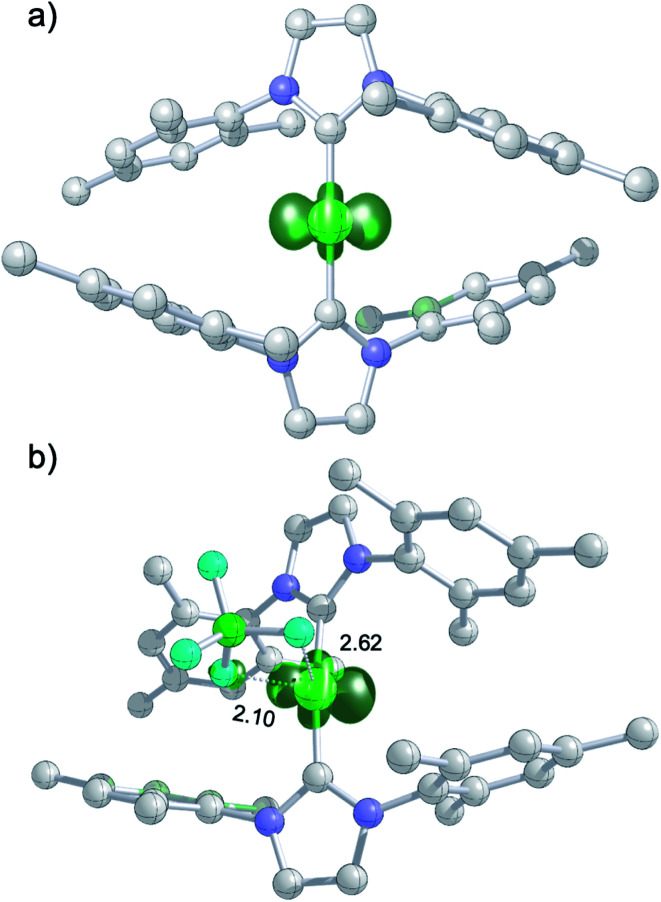
(a) Spin density plot for [Ni(Mes_2_Im)_2_]^+^; (b) molecular structure of **8DFT1** showing Ni–F^BF_4_−^ contacts (isovalue ± 0.0075 a_0_^−3^; lengths of Ni–F contacts in Å; hydrogen atoms not shown).

The DFT-optimized, *D*_2_-symmetric geometry of the [Ni(Mes_2_Im)_2_]^+^ radical cation of **8** agrees very well with the X-ray structure (Fig. 5; *e.g.*, *d*_Ni–C_ = 1.89 Å, exp: 1.894(3) Å). In the ^2^A electronic ground state, the spin density is localized at the metal center, with the unpaired electron residing in an s/d_*z*^2^_-type orbital (Fig. 5a).

The calculated *g* values for the radical cation [Ni(Mes_2_Im)_2_]^+^ (*g*_*xx*_ = 2.01, *g*_*yy*_ = 2.65, *g*_*zz*_ = 2.98), computed under gas-phase conditions, strongly differ from the experimental data with a maximum deviation of 0.36 (**8a**) and 0.85 (**8b**; see Table 2 and ESI, Table S3†). However, computations in the presence of the counter ion result in further structural motifs with impact on the computed *g* tensors (see ESI, Fig. S7†). A Ni–F contact with the counter ion in **8DFT1** (Table 2, entry 4, Fig. 5b) results in *g* tensor components closely corresponding to those of **8a** (maximum deviation: 0.03), while no species matching the EPR parameters of **8b** were identified in our computational exploration. However, none of the EPR signatures detected for the electrochemically-formed complex **8** appeared during the reaction of **1** with C_6_F_6_ (Fig. 2) and, in light of our CV results, it is unlikely that the [Ni(Mes_2_Im)_2_]^+^ cation is involved here.

**Table 2:** Experimental and DFT calculated *g* tensors for species **8**

Compound		*g* tensor components		
		*g*_*xx*_	*g*_*yy*_	*g*_*zz*_
**8a**	Exp. (solid state)	2.02	2.47	2.62
**8b**	Exp. (solid state)	1.98	2.06	2.13
[Ni(Mes_2_Im)_2_]^+^(gas phase)	DFT	2.01	2.65	2.98
**8DFT1** a	DFT	2.03	2.50	2.59

a DFT-optimized structure with Ni–F^BF_4_^−^^ contacts.

**Fig. 6:**
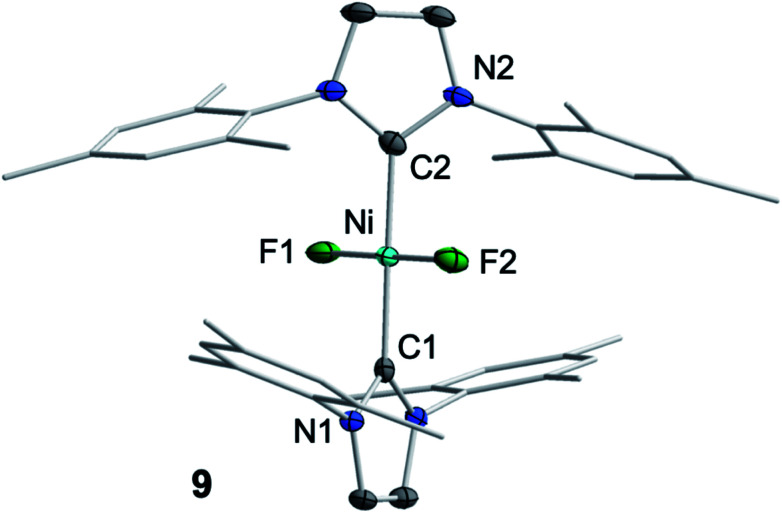
Molecular structure of *trans*-[Ni(Mes_2_Im)_2_(F)_2_] (**9**) in the solid state (ellipsoids drawn at the 50% probability level). Hydrogens atoms are omitted for clarity.

We then focused on identifying the byproducts of the reaction of **1** with C_6_F_6_. Stoichiometric reaction of **1** with C_6_F_6_ in thf overnight at room temperature led to a very small amount of a dark-green precipitate which was removed by filtration. After removal of all volatiles from the filtrate, the residue was washed with a large amount of hexane to extract the C–F bond activation product. The yellow residue, which remained after washing, was identified as the difluoride complex *trans*-[Ni(Mes_2_Im)_2_(F)_2_] (**9**) by elemental analysis, X-ray diffraction and ^1^H, ^19^F{^1^H} and ^13^C{^1^H} NMR spectroscopy (see ESI†). Most significantly, the fluoride resonance, detected as a singlet at −560 ppm in the ^19^F{^1^H} NMR spectrum, is shifted *ca.* 200 ppm to higher field compared to those of the mono-fluoride complexes **2–7** (−333 ppm to −362 ppm, *vide supra*). A similar high-field shifted fluoride resonance was also observed for the phosphine-stabilized platinum complex [Pt(P^i^Pr_3_)_2_(F)_2_] (−455.9 ppm) compared to [Pt(PPh_3_)_2_(F)(C_6_H_5_)] (−107.6 ppm).^13^ Crystals of **9** suitable for X-ray diffraction (Fig. 6, Table 1; see also ESI, Table S2 and Fig. S38†) were obtained after storing a saturated solution of the complex at room temperature in C_6_D_6_. Crystallographic analysis revealed a square planar coordination environment about the Ni^II^ center with a *trans*-arrangement of NHC and fluoride ligands.

**Scheme 5:**
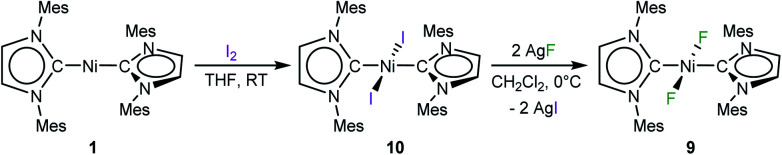
Synthesis of [Ni(Mes_2_Im)_2_(I)_2_] (**10**) and [Ni(Mes_2_Im)_2_(F)_2_] (**9**).

An independent sample of complex **9** was synthesized in 38% yield by fluorination of [Ni(Mes_2_Im)_2_(I)_2_] (**10**) using an excess (2.5 equiv.) of silver(i) fluoride in CH_2_Cl_2_ at 0 °C (Scheme 5). Complex **10** was synthesized by reaction of **1** with I_2_, isolated in 80% yield and characterized by elemental analysis, and ^1^H and ^13^C{^1^H} NMR spectroscopy (see ESI†). Interestingly, the resonance of the carbene carbon atoms is almost unaffected by substitution of the fluoride by the more electropositive iodide ligand, and was detected at 176.5 ppm (*cf.* [Ni(Mes_2_Im)_2_(F)_2_] (**9**): 174.6 ppm).

Thus, [Ni(Mes_2_Im)_2_(F)_2_] (**9**) was clearly identified as one of the side products of the reaction of **1** with C_6_F_6_. This complex is formed in low yield (17%) but in an amount similar to that of the insertion product *trans*-[Ni(Mes_2_Im)_2_(F)(C_6_F_5_)] (**3**). The amounts of complexes **9** and **3** total *ca.* 40% when the reaction of **1** with C_6_F_6_ is performed at room temperature, and thus the majority of the products formed in this reaction is still unaccounted for.

**Fig. 7:**
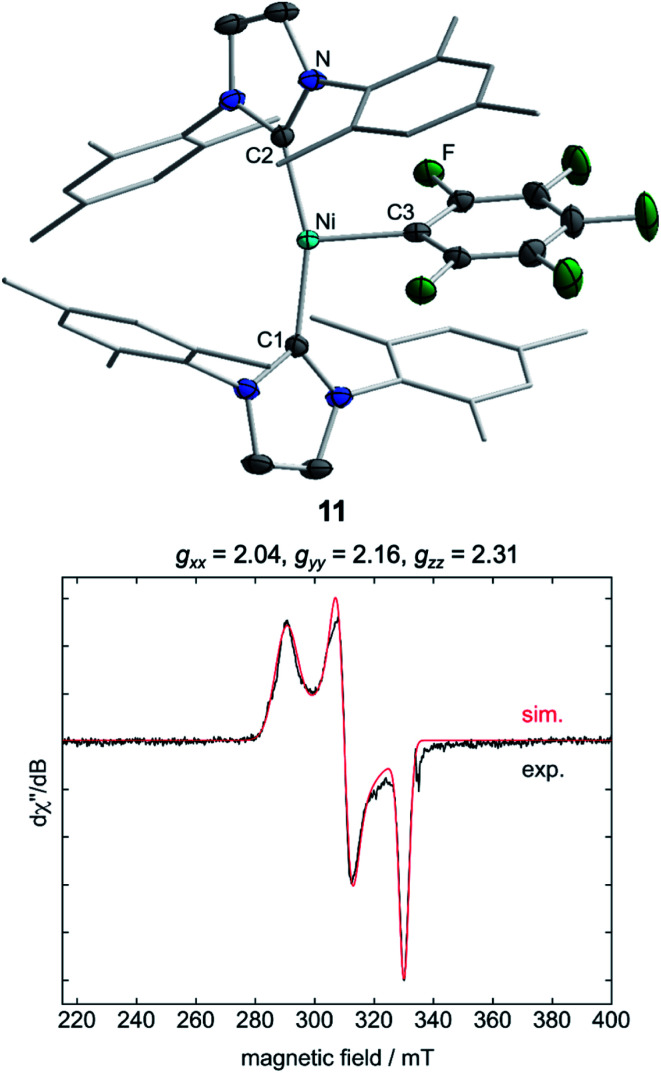
Molecular structure of *trans*-[Ni^I^(Mes_2_Im)_2_(C_6_F_5_)] (**11**) (top) in the solid state (ellipsoids drawn at the 50% probability level) and EPR spectrum at −203 °C of the isolated compound **11** (bottom). Hydrogen atoms are omitted for clarity.

**Fig. 8:**
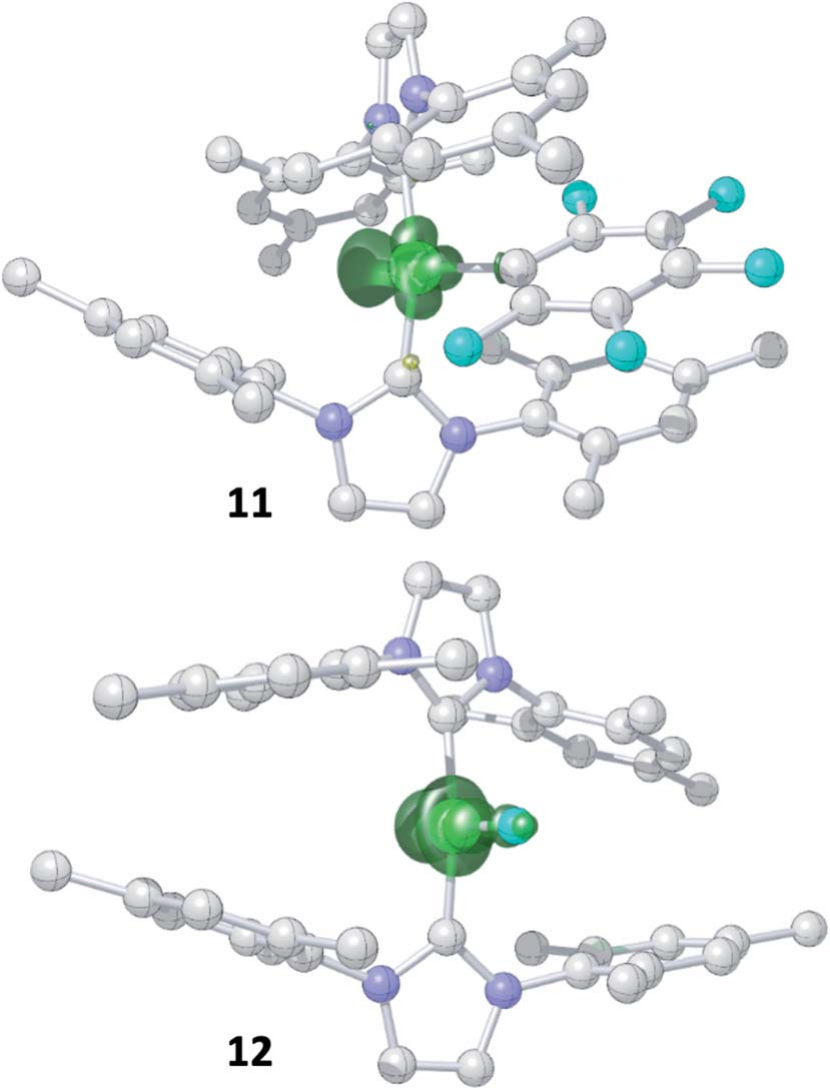
Spin density plots for *trans*-[Ni^I^(Mes_2_Im)_2_(C_6_F_5_)] (**11**) (top) and *trans*-[Ni^I^(Mes_2_Im)_2_(F)] (**12**) (bottom) (isovalue 0.0075 a_0_^−3^; hydrogen atoms are omitted for clarity).

**Scheme 6:**
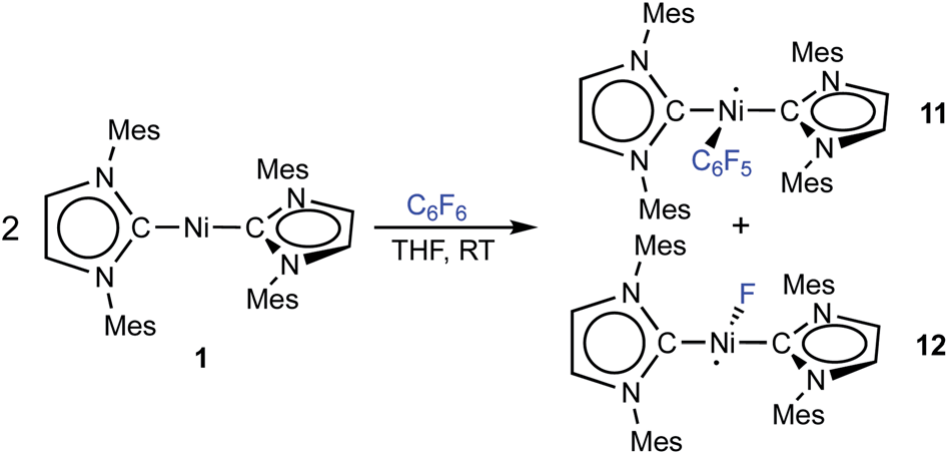
One-electron oxidative addition of C_6_F_6_ to [Ni(Mes_2_Im)_2_] (**1**) to yield the metal radicals *trans*-[Ni^I^(Mes_2_Im)_2_(C_6_F_5_)] (**11**) and *trans*-[Ni^I^(Mes_2_Im)_2_(F)] (**12**).

Storing the concentrated hexane mother liquor of the extract from the isolation of **9** (*vide supra*) for 3 days at −30 °C led to crystallization of the remaining C–F bond insertion product *trans*-[Ni(Mes_2_Im)_2_(F)(C_6_F_5_)] (**3**) and a novel nickel(i) complex *trans*-[Ni^I^(Mes_2_Im)_2_(C_6_F_5_)] (**11**) as yellow (**3**) and orange (**11**) crystals, respectively, which were manually separated in a glovebox (see ESI, Fig. S8†). The paramagnetic compound **11** was characterized by elemental analysis, EPR spectroscopy and X-ray diffraction. Determination of the room-temperature magnetic moment of **11** in solution (Evans method) gave a *μ*_eff_ value of 1.80 *μ*_B_, which is consistent with the presence of one unpaired electron. The molecular structure (Fig. 7, top, Table 1; see also ESI, Table S2 and Fig. S39†) and the EPR spectrum (Fig. 7, bottom) of **11** confirm that this complex is a three-coordinate nickel(i) radical. Simulation of the EPR spectrum of **11** gave a *g* tensor of *g*_*xx*_ = 2.04, *g*_*yy*_ = 2.16 and *g*_*zz*_ = 2.31, which was also observed in the EPR spectrum of the crude reaction mixture of **1** and C_6_F_6_ (Fig. 2). With the experimentally obtained *g* tensors and the molecular structure of the radical species **11** in hand, we carried out computational studies of the electronic properties of complex **11** and a likely radical counterpart from the reaction of **1** and C_6_F_6_, [Ni^I^(Mes_2_Im)_2_(F)] (**12**) (Fig. 8). Both complexes **11** and **12** would be the result of a one-electron oxidative addition reaction of two equiv. of **1** with one equiv. C_6_F_6_ (Scheme 6).

Molecular geometries, electronic structures and EPR parameters (*g* tensors) were thus calculated for the metal radicals *trans*-[Ni^I^(Mes_2_Im)_2_(C_6_F_5_)] (**11**) and *trans*-[Ni^I^(Mes_2_Im)_2_(F)] (**12**) (Fig. 8) in order to connect the experimentally observed EPR spectra from the reaction mixture of **1** and C_6_F_6_ (Fig. 2), the EPR spectra of the isolated compound **11**, and the corresponding isotropic *g* tensor components with the assigned structure of **11** (Fig. 7).

According to DFT calculations, complexes **11** and **12** are *C*_2_-symmetric doublet ground state species. The spin density is located at the metal center and the unpaired electron resides in an s/d_*z*^2^_-type orbital, yielding ^2^A electronic ground states (Fig. 8). Calculated and experimental *g* tensor components are in good agreement for species **11**, with a maximum difference of 0.03 in *g*_*zz*_. With the largest deviation being 0.08 for **12**, the agreement is still reasonable (Table 3).

**Table 3:** Comparison of experimental and calculated *g* tensors for species **11** and **12**

Compound	DFT/Exp^a^	*g* tensor components^b^		
		*g*_*xx*_	*g*_*yy*_	*g*_*zz*_
**11**	Exp. (isol.)^c^	2.04	2.16	2.31
	Exp. (react. mix.)^d^	2.04	2.17	2.32
	DFT	2.06	2.17	2.29
**12**	Exp. (react. mix.)^d^	1.93	2.46	2.64
	DFT	2.01	2.42	2.57

a The experimental *g*-tensor components are reorganized in ascending order from *g*_*xx*_ to *g*_*zz*_.

b EPR parameter have been calculated using DFT. The calculated values are rounded to match the number of digits of the experimental values.

c Exp. (isol.): see Fig. 7 (bottom).

d Exp. (react. mix.): see Fig. 2**I** and **II** (**I** corresponds to compound **12**; **II** corresponds to compound **11**).

To provide further evidence for the existence of *trans*-[Ni^I^(Mes_2_Im)_2_(C_6_F_5_)] (**11**) and *trans*-[Ni^I^(Mes_2_Im)_2_(F)] (**12**), we attempted to synthesize these complexes independently. The reaction of [Ni(Mes_2_Im)_2_][BF_4_] (**8**) with CsF led to a mixture of two complexes, which we were not able to separate. One of them was identified *via*^19^F{^1^H} NMR spectroscopy as *trans*-[Ni(Mes_2_Im)_2_(F)_2_] (**9**) (^19^F{^1^H} NMR resonance at −560 ppm), and the resulting mixture reveals an EPR resonance with *g* tensors (*g*_*xx*_ = 2.05, *g*_*yy*_ = 2.42, *g*_*zz*_ = 2.61) which are close to the *g*-tensors calculated for *trans*-[Ni^I^(Mes_2_Im)_2_(F)] (**12**). We are thus confident that the second metal radical obtained in the reaction mixture is the monofluoride complex *trans*-[Ni^I^(Mes_2_Im)_2_(F)] (**12**).

**Scheme 7:**
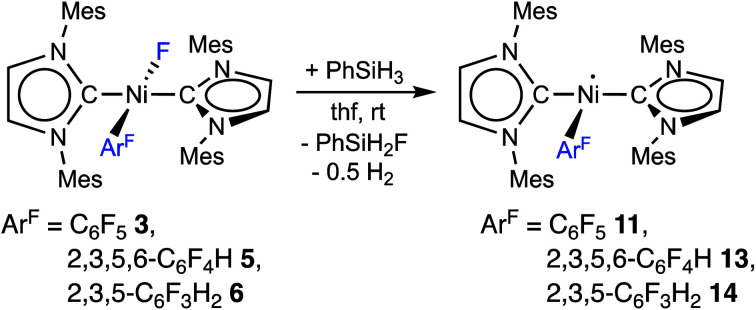
Synthesis of the metal radicals [Ni^I^(Mes_2_Im)_2_(C_6_F_5_)] (**11**), [Ni^I^(Mes_2_Im)_2_(2,3,5,6-C_6_F_4_H)] (**13**) and [Ni^I^(Mes_2_Im)_2_(2,3,5-C_6_F_3_H_3_)] (**14**).

The complex *trans*-[Ni^I^(Mes_2_Im)_2_(C_6_F_5_)] (**11**) as well as related *trans*-[Ni^I^(Mes_2_Im)_2_(2,3,5,6-C_6_F_4_H)] (**13**) and *trans*-[Ni^I^(Mes_2_Im)_2_(2,3,5-C_6_F_3_H_2_)] (**14**) can be synthesized from the reaction of *trans*-[Ni(Mes_2_Im)_2_(F)(Ar^F^)] (Ar^F^ = C_6_F_5_**3**, 2,3,5,6-C_6_F_4_H **5**, 2,3,5-C_6_F_3_H_2_**6**) with PhSiH_3_ (Scheme 7, see also ESI Fig. S9 and S10†).^14^

**Fig. 9:**
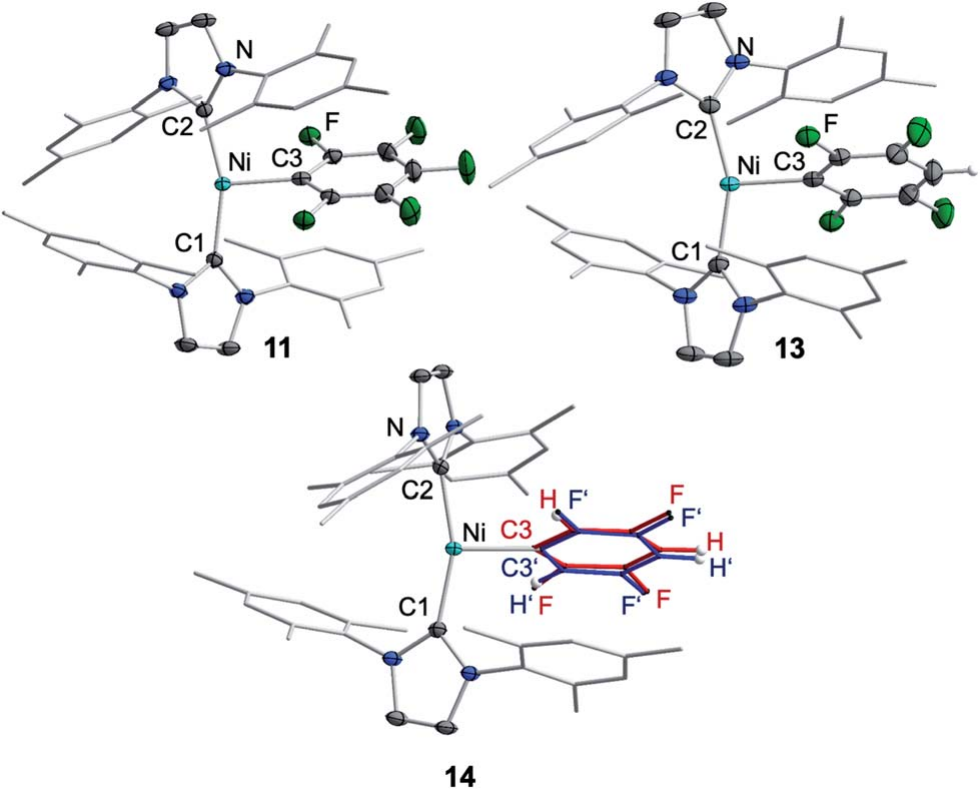
Molecular structures of *trans*-[Ni^I^(Mes_2_Im)_2_(C_6_F_5_)] (**11**) (top left), *trans*-[Ni^I^(Mes_2_Im)_2_(2,3,5,6-C_6_F_4_H)] (**13**) (top right) and *trans*-[Ni^I^(Mes_2_Im)_2_(2,3,5-C_6_F_3_H_2_)] (**14**) (bottom) in the solid state (ellipsoids drawn at the 50% probability level). Hydrogens atoms (with exception of the protons at fluoroarene rings) are omitted for clarity. Because of disorder of the fluoroaryl ligand of **14**, the ligand is represented by a ball and stick model in two different colors for clarity.

The metal radicals were characterized by elemental analysis, IR and EPR spectroscopy as well as single-crystal X-ray diffraction. All compounds are stable in the solid state as well as in solution for several days. If the reactions are performed in an NMR tube and followed by ^1^H and ^19^F{^1^H} NMR spectroscopy (see ESI; Fig. S9 and S10†), the resonances for the Mes_2_Im, pentafluorophenyl and fluoride ligands vanish, indicating the formation of a paramagnetic species. For complexes of the type *trans*-[Ni(NHC)_2_(H)(Ar^F^)], we expect hydride resonances in the region of *ca.* −13 ppm in the ^1^H NMR spectrum,^9*b*,*d*^ and a strong absorption in the IR spectrum in the region between 1600 and 2200 cm^−1^^15^ (we expect the Ni–H stretch to be at *ca.* 1850 cm^−1^ based on DFT calculations). However, such signals were absent for **11**, **13** and **14**. Thus, although complexes of the type *trans*-[Ni^I^(Mes_2_Im)_2_(Ar^F^)] cannot easily be distinguished from the corresponding hydride complexes *trans*-[Ni^I^(Mes_2_Im)_2_(H)(Ar^F^)] by X-ray diffraction (see below), we are confident that **11**, **13** and **14** are the metal radicals. Crystals of *trans*-[Ni^I^(Mes_2_Im)_2_(C_6_F_5_)] (**11**), *trans*-[Ni^I^(Mes_2_Im)_2_(2,3,5,6-C_6_F_4_H)] (**13**) and *trans*-[Ni^I^(Mes_2_Im)_2_(2,3,5-C_6_F_3_H_2_)] (**14**) suitable for X-ray diffraction (Fig. 9, Table 1; see also ESI Table S2 and Fig. S39–S41†) were obtained by storing saturated solutions of these compounds either in pentane or hexane at −30 °C. Complexes **11–13** adopt a distorted T-shaped structure, in which the NHC ligands occupy mutually *trans* positions. Due to the absence of the fluoride ligand, **11**, **13** and **14** exhibit shortened Ni–C distances to the fluoroaryl ligand and reduced C1–Ni–C2 angles compared to nickel(ii) complexes **3**, **4**, **5** and **6**, which is also a further indication of the absence of a metal hydride. The data is in line with the data observed for [Ni^I^(P^i^Pr_3_)_2_(C_6_F_5_)] reported by Johnson and co-workers previously (Table 1, see also ESI Table S2†).^16^ EPR spectra of compounds **11**, **13** and **14** were recorded in frozen thf solutions and reveal similar *g* tensors for the complexes, which are in good agreement with the calculated parameters (see ESI, Fig. S11–S13 and Table S4†).

Thus, the reaction of **1** with C_6_F_6_ affords the insertion product *trans*-[Ni(Mes_2_Im)_2_(F)(C_6_F_5_)] (**3**) in approximately 20% isolated yield, the difluoride complex *trans*-[Ni(Mes_2_Im)_2_(F)_2_] (**9**) in approximately 17% isolated yield, the three-coordinate nickel(i) metal radicals *trans*-[Ni^I^(Mes_2_Im)_2_(C_6_F_5_)] (**11**) (isolated yield: 10%), *trans*-[Ni^I^(Mes_2_Im)_2_(F)] (**12**) (not isolated), and a small amount of a decomposition product, *i.e.*, a dark green precipitate which was not characterized. *Trans*-[Ni^I^(Mes_2_Im)_2_(F)] (**12**) was not isolated; it was only observed in the EPR spectra after 5 s at −78 °C, and the signals vanish after about 10 s during the course of the reaction. Further investigation of the hexane mother liquor of the reaction of **1** and C_6_F_6_ revealed that the bis(aryl) nickel(ii) complex [Ni(Mes_2_Im)_2_(C_6_F_5_)_2_] (**15**) remains in solution and was identified in the reaction mixture by ^19^F{^1^H} NMR spectroscopy. The radical species **11** and **12** were identified by EPR spectroscopy in a frozen thf solution at −78 °C (Fig. 2). The diamagnetic products *trans*-[Ni(Mes_2_Im)_2_(F)(C_6_F_5_)] (**3**), *trans*-[Ni(Mes_2_Im)_2_(F)_2_] (**9**), and *trans*-[Ni(Mes_2_Im)_2_(C_6_F_5_)_2_] (**15**) were identified by NMR spectroscopy (see Fig. S14 of the ESI†).

To expand our study to less fluorinated systems, we reacted **1** with pentafluorobenzene. After 48 h at room temperature, the ^19^F{^1^H} and ^19^F NMR spectra recorded in C_6_D_6_ reveal the formation of the C–F bond activation product *trans*-[Ni(Mes_2_Im)_2_(F)(C_6_F_4_H)] (**5**), the nickel difluoride complex [Ni(Mes_2_Im)_2_(F)_2_] (**9**), and the corresponding bis(aryl) nickel(ii) complex [Ni(Mes_2_Im)_2_(C_6_F_4_H)_2_] (see ESI, Fig. S15†). Furthermore, an EPR spectrum of the frozen reaction mixture of **1** with pentafluorobenzene in thf recorded after 5 s at −78 °C (see ESI, Fig. S16†) revealed resonances for three different products, one of which is in accordance with *trans*-[Ni^I^(Mes_2_Im)_2_(F)] (**12**) and another has the same *g* tensor as observed for isolated [Ni^I^(Mes_2_Im)_2_(C_6_F_4_H)] (**13**). Thus, the reaction of **1** with C_6_F_5_H also follows a radical reaction mechanism akin to the reaction of **1** with C_6_F_6_ below.

### Mechanistic investigations

Experimental investigations and DFT studies reported previously^9*a*^ for the reaction of [Ni_2_(^i^Pr_2_Im)_4_(μ-(η^2^:η^2^)-COD)] and [Ni(^i^Pr_2_Im)_2_(η^2^-C_2_H_4_)], used as source of [Ni(^i^Pr_2_Im)_2_] (**1ipr**), with C_6_F_6_ suggested a concerted mechanism for the insertion of **1ipr** into the C–F bond, and no indications for radical reactivity were obtained. As presented above, however, paramagnetic complexes clearly emerge in the reaction of **1** and C_6_F_6_. To obtain further insight, we performed a quantum-chemical investigation (COSMO(THF)-PBE0-D/def2-TZVP, for details see ESI†)^17^ on the reaction pathways of C_6_F_6_ with [Ni(Mes_2_Im)_2_] (**1**) and with the sterically less encumbered [Ni(^i^Pr_2_Im)_2_] (**1ipr**).

**Scheme 8:**
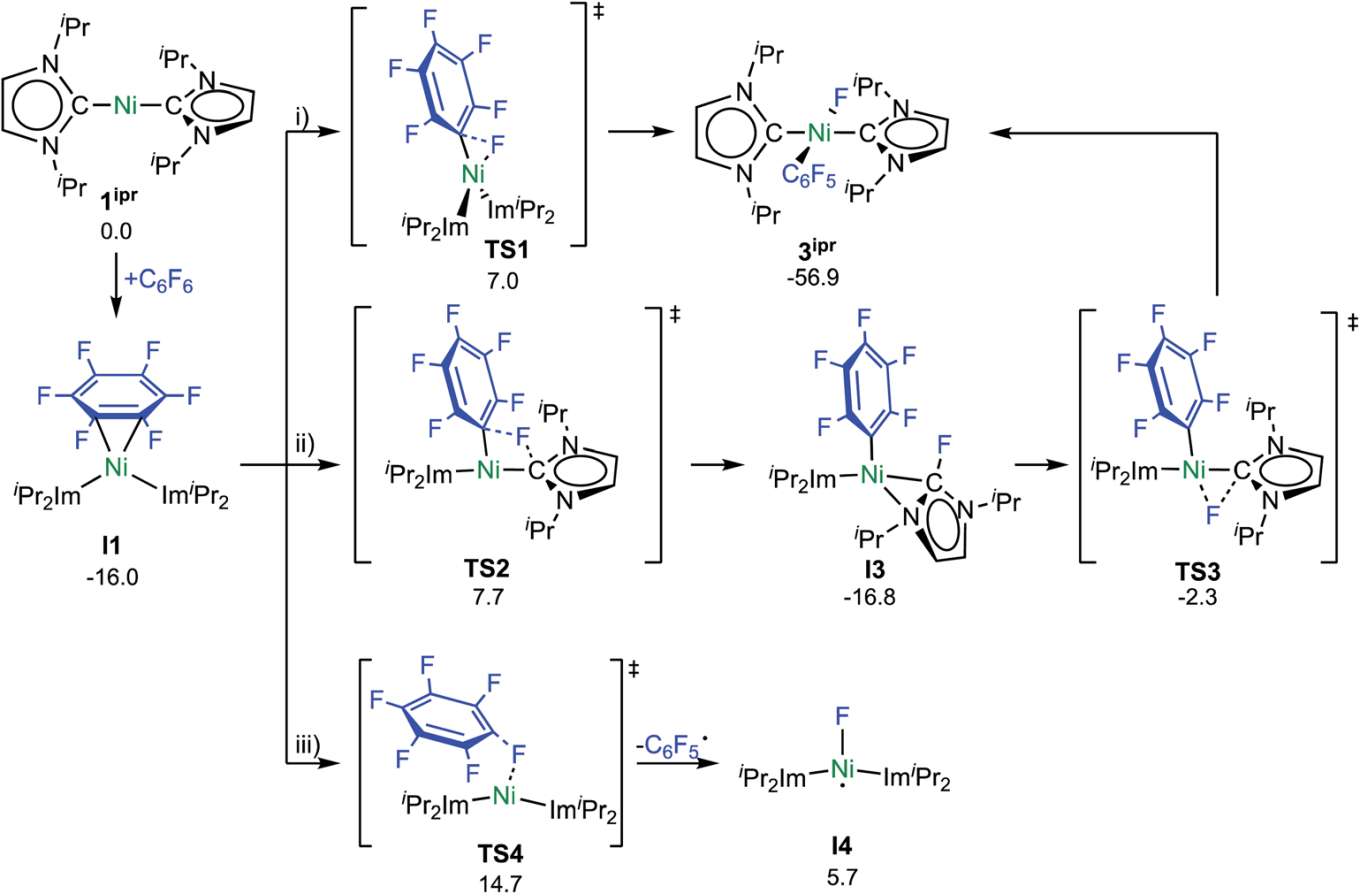
Calculated pathways for the C–F bond activation of C_6_F_6_ with **1ipr** (Δ*G*^298^ in kcal mol^−1^).

C–F bond activation in the latter reaction commences with the formation of a rather stable 16-electron η^2^ adduct between **1ipr** and C_6_F_6_ (**I1**, Scheme 8; see ESI, Fig. S17†). The DFT-optimized geometry of **I1** is in good agreement with the structure of the closely related complex [Ni(^i^Pr_2_Im)_2_(η^2^-C_10_F_8_)].^9*a*^ Three distinct reaction pathways are then possible. First, direct oxidative addition of the C–F bond to the nickel atom proceeds through **TS1** to yield the *trans* product **3ipr** with an effective activation barrier of Δ^‡^*G* = 23 kcal mol^−1^ relative to **I1** (see ESI, Fig. S18†). Alternative formation of the corresponding *cis*-[Ni(^i^Pr_2_Im)_2_(F)(C_6_F_5_)] (**I2**) and subsequent isomerization is kinetically disfavored (Δ^‡^*G*^eff^ = 27 kcal mol^−1^, see ESI Fig. S19 and S20†), as is dissociation of an NHC ligand (Δ*G*^298^ = 28 kcal mol^−1^, see Fig. S32†).

Second, NHC ligand cooperativity (see ESI; Fig. S21 and S23†) opens a kinetically competitive pathway to the *trans*-product **3ipr**, that is, addition of the C–F bond across the Ni–C^NHC^ bond through **TS2** to yield intermediate **I3**, in which coordination of the fluorinated NHC–F ligand to the nickel atom involves a bridging C–N bonding interaction. In **TS2**, the C_aryl_–F bond of 1.93 Å is strongly elongated compared to C_6_F_6_ (C_aryl_–F bond: 1.32 Å) and **TS1** (C_aryl_–F bond: 1.77 Å), while NHC–F bond formation is hardly visible (C/F distance: 2.40 Å). From **I3**, fluoride migration onto the nickel ion (**TS3**, with a low barrier of Δ^‡^*G* = 15 kcal mol^−1^) leads to **3ipr** with an overall barrier of Δ^‡^*G*^eff^ = 24 kcal mol^−1^. Third, homolytic C–F bond cleavage involves an effective barrier of Δ^‡^*G*^eff^ = 31 kcal mol^−1^ (**TS4**) and, hence, radical abstraction is kinetically disfavored here (see ESI, Fig. S22†).

**Scheme 9:**
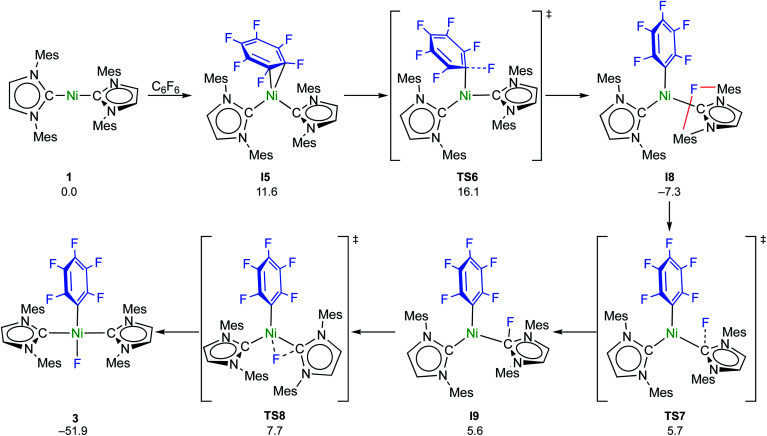
Calculated pathways for the heterolytic C–F bond cleavage of C_6_F_6_ by **1** and further reaction steps (Δ*G*^298^ in kcal mol^−1^).

C–F bond activation with the sterically more congested Mes–NHC complex **1** shows marked differences. Formation of the η^2^-C_6_F_6_ adduct **I5** (see ESI, Fig. S24†) is now endergonic by 12 kcal mol^−1^, and consecutive oxidative C–F bond addition *via***TS5** (Δ^‡^*G*^eff^ = 21 kcal mol^−1^, see ESI; Fig. S25†) leads to the *cis*-product **I6**. We attribute the endergonicity of the η^2^-C_6_F_6_ adduct formation (**I5**, ΔΔ*G* = 28 kcal mol^−1^ compared to the exergonic formation of **I1**) mainly to the increased steric demand of the mesityl groups. A trajectory to the *trans*-product is precluded by the steric demand of the mesityl substituents. NHC dissociation to yield [Ni(Mes_2_Im)(η^6^-C_6_F_6_)] and subsequent insertion into the C–F bond is associated with a large barrier (Δ^‡^*G*^eff^ = 34 kcal mol^−1^, see ESI; Fig. S32 and S33†) and is irrelevant here. Note that an alternative adduct formation stabilized by π-stacking interactions between C_6_F_6_ and one of the NHC mesityl substituents,^18^ such as **I7** (see ESI; Fig. S26†), is also endergonic and less favorable than **I5**. Furthermore, a “concerted” NHC-assisted process as in the ^i^Pr system does not exist. We found a multi-step sequence for the mesityl system instead (Scheme 9 and ESI; Fig. S27†), commencing with heterolytic C–F bond cleavage in **I5**, which exhibits a partially reduced C_6_F_6_ fragment (*q*_NPA_(C_6_F_6_) = −0.69). The fluoride anion expelled from the nickel coordination sphere is loosely held within the cleft formed by the mesityl substituents in **I8**. A similar stabilizing association of a fluoride anion by the methyl groups of mesityl substituents has been reported by Macgregor *et al.* for the C–F bond activation step in hydrodefluorination reactions.^19^ Formation of the *trans*-product **3** from here involves binding to the carbene carbon atom and subsequent F-shift onto the Ni center. The overall path involves a low effective barrier of 16 kcal mol^−1^ (**TS6**).

**Scheme 10:**
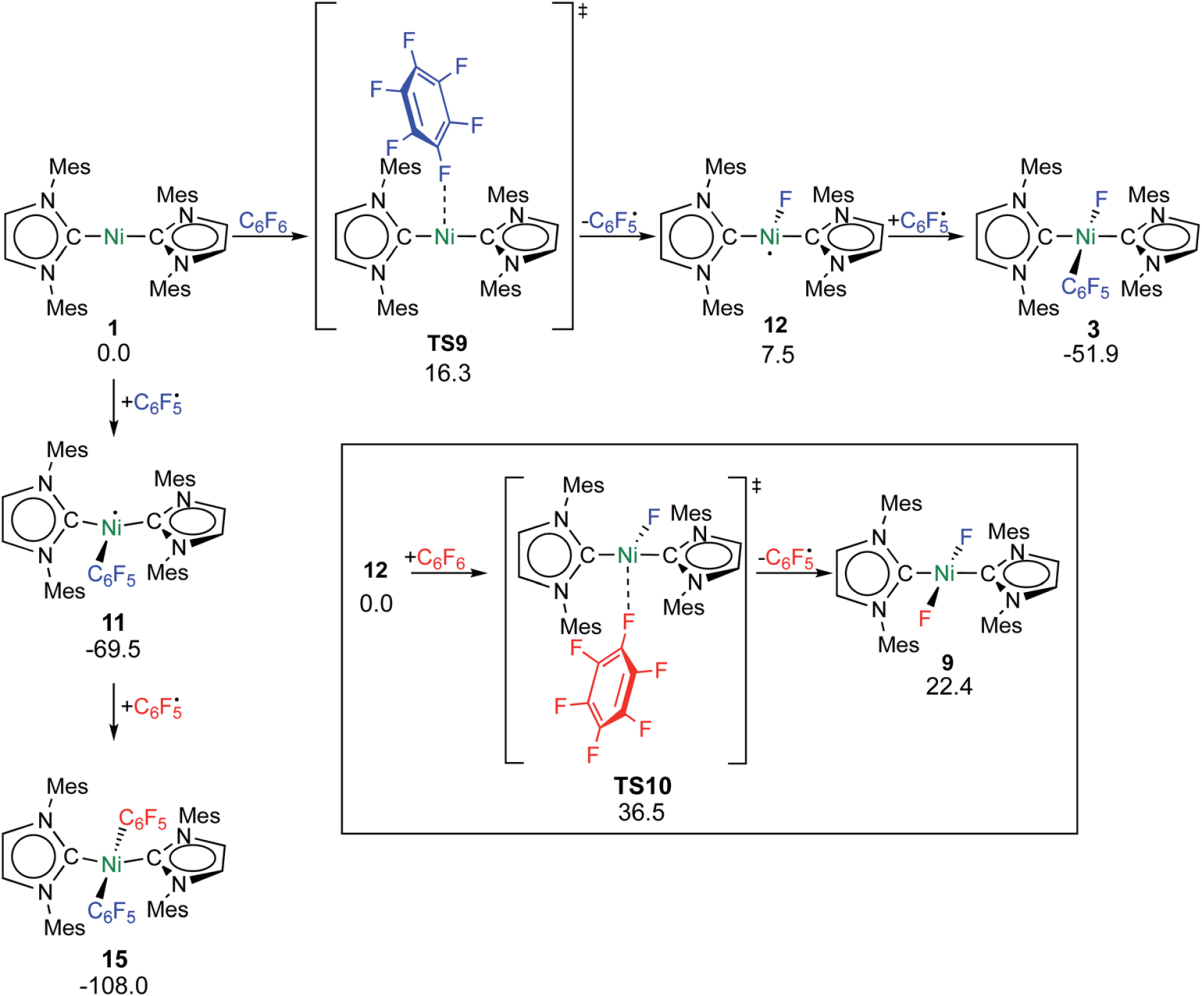
Calculated pathways for the homolytic C–F bond cleavage of C_6_F_6_ by **1** and further radical reaction steps (Δ*G*^298^ in kcal mol^−1^; energies of **TS10** and **9** are given relative to **12** + C_6_F_6_).

Fluorine radical abstraction to yield 
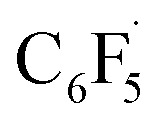
 and radical complex **12***via***TS9** is slightly endergonic and exhibits a barrier of 16 kcal mol^−1^ (Scheme 10 and ESI, Fig. S26 and S28†). Recombination of 
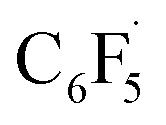
 and **12** to **3** then provides a large thermodynamic driving force. Alternative addition of 
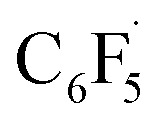
 to the initial complex **1** to yield radical species **11** is also a highly exergonic process (−69.5 kcal mol^−1^), as well as addition of a second equivalent of 
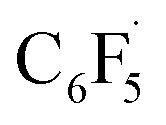
 to yield **15** (−108.0 kcal mol^−1^). Endergonic formation of difluoride complex **9** from **12** and another equiv. of C_6_F_6_, can be compensated by consumption of 
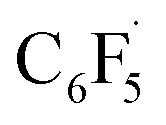
; however, a second fluorine abstraction step is prevented by the high kinetic barrier of 37 kcal mol^−1^*via***TS10** (see ESI; Fig. S29†). The mechanism for the formation of **9** remains obscure to us thus far. We compute the ligand exchange reaction **3** + **3** → **9** + **15** to be exceedingly endergonic (25.7 kcal mol^−1^), and also the disproportionation reactions of radicals **11** and **12** yielding **1** + **15** (31.0 kcal mol^−1^) or **1** + **9**, (14.9 kcal mol^−1^), are unlikely to contribute to the formation of **9** (see ESI, Fig. S30†). A dinuclear complex [{Ni(Mes_2_Im)_2_}_2_(μ-(η^2^:η^2^)-C_6_F_6_)], which would be an intermediate for an one-electron oxidative addition, is too high in energy to be considered (35 kcal mol^−1^, see ESI; Fig. S31†). Hence both, the radical pathway and the NHC-assisted multistep pathway represent kinetically competitive C–F bond activation steps in the reaction with [Ni(Mes_2_Im)_2_] (**1**).

## Discussion

It is now well established that nickel(0) complexes with phosphine, carbene, and even some nitrogen ligands undergo C–F oxidative addition with perfluoroarenes to yield complexes *trans*-[Ni(L)_2_(F)(C_6_F_5_)].^1,9,20^ Although the lack of clean kinetics for many of the C–F oxidative additions indicate complex mechanistic scenarios, there were strong indications that the conversion of C_6_F_6_ to the aryl fluoride complex follows the same type of mechanism as observed for typical C–H activation reactions of benzene. It has been demonstrated, for nickel NHC and phosphine complexes, that the first stage of C–F oxidative addition is the η^2^-coordination of the fluoroarene.^1*g*,9*a*,21,22^ The introduction of fluorine substituents on the arene results in a lower lying LUMO, which renders the fluorinated arene a better electron acceptor compared to H-arenes and makes the reaction of electron-poor C_6_F_6_ with an electron-rich, suitable nickel precursor more exothermic. The fluoroarene of [Ni(L)_2_(η^2^-C_6_F_6_)] is ene–diene distorted, and the arene fluoride substituents are bent out of the plane, as observed for **I1** and **I5**. Subsequent C–F oxidative addition is strongly exothermic for *trans*-[Ni(^i^Pr_2_Im)_2_(F)(C_6_F_5_)] (Δ*G*^298^ = −57 kcal mol^−1^) and *trans*-[Ni(Mes_2_Im)_2_(F)(C_6_F_5_)] (Δ*G*^298^ = −52 kcal mol^−1^). Computational studies reported previously^9*a*,23^ of the reaction pathways have supported the idea of concerted mechanisms involving a σ-complex as a three-center transition state between the C_6_F_6_ carbon and fluorine atoms and the transition metal atom. The transition state structures typically show limited elongation of the C–F bond and interaction of the electron-rich transition metal ion with the C–F σ* orbital leads to C–F bond breaking and formation of the M–C and M–F bond. We have demonstrated now for [Ni_2_(^i^Pr_2_Im)_4_(μ-(η^2^:η^2^)-COD)] and the related [Ni(^i^Pr_2_Im)_2_] (**1ipr**) synthon complexes that C–F bond activation of C_6_F_6_ occurs *via* both a concerted and an NHC-assisted pathway, as both are associated with very similar kinetic barriers of Δ^‡^*G*^eff^ = 23 kcal mol^−1^ for the concerted and of Δ^‡^*G*^eff^ = 24 kcal mol^−1^ for the NHC-assisted pathway. This situation will probably change if other substrates with other leaving groups, such as partially fluorinated arenes, fluoropyridines or other aryl halides, are involved in the reaction with the nickel complex; however, our calculations demonstrate that both reaction paths are feasible, at least for fluoroarenes.

The direction of the concerted oxidative addition in **TS1** to give the *trans* product is rather unusual.^24^ For the oxidative addition of A–B to d^10^-ML_2_ the important orbital interactions of the transition state are those between the filled σ(A–B) orbital and the empty d_σ_-type orbital of the metal, leading to electron donation from A–B to the metal center, and a second interaction between the filled d_π_-orbital of the metal and the σ*(A–B), leading to electron transfer from the metal to the ligand. Strong back-donation will lead to fission of the A–B bond. This back-donation is strongest if A–B lies within the bent-d^10^-ML_2_ plane and the σ*(A–B) orbital can interact with the d_*x*^2^–*y*^2^_ orbital (actually a d–p hybrid orbital), which is pointing at the two ligands L.^24^

However, it was also shown previously that concerted oxidative addition reactions may take place through a nonplanar transition state structure even for non-polar substrates with dihedral angle between ML_2_ and M(A–B) planes larger than 70°.^25^ It was demonstrated that this nonplanar transition state is connected to the planar product on the singlet surface and suggested that steric rather than electronic factors are responsible for the nonplanar transition state structure. Martin *et al.*,^25*c*^ for example, calculated at the B3LYP/LanL2DZ-level of theory a nonplanar transition state for the oxidative addition of C_6_H_5_–I to [Pd(dmpe)] (dmpe = bis{dimethylphosphino}ethane), in which the P–Pd–P and C–Pd–I planes are almost perpendicular to one another. Another example was provided by Jones *et al.*^25*d*^ for the oxidative addition of the C–CN σ-bond of organonitriles to the low-valent nickel complex [Ni(dmpe)]. The C–C–N plane of the transition state (calculated at the B3LYP/6-31G(d,p)-level of theory), which leads to C–CN bond cleavage, is rotated by 38° relative to the P–Ni–P plane.

The η^2^(C,C)-bonded complex [Ni(^i^Pr_2_Im)_2_(η^2^-C_6_F_6_)] (**I1**) is also the crucial reaction intermediate for the NHC-assisted pathway. The key step here is the addition of the C–F bond across the Ni–C_NHC_ bond and, thus, the unoccupied NHC p_π_-orbital plays a central role for this pathway as intramolecular fluoride acceptor. Fluoride transfer from the arene to the NHC leads to a η^2^-fluoro-imidazolyl intermediate (**I3**; Scheme 8) which rearranges with a second fluoride transfer step from the NHC to the nickel atom to give *trans*-[Ni(^i^Pr_2_Im)_2_(F)(C_6_F_5_)] (**3ipr**).

A phosphine-assisted process has been proposed before for the C–F bond activation of pentafluoropyridine with [Ni(PR_3_)_2_], based on the experimental observation of an unusual selectivity for the insertion into the 2-position of C_5_NF_5_ and on DFT calculations.^26^ However, another study performed on the reaction of pentafluoropyridine with [Ni(PEt_3_)_2_] suggested that pathways other than a concerted oxidative addition or a phosphine-assisted pathway account for the unusual selectivity.^27^ The detailed experimental analysis of the reactivity of a [Ni(PEt_3_)_2_] precursor with perfluoropyridine demonstrated the formation of a mononuclear adduct [Ni(PEt_3_)_2_(η^2^-C_5_F_5_N)], of dinuclear adducts [{Ni(PEt_3_)_2_}_2_)(μ-(η^2^:η^2^)-C_5_F_5_N)], some of which exhibit C–F bond activation, and a nickel(i) radical species [Ni(PEt_3_)_2_(2-C_5_F_4_N)]. Other heteroatom-assisted C–F bond activation processes have also been proposed for other metals mainly including boryl or silyl moieties.^28^

Despite precedent in the oxidative addition of other aryl carbon–halide bonds to nickel,^29,30^ there is only little experimental evidence for the involvement of radicals in C–F bond activation processes. It is known that some polyfluoro pyridines react with [Ni(PR_3_)_2_] to yield EPR-active complexes as likely intermediates,^16,27^ and some studies on C–F bond activation have shown unusual products with highly-fluorinated arenes that may be indicative of radical pathways.^16,22,31^ However, the clear identification of radical intermediates has not been possible so far and alternate mechanisms cannot be ruled out. Although DFT calculations were performed to examine the traditional concerted oxidative addition and phosphine-assisted pathways for C–F bond activation, radical pathways involving Ni(i) intermediates were rarely considered computationally.

Thus, the reaction of **1** with different fluoroarenes leads to nickel insertion into the C–F bond to give the nickel fluoroaryl fluoride complexes *trans*-[Ni(Mes_2_Im)_2_(F)(Ar^F^)], but EPR spectroscopy also provided evidence that at least three paramagnetic species are intermediates or products of the reaction of C_6_F_6_ with **1**. We provide evidence that simple electron transfer from [Ni(Mes_2_Im)_2_] (**1**) to C_6_F_6_, often considered as the first step in radical oxidative additions at nickel,^29^ is unlikely to occur. The redox potentials are not in line with intermolecular electron transfer to yield [Ni(Mes_2_Im)_2_]^+^ and C_6_F_6_^−^ and the EPR resonance of [Ni(Mes_2_Im)_2_]^+^, which has been established for the authentic complex [Ni(Mes_2_Im)_2_][BF_4_] (**8**), was not detected in the reaction mixture. Furthermore, many diamagnetic and radical products of the reaction of [Ni(Mes_2_Im)_2_] (**1**) to C_6_F_6_ were identified, namely the insertion product *trans*-[Ni(Mes_2_Im)_2_(F)(C_6_F_5_)] (**3**), the difluoride complex *trans*-[Ni(Mes_2_Im)_2_(F)_2_] (**9**), the bis(aryl) complex *trans*-[Ni^II^(Mes_2_Im)_2_(C_6_F_5_)_2_] (**15**), the nickel(i) complex *trans*-[Ni^I^(Mes_2_Im)_2_(C_6_F_5_)] (**11**), and the metal-centered radical *trans*-[Ni^I^(Mes_2_Im)_2_(F)] (**12**). DFT calculations performed on the reaction of [Ni(Mes_2_Im)_2_] (**1**) with C_6_F_6_ explain the occurrence of the radical species observed. Both an NHC-assisted and a radical process are kinetically equally favored routes for this reaction. Fluorine radical abstraction from C_6_F_6_ by **1** is associated with a barrier of only 16 kcal mol^−1^ and subsequent radical recombination steps provide the thermodynamic driving force required.

Matsubara *et al.* and Louie *et al.* reported the clean isolation of T-shaped three-coordinate radical species [Ni^I^(NHC)_2_(X)] (X = Cl, Br, I; NHC = Mes_2_Im, Dipp_2_Im) from the reaction of [Ni(NHC)_2_] with aryl halides.^30*a*,*b*,*d*^ We have demonstrated earlier that [Ni_2_(^i^Pr_2_Im)_4_(μ-(η^2^:η^2^)-COD)], a source of [Ni(^i^Pr_2_Im)_2_] (**1ipr**), reacts cleanly with aryl chlorides to yield the nickel(ii) complexes *trans*-[Ni(NHC)_2_(Cl)(Ar)].^32^ Our calculations show now that a trajectory to the *trans*-product by a concerted oxidative addition is precluded for [Ni(Mes_2_Im)_2_] (**1**) (and most probably also for [Ni(Dipp_2_Im)_2_]) by the steric demand of the mesityl substituents. As a consequence, other pathways such as electron transfer and radical abstraction must occur which are responsible for a limited or altered reactivity of complex [Ni(Mes_2_Im)_2_] (**1**) and analogues containing even more bulky *N*-aryl substituents compared to complexes of sterically less demanding NHCs. However, fluoride abstraction occurs for the reaction of **1** and C_6_F_6_ even at −78 °C to yield *trans*-[Ni^I^(Mes_2_Im)_2_(C_6_F_5_)] (**11**) and *trans*-[Ni^I^(Mes_2_Im)_2_(F)] (**12**). The latter is, in contrast to the complexes of the heavier homologues, very reactive and has defied thus far isolation. In turn, the complexes *trans*-[Ni^I^(Mes_2_Im)_2_(C_6_F_5_)] (**11**), [Ni^I^(Mes_2_Im)_2_(2,3,5,6-C_6_F_4_H)] (**12**) and [Ni^I^(Mes_2_Im)_2_(2,3,5-C_6_F_3_H_2_)] (**13**) seem to be much more stable than [Ni^I^(NHC)_2_(C_6_H_5_)] and have been synthesized and characterized. The increased stability of [Ni^I^(Mes_2_Im)_2_(2,3,5,6-C_6_F_4_H)] (**12**) can be explained by the increased Ni–C_Ar_ bond strength of the fluoroaryl ligand with respect to C_6_H_5_.^33^

Nelson and Maseras^34^ reported computational investigations of the reaction of [Ni(NHC)_2_] complexes with aryl halides Ph–X (X = Cl, Br, I) and demonstrated that steric effects determine the mechanism. Small NHC ligands (NHC = Me_2_Im^Me^) favor concerted oxidative addition *via* a η^2^(C,C) π-coordinated intermediate leading to *trans*-[Ni^II^(NHC)_2_(X)(Ar)] complexes whereas larger NHC ligands (*e.g.* NHC = Mes_2_Im) lead to halide abstraction to form [Ni^I^(X)(NHC)_2_] and a phenyl radical. We confirm here, by means of experiment and theory, that [Ni(NHC)_2_] complexes of sterically less demanding NHCs favor the reaction with fluoroarenes *via* a concerted oxidative addition proceeding through an η^2^(C,C) intermediate, and that for the bulkier NHC Mes_2_Im, C–F bond activation is achieved more easily by fluorine atom abstraction. However, for both mechanisms, we found an NHC-assisted pathway which is competitive, that accounts for the formation of diamagnetic products by a C–F bond activation step across the Ni–C_NHC_ bond. NHC-assisted pathways play an important role for complexes of both sterically demanding and less bulky NHC ligand. We believe that this dual reaction pathway concept, including NHC-assisted reaction pathways, should be of general importance and widely applicable for the reactivity of NHC transition metal complexes.

## Conclusions

We present herein a detailed account of the C–F bond activation of polyfluoroaromatics, especially of C_6_F_6_ using the nickel(0) complex [Ni(Mes_2_Im)_2_] (**1**). The reaction of **1** with different fluoroarenes leads to insertion of nickel into the C–F bond of the fluoroarene to give the nickel(ii) complexes *trans*-[Ni(Mes_2_Im)_2_(F)(Ar^F^)] (Ar^F^ = 4-CF_3_-C_6_F_4_**2**, C_6_F_5_**3**, 2,3,5,6-C_6_F_4_N **4**, 2,3,5,6-C_6_F_4_H **5**, 2,3,5-C_6_F_3_H_2_**6**, 3,5-C_6_F_2_H_3_**7**) in good to fair yields with the exception of the formation of the pentafluorophenyl complex *trans*-[Ni(Mes_2_Im)_2_(F)(C_6_F_5_)] (**3**) (less than 20%). Whereas the C–F bond activation process of C_6_F_6_ using [Ni(^i^Pr_2_Im)_2_] (**1ipr**) follows a concerted or NHC-assisted mechanism to give the insertion product *via* η^2^-coordinated intermediates, metal radical species were detected for the reaction of **1** with C_6_F_6_. EPR spectroscopy provided evidence that at least three paramagnetic products are intermediates or products of this reaction. The experiments reveal that simple electron transfer from [Ni(Mes_2_Im)_2_] (**1**) to C_6_F_6_ is unlikely to occur as (i) the redox potentials do not match for an electron transfer between [Ni(Mes_2_Im)_2_] (**1**) and C_6_F_6_ to give [Ni(Mes_2_Im)_2_]^+^ and C_6_F_6_^−^, and (ii) the EPR resonance for [Ni(Mes_2_Im)_2_]^+^, as established for the stable, isolated complex [Ni(Mes_2_Im)_2_][BF_4_] (**8**), was not detected in the reaction mixture. Several other byproducts were identified aside from the insertion product **3**, namely the difluoride complex *trans*-[Ni(Mes_2_Im)_2_(F)_2_] (**9**), the bis(aryl) complex *trans*-[Ni^II^(Mes_2_Im)_2_(C_6_F_5_)_2_] (**15**), the structurally-characterized nickel(i) complex *trans*-[Ni^I^(Mes_2_Im)_2_(C_6_F_5_)] (**11)** and the metal radical *trans*-[Ni^I^(Mes_2_Im)_2_(F)] (**12**). Complex **11** and related complexes [Ni^I^(Mes_2_Im)_2_(2,3,5,6-C_6_F_4_H)] (**13**) and [Ni^I^(Mes_2_Im)_2_(2,3,5-C_6_F_3_H_2_)] (**14**) were synthesized and characterized independently from the reaction of *trans*-[Ni(Mes_2_Im)_2_(F)(Ar^F^)] with PhSiH_3_.

DFT calculations were performed on the insertion of [Ni(^i^Pr_2_Im)_2_] (**1ipr**) and [Ni(Mes_2_Im)_2_] (**1**) into the C–F bond of C_6_F_6_, which explain the formation of radical species for the reaction with [Ni(Mes_2_Im)_2_] (**1**). For [Ni(^i^Pr_2_Im)_2_] (**1ipr**), the crucial reaction intermediate is an η^2^(C,C)-bonded complex [Ni(^i^Pr_2_Im)_2_(η^2^-C_6_F_6_)], from which two favorable pathways with almost identical barriers, *i.e.*, a concerted oxidative addition pathway and a NHC-assisted pathway, lead to the formation of *trans*-[Ni(^i^Pr_2_Im)_2_(F)(C_6_F_5_)]. For [Ni(Mes_2_Im)_2_] (**1**), an NHC-assisted and a radical pathway were identified with similar kinetic barriers. Fluorine atom abstraction from C_6_F_6_ at [Ni(Mes_2_Im)_2_] (**1**) occurs *via* end-on attack of C_6_F_6_, while the key intermediate for the NHC-assisted pathway is the η^2^(C,C) intermediate [Ni(Mes_2_Im)_2_(η^2^-C_6_F_6_)]. The NHC-assisted pathway can be interpreted as heterolytic C–F bond cleavage to yield ionic intermediates *trans*-[Ni(Mes_2_Im)_2_(C_6_F_5_)]^+^F^−^, in which the fluoride anion is stabilized within the sphere of the *trans*-[Ni(Mes_2_Im)_2_(C_6_F_5_)]^+^ cation. Several fluoride transfer steps, *i.e.*, migration to the NHC, NHC rotation, and fluoride transfer to the metal cation lead to the formation of *trans*-[Ni(Mes_2_Im)_2_(F)(C_6_F_5_)] (**3**).

## Conflicts of interest

The authors declare no conflict of interest.

## Supplementary Material

SC-011-D0SC04237D-s001

SC-011-D0SC04237D-s002
