# Supplementary material for: Coligand role in the NHC nickel catalyzed C–F bond activation: investigations on the insertion of bis(NHC) nickel into the C–F bond of hexafluorobenzene
Source: Chem Sci. 2020 Oct 6;11(40):11009–23. doi: 10.1039/d0sc04237d (PMC8162383; doi:10.1039/d0sc04237d)
Supplement: SC-011-D0SC04237D-s001 [file SC-011-D0SC04237D-s001.pdf]

Electronic Supplementary Material (ESI) for Chemical Science.
This journal is © The Royal Society of Chemistry 2020

Electronic Supplementary Information for:

Coligand Role in the NHC Nickel catalyzed C–F Bond Activation: Investigations on the Insertion of bis(NHC) Nickel into the C–F Bond of Hexafluorobenzene

Maximilian W. Kuntze-Fechner [a], Hendrik Verplancke [b], Lukas Tendera [a], Martin Diefenbach [b], Ivo Krummenacher [a,c], Holger Braunschweig [a,c], Todd B. Marder [a,c], Max C. Holthausen [b,*], Udo Radius [a,*]

[a]Institute for Inorganic Chemistry, Julius-Maximilians-Universität Würzburg, Am Hubland, 97074 Würzburg, Germany.

[b]Institute for Inorganic and Analytical Chemistry, Goethe-Universität Frankfurt, Max-von-Laue-Strasse 7, 60438 Frankfurt, Germany.

[c]Institute for Sustainable Chemistry & Catalysis with Boron, Julius-Maximilians-Universität Würzburg, Am Hubland, 97074 Würzburg, Germany.

1) Additional Figures and Tables
2) Experimental Section
3) Crystallographic Details
4) Computational Details
5) NMR-Spectra
6) Cartesian Coordinates

1) Additional Figures and Tables

Table S1: Selected NMR spectroscopic data for the compounds **2**-**7** in C_6D_6.

	^{1}H NMR [ppm]			^{13}C{^{1}H} NMR [ppm]	^{19}F{^{1}H} NMR [ppm]
	aryl-CH_{3ortho} (s)	aryl-CH_{3para} (s)	NC*H*C*H*N (s)	NHC-*C*	Ni-*F*
**2**	1.96	2.35	5.86	175.3	-360.8
**3**	1.96	2.38	5.88	176.4	-361.9
**4**	1.96	2.36	5.86	175.3	-357.9
**5**	2.00	2.40	5.91	177.2	-357.2
**6**	1.90 (br)<br>2.12 (br)	2.42	5.90	180.3	-344.2
**7**	1.48 (br)<br>2.52 (br)	2.42	5.90	183.2	-333.1

Table S2: Crystallographic data of compounds **1**, **3**, **4**, **5**, **6**, **8**, **9**, **11**, **13**, **14**, **[Ni^I(6-Mes)$_2$][Br]**[S1a] and **[Ni^I(P^{i}Pr$_3$)$_2$(C$_6$F$_5$)]**.[S1b]

	**d Ni–C1/C2**	**d Ni–C3$_{(ArF)}$**	**d Ni–F**	**∢ C1-Ni-C2**	**∢ NHC(C1):NHC(C2)**
**[Ni(Mes$_2$Im)$_2$] 1**	1.827(6)<br>1.830(6)	–	–	176.4	53.0
**3**	1.923(3)<br>1.922(3)	C3: 1.882(7)<br>C3': 1.944(5)	1.844(2)	175.6(1)	37.07(2)
**4**	1.923(3)<br>1.920(3)	1.883(3)	1.859(2)	174.3(1)	36.01(2)
**5**	1.921(2)<br>1.924(2)	1.896(3)	1.856(2)	176.4(1)	33.81(1)
**6**	1.912(3)<br>1.912(3)	1.854(5)	1.874(2)	176.7(1)	31.65(2)
**8**	1.894(3)<br>1.894(3)	–	–	174.5(1)	57.99(1)
**9**	1.903(3)<br>1.902(3)	–	F1: 1.845(2)<br>F2: 1.823(2)	178.5(1)	53.34(1)
**11**	1.923(2)<br>1.923(2)	1.984(3)	–	159.8(8)	82.37(1)
**13**	1.930(2)<br>1.930(2)	1.987(3)	–	157.3(8)	82.11(1)
**14**	1.918(1)<br>1.917(1)	C3: 1.869(1)<br>C3': 2.046(1)	–	159.5(5)	82.46(8)
**[Ni^I(6-Mes)$_2$][Br]**	1.939(3)<br>1.941(3)	–	–	179.3(1)	57.99(1)
**[Ni^I(P^{i}Pr$_3$)$_2$(C$_6$F$_5$)]**	P1: 2.243(5)<br>P2: 2.233(5)	1.973(2)	–	P1-Ni-P2<br>145.2(2)	–

All complexes *trans*-[Ni(Mes$_2$Im)$_2$F(Ar^F)] (Ar^F = C_6F_5 **3**, 2,3,5,6-C_5F_4N **4**, 2,3,5,6-C_6F_4H **5**, 2,3,5-$C_6F_3H_2$ **6**) adopt a square planar structure with a *trans*-arrangement of the NHC ligands. A decreasing degree of fluorination of the fluoroaryl ligand leads to a slight lengthening of the Ni–F bond lengths (Ni–F: **3**: 1.844(2) Å, **4**: 1.859(2) Å, **5**: 1.856(2) Å, **6**: 1.874(2) Å), while the distances from the nickel center to the fluoroaryl ligand gradually become shorter (Ni–C3: **3**: 1.944(5) Å, **4**: 1.883(3) Å, **5**: 1.896(3) Å, **6**: 1.854(5) Å). The nickel-NHC carbon distances of **3**, **4**, **5** and **6** lie within 0.01 Å (1.912(3)-1.923(3) Å) and the C1-Ni-C2 angles between the NHC ligands also vary only slightly (**3**: 175.6(1) °, **4**: 174.3(1) °, **5**: 176.4(1) °, **6**: 176.7(1) °). The NHC ligands are bent away from the aromatic ring towards the smaller fluoride ligand. The least square planes through both NHC ligands (defined by the C_3N_2 core of the NHC) intersect each other with angles between 31.65(2)° and 37.07(2)° (**3**: 37.07(2) °, **4**: 36.01(2) °, **5**: 33.81(1) °, **6**: 31.65(2) °). One NHC in each complex is almost perpendicular to the square plane of the nickel complex (**3**: NHC(C2): 82.27 °, **4**: NHC(C1): 85.94(7) °, **5**: NHC(C2) 83.00(1) °), whereas the other NHC is twisted between 64.47(7)° and 66.70(1) ° relative to the least squares plane.

Table S3: Experimental and DFT calculated *g* tensors for species **8**.

Compound		*g* tensor components		
		g_{xx}	g_{yy}	g_{zz}
**8a**	Exp. (solid state)	2.02	2.47	2.62
**8b**	Exp. (solid state)	1.98	2.06	2.13
**[Ni(Mes$_2$Im)$_2$]$^+$ (gas phase)**	DFT	2.01	2.65	2.98
**[Ni(Mes$_2$Im)$_2$]$^+$ (xrd)** [a]	DFT	2.01	2.65	3.01
**8^1_{xrd}**[b]	DFT	2.01	2.65	3.01
**8^2_{xrd}**[b]	DFT	2.02	2.65	3.02
**8^1_{DFT}**[c]	DFT	2.03	2.50	2.59
**8^2_{DFT}**[d]	DFT	2.01	2.66	2.98
**8^3_{DFT}**[e]	DFT	2.09	2.32	2.46

[a] *g* tensor calculated for the cation XRD structure with relaxed C–H bond lengths.
[b] *g* tensor calculated for the cation XRD structure with relaxed C–H bond lenghts and under consideration of the BF_4^- counter ion in two different positions.
[c] DFT-optimized structure with Ni–F^{BF4-} contacts.
[d] Geometry was relaxed starting from the XRD geometry 8^1_{xrd}; note that the optimized geometry features one artificial imaginary normal mode of I 37 cm^{-1} in C_2 symmetry – lifting the computational symmetry constraint towards C_1 eliminates this artefact. However it increases the relative energy.
[e] DFT-optimized structure with Ni–H^{Mes} contacts.

Table S4: Comparison of experimental and calculated *g* tensors for compounds **11**, **13** and **14**.

Compound	DFT/Exp[a]	*g* tensor components[b]		
		g_{xx}	g_{yy}	g_{zz}
**11**	Exp. (isol.)	2.04	2.16	2.31
	DFT	2.06	2.17	2.29
**13**	Exp. (isol.)	2.03	2.16	2.30
	DFT	2.06	2.17	2.29
**14**	Exp. (isol.)	2.03	2.17	2.24
	DFT	2.05	2.17	2.27

[a] The experimental g-tensor components are organized in ascending order from g_{xx} to g_{zz}
[b] EPR parameters have been calculated using DFT. The calculated values are rounded off to match the number of digits of the experimental values.

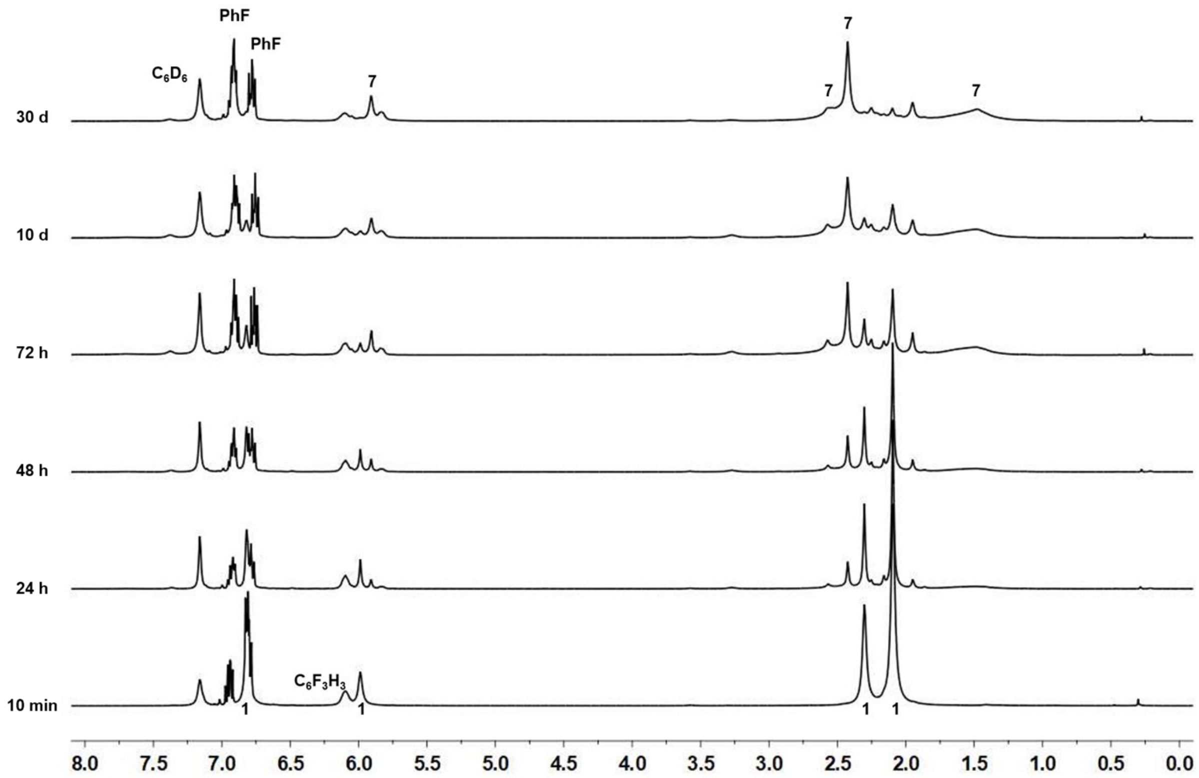

Figure S1: ^{1}H NMR (400.4 MHz) spectra of the reaction of [Ni(Mes$_2$Im)$_2$] (**1**) and 3 equivalents of 1,3,5-$C_6F_3H_3$ in C_6D_6 at different reaction times at room temperature, calibrated with a C_6H_5F in a capillary.

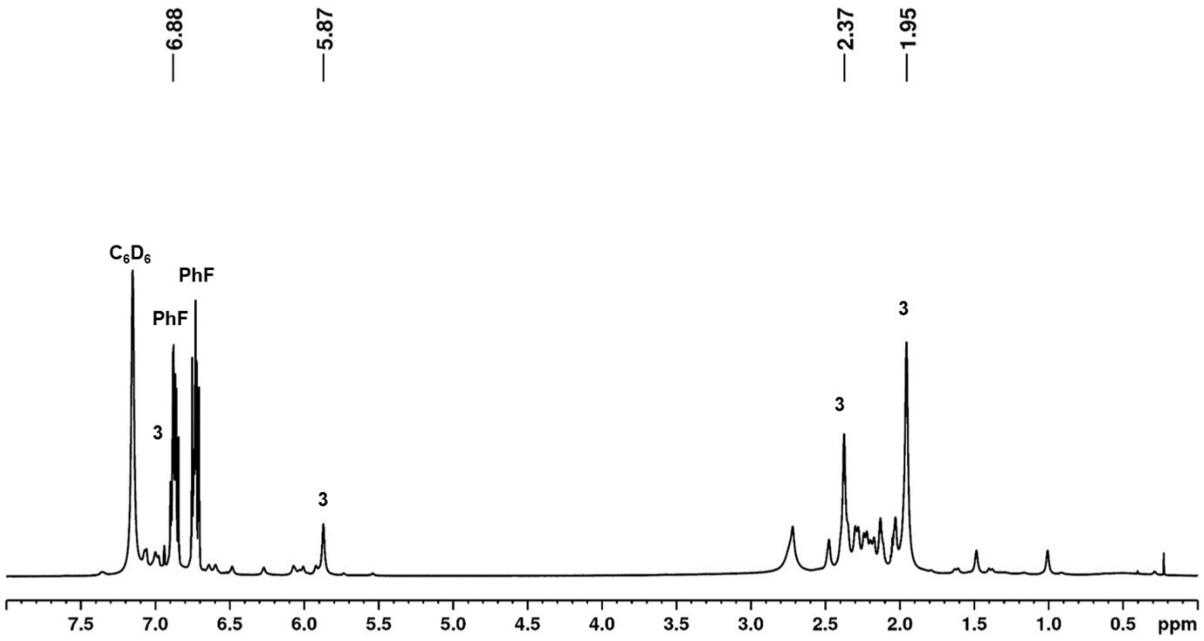

Figure S2: ^{1}H NMR (400.4 MHz) spectrum of the reaction of [Ni(Mes$_2$Im)$_2$] (**1**) with C_6F_6 at room temperature after 5 min, calibrated with a C_6H_5F in a capillary.

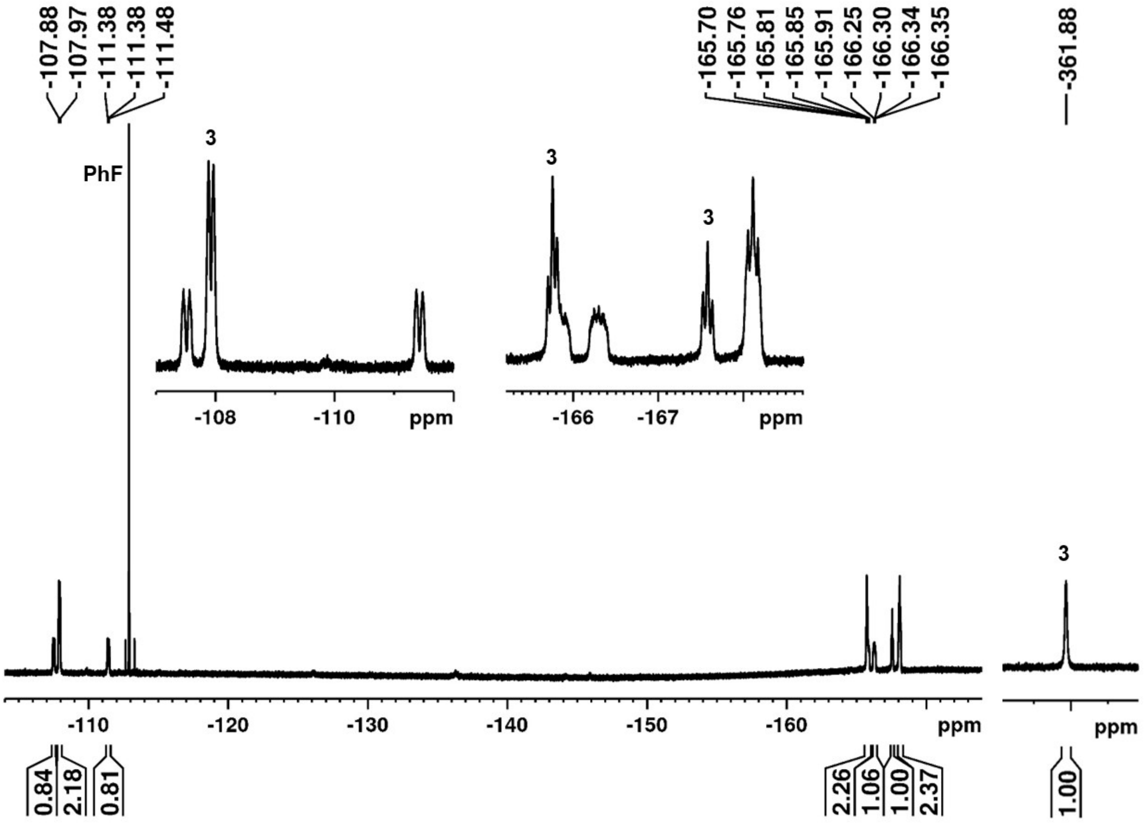

Figure S3: $^{19}F\{^1H\}$ NMR (370.6 MHz) spectrum of the reaction of [Ni(Mes$_2$Im)$_2$] (**1**) with C_6F_6 at room temperature after 5 min, calibrated with a C_6H_5F in a capillary.

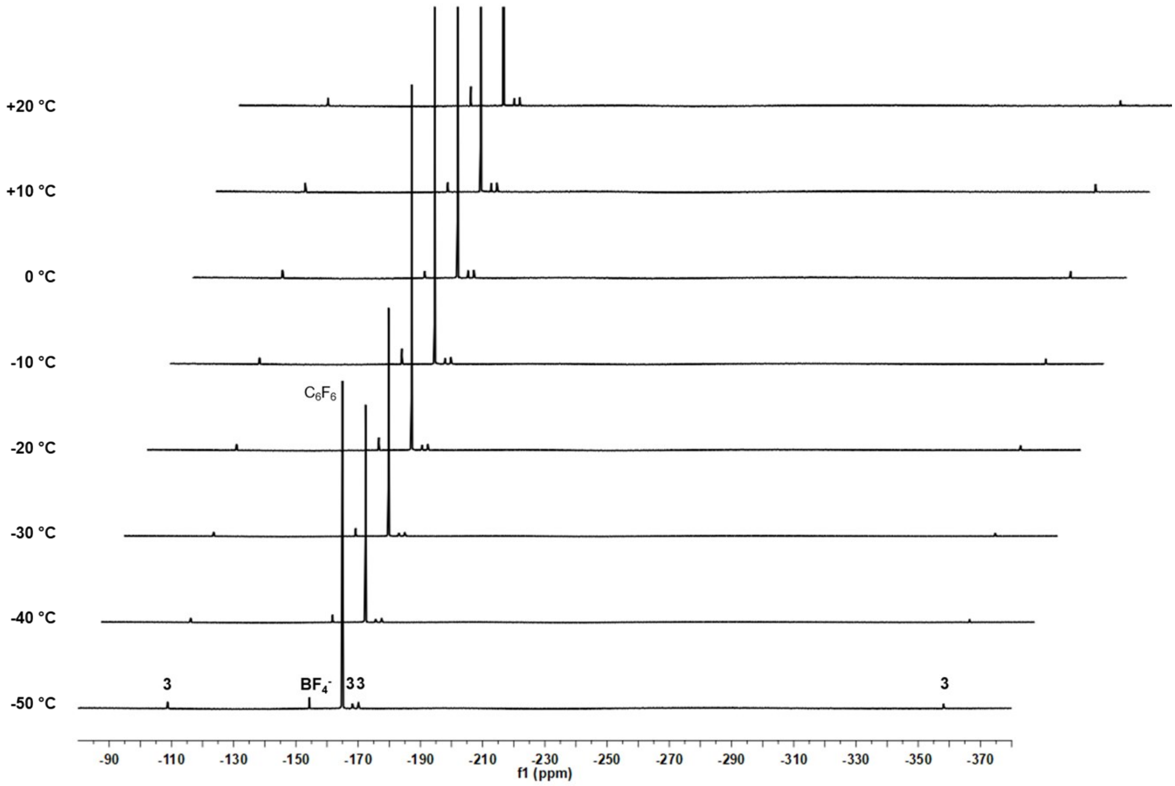

Figure S4: $^{19}F\{^1H\}$-VT NMR (188.1 MHz) spectra of the reaction mixture of [Ni(Mes$_2$Im)$_2$] (**1**) with C_6F_6 in thf-d_8 at various temperatures (-50 °C up to 20 °C).

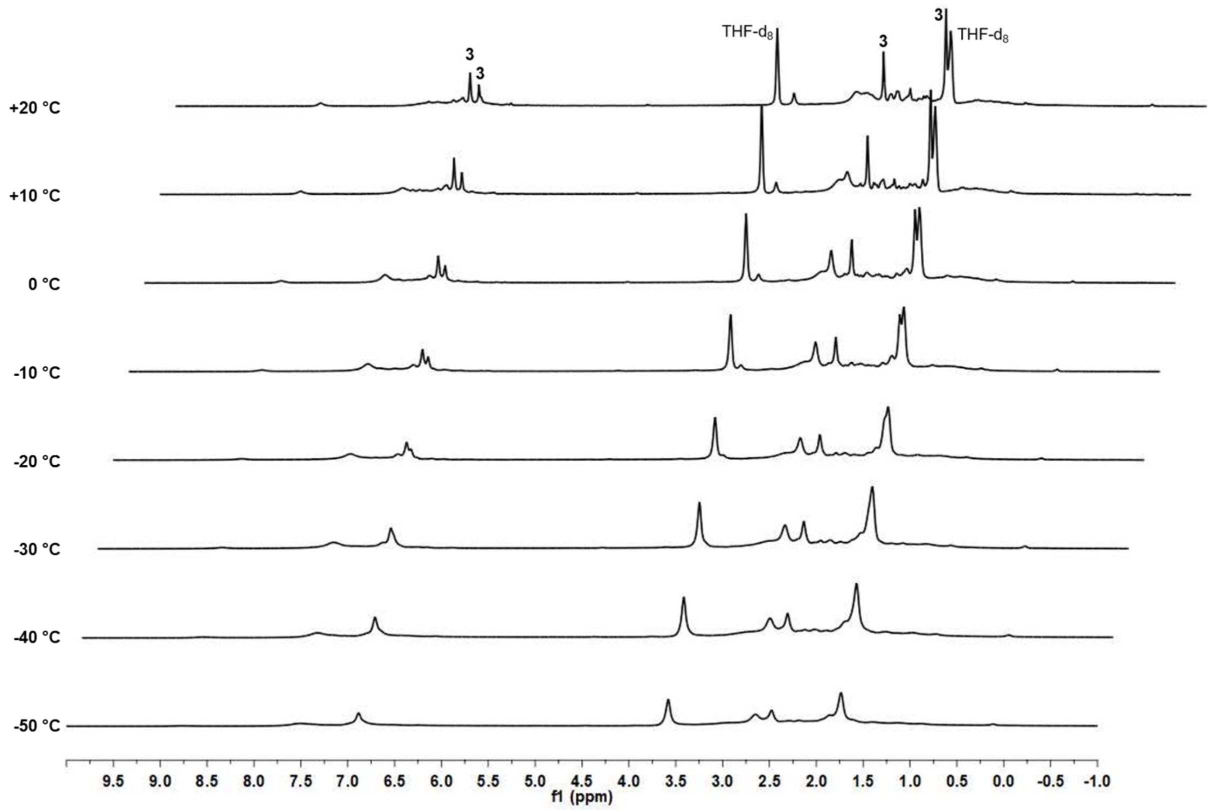

Figure S5: ^{1}H-VT NMR (200.1 MHz) spectra of the reaction mixture of [Ni(Mes$_2$Im)$_2$] (**1**) with C_6F_6 in thf-d$_8$ at various temperatures (-50 °C up to 20 °C).

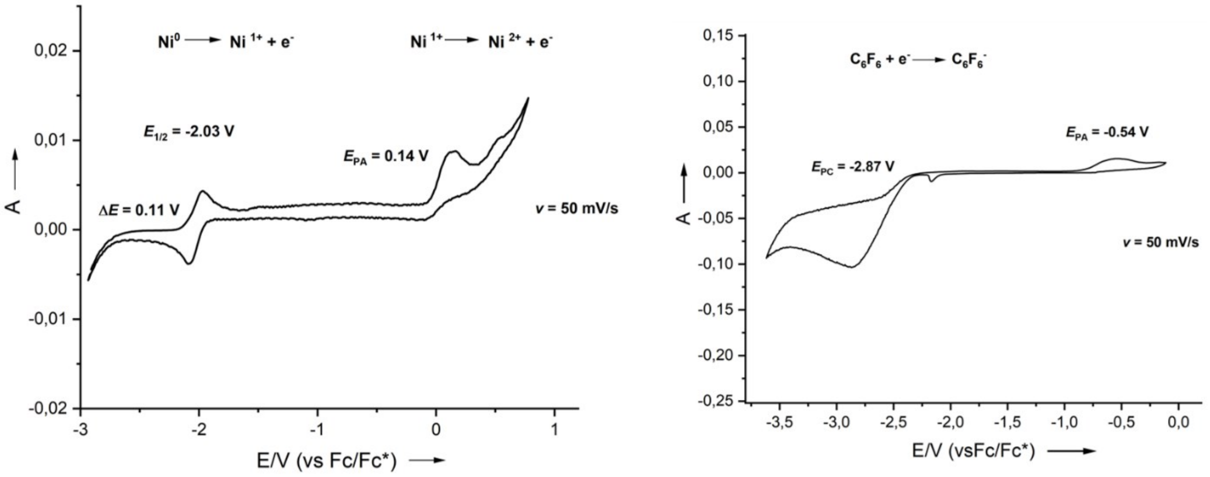

$$[Ni(Mes_2Im)_2] \xrightarrow{E_{1/2} = -2.03\ V} [Ni(Mes_2Im)_2]^{+\bullet} + e^-$$

$$[Ni(Mes_2Im)_2]^{+\bullet} \xrightarrow{E_{PA} = 0.14\ V} [Ni(Mes_2Im)_2]^{2+} + e^-$$

$$C_6F_6 + e^- \xrightarrow{E_{PC} = -2.87\ V} C_6F_6^{\bullet -}$$

Figure S6: Cyclic voltammograms of [Ni(Mes$_2$Im)$_2$] (**1**) (left) and C_6F_6 (right) in thf using 0.1 M $TBAPF_6$ as supporting electrolyte. Potentials are referenced to the ferrocene/ferrocenium couple.

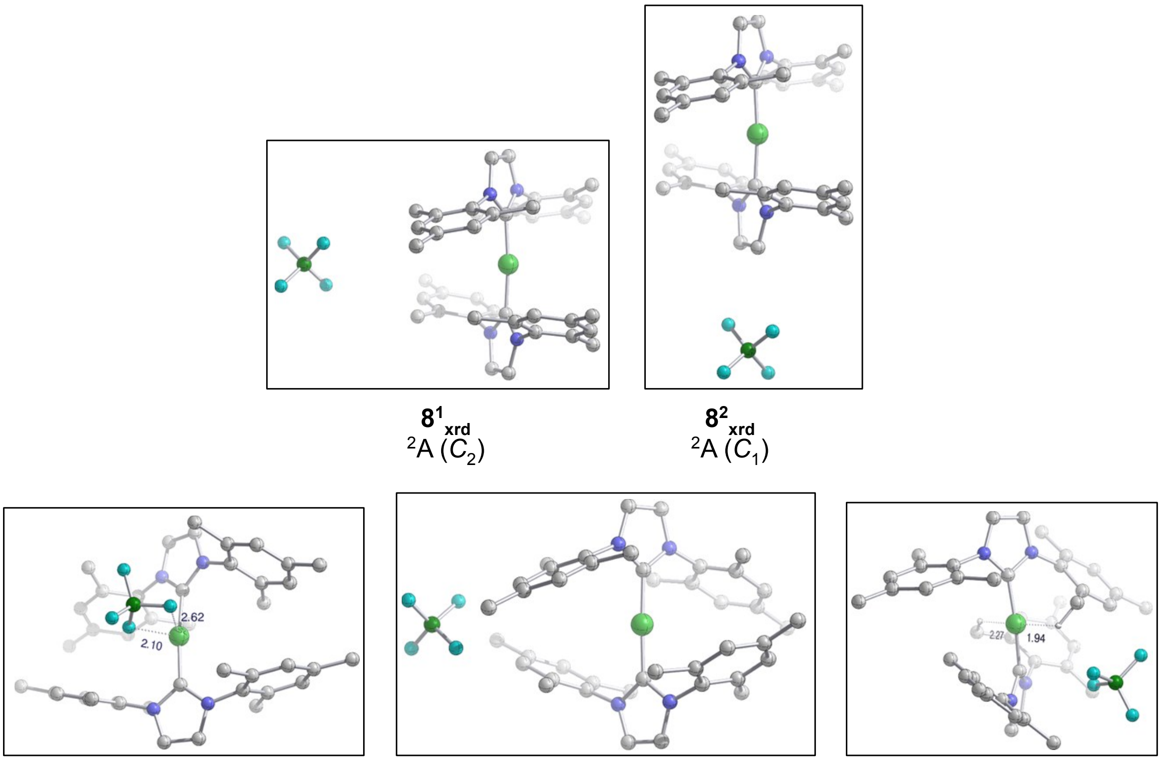

8^1_{xrd} ^{2}A (C_2) **8^2_{xrd}** ^{2}A (C_1)

8^1_{DFT} ^{2}A (C_1) **8^2_{DFT}** ^{2}A (C_2) **8^3_{DFT}** ^{2}A (C_1)

Figure S7: Geometries of conformers of $[Ni(Mes_2Im)_2][BF_4]$ **8** including the BF_4^- counter ion. Two structures derived from XRD (X-Ray diffraction) coordinates (**8^1**$_{xrd}$ ^{2}A(C_2) and **8^2**$_{xrd}$ ^{2}A(C_1)), DFT-relaxed geometry **8^2**$_{DFT}$ starting from the XRD-geometry, and two structures with Ni-H (**8^3**$_{DFT}$) and Ni-F (**8^1**$_{DFT}$) contacts.

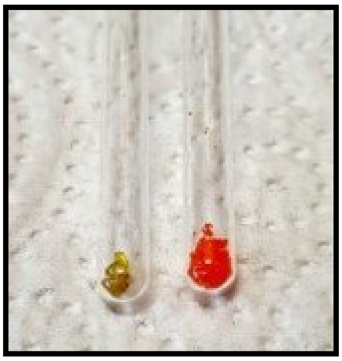

Figure S8: Isolated crystals of **3** (left) and **11** (right).

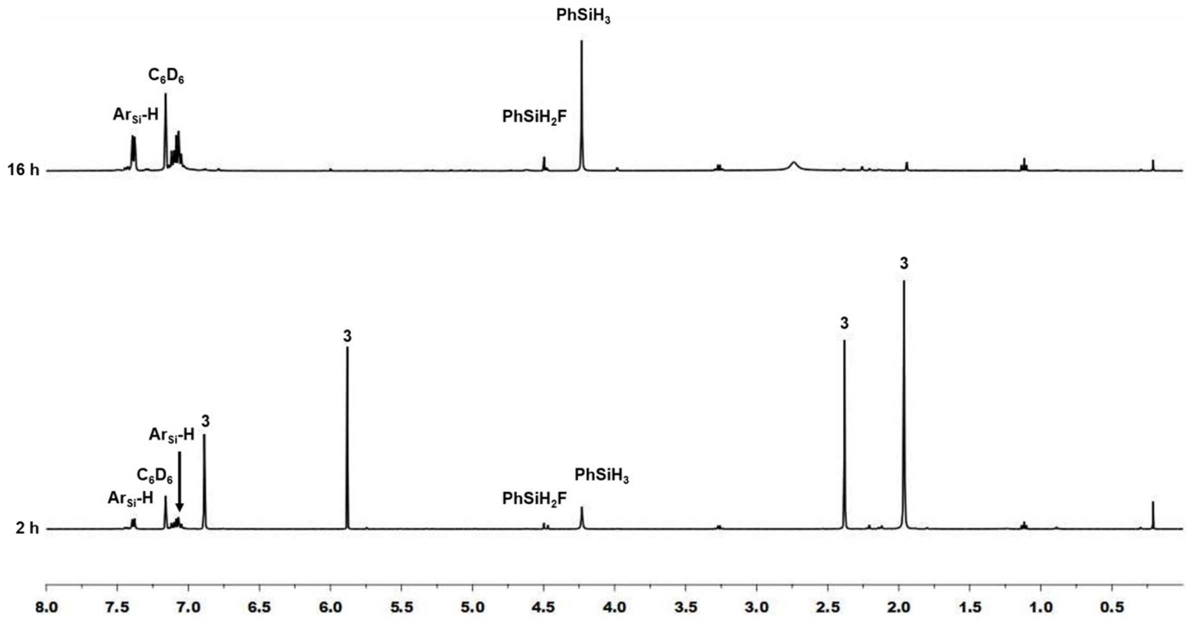

Figure S9: ^{1}H NMR (400.4 MHz) spectra of the reaction of $[Ni(Mes_2Im)_2F(C_6F_6)]$ (**3**) and $PhSiH_3$ in C_6D_6 at room temperature at different times.

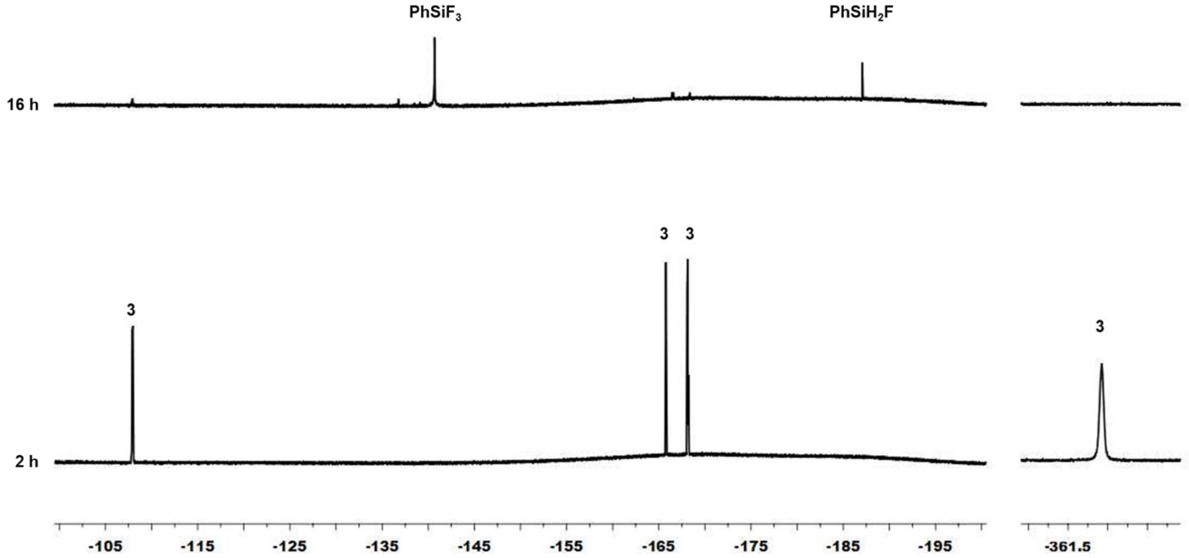

Figure S10: $^{19}F\{^{1}H\}$ NMR (370.6 MHz) spectra of the reaction of $[Ni(Mes_2Im)_2F(C_6F_6)]$ (**3**) and $PhSiH_3$ in C_6D_6 at room temperature at different times.

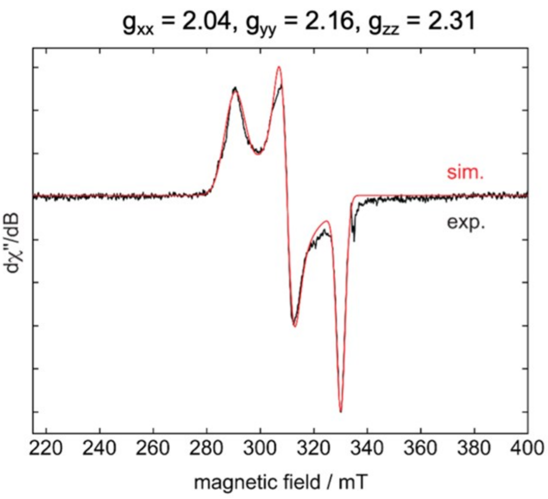

Figure S11: EPR spectrum of isolated [Ni^{I}(Mes$_2$Im)$_2$(C$_6$F$_5$)] (**11**) in thf at -203°C.

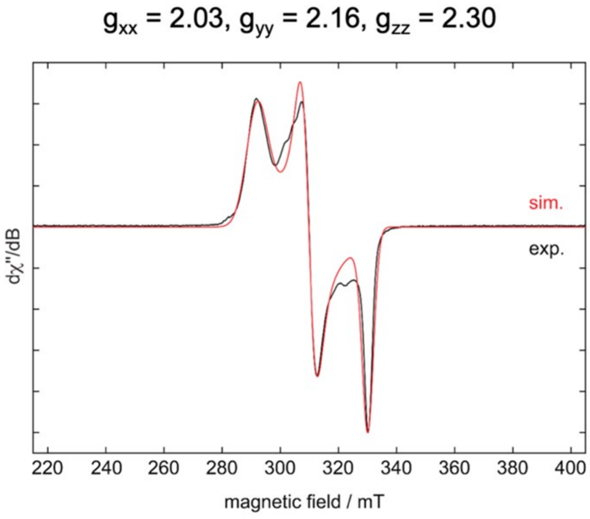

Figure S12: EPR spectrum of [Ni^{I}(Mes$_2$Im)$_2$(2,3,5,6-C$_6$F$_4$H)] (**13**) in thf at -203°C.

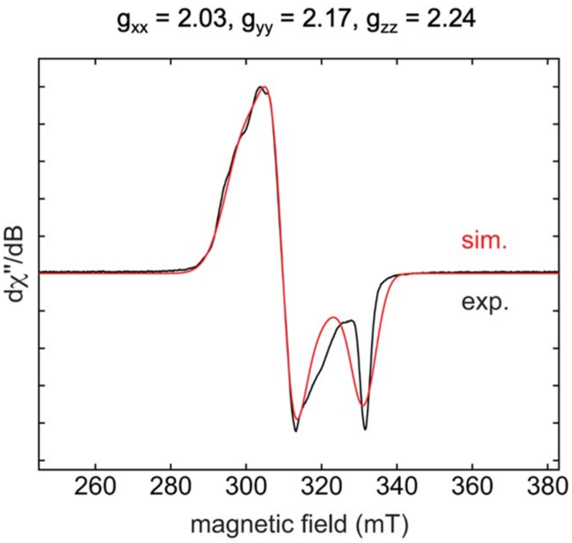

Figure S13: EPR spectrum of [Ni^{I}(Mes$_2$Im)$_2$(2,3,5-C$_6$F$_3$H$_2$)] (**14**) in thf at -203°C.

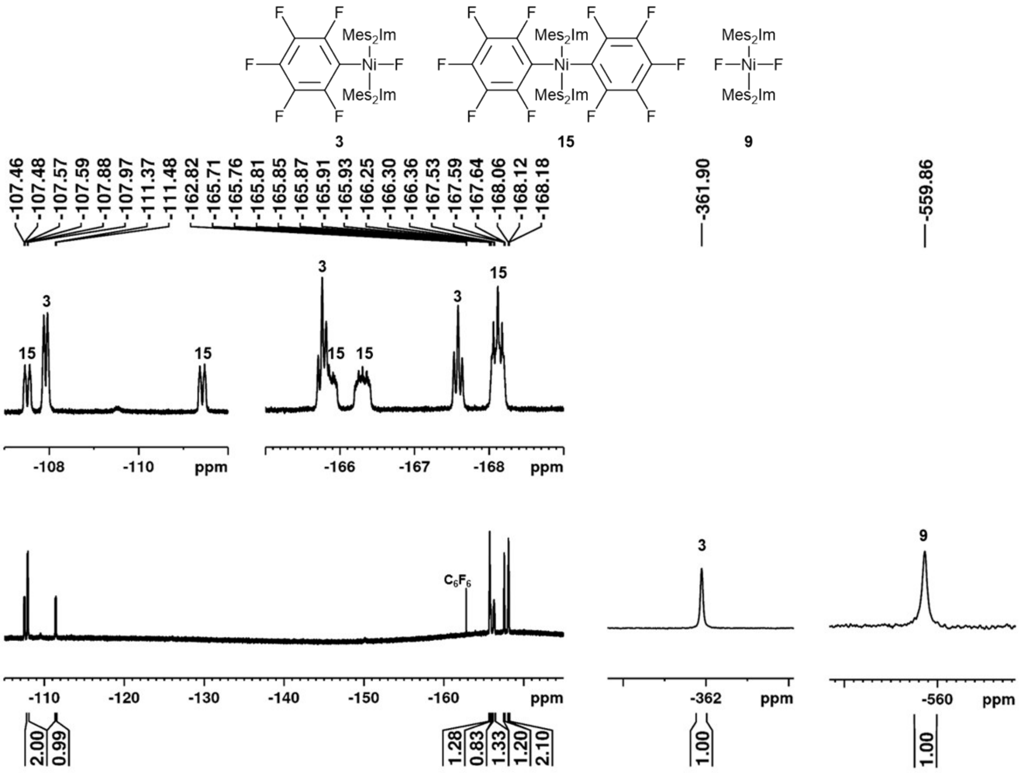

Figure S14: $^{19}F\{^1H\}$ NMR spectra of the reaction of **1** with C_6F_6 in C_6D_6 at room temperature after 48 h.

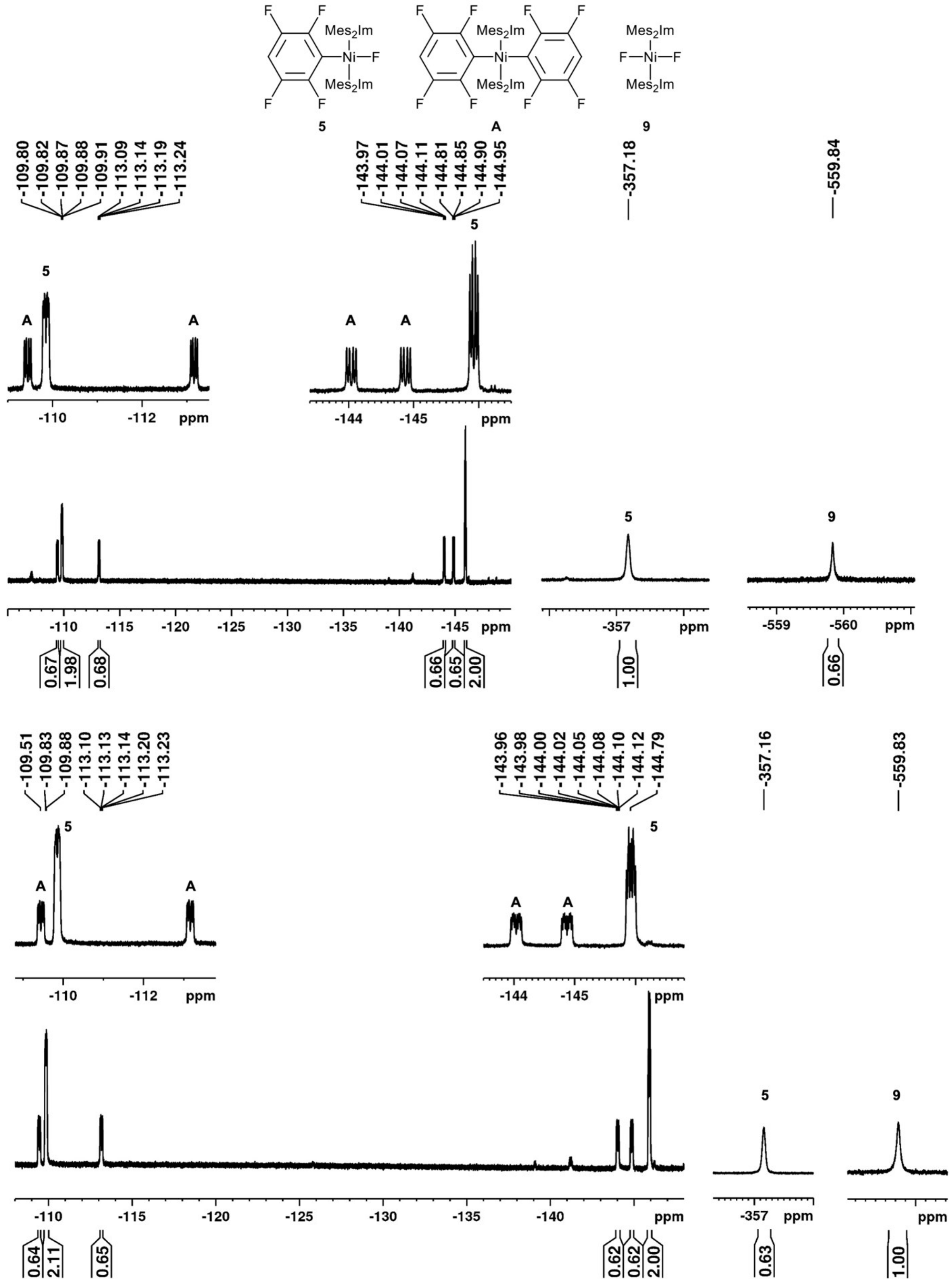

Figure S15a: $^{19}F\{^1H\}$ NMR (top) and ^{19}F NMR (bottom) spectrum (370.6 MHz) of the reaction mixture of $[Ni(Mes_2Im)_2]$ (**1**) with C_6F_5H in C_6D_6 at room temperature after 48 hours.

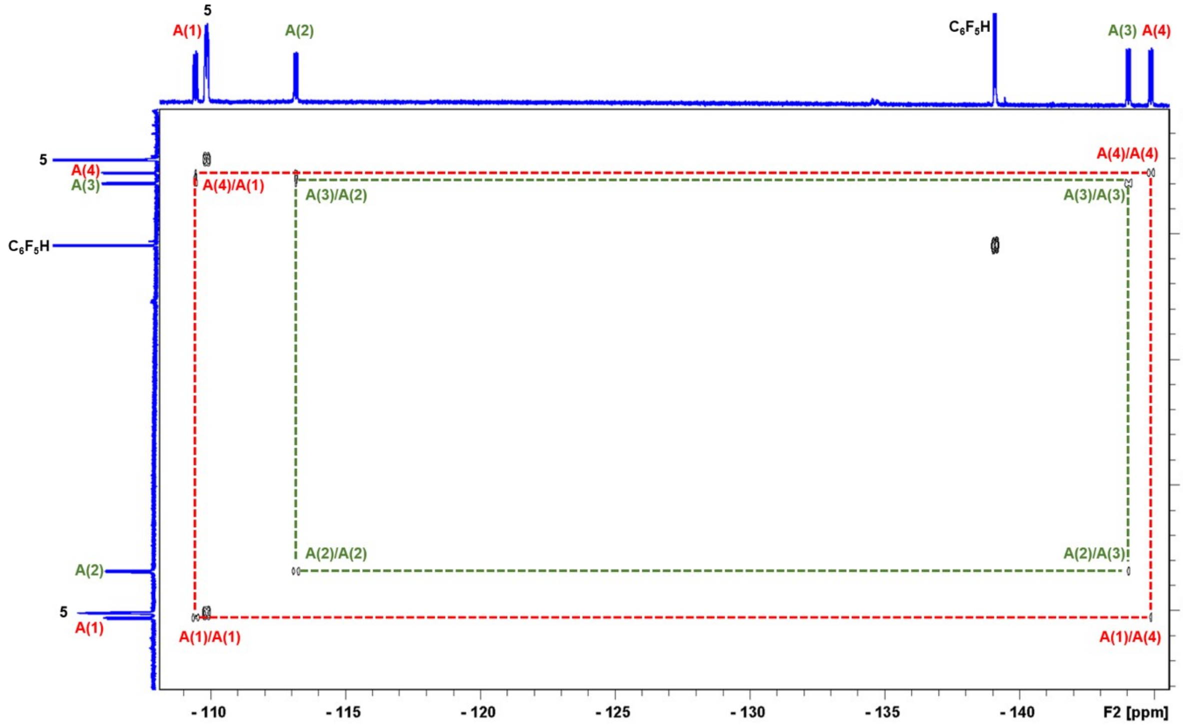

Figure S15b: Section of the $^{19}F\{^{1}H\}/^{19}F\{^{1}H\}$ COSY NMR spectrum of the reaction mixture of [Ni(Mes$_2$Im)$_2$] (**1**) with C_6F_5H in C_6D_6 at room temperature after 48 hours, which shows the crosspeaks for the fluoroaryl fluorine atoms.

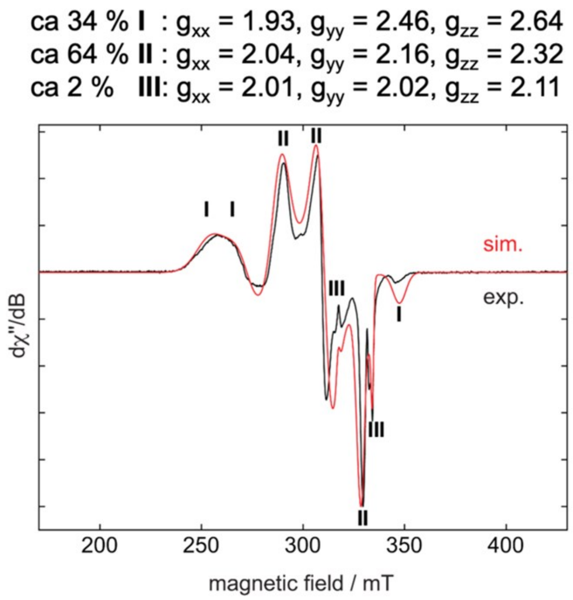

Figure S16: EPR spectrum (70 K) of a mixture of **1** and pentafluorobenzene after 10 min at -78 °C in thf.

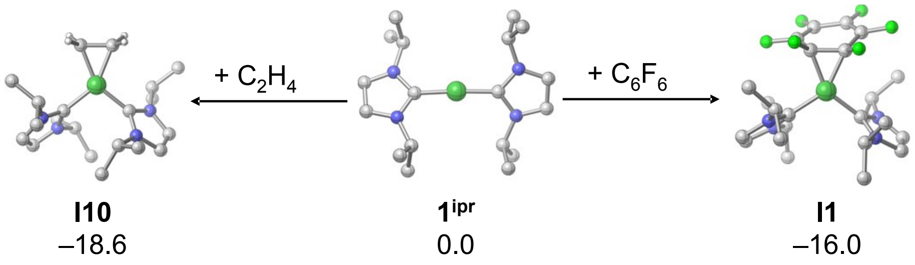

Figure S17: Computed η^2 coordination of C_2H_4 and C_6F_6 to **1**ipr (ΔG^{298} in kcal mol^{-1}; H atoms omitted for clarity).

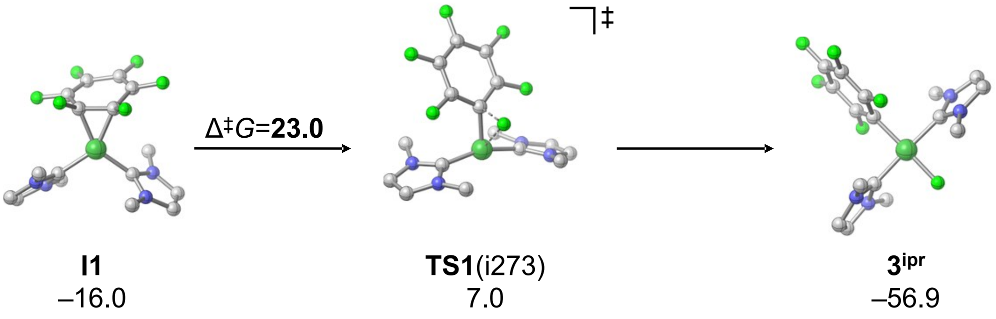

Figure S18: Oxidative addition pathway leading to the *trans* product **3**ipr (ΔG^{298} in kcal mol^{-1}); only secondary carbon atoms of ^{i}Pr groups are shown.

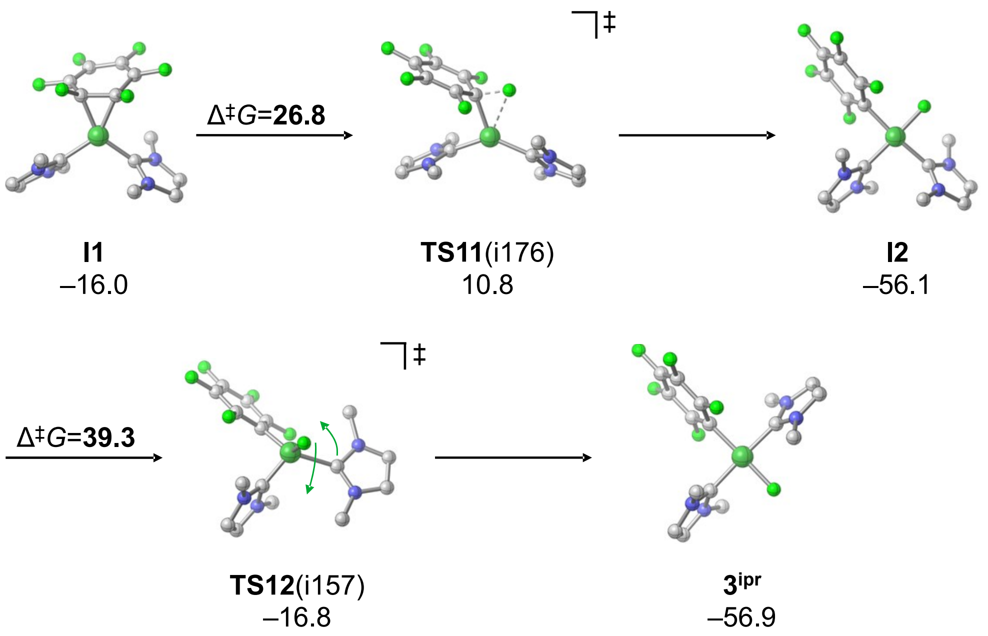

Figure S19: Oxidative addition pathway leading to the *cis* product *cis*-[Ni(^{i}Pr$_2$Im)$_2$F(C_6F_5)] **I2** with subsequent *cis/trans*-isomerization (ΔG^{298} in kcal mol^{-1}); only secondary carbon atoms of ^{i}Pr groups are shown.

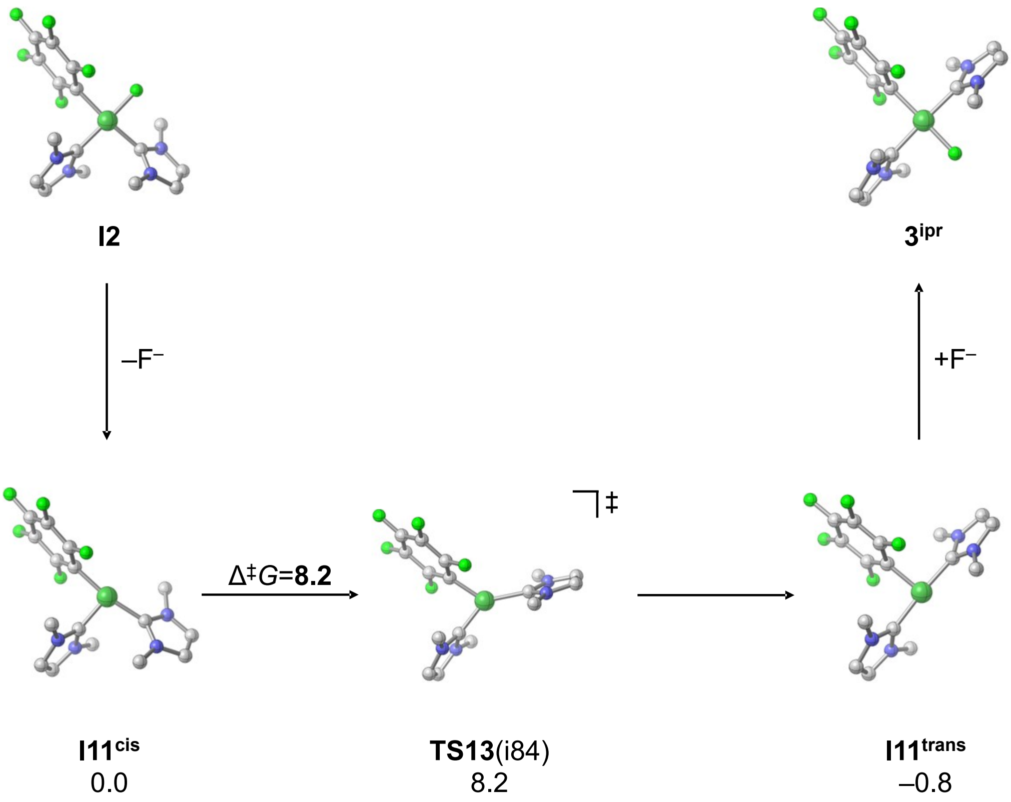

Figure S20: Alternative *cis*/*trans*-isomerization pathway of *cis*-[Ni(^{i}Pr$_2$Im)$_2$(C_6F_5)] (**I11^{cis}**) via [Ni(^{i}Pr$_2$Im)$_2$(C_6F_5)]$^+$ (ΔG^{298} in kcal mol^{-1}); only secondary carbon atoms of ^{i}Pr groups are shown.

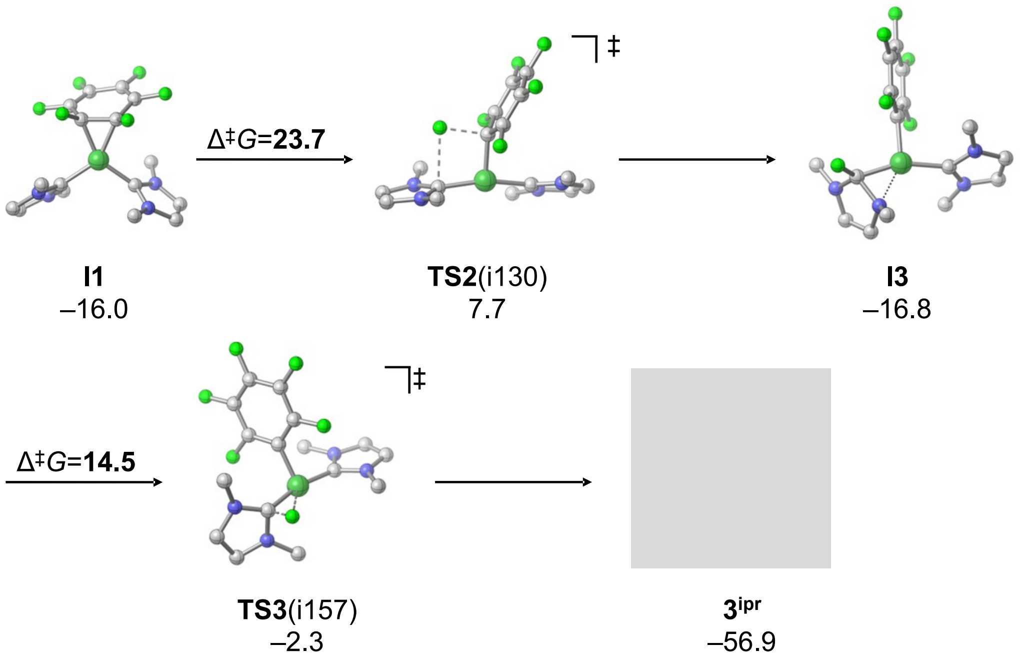

Figure S21: NHC-assisted pathway leading to **3**^{ipr} (ΔG^{298} in kcal mol^{-1}); only secondary carbon atoms of ^{i}Pr groups are shown.

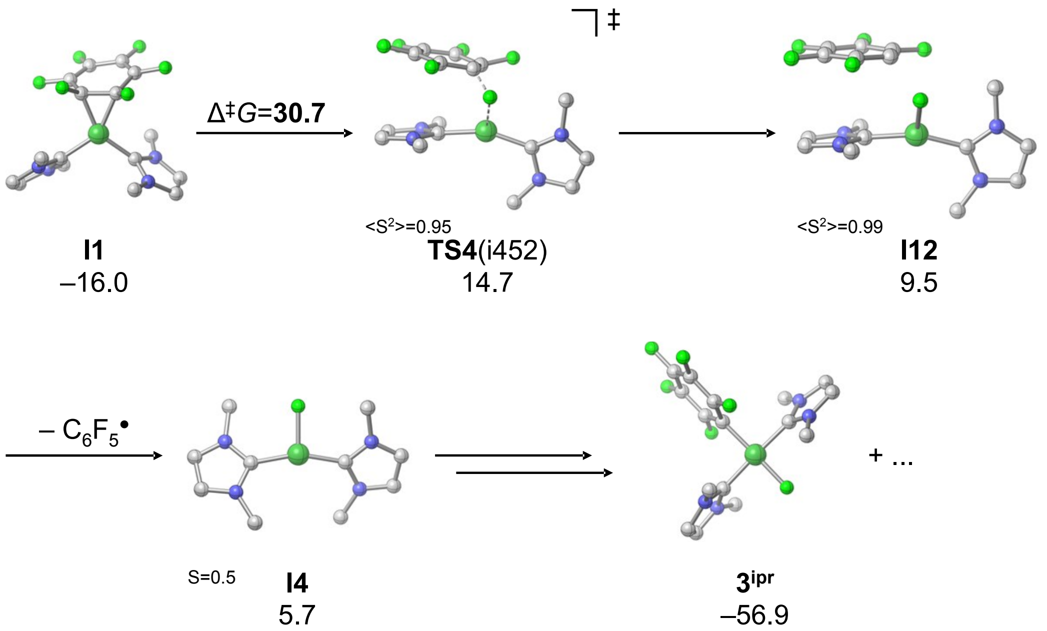

Figure S22: Calculations on the homolytic C–F bond cleavage of C_6F_6 by [Ni(^{i}Pr$_2$Im)$_2$] (**1**^{iPr}) (ΔG^{298} in kcal mol^{-1}); only secondary carbon atoms of ^{i}Pr groups are shown.

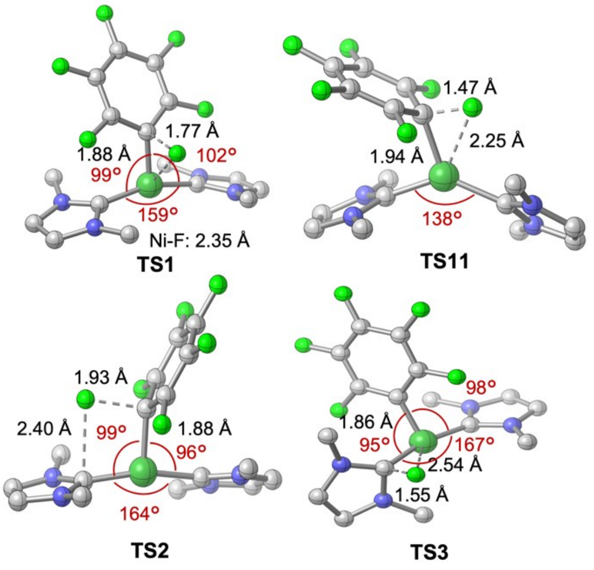

Figure S23: DFT optimized structures of the transition states **TS1**, **TS11**, **TS2** and **TS3** and important bond lengths and bond angles; only secondary carbon atoms of ^{i}Pr groups are shown.

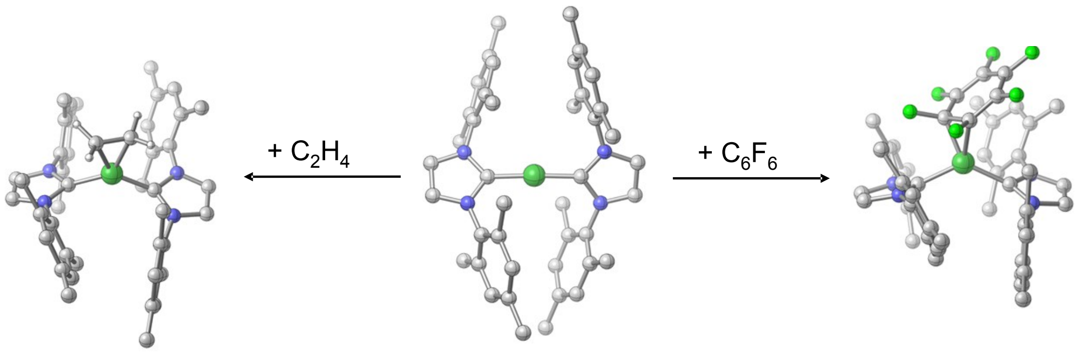

**I13**	**1**	**I5**
–4.1	0.0	11.6

Figure S24: Computed η^2 coordination of C_2H_4 and C_6F_6 to **1** (ΔG^{298} in kcal mol^{-1}; H atoms omitted for clarity).

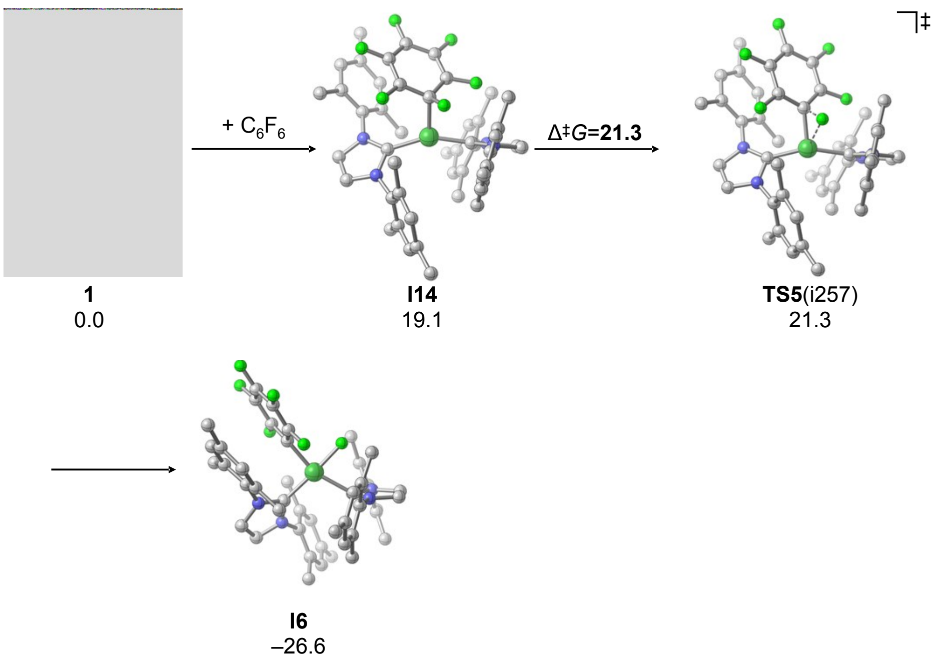

Figure S25: Calculations on the oxidative addition pathway for the reaction of C_6F_6 with [Ni(Mes$_2$Im)$_2$] (ΔG^{298} in kcal mol^{-1}; H atoms omitted for clarity).

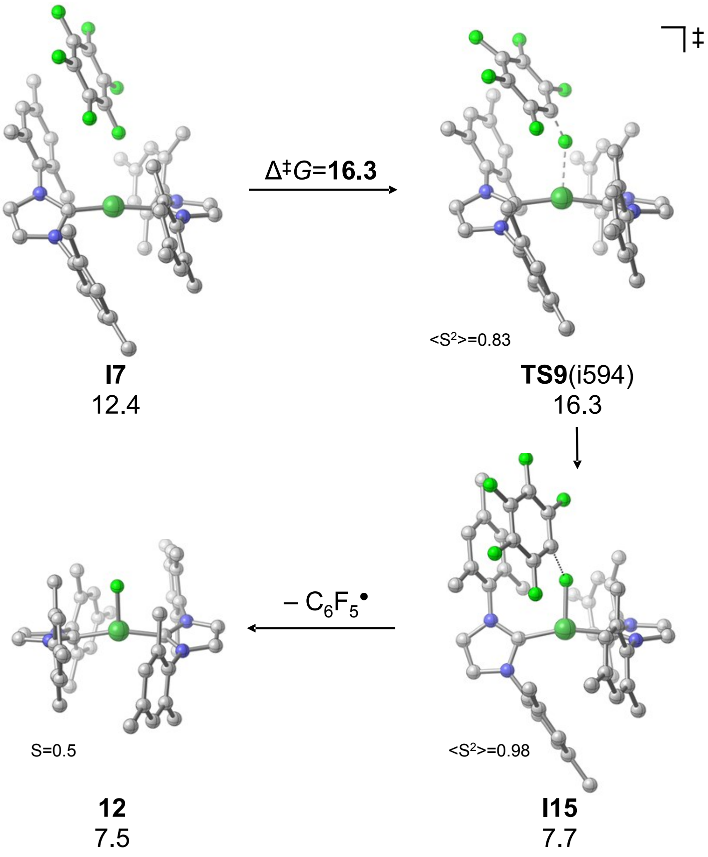

Figure S26: DFT calculations on the reaction of [Ni(Mes$_2$Im)$_2$] (**1**) with C_6F_6: Fluoride abstraction from C_6F_6 (ΔG^{298} in kcal mol^{-1}; H atoms omitted for clarity). The intermediate **I7** is stabilized by van der Waals π-stacking interactions between C_6F_6 and one of the NHC mesityl substituents, which are aligned almost parallel to one another with $C_{mesityl}$-$C_{fluorobenzene}$ contacts in the range between 3.35 Å and 3.73 Å.

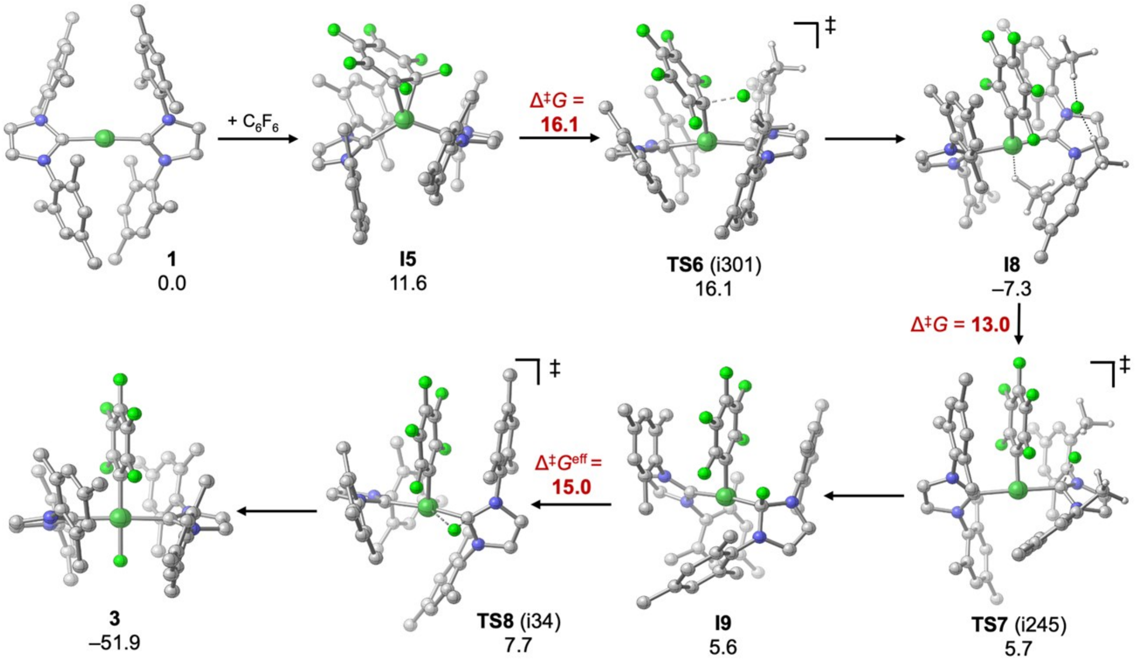

Figure S27: DFT calculations on the reaction of [Ni(Mes$_2$Im)$_2$] (**1**) with C_6F_6: NHC-assisted mechanism leading to *trans*-[Ni(Mes$_2$Im)$_2$(F)(C_6F_5)] (**3**) (ΔG^{298} in kcal mol^{-1}). Hydrogen atoms have been omitted for clarity.

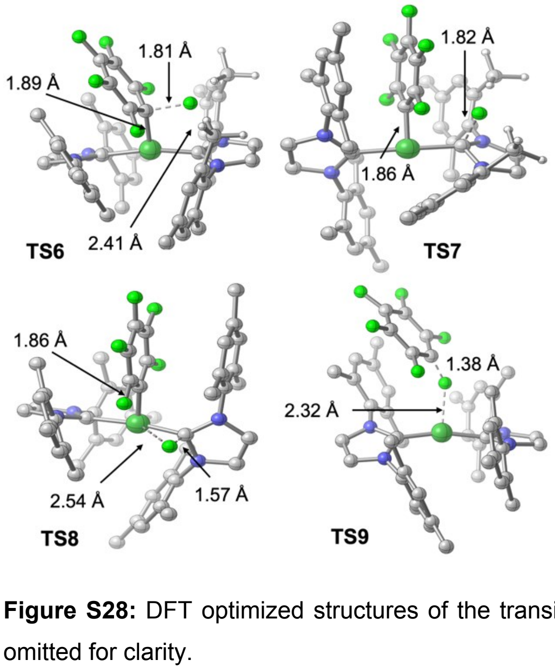

Figure S28: DFT optimized structures of the transition states **TS6** – **TS9** and important bond lengths; H atoms omitted for clarity.

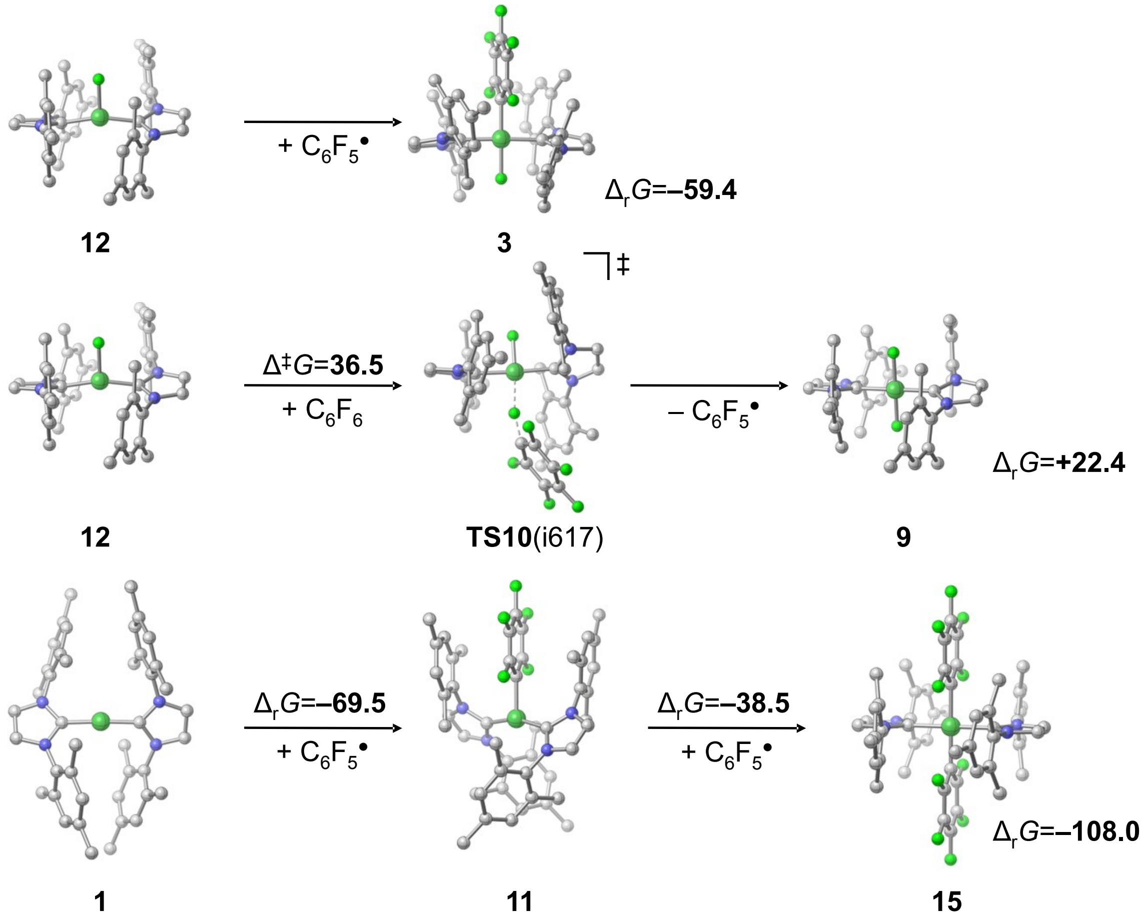

Figure S29: Free enthalpies for possible radical reaction steps, indicating that the reactions of $C_6F_5^\bullet$ with **12**, **1**, and **11** are highly exergonic, while the reaction of **12** with C_6F_6 is endergonic ($\Delta_r G^{298}$ in kcal mol^{-1}; H atoms omitted for clarity).

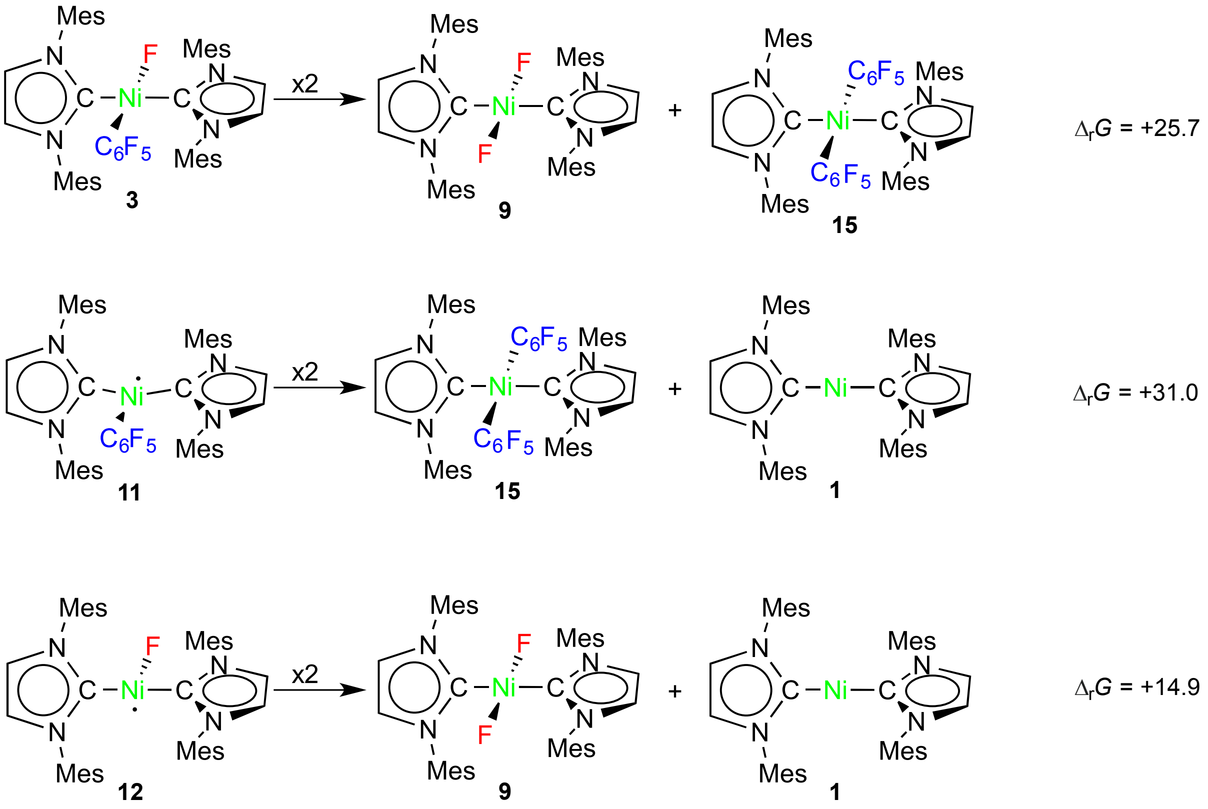

Figure S30: Free enthalpies for ligand redistribution reactions, indicating that the formation of **9** and **15** via these pathways is endergonic and thus precluded.

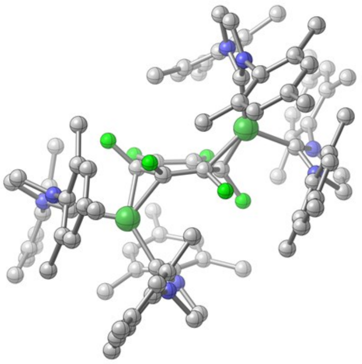

I16
35.3

Figure S31: Calculated molecular structure of the dinuclear complex [Ni(Mes$_2$Im)$_2$]$_2$(μ-η^2:η^2-C_6F_6)] **I16** (ΔG^{298} in kcal mol^{-1}; H atoms omitted for clarity), analogous to the dinuclear nickel C_6F_5H complex reported previously by Johnson *et al.*[S1b] The highly endergonic formation of **I16** prevents further dinuclear reaction pathways for this system.

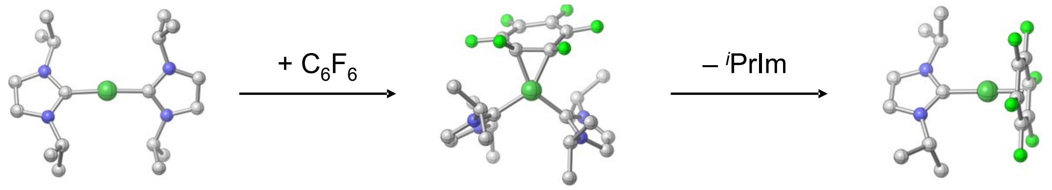

1^ipr^ 0.0 | **I1** –16.0 | **I17** 11.7

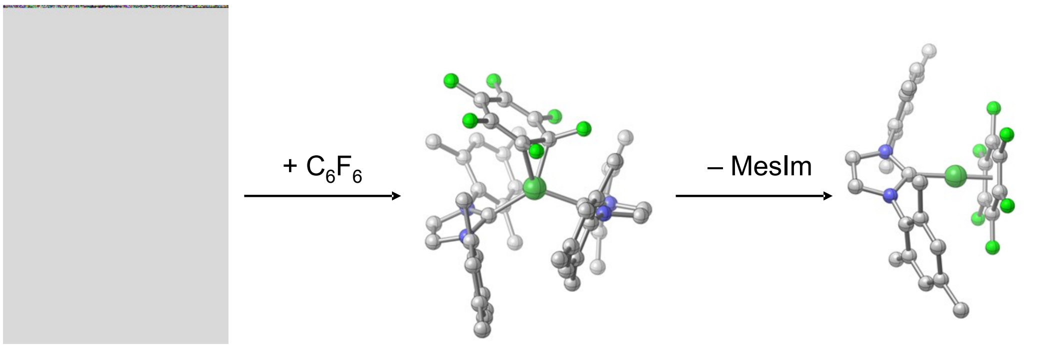

1 0.0 | **I5** 11.6 | **I18** 10.7

Figure S32: DFT calculations on the reaction of [Ni(^{i}Pr$_2$Im)$_2$] (**1^{ipr}**) and [Ni(Mes$_2$Im)$_2$] (**1**) with C_6F_6: NHC dissociation (ΔG^{298} in kcal mol^{-1}; H atoms omitted for clarity).

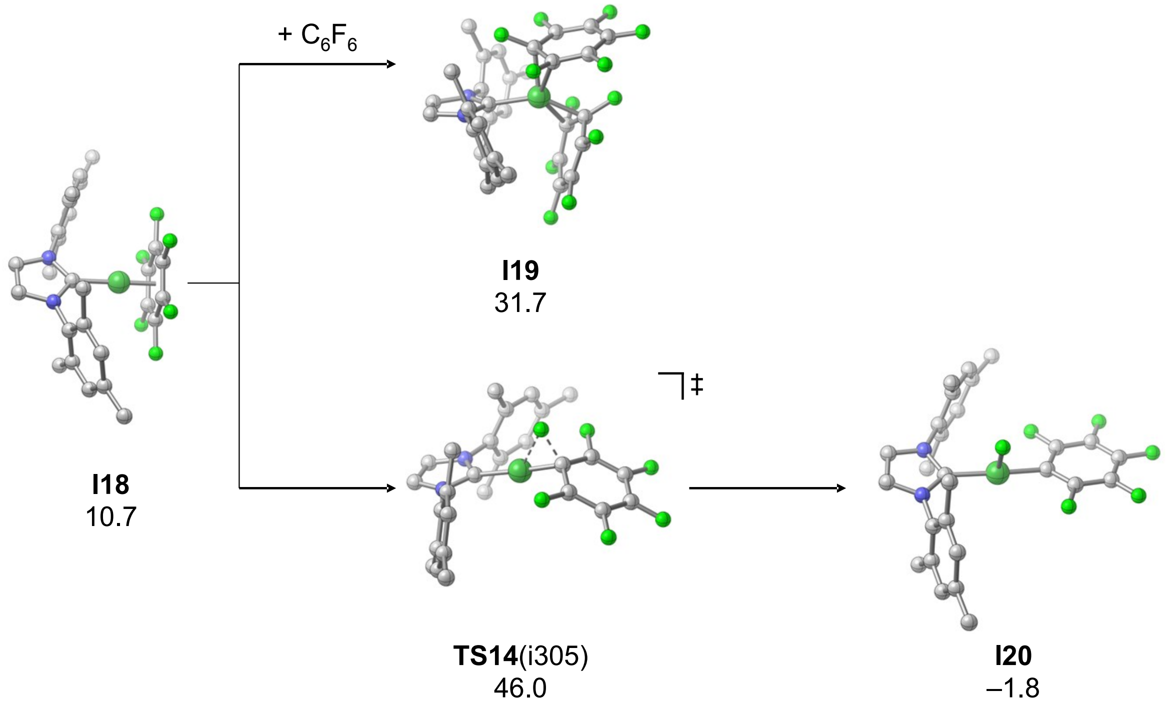

Figure S33: DFT calculations on the reaction of [Ni(Mes$_2$Im)$_2$] (**1**) with C_6F_6: Formation of [Ni(Mes$_2$Im)(η^6-C_6F_6)], addition of C_6F_6 (top) and insertion into the C-F bond of C_6F_6 (bottom) (ΔG^{298} in kcal mol^{-1}; H atoms omitted for clarity).

2) Experimental Section

General Information

[$Ni(Mes_2Im)_2$] (**1**) was prepared according to a literature procedure.[S2] All other starting materials were purchased from commercial sources and used without further purification. All solvents for synthetic reaction were purified by distillation from an appropriate drying agent (sodium with benzophenone as an indicator). All reactions and subsequent manipulations were performed under an argon atmosphere in BRAUN (Uni Lab MB 20G) or Innovative Technology (Pure Lab HE-2GB) glove boxes or using standard Schlenk techniques. NMR spectra were recorded on Bruker Avance 200 (^{1}H, 200.1 MHz, ^{19}F, 188.1 MHz), Bruker Avance 400 (^{1}H, 400.1 MHz, ^{19}F, 376.8 MHz, ^{13}C, 100.7 MHz) or Bruker Avance 500 (^{1}H, 500.1 MHz, ^{19}F, 470.6 MHz, ^{13}C, 125.8 MHz) spectrometers using C_6D_6 or thf-d_8 as the solvent. Assignment of the ^{1}H NMR data was supported by $^{1}H,^{1}H$ and $^{13}C,^{1}H$ correlation experiments. ^{13}C NMR spectra were broad-band proton decoupled ($^{13}C\{^{1}H\}$). Assignment of the ^{13}C NMR data was supported by $^{13}C,^{1}H$ correlation experiments. Chemical shifts are listed in parts per million (ppm) and were determined relative to internal C_6D_5H (^{1}H, δ = 7.16 ppm; C_6D_6) and C_4D_7HO (^{13}C, δ = 1.72, 3.58 ppm; thf-d_8) or to natural-abundance carbon resonances C_6D_6 (^{13}C, δ = 128.06 ppm) and thf-d_8 (^{13}C, δ = 25.31, 67.21 ppm) or external $CFCl_3$ (^{19}F, δ = 0 ppm). Coupling constants are quoted in Hertz. The ^{13}C NMR signals of the perfluoroarenes (if not mentionend) were not detected. Magnetic moment in solution of compound **11** was determined by the Evans method in C_6D_6 at 298 K with a C_6D_6 capillary as reference.[S3] Infrared spectra were recorded on a BRUKER ALPHA FT-IR and are reported in cm^{-1}. Elemental analyses were performed in the microanalytical laboratory of the Institute of Inorganic Chemistry of the University of Würzburg with an Elemental vario micro cube. EPR measurements at X-band (9.4 GHz) were carried out using a BRUKER ELEXSYS E580 CW EPR spectrometer equipped with an Oxford Instruments helium cryostat (ESR900) and a MercuryiTC temperature controller. The spectral simulations were performed using MATLAB 9.6 (R2019a) and the EasySpin 5.2.25 toolbox.[S4]

Experimental Procedures and Characterization of Products

Synthesis of [Ni(Mes$_2$Im)$_2$(F)(4-CF$_3$-C$_6$F$_4$)] (2)

Perfluorotoluene (53.1 μL, d = 1.67 g/cm^3, 88.6 mg, 375 μmol) was added to a solution of [Ni(Mes$_2$Im)$_2$] (**1**) (250 mg, 375 μmol) in 15 mL of pentane at room temperature and the reaction mixture was stirred for 5 h. Then, 30 mL of pentane were added and the precipitate was removed by filtration through a pad of Celite. After washing with 2x 30 mL of pentane the volatiles were removed *in vacuo* and the remaining solid was suspended in 8 mL of cold (0 °C) pentane. The product was collected by filtration, washed with 4 mL of cold (0 °C) pentane and dried *in vacuo* to give a yellow product (139 mg, 154 μmol, 41 %, 903.63 g/mol).

^{1}H NMR (400.4 MHz, C_6D_6, 296 K): δ = 1.96 (s, 24 H, CH_{3ortho}), 2.35 (s, 12 H, CH_{3para}), 5.86 (s, 4 H, NCHCHN), 6.87 (s, 8 H, aryl$_{NHC}$CH_{meta}).

^{19}F{^{1}H} NMR (376.8 MHz, C_6D_6, 296 K) δ = -55.1 (t, 3 F, $^4J_{FF}$ = 21 Hz, CF_3), -107.5 (m, 2 F, aryl-F_{ortho}), -149.2 (m, 2 F, aryl-F_{meta}), -360.8 (s, 1 F, Ni-F).

^{13}C{^{1}H}-NMR (100.7 MHz, C_6D_6, 296 K) δ = 18.1 (aryl$_{NHC}$-CH$_{3ortho}$), 21.3 (aryl$_{NHC}$-CH$_{3para}$), 122.5 (NCHCHN), 128.8 (aryl$_{NHC}$-C_{meta}), 136.6 (aryl$_{NHC}$-C_{ortho}), 136.9 (aryl$_{NHC}$-C_{para}), 138.0 (aryl$_{NHC}$-C_{ipso}), 175.3 (d, NCN).

IR (ATR [cm^{-1}]): 3134 (w), 2961 (w), 2918 (w), 2858 (w), 1626 (w), 1610 (w), 1567 (w),1486 (m), 1434 (s), 1399 (m), 1378 (w), 1313 (vs), 1261 (s), 1155 (m), 1119 (s), 1015 (m), 946 (s), 847 (m), 806 (m).

$C_{49}H_{48}F_8N_4Ni$ [853.62 g/mol] Calcd. (found): C, 65.13 (64.09); H, 5.35 (5.20); N, 6.20 (6.24).

Synthesis of [Ni(Mes$_2$Im)$_2$(F)(C$_6$F$_5$)] (3), [Ni(Mes$_2$Im)$_2$(F)$_2$] (9) and [Ni^I(Mes$_2$Im)$_2$(C$_6$F$_5$)] (11)

Hexafluorobenzene (51.8 μL, d = 1.616 g/cm^3, 83.7 mg, 450 μmol) was added to a solution of [Ni(Mes$_2$Im)$_2$] (**1**) (297 mg, 445 μmol) in 20 mL of thf at room temperature and the reaction mixture was stirred overnight. The precipitate was removed by filtration through a pad of Celite. The volatiles were removed *in vacuo* and the remaining solid was suspended in 35 mL of hexane. The product [Ni(Mes$_2$Im)$_2$(F)$_2$] (**9**) was collected by filtration, washed with 3x 40 mL of hexane and dried *in vacuo* to give a pale yellow product (**9**: 54 mg, 77 μmol, 17 %, 705.56 g/mol). The volatiles of the filtrate were removed *in vacuo* and the remaining solid (190 mg) was resolved in 50 mL of hexane and stored for 72 h at -30 °C to give **3** as yellow crystals and **11** as orange crystals. The supernatant was separated and the crystals of **3** and **11** were manually separated in a glovebox using a spatula (see SI, Figure S7). The

crystallization and separation of **3** (53 mg, 62 μmol, 14 %, 853.62 g/mol) and **11** (37 mg, 45 μmol, 10 %, 834.63 g/mol) was repeated until no more crystals precipitated.

[Ni(Mes$_2$Im)$_2$(F)(C$_6$F$_5$)] (3)

Yield: 53 mg (62 μmol,14 %, 853.62 g/mol).

^{1}H NMR (500.1 MHz, C_6D_6, 296 K): δ = 1.96 (s, 24 H, C*H*$_{3ortho}$), 2.38 (s, 12 H, C*H*$_{3para}$), 5.88 (s, 4 H, NC*H*C*H*N), 6.89 (s, 8 H, aryl$_{NHC}$-C*H*$_{meta}$).

^{19}F{^{1}H} NMR (470.6 MHz, C_6D_6, 296 K) δ = -107.9 (d, 2 F, $^2J_{FF}$ = 31.4 Hz, aryl-*F*$_{ortho}$), -165.8 (t, 1 F, $^2J_{FF}$ = 20.2 Hz, aryl-*F*$_{para}$), -168.1 (m, 2 F, aryl-*F*$_{meta}$), -361.9 (s, 1 F, Ni-*F*).

^{13}C{^{1}H} NMR (125.8 MHz, C_6D_6, 296 K): δ = 18.1 (aryl$_{NHC}$-*C*H$_{3ortho}$), 21.4 (aryl$_{NHC}$-*C*H$_{3para}$), 122.4 (N*C*H*C*HN), 128.8 (aryl$_{NHC}$-*C*$_{meta}$), 136.8 (aryl$_{NHC}$-*C*$_{ortho}$), 137.0 (aryl$_{NHC}$-*C*$_{para}$), 137.9 (aryl$_{NHC}$-*C*$_{ipso}$), 176.4 (N*C*N).

IR (ATR [cm^{-1}]): 2948 (w), 2915 (w), 2857 (w), 1491 (vs) 1447 (vs), 1400 (m), 1380 (m) 1320 (m), 1266 (m), 1223 (w), 1054 (m), 1044 (m), 953 (vs), 927 (w), 848 (s), 779 (m), 704 (s).

$C_{48}H_{48}F_6N_4Ni$ [853.62 g/mol] Calcd. (found): C, 67.54 (67.08); H, 5.67 (5.95); N, 6.56 (6.68).

[Ni(Mes$_2$Im)$_2$(F)$_2$] (9)

Yield: 54 mg, (77 μmol, 17 %, 705.56 g/mol).

^{1}H NMR (400.4 MHz, C_6D_6, 296 K): δ = 2.04 (s, 24 H, C*H*$_{3ortho}$), 2.48 (s, 12 H, C*H*$_{3para}$), 6.02 (s, 4 H, NC*H*NC*H*), 7.01 (s, 8 H, aryl$_{NHC}$C*H*$_{meta}$).

^{19}F{^{1}H} NMR (376.8 MHz, C_6D_6, 296 K) δ = -560 (s, 2 F, Ni–*F*).

^{13}C{^{1}H} NMR (100.7 MHz, C_6D_6, 296 K) δ = 18.0 (aryl$_{NHC}$-*C*H$_{3ortho}$), 21.5 (aryl$_{NHC}$-*C*H$_{3para}$), 120.9 (N*C*H*C*HN), 129.1 (aryl$_{NHC}$-*C*$_{meta}$), 137.0 (aryl$_{NHC}$-*C*$_{para}$), 137.2 (aryl$_{NHC}$-*C*$_{ortho}$), 137.6 (aryl$_{NHC}$-*C*$_{ipso}$), 174.6 (N*C*N).

IR (ATR [cm^{-1}]): 2922 (w), 1605 (w), 1547 (w), 1487 (m), 1461 (m), 1382 (w), 1232 (w), 990 (w), 964 (w), 949 (w), 929 (w),855 (w), 800 (w), 707 (s).

$C_{42}H_{48}F_2N_4Ni$ [705.56 g/mol] Calcd. (found): C, 71.50 (71.53), H, 6.86 (6.98), N, 7.94 (7.97).

[Ni$^{(I)}$(Mes$_2$Im)$_2$(C$_6$F$_5$)] (11)

Yield: 37 mg (45 μmol, 10 %, 834.63 g/mol).

IR (ATR [cm^{-1}]): 3133 (w), 3020 (w), 2957 (w), 2918 (w), 2858 (w),1479 (vs), 1433 (s), 1423 (s), 1392 (s), 1378 (m), 1358 (m), 1339 (m), 1301 (m), 1293 (m), 1258 (m), 1245 (s), 1029 (s), 939 (s), 927 (s), 847 (s).

$C_{48}H_{48}F_5N_4Ni$ [834.63 g/mol] Calcd. (found): C, 69.08 (69.41), H, 5.80 (5.74), N, 6.71 (6.56).

Synthesis of [Ni(Mes$_2$Im)$_2$(F)(2,3,5,6-C$_5$F$_4$N)] (4)

Perfluorpyridine (20 μL, d = 1.54 g/cm^3, 31 mg, 180 μmol) was added to a solution of [Ni(Mes$_2$Im)$_2$] (**1**) (118 mg, 177 μmol) in 12 mL of pentane at room temperature and the reaction mixture was stirred overnight. The volatiles were removed *in vacuo* and the remaining solid was suspended in 10 mL of pentane. The product was collected by filtration, washed with 4 mL of pentane twice and dried *in vacuo* to give a yellow product (103 mg, 123 μmol, 70 %, 836.62 g/mol).

^{1}H NMR (400.4 MHz, C_6D_6, 296 K): δ = 1.96 (s, 24 H, CH_{3ortho}), 2.36 (s, 12 H, CH_{3para}), 5.86 (s, 4 H, NC*H*NC*H*), 6.88 (s, 8 H, aryl$_{NHC}$CH_{meta}).

^{19}F{^{1}H} NMR (376.8 MHz, C_6D_6, 296 K) δ = -104.2 (m, 2 F,) -114.6 (m, 2 F,), -357.9 (s, 1 F, Ni-*F*).

^{13}C{^{1}H} NMR (100.7 MHz, C_6D_6, 296 K) δ = 18.1 (aryl$_{NHC}$-CH$_{3ortho}$), 21.3 (aryl$_{NHC}$-CH$_{3para}$), 122.6 (N*C*H*C*HN), 128.9 (aryl$_{NHC}$-C_{meta}), 136.7 (aryl$_{NHC}$-C_{ortho}), 136.8 (aryl$_{NHC}$-C_{para}), 138.1 (aryl$_{NHC}$-C_{ipso}), 175.3 (N*C*N).

IR (ATR [cm^{-1}]): 2915 (w), 2855(w), 1613 (w), 1591 (w), 1487 (m), 1430 (s), 1418 (s), 1406 (s), 1380 (m), 1320 (m), 1267 (m), 1225 (w), 1198 (s), 1082 (w), 1034 (m), 928 (m), 917 (s), 847 (vs), 814 (m) , 744 (m), 703 (vs), 575 (m), 528 (s), 506 (s).

$C_{47}H_{48}F_5N_5Ni$ [836.62 g/mol] Calcd. (found): C, 67.48 (67.62); H, 5.78 (6.04); N, 8.37 (8.41).

Synthesis of [Ni(Mes$_2$Im)$_2$(F)(2,3,5,6-C$_6$F$_4$H)] (5)

Pentafluorobenzene (38 μL, d = 1.514 g/cm^3, 58 mg, 345 μmol) was added to a solution of [Ni(Mes$_2$Im)$_2$] (**1**) (225 mg, 337 μmol) in 20 mL of hexane at room temperature and the reaction mixture was stirred overnight. The volatile were removed *in vacuo* and the remaining solid was

suspended in 10 mL of pentane. The product was collected by filtration, washed with 3x 10 mL of hexane and dried *in vacuo* to give a yellow product (180 mg, 213 μmol, 64 %, 835.63 g/mol).

^{1}H NMR (400.4 MHz, C_6D_6, 296 K): δ = 2.00 (s, 24 H, C*H*$_{3ortho}$), 2.40 (s, 12 H, C*H*$_{3para}$), 5.91 (s, 4 H, NC*H*NC*H*), 6.16-6.20 (m, 1 H, aryl$_F$-*H*), 6.92 (s, 8 H, aryl$_{NHC}$C*H*$_{meta}$).

^{19}F{^{1}H} NMR (376.8 MHz, C_6D_6, 296 K) δ = -109.9 (m, 2 F), -145.9 (m, 2 F), -357.2 (s, 1 F, Ni-*F*).

^{13}C{^{1}H} NMR (100.7 MHz, C_6D_6, 296 K) δ = 18.1 (aryl$_{NHC}$-*C*H$_{3ortho}$), 21.4 (aryl$_{NHC}$-*C*H$_{3para}$), 122.4 (N*C*H*C*HN), 128.8 (aryl$_{NHC}$-*C*$_{meta}$), 136.9 (aryl$_{NHC}$-*C*$_{ortho}$), 137.1 (aryl$_{NHC}$-*C*$_{para}$), 137.8 (aryl$_{NHC}$-*C*$_{ipso}$), 177.2 (N*C*N).

IR (ATR [cm^{-1}]): 2913 (w), 2855 (w), 1608 (w), 1588 (w), 1486 (s), 1434 (s), 1399 (m), 1263 (m), 1156 (m), 1035 (m), 927 (m), 889 (s), 878 (s), 847 (vs), 802 (s), 703 (vs).

$C_{48}H_{49}F_5N_4Ni$ [835.63 g/mol] Calcd. (found): C, 68.99 (69.02); H, 5.91 (6.18); N, 6.70 (6.95).

Synthesis of [Ni(Mes$_2$Im)$_2$(F)(2,3,5-C$_6$F$_3$H$_2$)] (6)

The compound of 1,2,3,5-tetrafluorobenzene (39 μL, d = 1.393 g/cm^3, 54 mg, 360 μmol) was added to a solution of [Ni(Mes$_2$Im)$_2$] (**1**) (239 mg, 350 μmol) in 20 mL of toluene at room temperature and the reaction mixture was stirred overnight. The volatiles were removed *in vacuo* and the remaining solid was suspended in 5 mL of hexane. The product was collected by filtration, washed with 3x 10 mL of hexane and dried *in vacuo* to give a yellow product (200 mg, 245 μmol, 70 %, 817.64 g/mol).

^{1}H NMR (500.1 MHz, C_6D_6, 296 K): δ = 1.90 (s, br 12 H, C*H*$_{3ortho}$), 2.12 (s, br 12 H, C*H*$_{3ortho}$), 2.42 (s, 12 H, C*H*$_{3para}$), 5.52 (d, br 1 H, aryl$_F$-*H*$_{ortho}$) 5.90 (s, 4 H, NC*H*NC*H*), 6.13 - 6.18 (m, 1 H, aryl$_F$-*H*$_{para}$), 6.87 (s, 4 H, aryl$_{NHC}$C*H*$_{meta}$), 6.94 (s, 4 H, aryl$_{NHC}$C*H*$_{meta}$).

^{19}F{^{1}H} NMR (470.6 MHz, C_6D_6, 296 K) δ = -116.8 - -116.9 (m, 1 F, aryl-*F*$_{meta(3)}$) -122.8 - -122.8 (m, 1 F, aryl-*F*$_{meta(5)}$), -141.1 (dd, 1 F, aryl-*F*$_{ortho}$), -344.2 (s, 1 F, Ni-*F*).

^{13}C{^{1}H} NMR (100.7 MHz, C_6D_6, 296 K): δ = 18.1 (aryl$_{NHC}$-*C*H$_{3ortho}$), 21.5 (aryl$_{NHC}$-*C*H$_{3para}$), 96.2 (aryl$_{ArF}$-*C*H$_{para}$), 121.0 (aryl$_{ArF}$-*C*H$_{ortho}$), 122.2 (N*C*H*C*HN), 128.9 (aryl$_{NHC}$-*C*$_{meta}$), 129.1 (aryl$_{NHC}$-*C*$_{ortho}$), 137.1 (aryl$_{NHC}$-*C*$_{para}$), 137.8 (aryl$_{NHC}$-*C*$_{ipso}$), 180.3 (N*C*N).

IR (ATR [cm^{-1}]): 3008 (w), 2944 (w), 2914 (m), 2855 (w), 1607 (m), 1574 (m), 1486 (m), 1433 (m), 1399 (m), 1380 (m), 1319 (m), 1264 (m), 1221 (m), 1191 (m), 1160 (m), 1093 (m), 1036 (m), 975 (m), 928 (m), 887 (m), 848 (s), 799 (m), 759 (m), 703 (s), 577 (m), 518 (s), 496 (s).

$C_{48}H_{50}F_4N_4Ni$ [817.64 g/mol] Calcd. (found): C, 70.51 (70.09); H, 6.16 (5.83); N, 6.85 (6.93).

Synthesis of [Ni(Mes$_2$Im)$_2$(F)(3,5-C$_6$F$_2$H$_3$)] (7)

The compound 1,3,5-trifluorobenzene (57 μL, d = 1.277 g/cm^3, 73 mg, 550 μmol) was added to a solution of [Ni(Mes$_2$Im)$_2$] (**1**) (150 mg, 225 μmol) in 15 mL of toluene at room temperature and the reaction mixture was stirred for 4 weeks. The volatiles were removed *in vacuo* and the remaining solid was suspended in 5 mL of hexane. The product was collected by filtration, washed with 3x 10 mL of hexane and dried *in vacuo* to give a yellow product (94 mg, 117 μmol, 52 %, 799.65 g/mol).

^{1}H NMR (400.4 MHz, C_6D_6, 296 K): δ = 1.48 (s, br 12 H, CH_{3ortho}), 2.42 (s, 12 H, CH_{3para}), 2.52 (s, br 12 H, CH_{3ortho}), 5.83 (d, br, 2 H, aryl$_F$-H_{ortho}) 5.90 (s, 4 H, NC*H*NC*H*), 6.12 (tt, 1 H, aryl$_F$-H_{para}), 6.91 (s, 8 H, aryl$_{NHC}$CH_{meta}).

^{19}F{^{1}H} NMR (376.8 MHz, C_6D_6, 296 K) δ = -118.7 (s, 2 F, aryl-F_{meta}), -333.1 (s, 1 F, Ni-*F*).

^{19}F NMR (188.1 MHz, C_6D_6, 296 K) δ = -118.7 (m, 2 F, aryl-F_{meta}), -333.1 (s, 1 F, Ni-*F*).

^{13}C{^{1}H} NMR (100.7 MHz, C_6D_6, 296 K) δ = 18.1 (br, aryl$_{NHC}$-CH_{3ortho}), 21.4 (aryl$_{NHC}$-CH_{3para}), 94.1 (aryl$_F$-CH_{para}), 120.6 (aryl$_F$-CH_{ortho}), 121.8 (N*C*H*C*HN), 127.8 – 128.3 (covered by C_6D_6, aryl$_{NHC}$-$CH_{meta+ortho}$), 137.1 (aryl$_{NHC}$-C_{ipso}), 137.7 (aryl$_{NHC}$-CH_{para}) 183.2 (N*C*N).

^{1}H NMR (400.4 MHz, thf-d$_8$, 296 K): δ = 1.34 (s, br 12 H, CH_{3ortho}), 2.21 (s, br 12 H, CH_{3ortho}), 2.52 (s, 12 H, CH_{3para}), 5.51 (d, br, 2 H, aryl$_F$-H_{ortho}), 5.65 (tt, 1 H, $^3J_{HF}$ = 9.6 Hz, $^4J_{HH}$ = 2.3 Hz, aryl$_F$-H_{para}) 6.74 (s, 4 H, NC*H*NC*H*), 6.89 (s, 8 H, aryl$_{NHC}$CH_{meta}).

^{19}F{^{1}H} NMR (376.8 MHz, thf-d$_8$, 296 K) δ = -119.9 (s, 2 F, aryl-F_{meta}), -335.0 (s, 1 F, Ni-*F*).

^{19}F-NMR (376.8 MHz, thf-d$_8$, 296 K) δ = -119.9 (t, 2 F, $^3J_{FH}$ = 7.4 Hz, aryl-F_{meta}), -335.0 (s,br 1 F, Ni-*F*).

^{13}C{^{1}H} NMR (100.7 MHz, thf-d$_8$, 296 K) δ = 18.1 (br, aryl$_{NHC}$-CH_{3ortho}), 21.3 (aryl$_{NHC}$-CH_{3para}), 93.6 (aryl$_F$-CH_{para}), 120.7 (aryl$_F$-CH_{ortho}), 122.7 (N*C*H*C*HN), 129.3 (br, aryl$_{NHC}$-$CH_{meta+ortho}$), 137.6 (aryl$_{NHC}$-C_{ipso}), 137.9 (aryl$_{NHC}$-CH_{para}) 183.3 (N*C*N).

IR (ATR [cm^{-1}]): 3024 (w), 3004 (w), 2948 (w), 2916 (m), 2857 (m), 2198 (w), 1582 (m), 1566 (m), 1486 (s), 1399 (m), 1319 (m), 1264 (m), 1242 (m), 1223 (m), 1093 (m), 1034 (m), 958 (m), 926 (m), 848 (s), 808 (m), 701 (s), 577 (m), 522 (m), 485 (s).

$C_{48}H_{51}F_3N_4Ni$ [799.65 g/mol] Calcd. (found): C, 72.10 (71.97); H, 6.43 (6.58); N, 7.01 (7.00).

Synthesis of [Ni(Mes$_2$Im)$_2$][BF$_4$] (8)

[Ni(Mes$_2$Im)$_2$] (**1**) (1.00 g, 1.50 mmol) was added to suspension of [FeCp$_2$][BF$_4$] (409 mg, 1.50 mmol) in 80 mL of thf and the reaction mixture was stirred overnight. The precipitate was collected by filtration, washed with thf and dried *in vacuo* to give an off-white product (933 mg, 1.24 mmol, 83 %, 754.37 g/mol).

^{11}B{^{1}H} NMR (128.5 MHz, CD$_3$CN, 296 K) δ = -1.15 (s, 1 B, ^{11}B*F*$_4$).

^{19}F{^{1}H} NMR (376.8 MHz, CD$_3$CN, 296 K) δ = -151.8 (s, 4 F, ^{11}B*F*$_4$), -151.8 (s, 4 F, ^{10}B*F*$_4$).

IR (ATR [cm^{-1}]): 3171 (w), 2975 (w), 2918 (w), 2885 (w), 1609 (w), 1486 (m), 1439 (w), 1397 (m), 1309 (w), 1231 (w), 1117 (m), 1095 (m), 1050 (s), 1034 (s), 927 (m), 879 (w), 862 (m), 758 (w), 738 (m) 644 (w), 575 (m), 518 (m), 424 (w).

$C_{42}H_{48}BF_4N_4Ni$ [754.37 g/mol] Calcd. (found): C, 66.87 (66.54); H, 6.41 (6.73); N, 7.43 (7.60).

HRMS-ESI m/z (%) calcd. for [$C_{42}H_{48}N_4Ni$]$^+$: 666.3233(100), 667.3266(45), 668.3187(38), 669.3221(17); found: 666.3218(100), 667.3261(45), 668.3181(38), 669.3215(18).

m/z (%) calcd. for [BF$_4$]$^-$ 86.0065(25), 87.0029(100); found: 86.0057(25), 87.0035(100).

Synthesis of [Ni(Mes$_2$Im)$_2$(F)$_2$] (9)

[Ni(Mes$_2$Im)$_2$(I)$_2$] (**10**) (52 mg, 56.8 μmol) in 10 mL of CH_2Cl_2 was added to a suspension of AgF (40 mg, 170 μmol) in 15 mL of CH_2Cl_2 at 0 °C and the reaction mixture was allowed to warm to room temperature overnight. The precipitate was removed by filtration through a pad of Celite. After washing with 10 mL of CH_2Cl_2, the volatiles were removed *in vacuo* and the remaining solid was suspended in 10 mL of hexane. The product was collected by filtration, washed with 2x 4 mL of hexane and dried *in vacuo* to give a pale yellow product (15 mg, 21 μmol, 38 %, 705.56 g/mol).

^{1}H NMR (400.4 MHz, C$_6$D$_6$, 296 K): δ = 2.04 (s, 24 H, C*H*$_{3ortho}$), 2.48 (s, 12 H, C*H*$_{3para}$), 6.02 (s, 4 H, NC*H*NC*H*), 7.01 (s, 8 H, aryl$_{NHC}$C*H*$_{meta}$).

^{19}F{^{1}H} NMR (376.8 MHz, C$_6$D$_6$, 296 K) δ = -560.0 (s, 2 F, Ni-*F*).

^{13}C{^{1}H} NMR (100.7 MHz, C$_6$D$_6$, 296 K) δ = 18.0 (aryl$_{NHC}$-*C*H$_{3ortho}$), 21.5 (aryl$_{NHC}$-*C*H$_{3para}$), 120.9 (N*C*H*C*HN), 129.1 (aryl$_{NHC}$-*C*$_{meta}$), 137.0 (aryl$_{NHC}$-*C*$_{para}$), 137.3 (aryl$_{NHC}$-*C*$_{ortho}$), 137.6 (aryl$_{NHC}$-*C*$_{ipso}$), 174.6 (N*C*N).

IR (ATR [cm^{-1}]): 2922 (w), 1605 (w), 1547 (w), 1487 (m), 1461 (m), 1382 (w), 1232 (w), 990 (w), 964 (w), 949 (w), 929 (w),855 (w), 800 (w), 707 (s).

$C_{42}H_{48}F_2N_4Ni$ [705.56 g/mol] Calcd. (found): C, 71.50 (71.53), H, 6.86 (6.98), N, 7.94 (7.97).

Synthesis of [Ni(Mes$_2$Im)$_2$(I)$_2$] (10)

I_2 (76.1 mg, 300 μmol) in 5 mL of thf was added to a solution of [Ni(Mes$_2$Im)$_2$] (**1**) (200 mg, 300 μmol) in 15 mL of thf at room temperature and the reaction mixture was stirred overnight. The volatiles were removed *in vacuo* and the remaining solid was suspended in 10 mL of hexane. The product was collected by filtration, washed with 2x 4 mL of pentane and dried *in vacuo* to give a yellow product (220 mg, 239 μmol, 80 %, 921.38 g/mol).

^{1}H NMR (400.4 MHz, C_6D_6, 296 K): δ = 2.29 (s, 24 H, CH_{3ortho}), 2.38 (s, 12 H, CH_{3para}), 6.03 (s, 4 H, NC*H*NC*H*), 6.95 (s, 8 H, aryl$_{NHC}$CH_{meta}).

^{13}C{^{1}H} NMR (100.7 MHz, C_6D_6, 296 K) δ = 21.3 (aryl$_{NHC}$-CH_{3ortho}), 22.8 (aryl$_{NHC}$-CH_{3para}), 124.2 (N*C*H*C*HN), 129.9 (aryl$_{NHC}$-C_{meta}), 136.7 (aryl$_{NHC}$-C_{ortho}), 137.5 (aryl$_{NHC}$-C_{para}), 137.6 (aryl$_{NHC}$-C_{ipso}), 176.5 (N*C*N).

IR (ATR [cm^{-1}]): 3140 (w), 2956 (w), 2915 (w), 2856 (w),1607 (w), 1481 (m), 1443 (w), 1396 (m), 1377 (m), 1312 (m), 1260 (m), 1165 (w), 1080 (m), 1027 (m), 961 (m), 850 (m), 729 (m), 699 (s).

$C_{42}H_{48}I_2N_4Ni$ [921.38 g/mol] Calcd. (found): C, 54.75 (54.89), H, 5.25 (5.34), N, 6.08 (5.64).

Synthesis of [Ni^I(Mes$_2$Im)$_2$(C$_6$F$_5$)] (11)

$PhSiH_3$ (25 μL, d = 0.878 g/cm^3, 22 mg, 200 μmol) was added to a solution of [Ni(Mes$_2$Im)$_2$F(C$_6$F$_5$)] (**3**) (80.0 mg, 93.7 μmol) in 10 mL of benzene and the reaction mixture was stirred for 48 h at room temperature. The volatiles were removed *in vacuo* and the remaining solid was suspended in 10 mL of hexane. The product was collected by filtration, washed with 2x 2 mL of hexane and dried *in vacuo* to give an orange product (15 mg, 18 μmol, 19 %, 834.63 g/mol). The supernatant was cooled to -30 °C overnight and the product precipitate of in the form of an orange solid. The suspension was decanted and the product was dried *in vacuo* (42 mg, 50 μmol, 53 %, 834.63 g/mol).

IR (ATR [cm^{-1}]): 3133 (w), 3020 (w), 2957 (w), 2918 (w), 2858 (w),1479 (vs), 1433 (s), 1423 (s), 1392 (s), 1378 (m), 1358 (m), 1339 (m), 1301 (m), 1293 (m), 1258 (m), 1245 (s), 1029 (s), 939 (s), 927 (s), 847 (s).

$C_{48}H_{48}F_5N_4Ni$ [834.63 g/mol] Calcd. (found): C, 69.08 (69.41), H, 5.80 (5.74), N, 6.71 (6.56).

magnetic moment (Evans): μ_{eff} (C_6D_6, 298 K) = 1.80 μ_B

Synthesis of [Ni^I(Mes$_2$Im)$_2$(2,3,5,6-C$_6$F$_4$H)] (13)

$PhSiH_3$ (48 μL, d = 0.878 g/cm^3, 42 mg, 386 μmol) was added to a solution of [Ni(Mes$_2$Im)$_2$F(2,3,5,6-C$_6$F$_4$H)] (**5**) (81 mg, 97 μmol) in 8 mL of benzene and the reaction mixture was stirred for 5 h at room temperature. The volatiles were removed *in vacuo* and the remaining solid was suspended in 5 mL of hexane. The product was collected by filtration and washed with 2x 2 mL of hexane and dried *in vacuo* to give an orange product (18 mg, 22 μmol, 23 %, 816.64 g/mol). The supernatant was cooled to -30 °C overnight and the product precipitate of in the form of an orange solid. The suspension was decanted and the product was dried *in vacuo* (38 mg, 46 μmol, 48 %, 816.64 g/mol).

IR (ATR [cm^{-1}]): 3131 (w), 2959 (w), 2917 (w), 2857 (w), 1484 (s), 1423 (vs), 1389 (s), 1291 (s), 1258 (vs), 1155 (s), 1144 (s), 1078 (m), 1070 (m), 1030 (m), 1014 (m), 923 (m), 864 (vs), 849 (vs), 798 (s).

$C_{48}H_{49}F_4N_4Ni$ [816.64 g/mol] Calcd. (found): C, 70.60 (70.79), H, 6.05 (6.08), N, 6.86 (6.83).

Synthesis of [Ni^I(Mes$_2$Im)$_2$(2,3,5-C$_6$F$_3$H$_2$)] (14)

$PhSiH_3$ (29 μL, d = 0.878 g/cm^3, 26 mg, 238 μmol) was added to a solution of [Ni(Mes$_2$Im)$_2$F(2,3,5-C$_6$F$_3$H$_2$)] (**6**) (98 mg, 120 μmol) in 8 mL of toluene and the reaction mixture was stirred 5 h at 60 °C. The volatiles were removed *in vacuo* and the remaining solid was suspended in 5 mL of hexane. The product was collected by filtration and washed with 2x 2 mL of hexane and dried *in vacuo* to give an orange product (5 mg, 6 μmol, 5 %, 798.64 g/mol). The supernatant was cooled to -30 °C for 72 h and the product precipitate in the form of a red solid. The suspension was decanted and the product dried *in vacuo* (58 mg, 73 μmol, 61 %, 798.64 g/mol).

IR (ATR [cm^{-1}]): 3130 (w), 2962 (w), 2914 (w), 2856 (w) 1602 (w), 1562 (w), 1485 (m), 1433 (m), 1414 (m), 1279 (m), 1258 (s), 1141 (m), 1078 (s), 1015 (s), 964 (m), 923 (m), 848 (m), 794 (vs).

$C_{48}H_{50}F_3N_4Ni$ [798.64 g/mol] Calcd. (found): C, 72.19 (71.27), H, 6.31 (6.55), N, 7.02 (6.87).

3) Crystallographic Details

The crystal data were collected on a Bruker X8-APEX II diffractometer with a CCD area detector and graphite monochromated Mo-K_{α} radiation. The structures were solved using the intrinsic phasing method (ShelXT), refined with the ShelXL program [S5] and expanded using Fourier techniques. All non-hydrogen atoms were refined anisotropically. Hydrogen atoms were included in structure factor calculations and were assigned idealized geometric positions. Crystallographic data have been deposited with the Cambridge Crystallographic Data Centre as supplementary publication nos. CCDC-2017698 (**3**), CCDC-2017693 (**4**), CCDC-2017696 (**5**), CCDC-2017694 (**8**), CCDC-2017695 (**9**), CCDC-2017700 (**11**), CCDC-2017697 (**13**) and CCDC-2017699 (**14**). These data can be obtained free of charge from The Cambridge Crystallographic Data Centre *via* www.ccdc.cam.ac.uk/data_request/cif.

[Ni(Mes₂Im)₂F(C₆F₅)] (3)

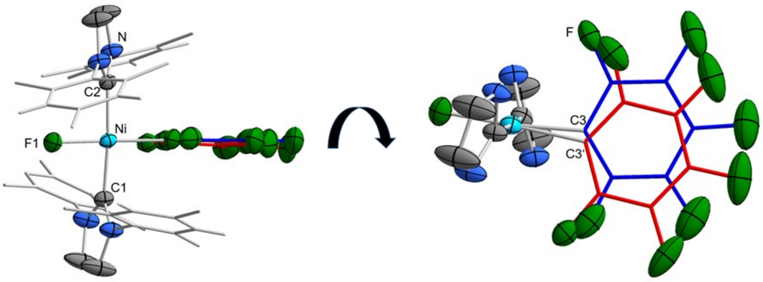

Figure S34: Molecular structure of $[Ni(Mes_2Im)_2F(C_6F_5)]$ (**3**) in the solid state (ellipsoids set at 50 % probability level). Hydrogen atoms are omitted for clarity. Selected bond lengths [Å] and angles [°]: Ni–C1 1.923(3), Ni–C2 1.922(3), Ni–F 1.844(2), Ni–C3 1.944(5), Ni–C3‘ 1.882(7); C1–Ni–C2 175.6(1), C1–Ni–F 87.11(1), C1–Ni–C3 93.02(3), C1–Ni–C3‘ 91.36(2), C2–Ni–F1 88.58(1), C2–Ni–C3 91.37(3), C2–Ni–C3‘ 92.92(2), F1–Ni–C3 173.6(2), F1–Ni–C3‘ 177.8(1) NHC(C1):NHC(C2) 37.07(2), NHC(C1):C1-Ni-F 65.67(1), NHC(C1):C1-Ni-C2-C3-F1 65.51(5), NHC(C2):C2-Ni-F 82.15(1), NHC(C2):C2-Ni-C1-C3-F1 82.27(4).

Crystallographic data for **3**:

Formula: $C_{48}H_{48}F_6N_4Ni$, M_r = 853.61, yellow block, 0.305 x 0.320 x 0.337 mm, orthorhombic space group P_{bca}, a = 20.5349(10) Å, b = 18.6375(9) Å, c = 22.7188(12) Å, α = 90 °, β = 90 °, γ = 90 °, V = 8694.9(8) Å^3, Z = 8, ρ_{calcd} = 1.304 g/cm^3, μ = 0.510 mm^{-1}, F(000) = 3568, T = 200(2) K, 115783 reflections measured in the range 1.726 ° < θ < 26.806 °, completeness 100 %, 9293 independent reflections, 6964 observed reflections [$I > 2\sigma(I)$], 620 parameters, 372 restraints; all data: R_1 = 0.0815 and wR_2 = 0.1358, $I > 2\sigma(I)$: R_1 = 0.0567 and wR_2 = 0.1233, Goof = 1.090, largest difference peak/hole 0.535/-0.497 e Å^{-3}.

[Ni(Mes$_2$Im)$_2$F(C$_6$F$_4$N)] (4)

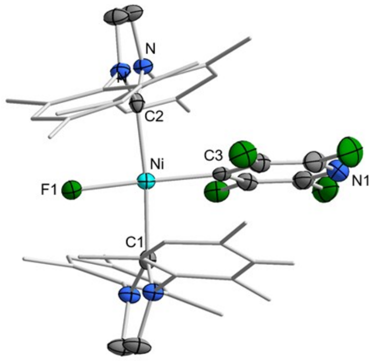

Figure S35: Molecular structure of [Ni(Mes$_2$Im)$_2$F(C$_6$F$_4$N)] (**4**) in the solid state (ellipsoids set at 50 % probability level). Hydrogen atoms are omitted for clarity. Selected bond lengths [Å] and angles [°]: Ni–C1 1.923(3), Ni–C2 1.920(3), Ni–C3 1.883(3), Ni–F1 1.859(2); C1–Ni–C2 174.3(1), C1–Ni–C3 93.73(1), C2–Ni–C3 91.94(1), C1–Ni–F 84.94(1), C2–Ni–F 89.60(1), NHC(C1):NHC(C2) 36.01(2), NHC(C1):C1-Ni-F 86.105(1), NHC(C1):C1-Ni-C2-C3-F1 85.94(7), NHC(C2):C2-Ni-F 64.241(1), NHC(C2):C2-Ni-C1-C3-F1 64.47(7).

Crystallographic data for **4**:

Formula: $C_{47}H_{48}F_5N_5Ni$, M_r = 836.61, yellow block, 0.202 x 0.202 x 0.217 mm, orthorhombic space group P_{bca}, a = 20.0292(16) Å, b = 18.4196(14) Å, c = 22.4625(16) Å, α = 90 °, β = 90 °, γ = 90 °, V = 8287.1(11) Å^3, Z = 8, ρ_{calcd} = 1.341 g/cm^3, μ = 0.531 mm^{-1}, F(000) = 3504, T = 100(2) K, 48652 reflections measured in the range 1.754 ° < θ < 26.784 °, completeness 100 %, 8820 independent reflections, 5766 observed reflections [$I > 2\sigma(I)$], 535 parameters, 0 restraints; all data: R_1 = 0.0968 and wR_2 = 0.1541, $I > 2\sigma(I)$: R_1 = 0.0500 and wR_2 = 0.1240, Goof = 1.035, largest difference peak/hole 0.883/-0.940 e Å^{-3}.

[Ni(Mes$_2$Im)$_2$F(2,3,5,6-C$_6$F$_4$H)] (5)

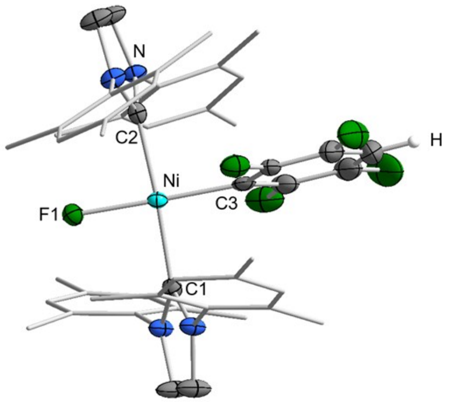

Figure S36: Molecular structure of [Ni(Mes$_2$Im)$_2$F(2,3,5,6-C$_6$F$_4$H)] (**5**) in the solid state (ellipsoids set at 50 % probability level). Hydrogen atoms are omitted for clarity. Selected bond lengths [Å] and angles [°]: Ni–C1 1.921(2), Ni–C2 1.924(2), Ni–C3 1.896(3), Ni–F1 1.856(2); C1–Ni–C2 176.4(1), C1–Ni–C3 91.59(1), C2–Ni–C3 92.03(1), C1–Ni–F 89.41(9), C2–Ni–F 86.98(9), NHC(C1):NHC(C2) 33.81(1), NHC(C1):C1-Ni-F 66.759(1), NHC(C1):C1-Ni-C2-C3-F1 66.70(1), NHC(C2):C2-Ni-F 82.919(1), NHC(C2):C2-Ni-C1-C3-F1 83.00(1).

Crystallographic data for **5**:

Formula: $C_{48}H_{49}F_5N_4Ni$, M_r = 835.62, yellow block, 0.196 x 0.274 x 0.312 mm, orthorhombic space group P_{bca}, a = 20.0814(16) Å, b = 18.4603(14) Å, c = 22.7528(18) Å, α = 90 °, β = 90 °, γ = 90 °, V = 8434.7(11) Å^3, Z = 8, ρ_{calcd} = 1.316 g/cm^3, μ = 0.521 mm^{-1}, F(000) = 3504, T = 100(2) K, 67305 reflections measured in the range 1.745 ° < θ < 26.787 °, completeness 100 %, 8981 independent reflections, 6748 observed reflections [$I > 2\sigma(I)$], 535 parameters, 0 restraints; all data: R_1 = 0.0724 and wR_2 = 0.1166, $I > 2\sigma(I)$: R_1 = 0.0463 and wR_2 = 0.1023, Goof = 1.039, largest difference peak/hole 0.499/-0.526 e Å^{-3}.

[Ni(Mes$_2$Im)$_2$][BF$_4$] (8)

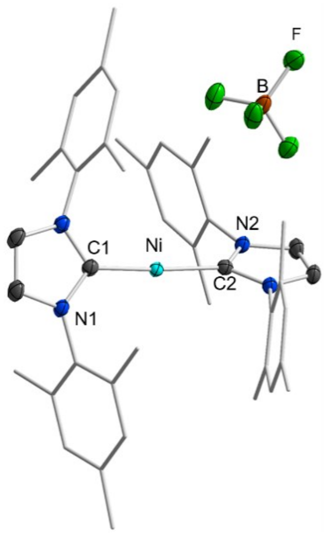

Figure S37: Molecular structure of [Ni(Mes$_2$Im)$_2$][BF$_4$] (**8**) in the solid state (ellipsoids set at 50 % probability level). Hydrogen atoms are omitted for clarity. Selected bond lengths [Å] and angles [°]: **8**: Ni–C1 1.894(3), Ni–C2 1.894(3); C1-Ni1-C2 174.5(1), NHC(C1):NHC(C2) 57.99(1).

Crystallographic data for **8**:

Formula: $C_{42}H_{48}BF_4N_4Ni$, M_r = 754.36, colorless block, 0.120 x 0.486 x 0.512 mm, orthorhombic space group P_{nna}, a = 15.9816(12) Å, b = 31.300(2) Å, c = 15.8502(12) Å, α = 90 °, β = 90 °, γ = 90 °, V = 7928.7(10) Å^3, Z = 8, ρ_{calcd} = 1.264 g/cm^3, μ = 0.542 mm^{-1}, F(000) = 3176, T = 100(2) K, 91900 reflections measured in the range 1.301 ° < θ < 26.089 °, completeness 99.9 %, 7841 independent reflections, 6046 observed reflections [$I > 2\sigma(I)$], 483 parameters, 0 restraints; all data: R_1 = 0.0528 and wR_2 = 0.1166, $I > 2\sigma(I)$: R_1 = 0.0759 and wR_2 = 0.1267, Goof = 1.100, largest difference peak/hole 2.019/-0.630 e Å^{-3}.

trans-[Ni(Mes$_2$Im)$_2$F$_2$] (9)

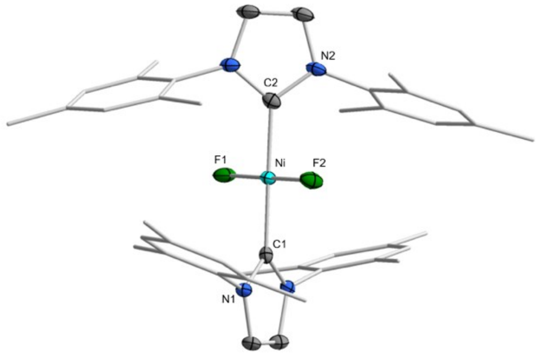

9

Figure S38: Molecular structure of *trans*-[Ni(Mes$_2$Im)$_2$F$_2$] (**9**) in the solid state (ellipsoids set at 50 % probability level). Hydrogen atoms are omitted for clarity. Selected bond lengths [Å] and angles [°]: Ni–C1 1.9028(3), Ni–C2 1.9018(3), Ni–F1 1.8449(2), Ni–F2 1.8233(2); C1-Ni-C2 178.5(1), C1-Ni-F1 90.03(1), C1-Ni-F2 90.17(1), C2-Ni-F1 89.33(1), C2-Ni-F2 90.48(1), F1-Ni-F2 179.8(8), NHC(C1):NHC(C2) 53.34(1).

Crystallographic data for **9**:

Formula: $C_{42}H_{48}F_2N_4Ni$, M_r = 705.55, orange block, 0.251 x 0.256 x 0.302 mm, orthorhombic space group P_{bca}, a = 17.054(2) Å, b = 18.722(3) Å, c = 22.837(3) Å, α = 90 °, β = 90 °, γ = 90 °, V = 7291.4(17) Å^3, Z = 8, ρ_{calcd} = 1.285 g/cm^3, μ = 0.578 mm^{-1}, F(000) = 2992, T = 100(2) K, 96913 reflections measured in the range 2.176 ° < θ < 27.173 °, completeness 99.7 %, 8064 independent reflections, 5785 observed reflections [$I > 2\sigma(I)$], 454 parameters, 0 restraints; all data: R_1 = 0.0576 and wR_2 = 0.1478, $I > 2\sigma(I)$: R_1 = 0.0891 and wR_2 = 0.1766, Goof = 1.051, largest difference peak/hole 0.729/-0.921 e Å^{-3}.

[Ni^I(Mes$_2$Im)$_2$(C$_6$F$_5$)] (11)

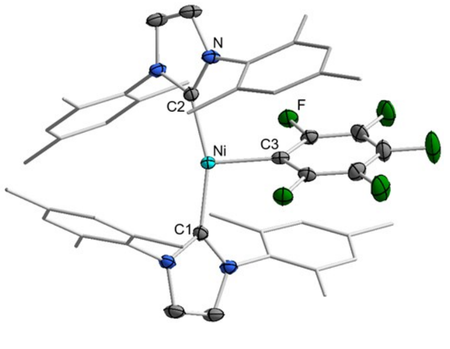

Figure S39: Molecular structure of [Ni^I(Mes$_2$Im)$_2$(C$_6$F$_5$)] (**11**) in the solid state (ellipsoids set at 50 % probability level). Hydrogen atoms are omitted for clarity. Selected bond lengths [Å] and angles [°]: Ni–C1 1.923(2), Ni–C2 1.923(2), Ni–C3 1.984(3); C1-Ni-C2 159.8(8), C1-Ni-C3 100.1(6), C2-Ni-C3 100.1(6), NHC(C1):NHC(C2) 82.37(1) NHC(C1):C1-Ni-C3 41.59(6), NHC(C1):C2-Ni-C1-C3 41.67(8), NHC(C2):C2-Ni-C3 41.59(6), NHC(C2):C2-Ni-C1-C3 41.67(8).

Crystallographic data for **11**:

Formula: $C_{48}H_{48}F_5N_4Ni$, M_r = 834.61, orange block, 0.088 x 0.150 x 0.195 mm, monoclinic space group *C*2/c, a = 17.3579(3) Å, b = 13.3131(2) Å, c = 19.0795(3) Å, α = 90 °, β = 105.933 °, γ = 90 °, V = 4239.65(12) Å^3, Z = 4, ρ_{calcd} = 1.308 g/cm^3, μ = 1.159 mm^{-1}, *F*(000) = 1748, T = 100(10) K, 25553 reflections measured in the range 4.103 ° < θ < 77.702 °, completeness 99.9%, 4419 independent reflections, 4147 observed reflections [$I > 2\sigma(I)$], 270 parameters, 0 restraints; all data: R_1 = 0.0430 and wR_2 = 0.1096, $I > 2\sigma(I)$: R_1 = 0.0400 and wR_2 = 0.1067, Goof = 1.083, largest difference peak/hole 1.409/-0.449 e Å^{-3}.

[Ni(Mes$_2$Im)$_2$(2,3,5,6-C$_6$F$_4$H)] (13)

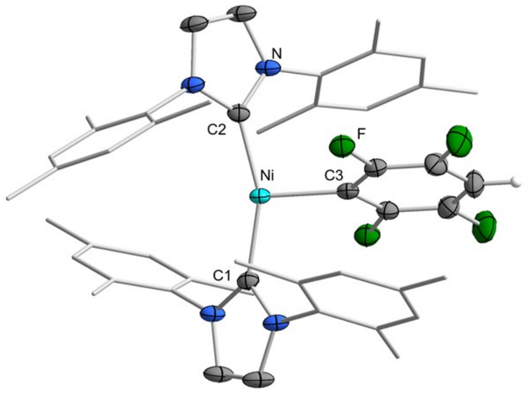

Figure S40: Molecular structure of [Ni(Mes$_2$Im)$_2$(2,3,5,6-C$_6$F$_4$H)] (**13**) in the solid state (ellipsoids set at 50 % probability level). Hydrogen atoms are omitted for clarity. Selected bond lengths [Å] and angles [°]: Ni–C1 1.930(2), Ni–C2 1.930(2), Ni–C3 1.987(3); C1-Ni-C2 157.3(8), C1-Ni-C3 101.3(6), C2-Ni-C3 101.3(6), NHC(C1):NHC(C2) 82.10(1), NHC(C1):C1-Ni-C3 41.58(8), NHC(C1):C1-Ni-C2-C3 41.58(8), NHC(C2):C2-Ni-C1 41.58(9), NHC(C2):C2-Ni-C1-C3 41.58(9).

Crystallographic data for **13**:

Formula: $C_{48}H_{49}F_4N_4Ni$, M_r = 816.62, orange block, 0.33 x 0.35 x 0.40 mm, monoclinic space group *C*2/c, a = 21.976(3) Å, b = 13.1060(15) Å, c = 17.395(3) Å, α = 90 °, β = 122.824(3) °, γ = 90 °, V = 4210.2(11) Å^3, Z = 4, ρ_{calcd} = 1.288 g/cm^3, μ = 0.517 mm^{-1}, *F*(000) = 1716, T = 100(2) K, 20673 reflections measured in the range 1.905 ° < θ < 26.855 °, completeness 100 %, 4504 independent reflections, 3821 observed reflections [$I > 2\sigma(I)$], 265 parameters, 0 restraints; all data: R_1 = 0.0533 and wR_2 = 0.1214, $I > 2\sigma(I)$: R_1 = 0.0440 and wR_2 = 0.1150, Goof = 1.040, largest difference peak/hole 1.137/-0.557 e Å^{-3}.

[$Ni^{I}(Mes_2Im)_2(2,3,5\text{-}C_6F_3H_2)$] (14)

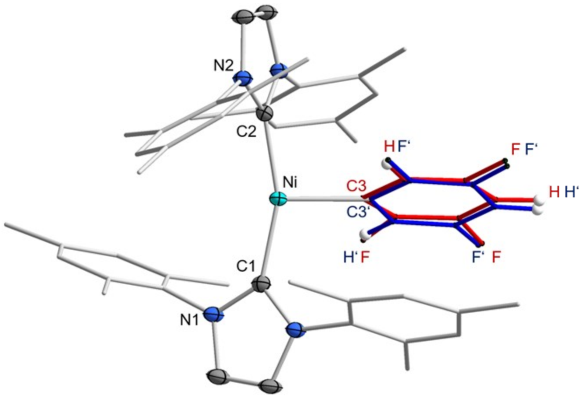

Figure S41: Molecular structure of [$Ni^{I}(Mes_2Im)_2(2,3,5\text{-}C_6F_3H_2)$] (**14**) in the solid state (ellipsoids set at 50 % probability level). Hydrogen atoms are omitted for clarity. Selected bond lengths [Å] and angles [°]: Ni–C1 1.918(1), Ni–C2 1.917(1), Ni–C3 1.869(1), Ni–C3' 2.046(1), C1-Ni-C2 159.5(5), C1-Ni-C3 101.6(4), C1-Ni-C3' 100.9(3), C2-Ni-C3 98.88(4), C2-Ni-C3' 99.58(3), NHC(C1):NHC(C2) 82.46(8), NHC(C1):C1-Ni-C3 59.61(3), NHC(C1):C1-Ni-C2-C3 60.81(7), NHC(C2):C2-Ni-C3 38.82(2), NHC(C2):C2-Ni-C1-C3 37.61(6).

Crystallographic data for **14**:

Formula: $C_{48}H_{50}F_3N_4Ni$, M_r = 798.63, red block, 0.120 x 0.131 x 0.310 mm, monoclinic space group $P2_1/c$, a = 13.8929(5) Å, b = 10.8012(4) Å, c = 27.4228(9) Å, α = 90 °, β = 91.771(2) °, γ = 90 °, V = 4113.1(3) Å^3, Z = 4, ρ_{calcd} = 1.290 g/cm^3, μ = 0.524 mm^{-1}, F(000) = 1684, T = 100(2) K, 85250 reflections measured in the range 2.389 ° < θ < 30.522 °, completeness 99.9 %, 12539 independent reflections, 10035 observed reflections [$I > 2\sigma(I)$], 587 parameters, 0 restraints; all data: R_1 = 0.0527 and wR_2 = 0.0957, $I > 2\sigma(I)$: R_1 = 0.0370 and wR_2 = 0.0883, Goof = 1.020, largest difference peak/hole 0.394/-0.401 e Å^{-3}

4) Computational Details

All quantum-chemical calculations were carried out with the Gaussian 16 program.[S6]

EPR parameters: The D3BJ[S7-S8] dispersion-corrected GGA functional PBE-D[S9] together with the def2-SVP basis set[S10] and the corresponding W06 auxiliary Coulomb fitting basis set[S11] were employed for geometry optimization and Hessian calculations (RI-PBE-D/def2-SVP/W06). EPR parameters were obtained from single-point calculations with the D3BJ dispersion-corrected hybrid-GGA functional PBE0-D[S12] in combination with the segmented polarization-consistent pcSseg-2 basis set of Jensen,[S13] which is specifically optimized for the calculation of magnetic properties (PBE0-D/pcSseg-2).

Mechanistic investigations: Geometry optimization and Hessian calculations were performed at the PBE0-D/def2-SVP level of DFT. Zero-point vibrational energies and thermal contributions to Gibbs free energies at 298.15 K were obtained at this level using standard procedures implemented in the Gaussian program. Relative energies were computed at the PBE0-D level with the split-valence triple-zeta orbital basis set def2-TZVP[S10] and the implicit solvent model COSMO (solvent: tetrahydrofuran)[S14-S15] (COSMO(thf)-PBE0-D/def2-TZVP). DFT energies for broken-symmetry wave functions were corrected according to Yamaguchi's spin-projection formula:[S16-S17]

$$E_S = E_T - \frac{2\,(E_T - E_{BS})}{\langle S^2 \rangle_T - \langle S^2 \rangle_{BS}}$$

5) NMR-Spectra

[Ni(Mes$_2$Im)$_2$F(4-CF$_3$-C$_6$F$_4$)] (2)

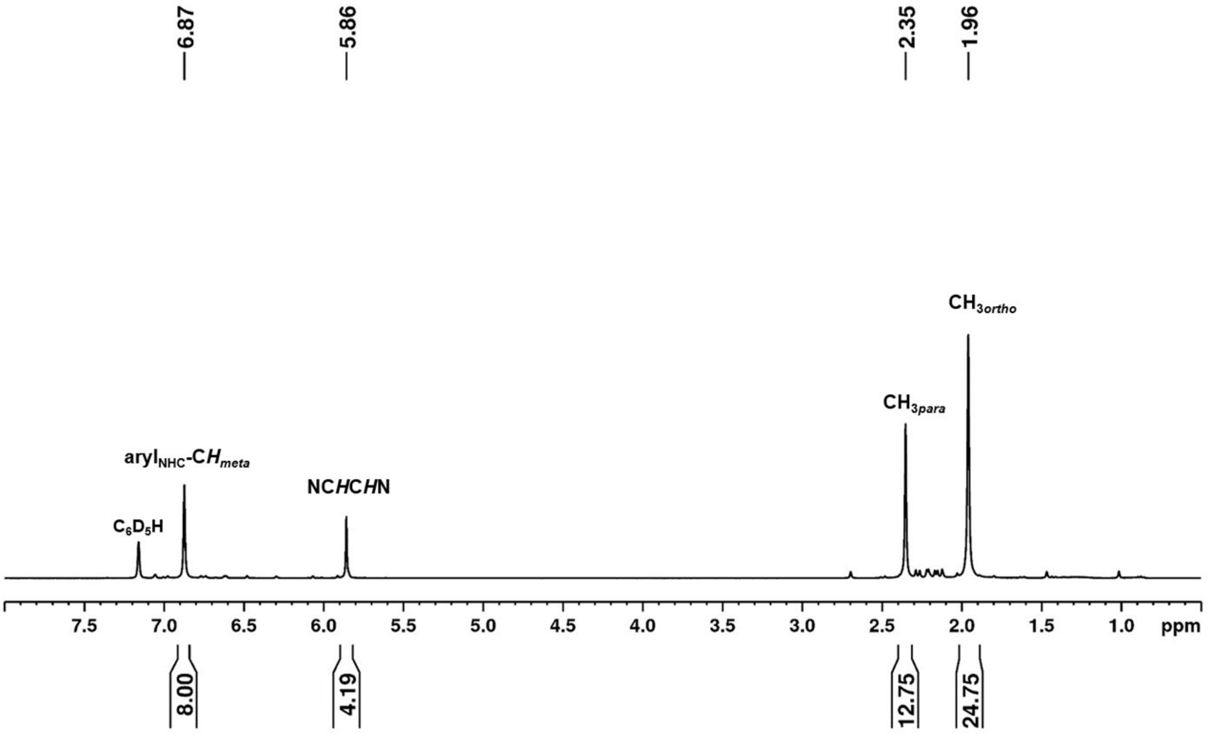

Figure S42: ^{1}H NMR (400.4 MHz) spectrum of [Ni(Mes$_2$Im)$_2$F(4-CF$_3$-C$_6$F$_4$)] (**2**) in C$_6$D$_6$.

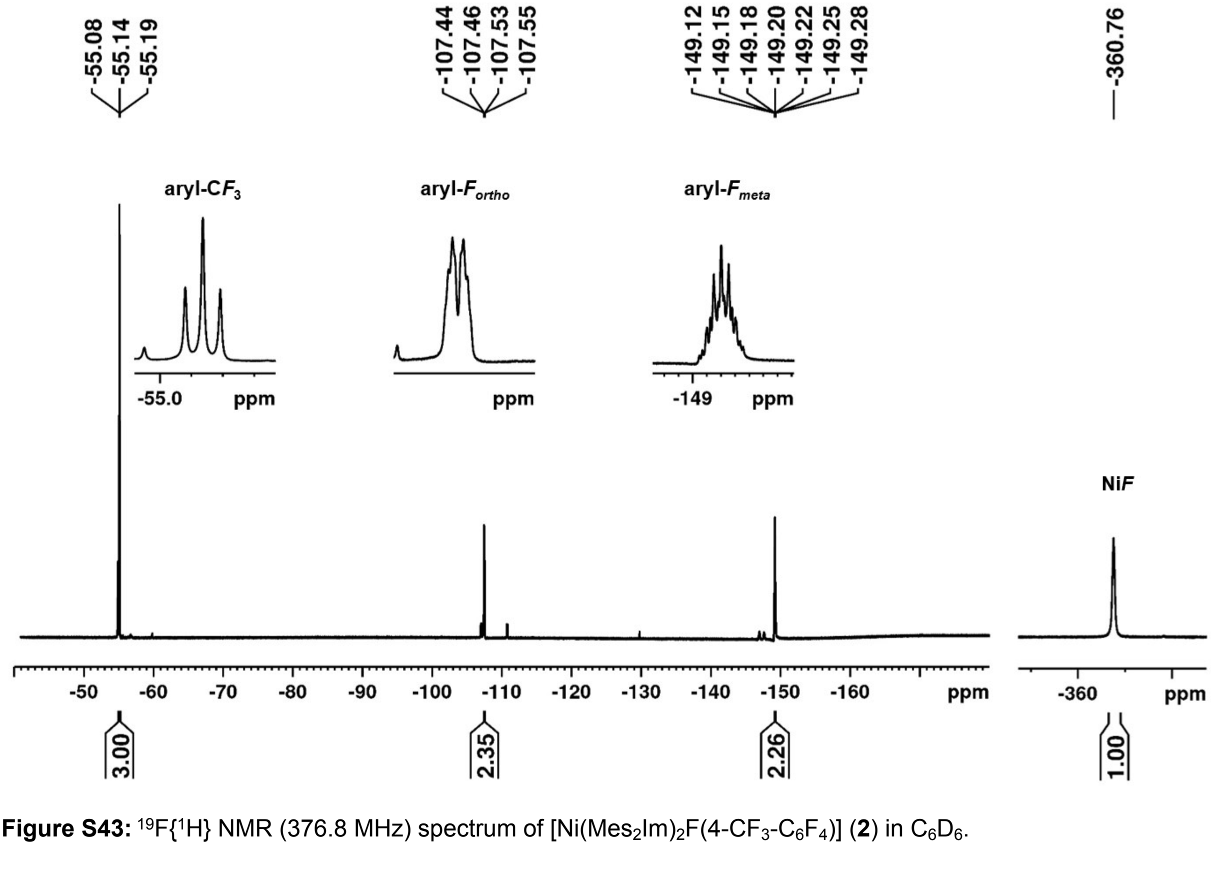

Figure S43: ^{19}F{^{1}H} NMR (376.8 MHz) spectrum of [Ni(Mes$_2$Im)$_2$F(4-CF$_3$-C$_6$F$_4$)] (**2**) in C$_6$D$_6$.

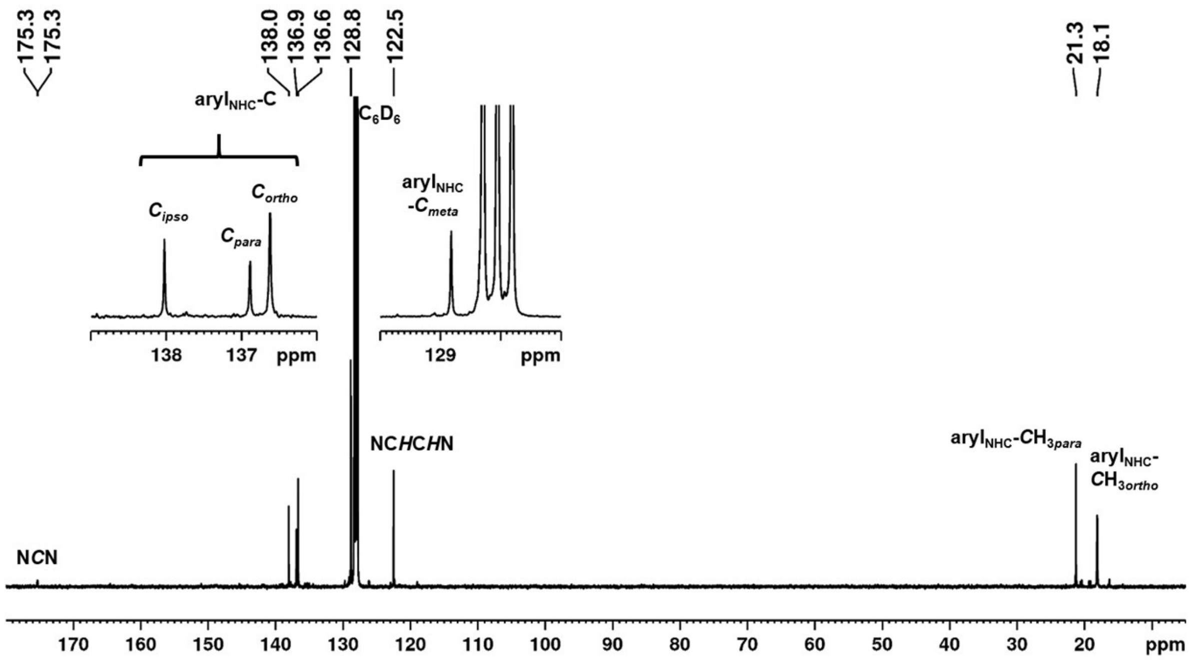

Figure S44: $^{13}C\{^{1}H\}$ NMR (100.7 MHz) spectrum of $[Ni(Mes_2Im)_2F(4\text{-}CF_3\text{-}C_6F_4)]$ **2** in C_6D_6.

$[Ni(Mes_2Im)_2F(C_6F_5)]$ (3)

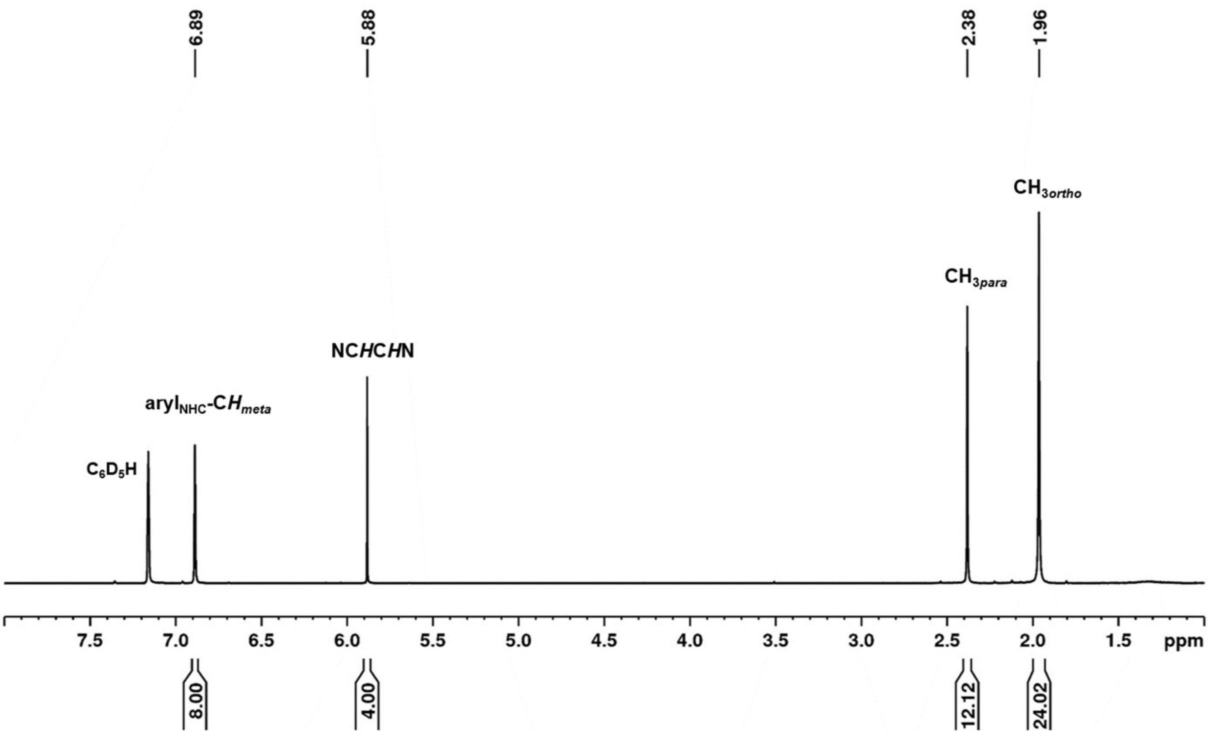

Figure S45: ^{1}H NMR (500.1 MHz) spectrum of $[Ni(Mes_2Im)_2F(C_6F_5)]$ (**3**) in C_6D_6.

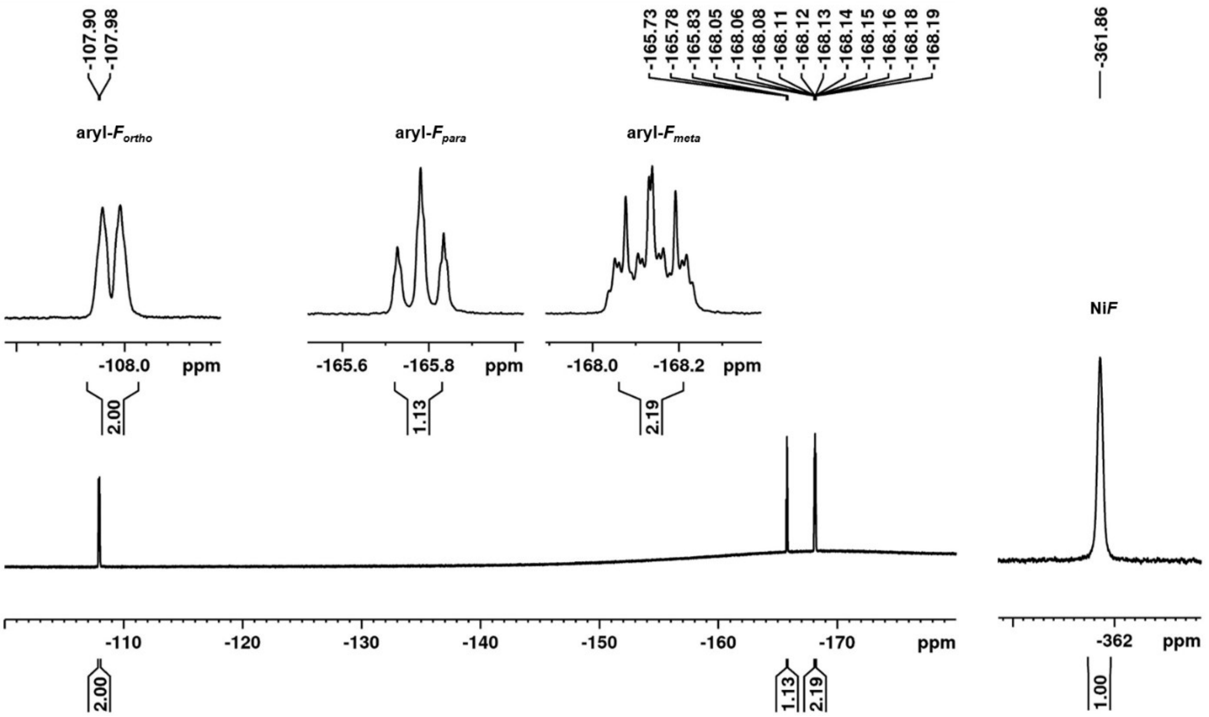

Figure S46: $^{19}F\{^1H\}$ NMR (470.6 MHz) spectrum of $[Ni(Mes_2Im)_2F(C_6F_5)]$ (**3**) in C_6D_6.

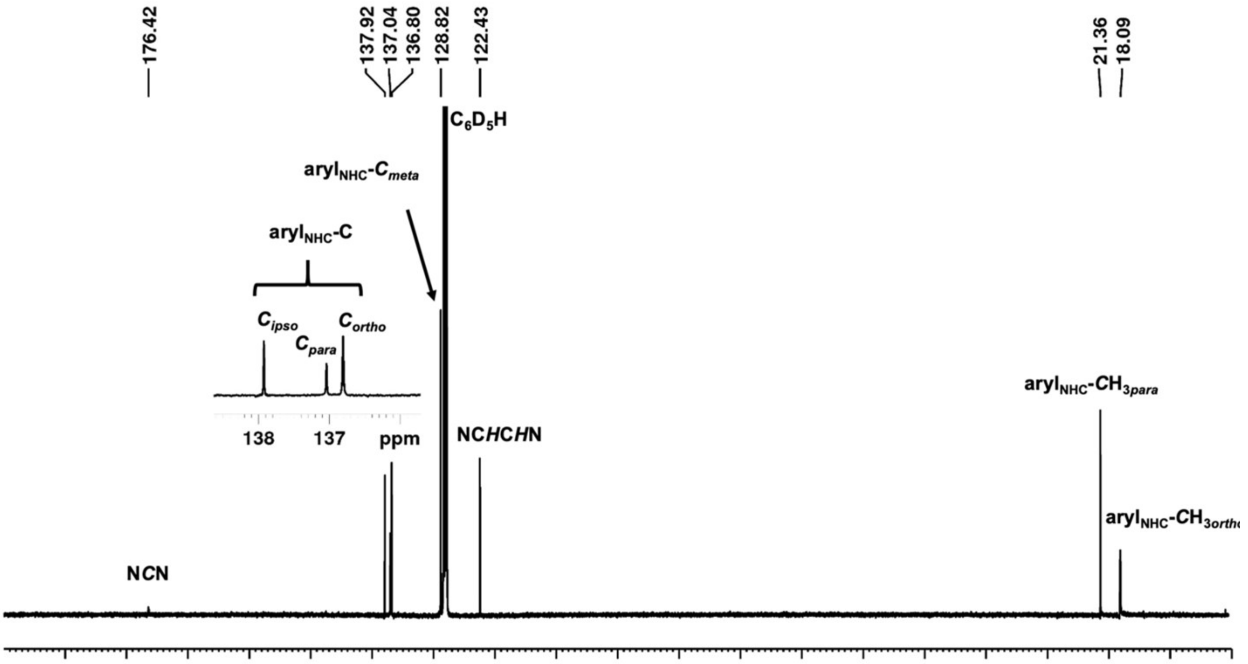

Figure S47: $^{13}C\{^1H\}$ NMR (125.8 MHz) spectrum of $[Ni(Mes_2Im)_2F(C_6F_5)]$ (**3**) in C_6D_6.

[Ni(Mes$_2$Im)$_2$F(2,3,5,6-C$_5$NF$_4$H)] (4)

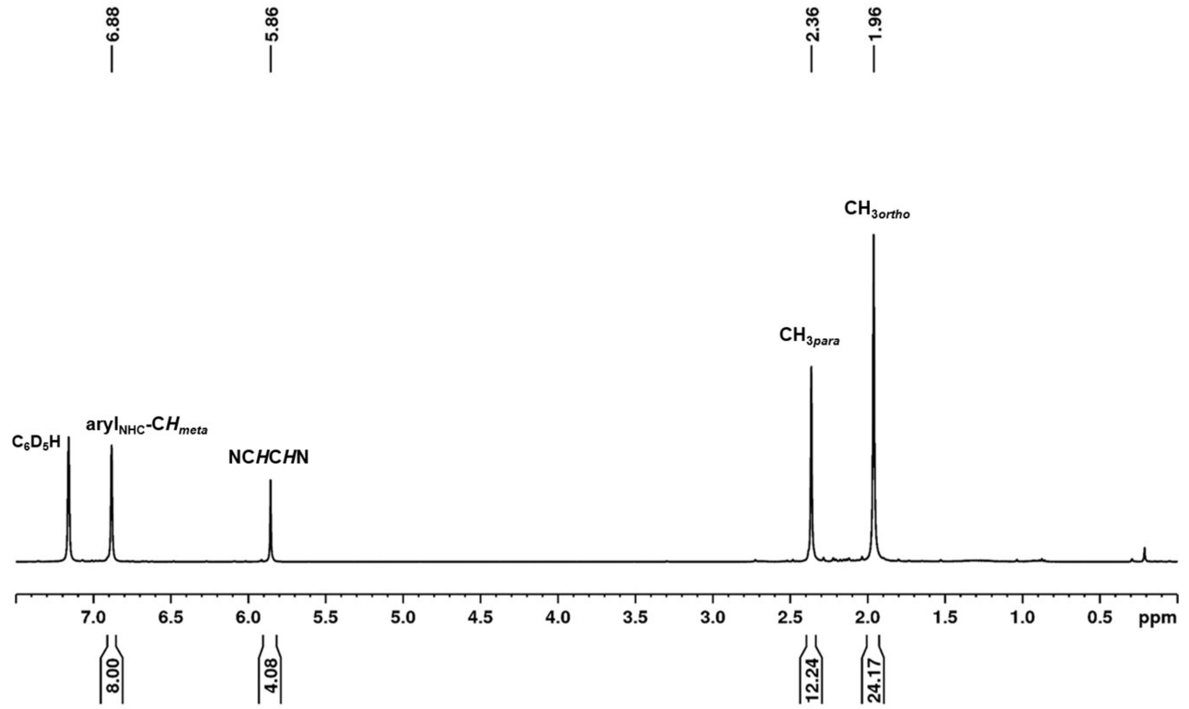

Figure S48: ^{1}H NMR (400.4 MHz) spectrum of [Ni(Mes$_2$Im)$_2$F(C$_5$NF$_4$H)] (**4**) in C$_6$D$_6$.

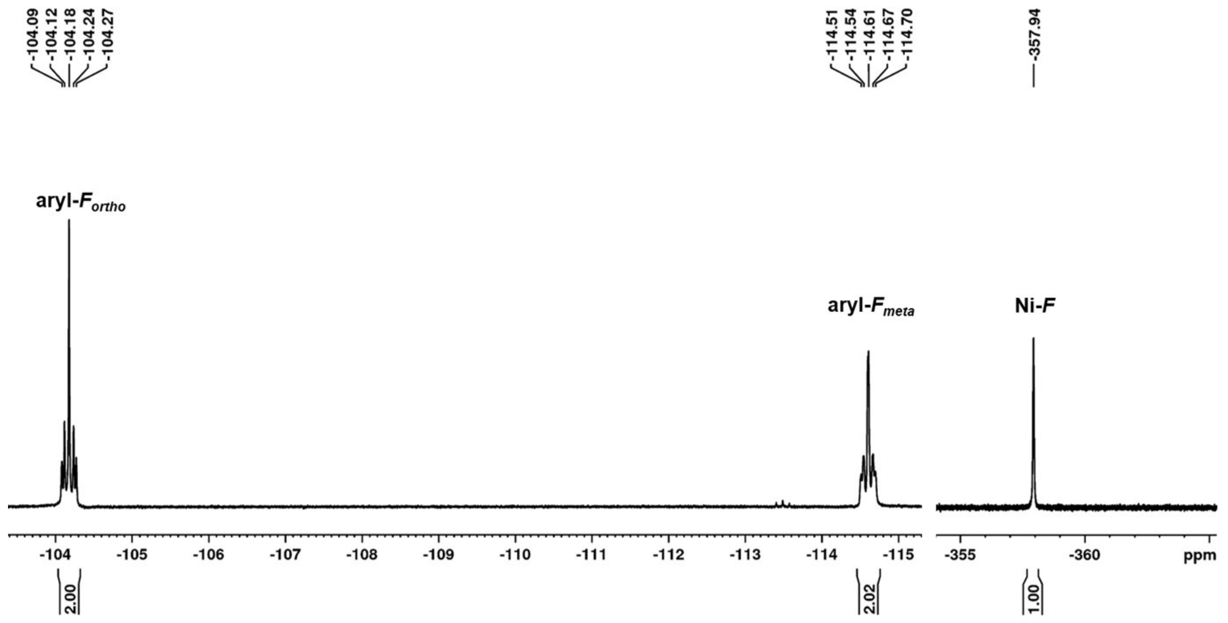

Figure S49: ^{19}F{^{1}H} NMR (376.8 MHz) spectrum of [Ni(Mes$_2$Im)$_2$F(C$_5$NF$_4$H)] (**4**) in C$_6$D$_6$.

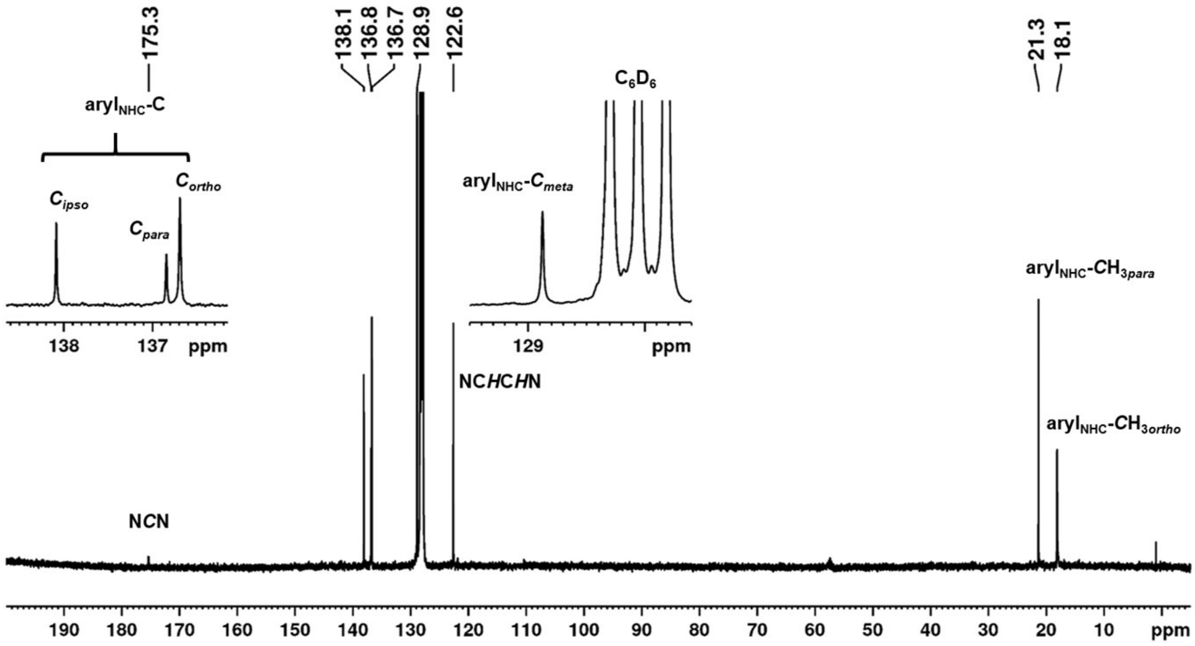

Figure S50: $^{13}C\{^1H\}$ NMR (100.7 MHz) spectrum of [Ni(Mes$_2$Im)$_2$F(C_5NF_4H)] (**4**) in C_6D_6.

[Ni(Mes$_2$Im)$_2$F(2,3,5,6-C_6F_4H)] (5)

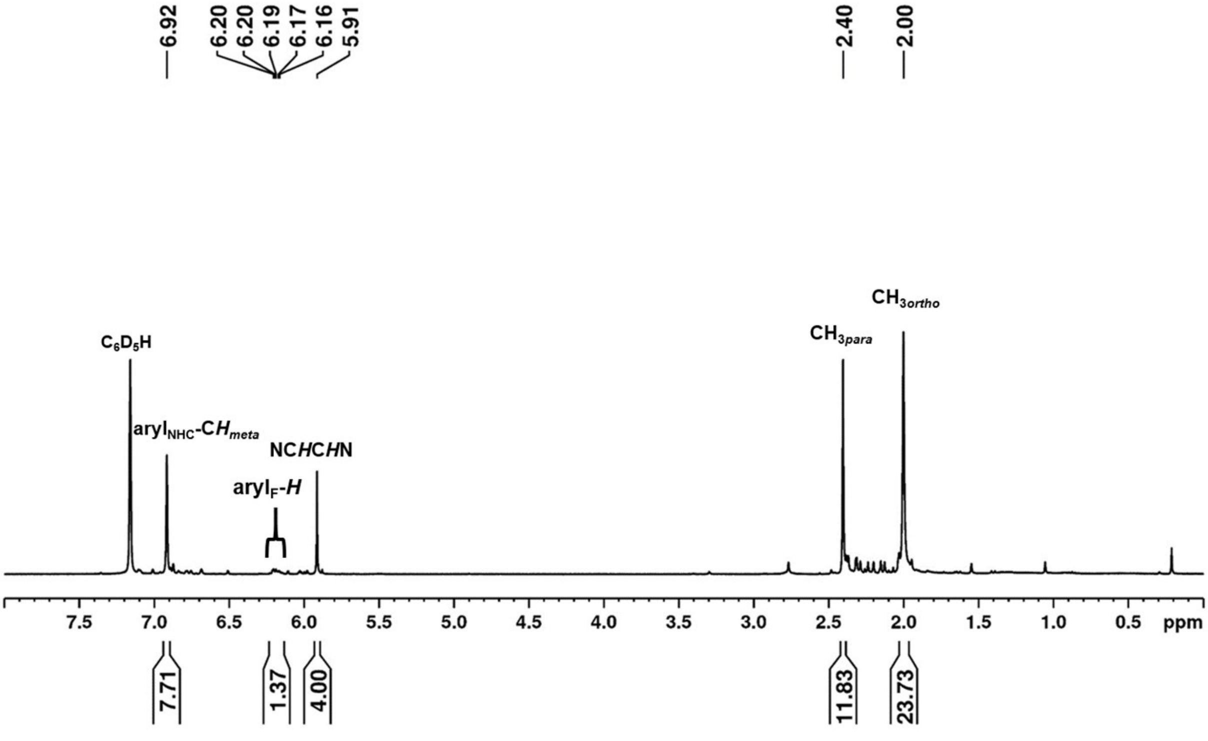

Figure S51: ^1H NMR (400.4 MHz) spectrum of [Ni(Mes$_2$Im)$_2$F(2,3,5,6-C_6F_4H)] (**5**) in C_6D_6.

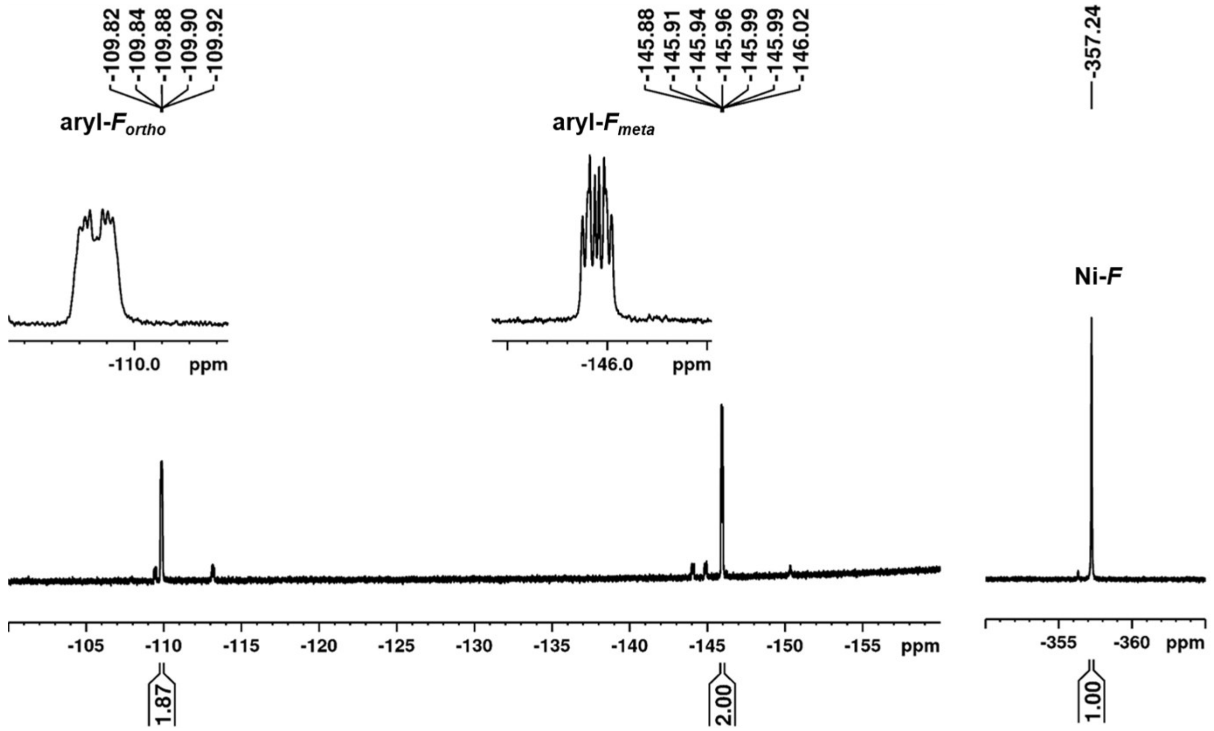

Figure S52: $^{19}F\{^1H\}$ NMR (376.8 MHz) spectrum of [Ni(Mes$_2$Im)$_2$F(2,3,5,6-C_6F_4H)] (**5**) in C_6D_6.

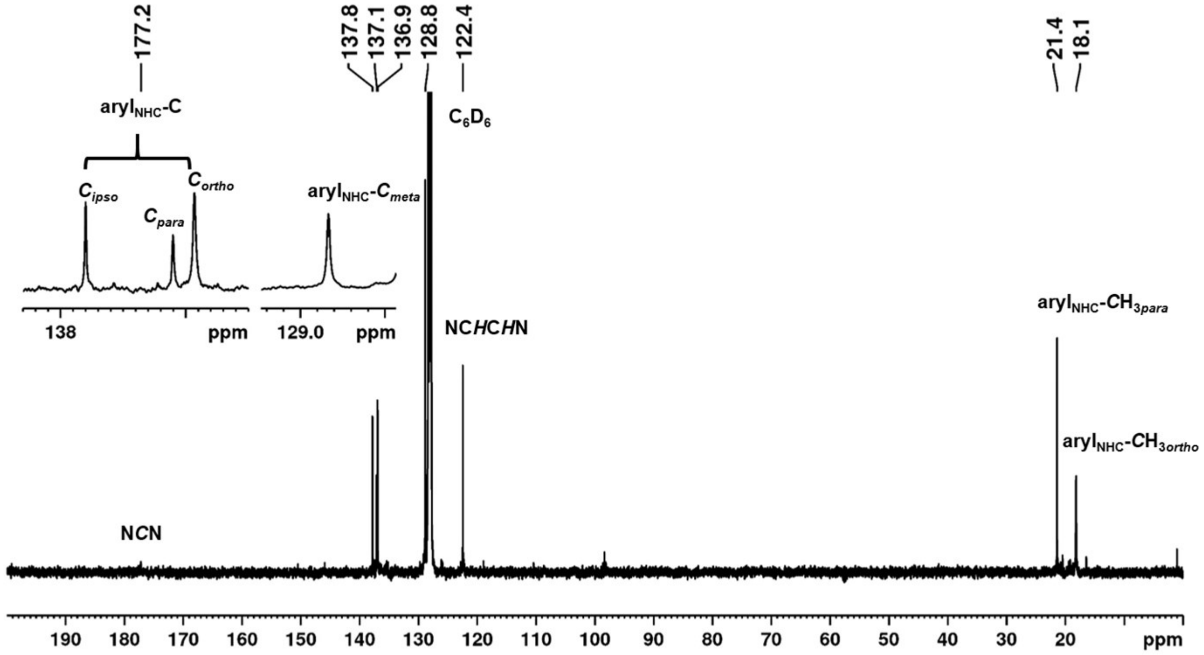

Figure 53: $^{13}C\{^1H\}$ NMR (100.7 MHz) spectrum of [Ni(Mes$_2$Im)$_2$F(2,3,5,6-C_6F_4H)] (**5**) in C_6D_6.

[Ni(Mes$_2$Im)$_2$F(2,3,5-C$_6$F$_3$H$_2$)] (6)

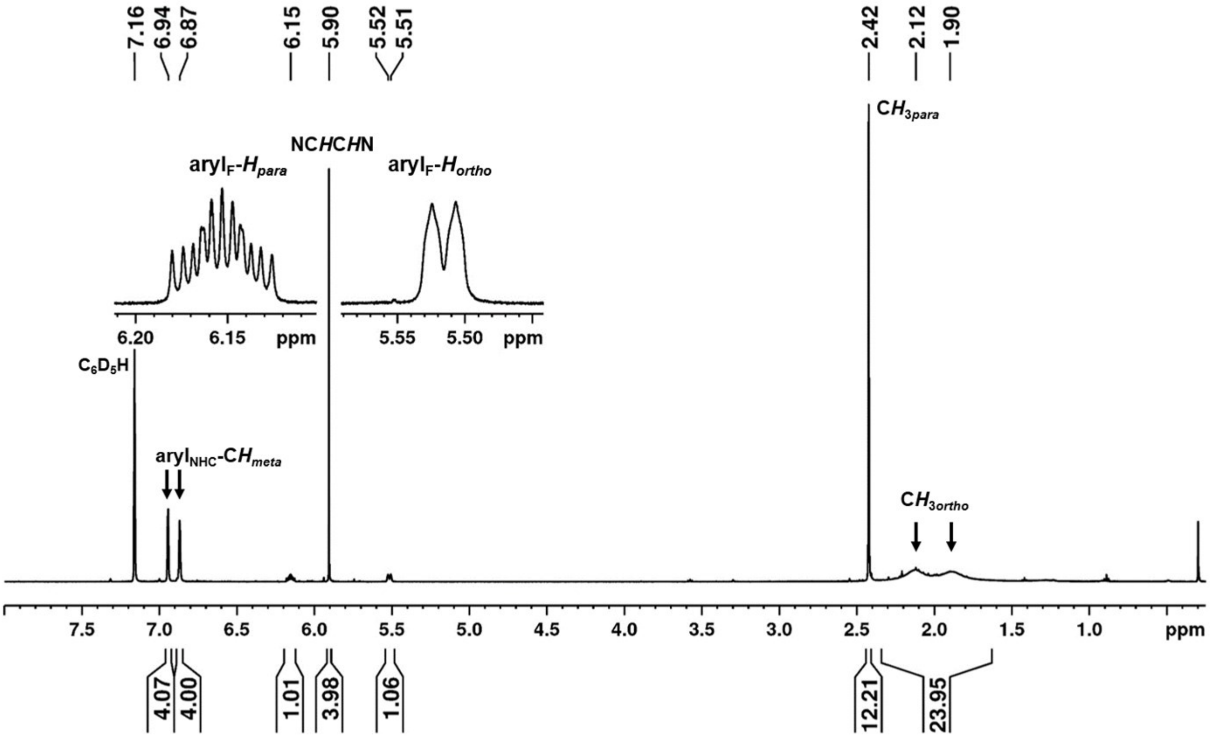

Figure S54: ^{1}H NMR (500.1 MHz) spectrum of [Ni(Mes$_2$Im)$_2$F(2,3,5-C$_6$F$_3$H$_2$)] (**6**) in C$_6$D$_6$.

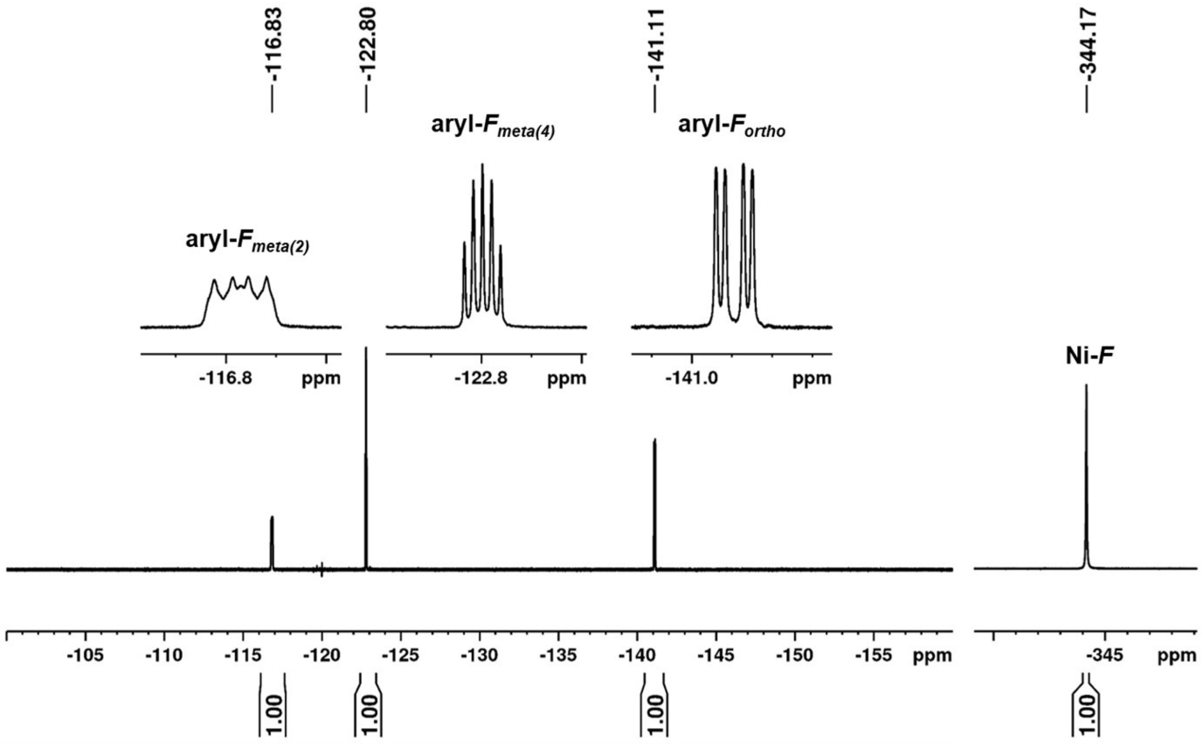

Figure S55: ^{19}F{^{1}H} NMR (470.6 MHz) spectrum of [Ni(Mes$_2$Im)$_2$F(2,3,5-C$_6$F$_3$H$_2$)] (**6**) in C$_6$D$_6$.

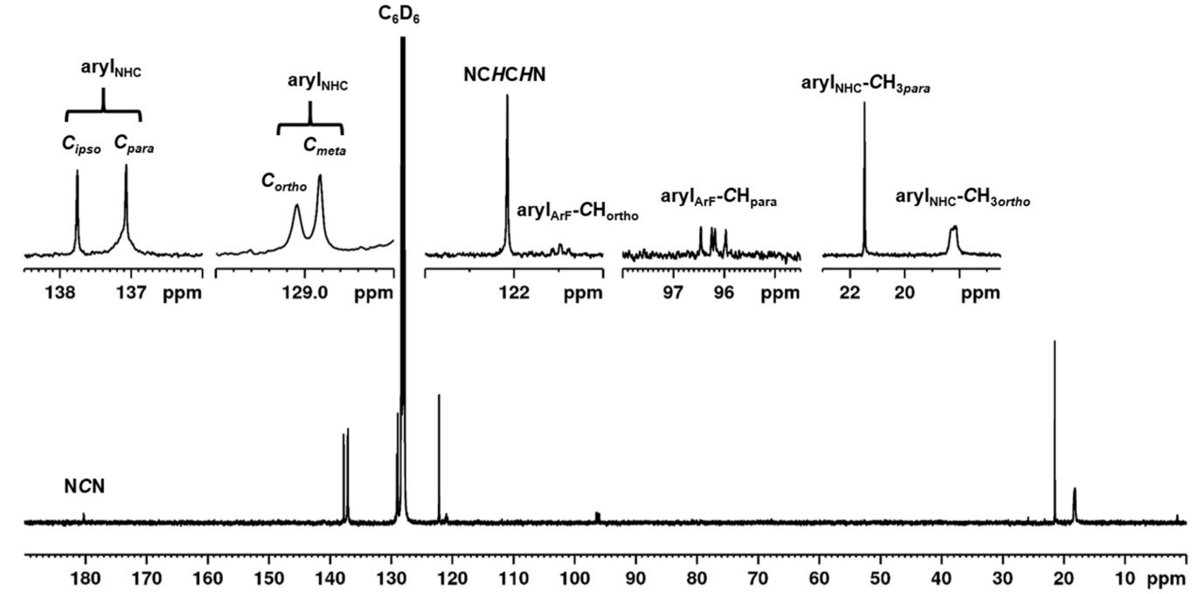

Figure S56: $^{13}C\{^{1}H\}$ NMR (100.7 MHz) spectrum of [Ni(Mes$_2$Im)$_2$F(2,3,5-$C_6F_3H_2$)] (**6**) in C_6D_6.

[Ni(Mes$_2$Im)$_2$F(3,5-$C_6F_2H_3$)] (7)

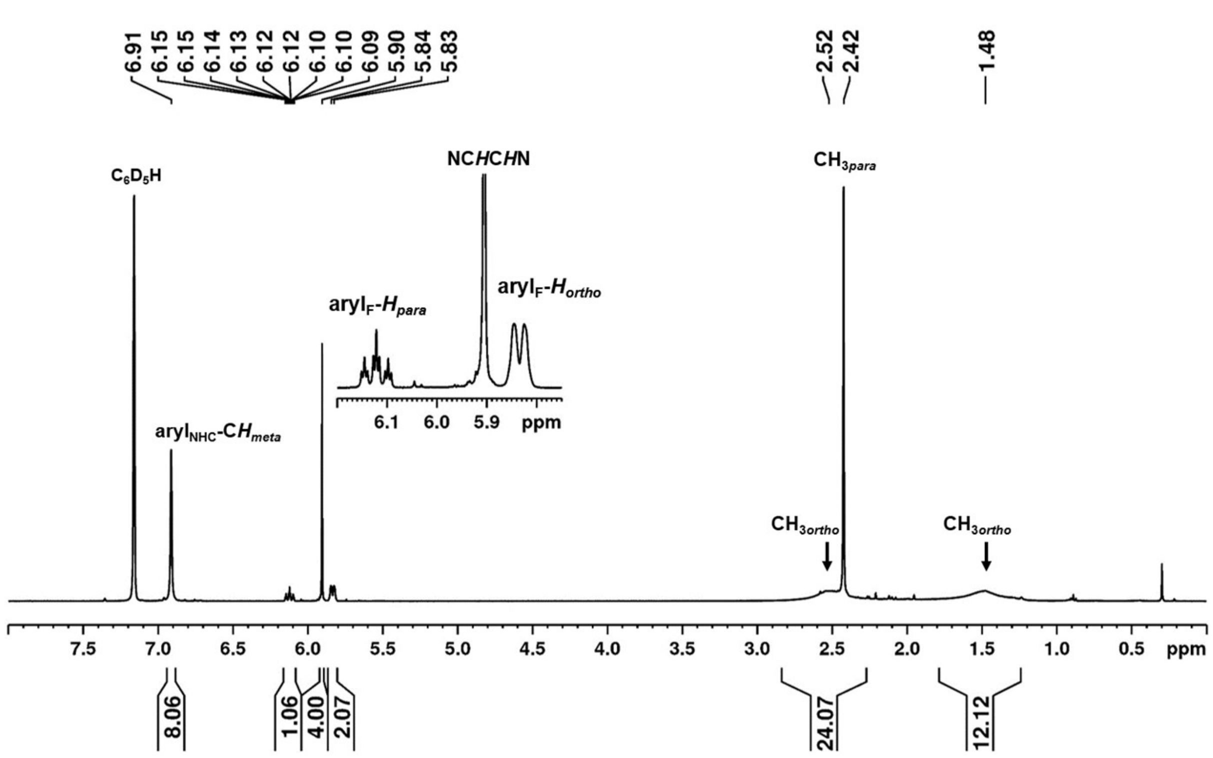

Figure S57: ^{1}H NMR (400.4 MHz) spectrum of [Ni(Mes$_2$Im)$_2$F(3,5-$C_6F_2H_3$)] (**7**) in C_6D_6.

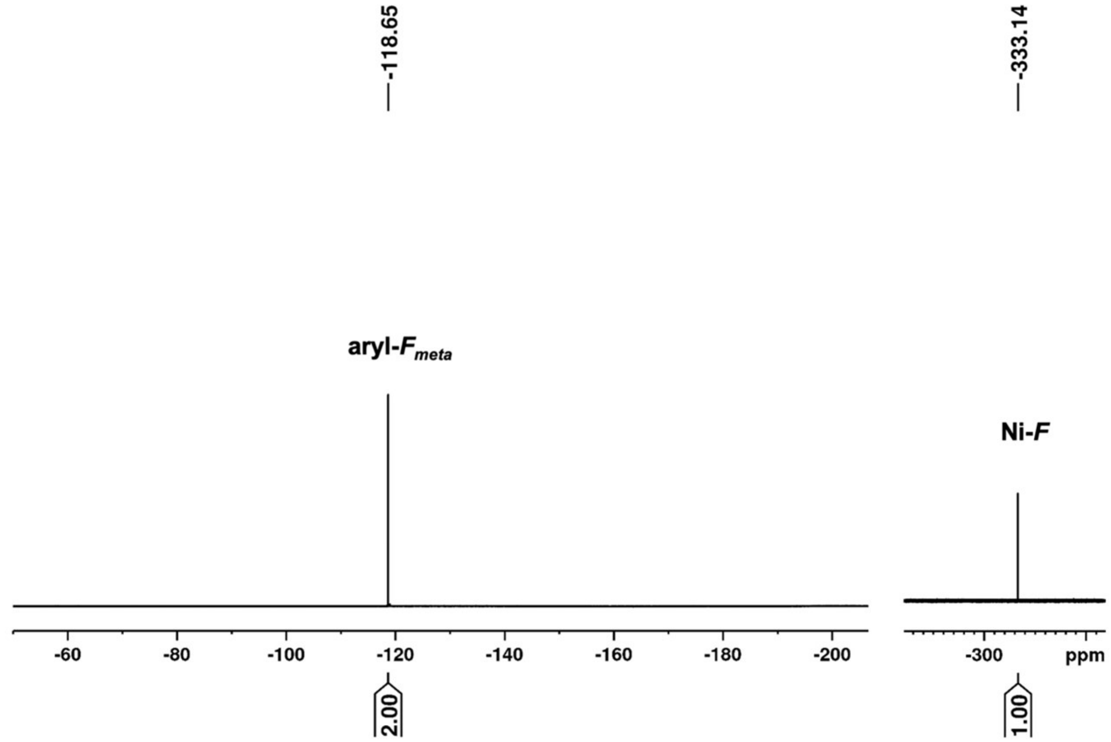

Figure S58: $^{19}F\{^1H\}$ NMR (376.8 MHz) spectrum of [Ni(Mes$_2$Im)$_2$F(C$_6$F$_2$H$_3$)] (**7**) in C_6D_6.

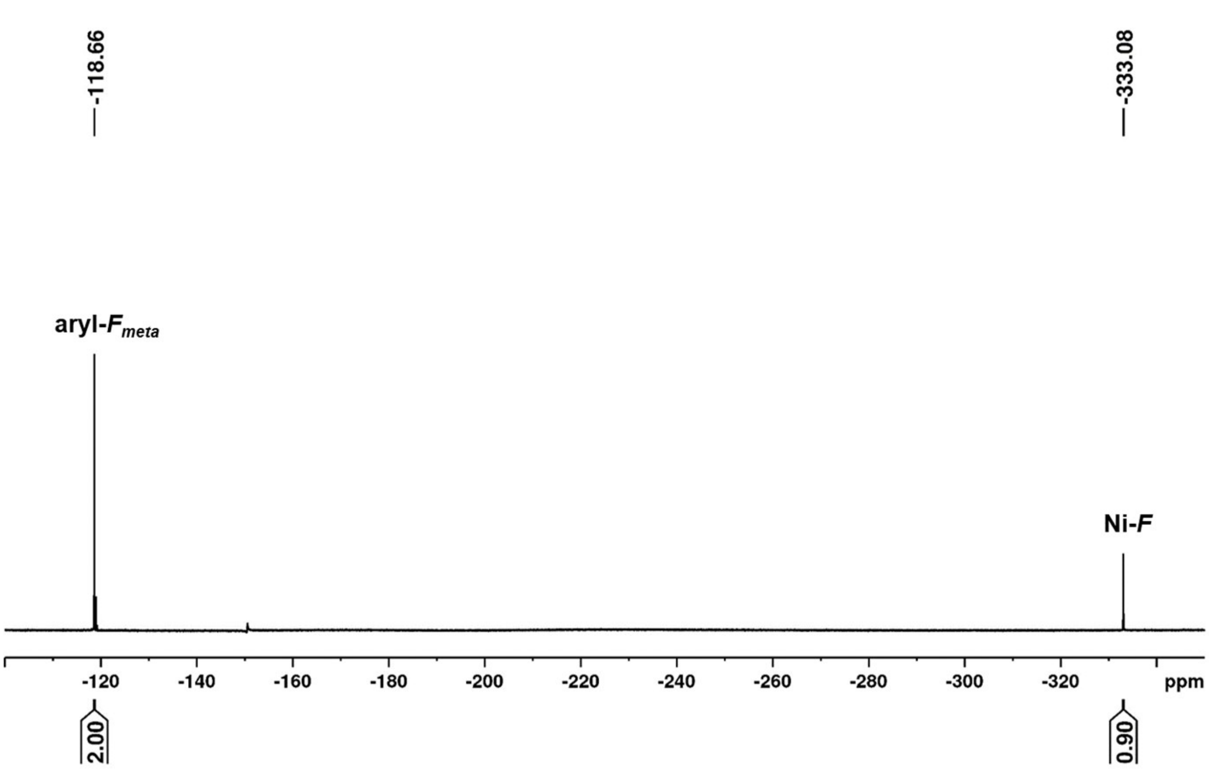

Figure S59: ^{19}F NMR (188.1 MHz) spectrum of [Ni(Mes$_2$Im)$_2$F(C$_6$F$_2$H$_3$)] (**7**) in C_6D_6.

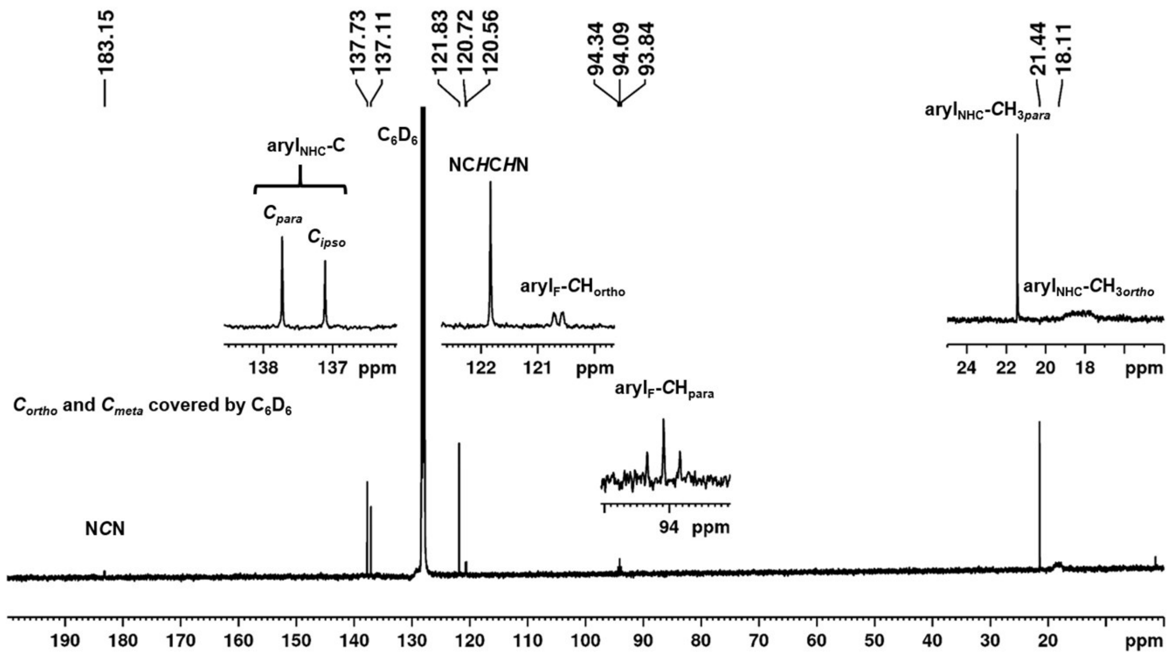

Figure S60: $^{13}C\{^1H\}$ NMR (100.7 MHz) spectrum of [Ni(Mes$_2$Im)$_2$F(3,5-$C_6F_2H_3$)] (**7**) in C_6D_6.

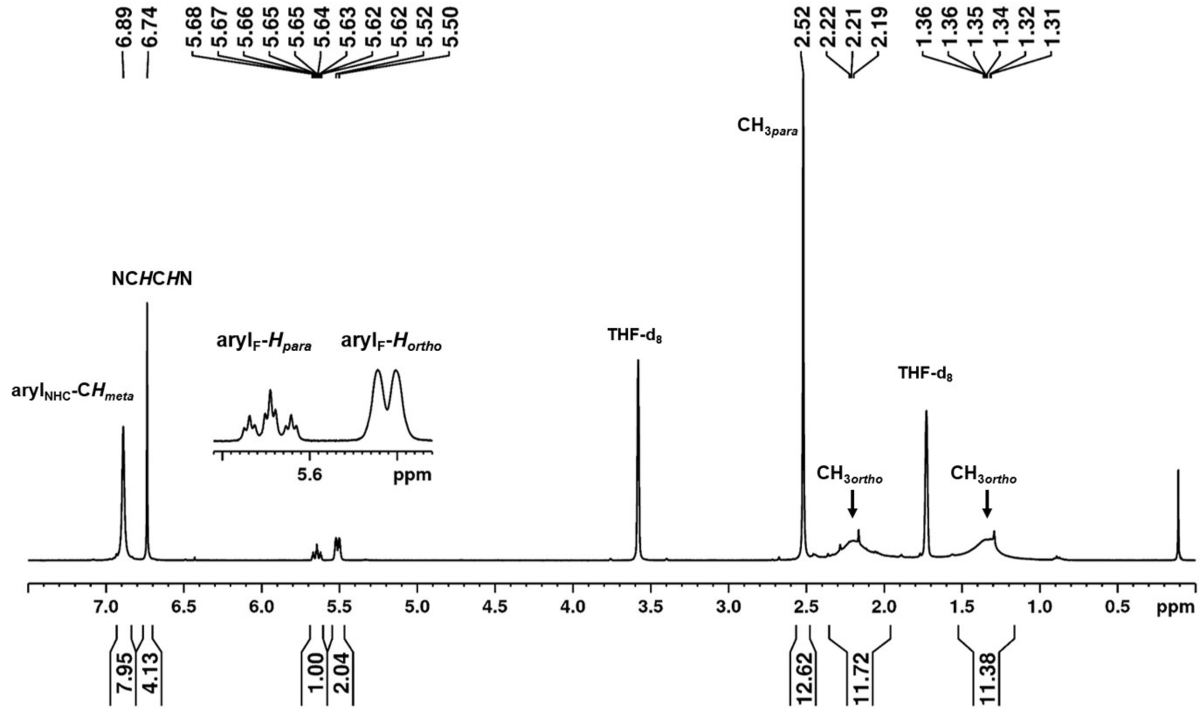

Figure S61: ^1H NMR (400.4 MHz) spectrum of [Ni(Mes$_2$Im)$_2$F(3,5-$C_6F_2H_3$)] (**7**) in thf-d_8.

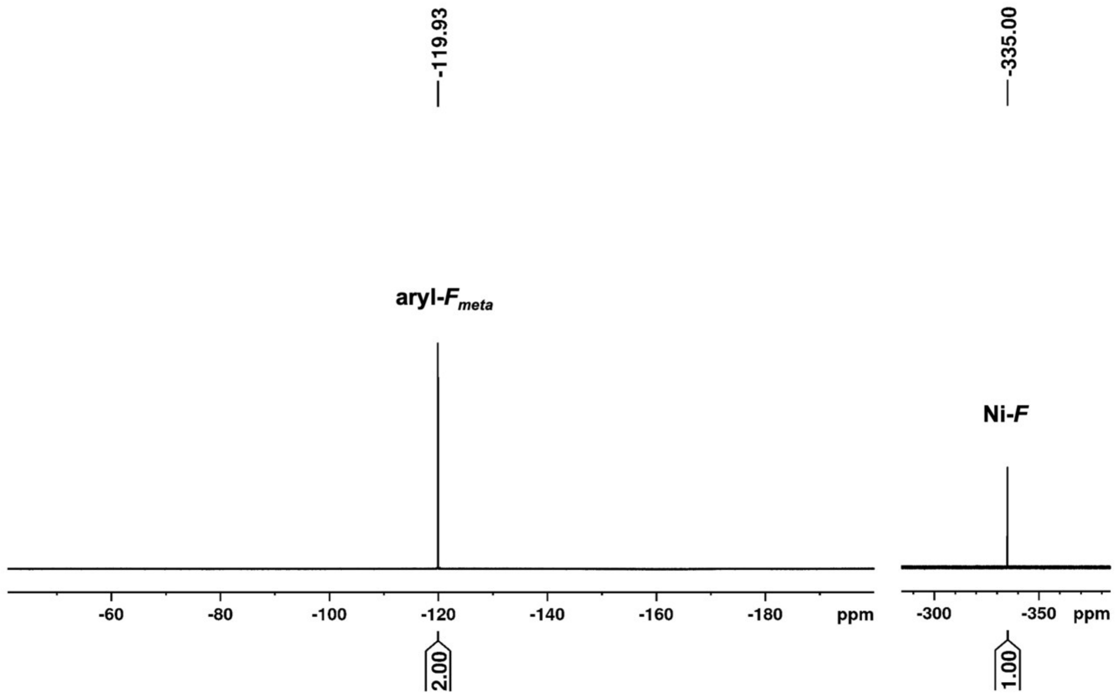

Figure S62: $^{19}F\{^{1}H\}$ NMR (376.8 MHz) spectrum of [Ni(Mes$_2$Im)$_2$F(C$_6$F$_2$H$_3$)] (**7**) in thf-d$_8$.

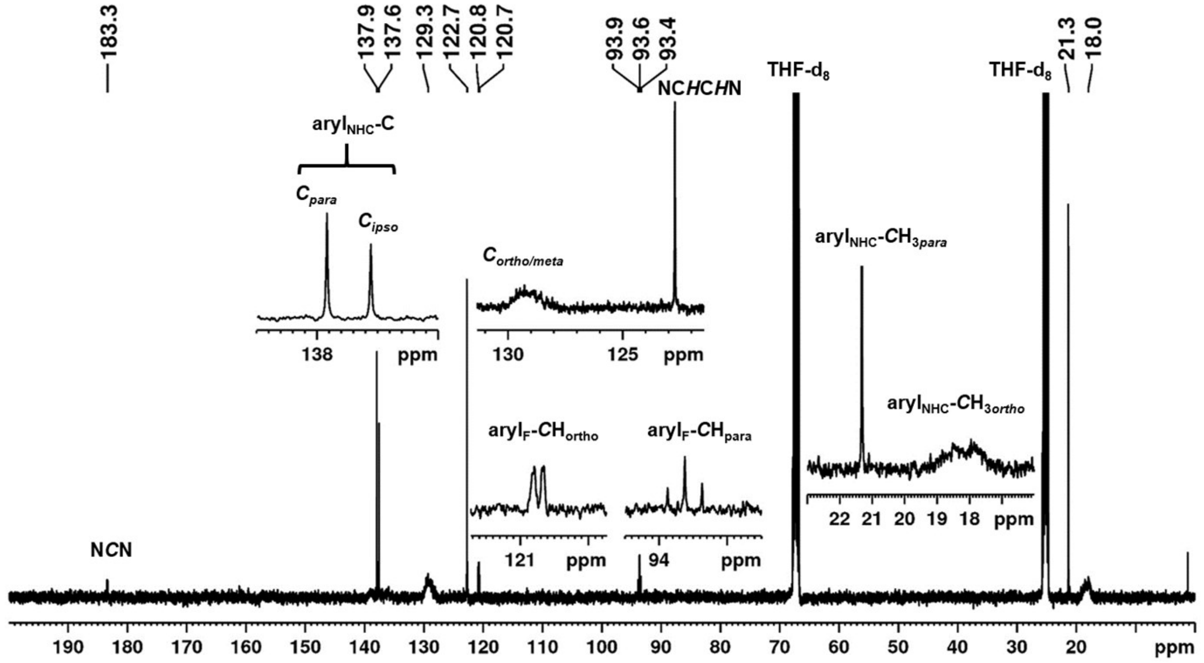

Figure S63: $^{13}C\{^{1}H\}$ NMR (100.7 MHz) spectrum of [Ni(Mes$_2$Im)$_2$F(3,5-C$_6$F$_2$H$_3$)] (**7**) in thf-d$_8$.

$[Ni(Mes_2Im)_2][BF_4]$ (8)

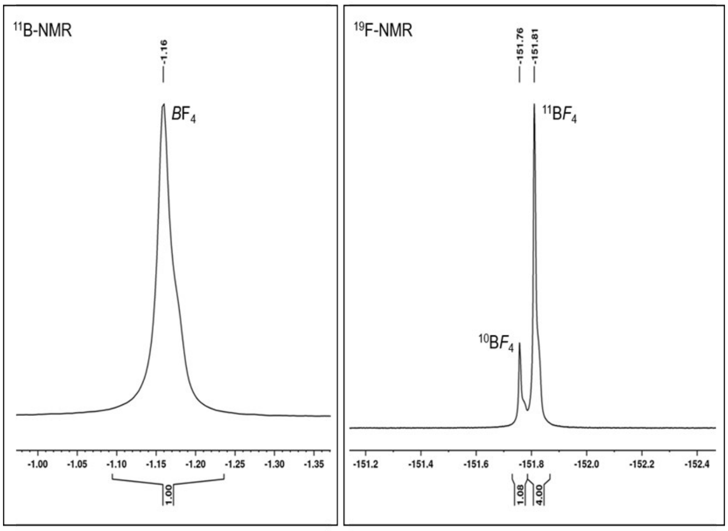

Figure S64: $^{11}B\{^1H\}$ NMR (128.5 MHz) spectrum (left) and $^{19}F\{^1H\}$ NMR (376.8 MHz) spectrum (right) of $[Ni(Mes_2Im)_2][BF_4]$ (**8**) in CD_3CN.

$[Ni(Mes_2Im)_2F_2]$ (9)

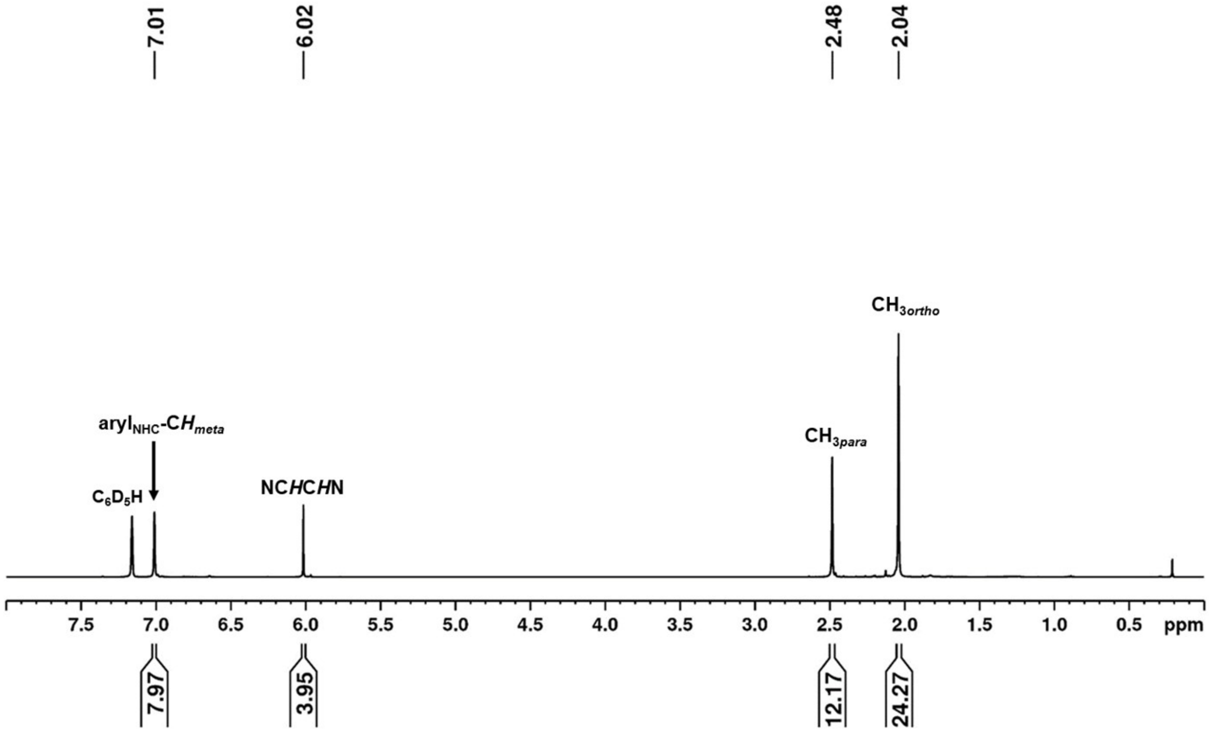

Figure S65: ^1H NMR (400.4 MHz) spectrum of $[Ni(Mes_2Im)_2F_2]$ (**9**) in C_6D_6.

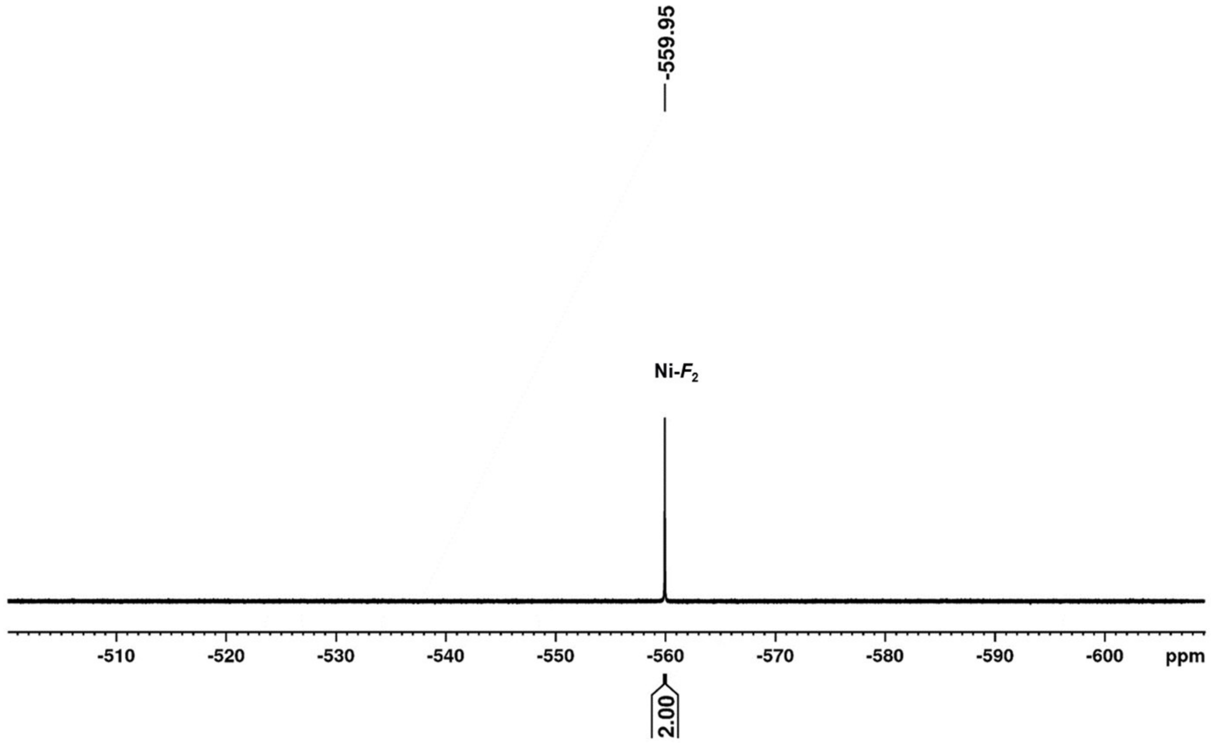

Figure S66: $^{19}F\{^1H\}$ NMR (376.8 MHz) spectrum of [Ni(Mes$_2$Im)$_2$F$_2$] (**9**) in C_6D_6.

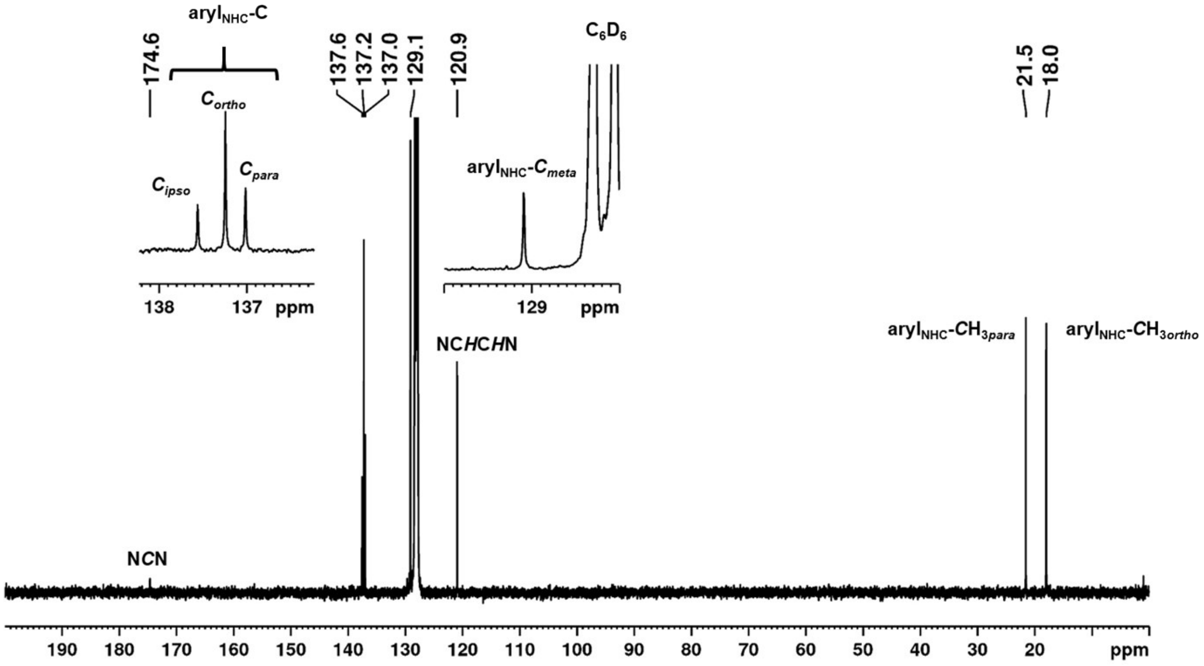

Figure S67: $^{13}C\{^1H\}$ NMR (100.7 MHz) spectrum of [Ni(Mes$_2$Im)$_2$F$_2$] (**9**) in C_6D_6.

[Ni(Mes$_2$Im)$_2$I$_2$] (10)

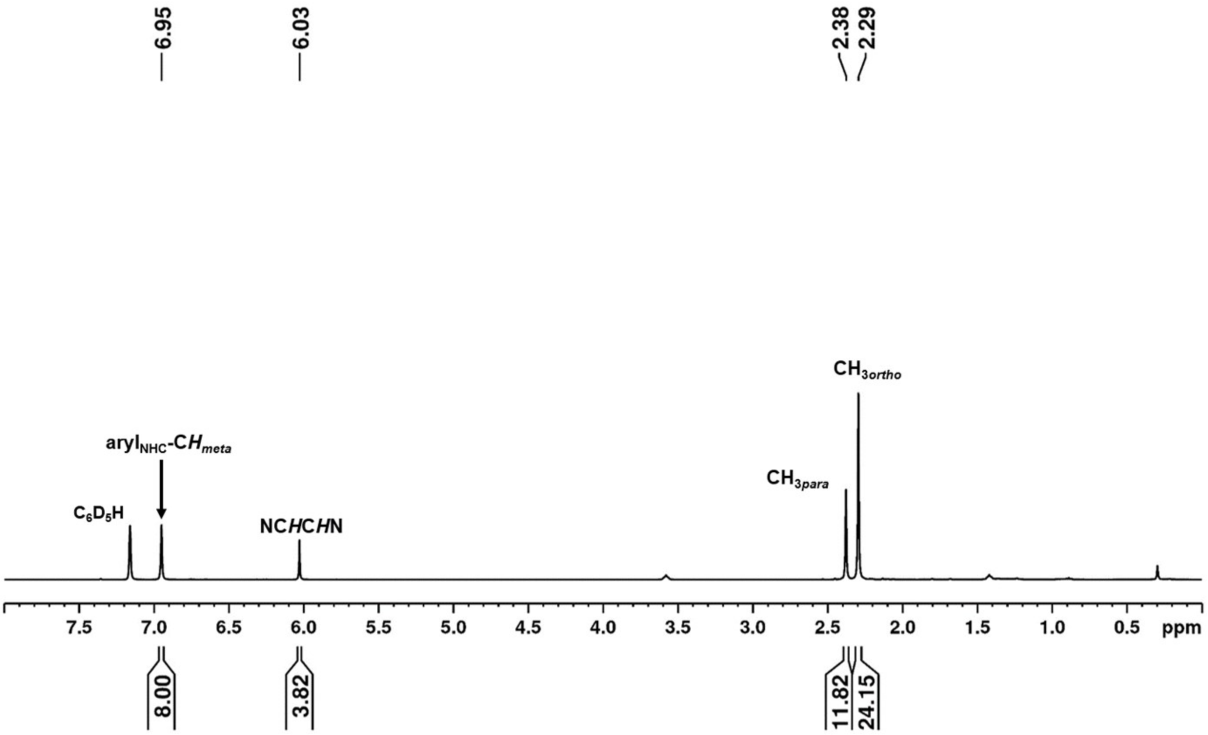

Figure S68: ^{1}H NMR (400.4 MHz) spectrum of [Ni(Mes$_2$Im)$_2$(I)$_2$] (**10**) in C_6D_6.

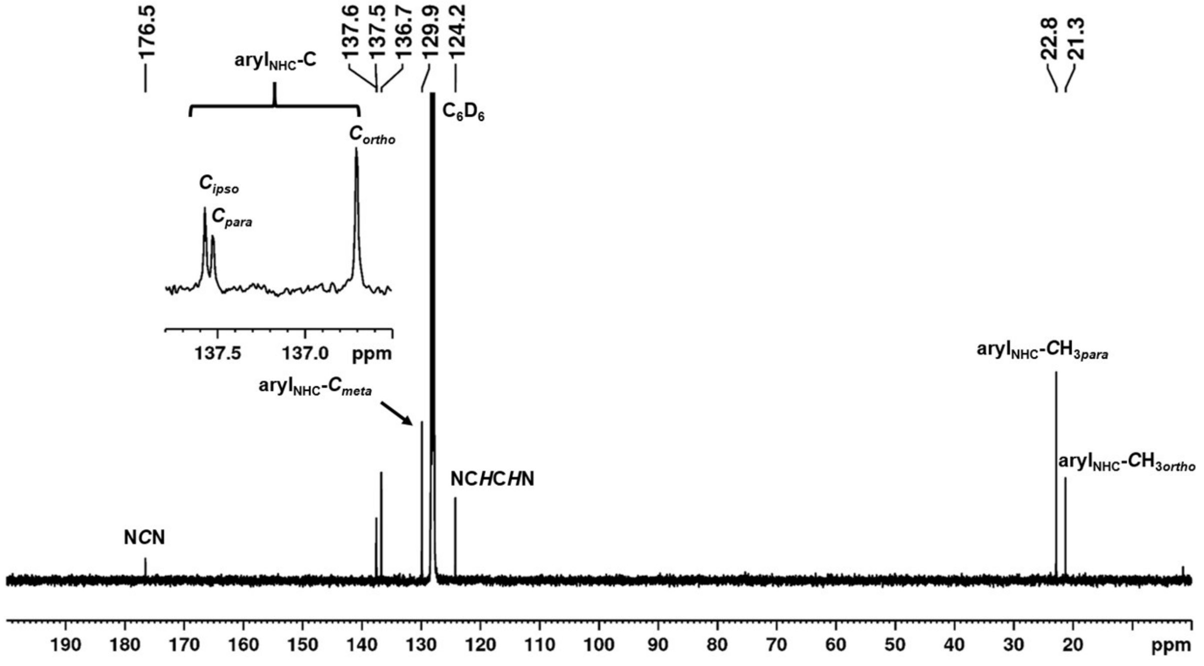

Figure S69: $^{13}C\{^1H\}$ NMR (100.7 MHz) spectrum of [Ni(Mes$_2$Im)$_2$(I)$_2$] (**10**) in C_6D_6.

6) Cartesian Coordinates

Cartesian coordinates are given in Å and energies in H.

EPR calculations:

106

11_opt ! 2-A (C2): E(UPBE-D3(BJ)/def2SVP) = -4079.39117829

E(UPBE0-D3BJ/pcSseg-2) = -4082.52860704

Ni	0.000000000000	0.000000000000	0.419689554739
N	-0.000000000000	2.940470319303	-0.037324637296
N	-1.332726330706	2.362750156753	1.562442292633
C	-0.422284350895	1.832214448829	0.674591496582
C	-0.629594213327	4.104373850981	0.397900981126
H	-0.411427629952	5.081107947635	-0.042273369983
C	-1.475080198222	3.741507993909	1.410904121205
H	-2.148739074574	4.331312670662	2.039090577696
C	-1.912228399914	1.600490103739	2.629802339156
C	-1.176461682685	1.485763048077	3.831664325367
C	-1.757331164387	0.765784928851	4.888233127182
H	-1.199146937507	0.669989543048	5.833506819640
C	-3.020915816195	0.156436943462	4.765243285596
C	-3.721699734311	0.305443696772	3.553941119280
H	-4.716316162708	-0.156251928987	3.445010354222
C	-3.195393341670	1.035723693218	2.472404577637
C	0.195293855896	2.094872987546	3.941362984408
H	0.196714743378	3.162082216639	3.639023908650
H	0.900488786158	1.568143842392	3.263872720275
H	0.588212380700	2.017952352772	4.971767012117
C	-3.587514838191	-0.668235174564	5.893300355540
H	-3.326083379860	-0.243120965759	6.882878048318
H	-3.181281046709	-1.703113721593	5.867683588486
H	-4.690238526309	-0.749057390537	5.830650080483
C	-3.959331266266	1.214288232829	1.187964465759
H	-4.906378136546	0.642661783030	1.204998043049
H	-3.361277340932	0.886255737927	0.315375415550

H	-4.208345566736	2.282067523651	1.012672360057
C	0.963555818326	2.887627485705	-1.098677752141
C	2.282931276556	2.479213695665	-0.800009318161
C	3.187284809824	2.351385031902	-1.868201226989
H	4.211474913595	2.007486179894	-1.653107503574
C	2.813639810280	2.614872479099	-3.197322338374
C	1.499044721250	3.051976899956	-3.444736793209
H	1.183866859213	3.252504737632	-4.481363936162
C	0.552547093395	3.194531970185	-2.416657762655
C	2.690595998200	2.129433122825	0.604952941377
H	3.783235583826	1.975044690320	0.680128492615
H	2.174147368100	1.195498558545	0.928661691285
H	2.389071901887	2.911421949213	1.331507220430
C	3.766274014167	2.364345027184	-4.337993631359
H	3.621358767449	3.089297155667	-5.163445867786
H	3.591698423791	1.349208076231	-4.753619130294
H	4.823221391413	2.413036028329	-4.010472616975
C	-0.862058682044	3.612849897163	-2.730595594077
H	-1.041124364708	4.684084793487	-2.496992398720
H	-1.600006666156	3.024467887738	-2.152222654997
H	-1.081719347936	3.466380002826	-3.805099824628
C	-0.000000000000	-0.000000000000	-1.522624249792
C	-1.123195500625	0.343448028244	-2.277428377015
C	-1.152850881855	0.362953499064	-3.677442143959
C	-0.000000000000	-0.000000000000	-4.390212942171
F	-2.284627115878	0.724627364463	-1.657121366789
F	-2.265260311678	0.725208207265	-4.355353627007
F	-0.000000000000	-0.000000000000	-5.735139323793
C	1.123195500625	-0.343448028244	-2.277428377015
C	1.152850881855	-0.362953499064	-3.677442143959
F	2.284627115878	-0.724627364463	-1.657121366789
F	2.265260311678	-0.725208207265	-4.355353627007
N	-0.000000000000	-2.940470319303	-0.037324637296
N	1.332726330706	-2.362750156753	1.562442292633

C	0.422284350895	-1.832214448829	0.674591496582
C	0.629594213327	-4.104373850981	0.397900981126
H	0.411427629952	-5.081107947635	-0.042273369983
C	1.475080198222	-3.741507993909	1.410904121205
H	2.148739074574	-4.331312670662	2.039090577696
C	1.912228399914	-1.600490103739	2.629802339156
C	1.176461682685	-1.485763048077	3.831664325367
C	1.757331164387	-0.765784928851	4.888233127182
H	1.199146937507	-0.669989543048	5.833506819640
C	3.020915816195	-0.156436943462	4.765243285596
C	3.721699734311	-0.305443696772	3.553941119280
H	4.716316162708	0.156251928987	3.445010354222
C	3.195393341670	-1.035723693218	2.472404577637
C	-0.195293855896	-2.094872987546	3.941362984408
H	-0.196714743378	-3.162082216639	3.639023908650
H	-0.900488786158	-1.568143842392	3.263872720275
H	-0.588212380700	-2.017952352772	4.971767012117
C	3.587514838191	0.668235174564	5.893300355540
H	3.326083379860	0.243120965759	6.882878048318
H	3.181281046709	1.703113721593	5.867683588486
H	4.690238526309	0.749057390537	5.830650080483
C	3.959331266266	-1.214288232829	1.187964465759
H	4.906378136546	-0.642661783030	1.204998043049
H	3.361277340932	-0.886255737927	0.315375415550
H	4.208345566736	-2.282067523651	1.012672360057
C	-0.963555818326	-2.887627485705	-1.098677752141
C	-2.282931276556	-2.479213695665	-0.800009318161
C	-3.187284809824	-2.351385031902	-1.868201226989
H	-4.211474913595	-2.007486179894	-1.653107503574
C	-2.813639810280	-2.614872479099	-3.197322338374
C	-1.499044721250	-3.051976899956	-3.444736793209
H	-1.183866859213	-3.252504737632	-4.481363936162
C	-0.552547093395	-3.194531970185	-2.416657762655
C	-2.690595998200	-2.129433122825	0.604952941377

H	-3.783235583826	-1.975044690320	0.680128492615
H	-2.174147368100	-1.195498558545	0.928661691285
H	-2.389071901887	-2.911421949213	1.331507220430
C	-3.766274014167	-2.364345027184	-4.337993631359
H	-3.621358767449	-3.089297155667	-5.163445867786
H	-3.591698423791	-1.349208076231	-4.753619130294
H	-4.823221391413	-2.413036028329	-4.010472616975
C	0.862058682044	-3.612849897163	-2.730595594077
H	1.041124364708	-4.684084793487	-2.496992398720
H	1.600006666156	-3.024467887738	-2.152222654997
H	1.081719347936	-3.466380002826	-3.805099824628

96

12_opt ! 2-A (C2): E(UPBE-D3(BJ)/def2SVP) = -3452.51607609

E(UPBE0-D3BJ/pcSseg-2) = -3454.95830517

Ni	0.000000000000	0.000000000000	0.105314268319
N	0.633514977614	-2.904655012499	0.088562020864
N	-1.419801159160	-2.485748666245	-0.418090187343
C	-0.230238731429	-1.859223096770	-0.133918277162
C	0.000000000000	-4.139115670631	-0.050183549769
H	0.533453891427	-5.083235742962	0.093164333808
C	-1.304960940799	-3.873690949064	-0.372047903354
H	-2.153198957705	-4.536413526245	-0.566394396485
C	-2.639932009105	-1.772119444047	-0.666131913707
C	-3.028611348512	-1.539630156408	-2.003182521485
C	-4.265211637508	-0.911887593070	-2.227616491545
H	-4.584202837707	-0.721990789918	-3.265502455311
C	-5.101746823614	-0.526161847923	-1.162588598827
C	-4.651247021699	-0.735040979811	0.153405689824
H	-5.275499451391	-0.402045322364	0.997055653831
C	-3.417693449079	-1.349334421639	0.434640780632
C	-2.107630760267	-1.912941236886	-3.134953359513
H	-1.894087355874	-3.001799841192	-3.157548734969
H	-1.126773128554	-1.404513602105	-3.012463481490

H	-2.535186582018	-1.624429997596	-4.113784000758
C	-6.455805722723	0.080388479985	-1.434110518033
H	-6.422657298330	0.789955069161	-2.285788878224
H	-6.847624923205	0.619511200304	-0.550480651838
H	-7.197309127914	-0.703968908092	-1.699043070101
C	-2.910977940157	-1.511834183459	1.841485463052
H	-3.660786393527	-1.162621448675	2.576105183844
H	-1.968699170808	-0.932354575566	1.986043582029
H	-2.664152624691	-2.570354758335	2.067799611004
C	2.010345540911	-2.756434785007	0.469978898344
C	2.991812440028	-2.699284570332	-0.540936435711
C	4.339771441168	-2.670692191515	-0.144415964929
H	5.119294634752	-2.645115650958	-0.921378734069
C	4.712213589757	-2.665584917306	1.211960275827
C	3.696924305384	-2.669042005630	2.186773080975
H	3.970659804667	-2.641587720950	3.254071224262
C	2.333152228174	-2.716436332001	1.845366955491
C	2.589349706658	-2.614705644761	-1.989054947452
H	3.474427123599	-2.604879601811	-2.652580719001
H	2.014625782700	-1.678577080517	-2.158933559005
H	1.929005919820	-3.453669767880	-2.291273214449
C	6.164226519715	-2.612259287195	1.618240229926
H	6.363412174149	-3.242600119748	2.507977322096
H	6.467190841558	-1.576481940458	1.886942538570
H	6.831791984807	-2.948217188726	0.800811683674
C	1.247521814173	-2.660651001314	2.882084765671
H	0.512409835490	-3.482559504352	2.757016271917
H	0.690848748619	-1.698235987845	2.760543561074
H	1.666751578985	-2.718450315195	3.904459240616
F	0.000000000000	0.000000000000	2.045934477407
N	-0.633514977614	2.904655012499	0.088562020864
N	1.419801159160	2.485748666245	-0.418090187343
C	0.230238731429	1.859223096770	-0.133918277162
C	0.000000000000	4.139115670631	-0.050183549769

H	-0.533453891427	5.083235742962	0.093164333808
C	1.304960940799	3.873690949064	-0.372047903354
H	2.153198957705	4.536413526245	-0.566394396485
C	2.639932009105	1.772119444047	-0.666131913707
C	3.028611348512	1.539630156408	-2.003182521485
C	4.265211637508	0.911887593070	-2.227616491545
H	4.584202837707	0.721990789918	-3.265502455311
C	5.101746823614	0.526161847923	-1.162588598827
C	4.651247021699	0.735040979811	0.153405689824
H	5.275499451391	0.402045322364	0.997055653831
C	3.417693449079	1.349334421639	0.434640780632
C	2.107630760267	1.912941236886	-3.134953359513
H	1.894087355874	3.001799841192	-3.157548734969
H	1.126773128554	1.404513602105	-3.012463481490
H	2.535186582018	1.624429997596	-4.113784000758
C	6.455805722723	-0.080388479985	-1.434110518033
H	6.422657298330	-0.789955069161	-2.285788878224
H	6.847624923205	-0.619511200304	-0.550480651838
H	7.197309127914	0.703968908092	-1.699043070101
C	2.910977940157	1.511834183459	1.841485463052
H	3.660786393527	1.162621448675	2.576105183844
H	1.968699170808	0.932354575566	1.986043582029
H	2.664152624691	2.570354758335	2.067799611004
C	-2.010345540911	2.756434785007	0.469978898344
C	-2.991812440028	2.699284570332	-0.540936435711
C	-4.339771441168	2.670692191515	-0.144415964929
H	-5.119294634752	2.645115650958	-0.921378734069
C	-4.712213589757	2.665584917306	1.211960275827
C	-3.696924305384	2.669042005630	2.186773080975
H	-3.970659804667	2.641587720950	3.254071224262
C	-2.333152228174	2.716436332001	1.845366955491
C	-2.589349706658	2.614705644761	-1.989054947452
H	-3.474427123599	2.604879601811	-2.652580719001
H	-2.014625782700	1.678577080517	-2.158933559005

H	-1.929005919820	3.453669767880	-2.291273214449
C	-6.164226519715	2.612259287195	1.618240229926
H	-6.363412174149	3.242600119748	2.507977322096
H	-6.467190841558	1.576481940458	1.886942538570
H	-6.831791984807	2.948217188726	0.800811683674
C	-1.247521814173	2.660651001314	2.882084765671
H	-0.512409835490	3.482559504352	2.757016271917
H	-0.690848748619	1.698235987845	2.760543561074
H	-1.666751578985	2.718450315195	3.904459240616

106

13_opt ! 2-A (C2): E(UPBE-D3(BJ)/def2SVP) = -3980.32739022

E(UPBE0-D3BJ/pcSseg-2) = -3983.34948566

Ni	-0.000000000000	-0.000000000000	0.299499000320
N	0.000000000000	2.940559342129	-0.141595356401
N	-1.346132711472	2.356196965822	1.444495069944
C	-0.427651632522	1.829426039056	0.562262461671
C	-0.634251740262	4.102491073216	0.291825883112
H	-0.413194698149	5.080912579950	-0.143128107685
C	-1.488135168035	3.735474303569	1.296283278983
H	-2.167506856555	4.322666291324	1.920761163297
C	-1.928442625547	1.591828430222	2.508746049326
C	-1.196436380867	1.476643567765	3.713043692134
C	-1.778802498308	0.753248729948	4.766303474041
H	-1.223089917748	0.656402319537	5.712974030999
C	-3.040706245632	0.141302923975	4.638084162972
C	-3.738178148313	0.291693812651	3.425158673861
H	-4.731421864565	-0.172011095609	3.312218181870
C	-3.209915330552	1.024894055154	2.346415720875
C	0.173663694181	2.088390723654	3.828062095755
H	0.173607923674	3.156381124582	3.528452131921
H	0.881353765661	1.564866462825	3.150785693523
H	0.564241743561	2.009476378008	4.859228911934
C	-3.608446856628	-0.687014449245	5.762938896108

H	-3.364921758315	-0.253949036247	6.753722977461
H	-3.186517380625	-1.715760882117	5.746814473973
H	-4.709049250942	-0.784139213069	5.687812957652
C	-3.969206233496	1.202444317882	1.059155053220
H	-4.916820616256	0.631651154642	1.073700525566
H	-3.368159802944	0.872762164815	0.189142661049
H	-4.216433795522	2.270183428858	0.881239902921
C	0.974711944329	2.889361252593	-1.192342371536
C	2.290364775514	2.478786648702	-0.879444973180
C	3.205569978678	2.349547981142	-1.937572209091
H	4.226582054577	2.002986991021	-1.711729881954
C	2.846716982309	2.615642025929	-3.270366231619
C	1.536804521391	3.057595397035	-3.531269865079
H	1.233242930845	3.261452871970	-4.570479761626
C	0.578615122048	3.199004699394	-2.513821801149
C	2.683777807231	2.130966488719	0.530055258483
H	3.776105185285	1.980654443756	0.617228346264
H	2.167663297378	1.195296996791	0.848684162990
H	2.371598554403	2.912236629521	1.252835091300
C	3.812498434412	2.359911592275	-4.398683719075
H	3.641585049823	3.045619331404	-5.252085097992
H	3.680528615184	1.322274425750	-4.772844192101
H	4.865771776088	2.464005453013	-4.071531283851
C	-0.832502524058	3.617991174628	-2.841873203105
H	-1.018187348121	4.685751229461	-2.597733054329
H	-1.576221077876	3.019966992856	-2.280830785512
H	-1.037095774638	3.483101529729	-3.920758732081
C	0.000000000000	0.000000000000	-1.639912980017
C	-1.125311501113	0.342597843630	-2.395288099166
C	-1.141419717022	0.354558978823	-3.795847172429
C	0.000000000000	0.000000000000	-4.523028664671
F	-2.286150435645	0.725632664479	-1.773490139634
F	-2.270002125914	0.718682251965	-4.455365836358
H	0.000000000000	0.000000000000	-5.620371855152

C	1.125311501113	-0.342597843630	-2.395288099166
C	1.141419717022	-0.354558978823	-3.795847172429
F	2.286150435645	-0.725632664479	-1.773490139634
F	2.270002125914	-0.718682251965	-4.455365836358
N	-0.000000000000	-2.940559342129	-0.141595356401
N	1.346132711472	-2.356196965822	1.444495069944
C	0.427651632522	-1.829426039056	0.562262461671
C	0.634251740262	-4.102491073216	0.291825883112
H	0.413194698149	-5.080912579950	-0.143128107685
C	1.488135168035	-3.735474303569	1.296283278983
H	2.167506856555	-4.322666291324	1.920761163297
C	1.928442625547	-1.591828430222	2.508746049326
C	1.196436380867	-1.476643567765	3.713043692134
C	1.778802498308	-0.753248729948	4.766303474041
H	1.223089917748	-0.656402319537	5.712974030999
C	3.040706245632	-0.141302923975	4.638084162972
C	3.738178148313	-0.291693812651	3.425158673861
H	4.731421864565	0.172011095609	3.312218181870
C	3.209915330552	-1.024894055154	2.346415720875
C	-0.173663694181	-2.088390723654	3.828062095755
H	-0.173607923674	-3.156381124582	3.528452131921
H	-0.881353765661	-1.564866462825	3.150785693523
H	-0.564241743561	-2.009476378008	4.859228911934
C	3.608446856628	0.687014449245	5.762938896108
H	3.364921758315	0.253949036247	6.753722977461
H	3.186517380625	1.715760882117	5.746814473973
H	4.709049250942	0.784139213069	5.687812957652
C	3.969206233496	-1.202444317882	1.059155053220
H	4.916820616256	-0.631651154642	1.073700525566
H	3.368159802944	-0.872762164815	0.189142661049
H	4.216433795522	-2.270183428858	0.881239902921
C	-0.974711944329	-2.889361252593	-1.192342371536
C	-2.290364775514	-2.478786648702	-0.879444973180
C	-3.205569978678	-2.349547981142	-1.937572209091

H	-4.226582054577	-2.002986991021	-1.711729881954
C	-2.846716982309	-2.615642025929	-3.270366231619
C	-1.536804521391	-3.057595397035	-3.531269865079
H	-1.233242930845	-3.261452871970	-4.570479761626
C	-0.578615122048	-3.199004699394	-2.513821801149
C	-2.683777807231	-2.130966488719	0.530055258483
H	-3.776105185285	-1.980654443756	0.617228346264
H	-2.167663297378	-1.195296996791	0.848684162990
H	-2.371598554403	-2.912236629521	1.252835091300
C	-3.812498434412	-2.359911592275	-4.398683719075
H	-3.641585049823	-3.045619331404	-5.252085097992
H	-3.680528615184	-1.322274425750	-4.772844192101
H	-4.865771776088	-2.464005453013	-4.071531283851
C	0.832502524058	-3.617991174628	-2.841873203105
H	1.018187348121	-4.685751229461	-2.597733054329
H	1.576221077876	-3.019966992856	-2.280830785512
H	1.037095774638	-3.483101529729	-3.920758732081

106

14_opt ! 2-A (C1): E(UPBE-D3(BJ)/def2SVP) = -3881.24599981

E(UPBE0-D3BJ/pcSseg-2) = -3884.15487437

Ni	-0.277268891804	0.032600499212	0.020203878934
N	0.115708717715	-1.484410649993	2.545321088131
N	-1.456378604345	-0.010586785064	2.713130566287
C	-0.567658296893	-0.538121161357	1.799380102750
C	-0.342154157814	-1.535596137270	3.860003352215
H	0.074708901473	-2.232712148224	4.591900606328
C	-1.337018403016	-0.602187676240	3.970390964036
H	-1.968564967855	-0.312370108829	4.815142154252
C	-2.480316562997	0.921497359113	2.343633683388
C	-3.734816450978	0.405813374516	1.945366065826
C	-4.742545084172	1.323823098909	1.608849009940
H	-5.726708997011	0.939103386083	1.295456710341
C	-4.522014259998	2.714731260390	1.642972552217

C	-3.258165395912	3.183514350836	2.047306059472
H	-3.069950934568	4.268717388998	2.080666474805
C	-2.220815857840	2.306109433810	2.412482258192
C	-3.941383534538	-1.081706933008	1.846068673201
H	-3.706986887763	-1.596822004710	2.800114732850
H	-3.262389794404	-1.505718333253	1.075815712863
H	-4.981212613203	-1.326043562098	1.560457334623
C	-5.605357622586	3.671731980940	1.213413217046
H	-6.602801441476	3.349003086775	1.573719559738
H	-5.665189249582	3.727758187624	0.104631726564
H	-5.418528285998	4.698092434405	1.584621449493
C	-0.862747723618	2.805525877234	2.826019135497
H	-0.822212221784	3.910746897562	2.828361593083
H	-0.085063675074	2.427160482516	2.129451657048
H	-0.582449798831	2.442694054160	3.836320013135
C	1.177566398767	-2.293909659723	2.023095344883
C	0.885823693675	-3.208465363227	0.985496247768
C	1.954879905426	-3.926645390018	0.425141250937
H	1.745594719430	-4.621168125118	-0.403988892024
C	3.278843343658	-3.760193166689	0.867883803787
C	3.517443652991	-2.871288669592	1.931445014594
H	4.549065608336	-2.721111768955	2.287750057854
C	2.487831419902	-2.122836350629	2.525684679515
C	-0.514762790736	-3.368876998031	0.462121416594
H	-0.589331436719	-4.226634715954	-0.232007472923
H	-0.830490482057	-2.445856360718	-0.077179467656
H	-1.249550048991	-3.508310061049	1.281453891809
C	4.421520139613	-4.455667423120	0.173943972406
H	5.252839410277	-4.680807083470	0.871131740373
H	4.825157200255	-3.797416770341	-0.624617771742
H	4.099569557329	-5.401652670686	-0.304207399462
C	2.796177781812	-1.149575118853	3.634903863556
H	2.639375950984	-1.602420405642	4.637829603873
H	2.155869096728	-0.249491992823	3.574529329742

H	3.849777406749	-0.816215374316	3.579769707441
C	1.649339267336	0.098770848782	0.103545132228
C	2.356283117345	0.940137432978	0.994967914271
C	3.752334607041	0.951368714231	1.022740905239
C	4.529620931742	0.136770787970	0.190246752587
H	1.829025234843	1.616306128659	1.686362040387
F	4.394300041462	1.776715976885	1.897883176167
H	5.626386229861	0.148665168345	0.220945918224
C	2.437151591006	-0.708894287581	-0.723686063629
C	3.836190905019	-0.696301326413	-0.695202104363
F	1.856336583395	-1.535551789951	-1.654055498856
F	4.534686846269	-1.493254835448	-1.543665665418
N	0.179671476977	1.485408572545	-2.539995121958
N	-1.343506939894	-0.036986024774	-2.715350207501
C	-0.512353349914	0.553988731038	-1.786852665871
C	-0.208007980219	1.461853810539	-3.877472111687
H	0.226418598673	2.137025126499	-4.619778808754
C	-1.172692890283	0.497541002837	-3.991946196067
H	-1.756595381825	0.155568527915	-4.851285592889
C	-2.376817098478	-0.961118194890	-2.350025986996
C	-3.591490975485	-0.432384222368	-1.853877342009
C	-4.607695569035	-1.337682436361	-1.510453516939
H	-5.559842167392	-0.941572285528	-1.121758365506
C	-4.437598215042	-2.730239097969	-1.639844264097
C	-3.220601944691	-3.210442811173	-2.156902288366
H	-3.074941666807	-4.296431093838	-2.274171160521
C	-2.174577044848	-2.345500763906	-2.529433513076
C	-3.755918919052	1.052419833496	-1.675405719450
H	-3.465700293577	1.610163350950	-2.589116237293
H	-3.093819694424	1.411072325954	-0.858637295645
H	-4.797811674437	1.314153474577	-1.413933780201
C	-5.527575882299	-3.676649624729	-1.203373008292
H	-6.528060488866	-3.325020766583	-1.527336387699
H	-5.558856122963	-3.759311658069	-0.095152390063

H	-5.372668969887	-4.696389751264	-1.605999104129
C	-0.874236573550	-2.865803047882	-3.081161048785
H	-0.858510255368	-3.972093802558	-3.086293364910
H	-0.013688306683	-2.500630017114	-2.486364578599
H	-0.705924428212	-2.517763090286	-4.121874774383
C	1.176099431849	2.356513604434	-1.989661641155
C	0.777057146490	3.326793239676	-1.043725771053
C	1.780214224364	4.098202786849	-0.432388824123
H	1.486060995503	4.844081809658	0.323912613222
C	3.141636752396	3.929075088330	-0.741020548653
C	3.488427817660	2.984600113351	-1.723994795547
H	4.549946775410	2.834378024763	-1.977224959306
C	2.527974375424	2.182712250436	-2.362898177897
C	-0.670643957986	3.493743291863	-0.668569541005
H	-0.820403438039	4.382312017342	-0.026811117689
H	-1.032170393765	2.593430865793	-0.122641891033
H	-1.319767922167	3.588852639759	-1.563137145877
C	4.210582748299	4.681136020584	0.009238735546
H	5.060610634523	4.954440545244	-0.647450878264
H	4.613774767421	4.041448551707	0.823119358333
H	3.816343237006	5.606546202132	0.473051541534
C	2.941013330788	1.152100161814	-3.382773174035
H	2.752345313823	1.500611131075	-4.420786071882
H	2.388970326856	0.202028499882	-3.250434576731
H	4.021231007719	0.929298149165	-3.297267862214

100

8_1_dft_opt ! 2-A (C1): E(UPBE-D3(BJ)/def2SVP) = -3776.57139912

E(UPBE0-D3BJ/pcSseg-2) = -3779.40710069

Ni	0.025664701513	-0.051672394445	-0.027538258749
N	-0.973085058367	2.605552691029	-0.834047576824
N	1.131249351358	2.699747931466	-0.364059997787
C	0.087357224250	1.811714358834	-0.470602966032
C	-0.603683955178	3.944559196685	-0.943670559860

H	-1.316069010085	4.721303832241	-1.234969540172
C	0.730140385782	4.003438951037	-0.646771537005
H	1.429101062315	4.843701149314	-0.623250144132
C	2.488208755084	2.338839318950	-0.059814350226
C	3.065010487517	2.773307231671	1.154165613085
C	4.411365896637	2.439101935776	1.388540483906
H	4.871900884526	2.759519979148	2.336963385474
C	5.179982782200	1.719473321379	0.457987716134
C	4.568144156663	1.321865595709	-0.744651668947
H	5.153967016515	0.772079278746	-1.496036399769
C	3.223113539018	1.611795363147	-1.024695360408
C	2.286076013047	3.577771999641	2.159736828381
H	1.254018968023	3.198647743746	2.281423634022
H	2.219770862431	4.645794894032	1.859706340595
H	2.782774655642	3.546091287502	3.147994876394
C	6.612956614474	1.354918466615	0.754429070067
H	6.676893686901	0.346177824867	1.217928276647
H	7.079990456309	2.066771388031	1.462901625895
H	7.228402694131	1.329077444572	-0.166927517617
C	2.587600332251	1.158664317297	-2.312495684441
H	1.824451988459	0.375800995474	-2.112566509279
H	3.341208606697	0.732816609422	-3.000443892040
H	2.056640914013	1.986322922097	-2.825986602046
C	-2.288321623759	2.126441934964	-1.160131000908
C	-3.357433657193	2.385804203456	-0.273578637071
C	-4.645802659538	1.995246098064	-0.679864527868
H	-5.490311749593	2.188715131203	0.001253084381
C	-4.885887393940	1.371803744804	-1.917624825739
C	-3.787050908759	1.107631300152	-2.755097178640
H	-3.950592241433	0.601719924295	-3.720360297180
C	-2.478110329654	1.476337089077	-2.400412927198
C	-3.122313585716	3.050754674467	1.055010217655
H	-2.275083409826	2.586713707344	1.596772187891
H	-4.026145641844	2.988044897649	1.689895517475

H	-2.871716228130	4.126308467494	0.935257922643
C	-6.288567966657	1.011836702959	-2.339421202799
H	-6.881877303183	0.621543220469	-1.488752367274
H	-6.292592106590	0.248737789545	-3.142487688866
H	-6.829020038677	1.901463584805	-2.728447962473
C	-1.305229635021	1.166709687542	-3.291329297675
H	-0.739424503558	2.082583689359	-3.561991070146
H	-1.625331010784	0.661608932453	-4.221740077820
H	-0.591299987716	0.511166025749	-2.745022285537
N	1.020815749739	-2.834420452789	-0.072971825036
N	-1.084426468020	-2.771808849428	0.394210459688
C	-0.024852858359	-1.952495658589	0.071978413597
C	0.627854116717	-4.153523934567	0.145291018507
H	1.331108107201	-4.987832699808	0.071625958897
C	-0.707593776412	-4.111440473494	0.440397656334
H	-1.424335657641	-4.904086443297	0.673176642422
C	-2.441647643577	-2.333409198916	0.579452367173
C	-2.979325525203	-2.260784926335	1.881776987325
C	-4.321380873726	-1.854078573427	2.003614920688
H	-4.756667612909	-1.780879231466	3.013228816857
C	-5.114036054382	-1.538291722644	0.887508204121
C	-4.541933956063	-1.652280132547	-0.393390363399
H	-5.151864183159	-1.433560111781	-1.282454779789
C	-3.207260287821	-2.046660055715	-0.573047184583
C	-2.155697652049	-2.558683469369	3.104505898755
H	-1.572096095580	-1.661664144197	3.400814280043
H	-1.431701692166	-3.380097944594	2.939887581528
H	-2.805076984559	-2.834028511956	3.957608489659
C	-6.533149860686	-1.057780041269	1.056988465607
H	-6.570397602718	0.053390228466	1.063485264656
H	-6.974151208588	-1.404236860682	2.012044398096
H	-7.184986948798	-1.400684244354	0.228665724587
C	-2.599210821095	-2.158629900916	-1.945796047944
H	-1.785181939157	-1.412978737106	-2.062233747439

H	-3.354042798052	-1.978945265678	-2.733360320633
H	-2.141811359528	-3.156113795255	-2.113126592211
C	2.356905118907	-2.473893261792	-0.455326983110
C	3.321620250949	-2.231577711680	0.547675138590
C	4.646345576934	-2.013522338553	0.129824700118
H	5.414729724799	-1.829280772318	0.897491590329
C	5.014630778567	-2.019553652803	-1.228098412504
C	4.010121781607	-2.215415176107	-2.194128103756
H	4.274226034100	-2.193531980525	-3.263884686579
C	2.672444584059	-2.449212580238	-1.831463772936
C	2.930434261286	-2.172054788667	1.998637267151
H	2.318550726552	-1.265547156765	2.195810125666
H	3.823360742555	-2.135776634025	2.650485125869
H	2.312705363840	-3.043113677987	2.297520070288
C	6.454127777987	-1.832585493864	-1.637809410867
H	6.995096453679	-1.167424884459	-0.936688145708
H	6.538453244799	-1.404964778280	-2.656543172362
H	6.994114059064	-2.803925529242	-1.646333765290
C	1.593779185033	-2.650108832467	-2.863643893895
H	1.124648957001	-3.652650734349	-2.778252046388
H	1.993822482092	-2.541774524070	-3.889214189292
H	0.774009266217	-1.913464407174	-2.723405672998
F	-0.550366463154	0.133015807213	3.918978092943
F	1.052804552252	0.557482255798	2.302335566565
B	-0.276355722153	0.790423552805	2.729263035626
F	-1.153104351953	0.246216188203	1.680129261238
F	-0.532583655283	2.165449165478	2.813828944031

100

8_1_xrd_opt ! 2-A (C2): E(UPBE-D3(BJ)/def2SVP) = -3776.47417277

E(UPBE0-D3BJ/pcSseg-2) = -3779.36617894

Ni	0.000000000000	0.000000000000	0.935755973750
N	-0.549777211387	2.790533530812	0.254300973726
N	1.319794002185	2.611657429939	1.266095973825

C	0.261007022017	1.873317044033	0.844660973767
C	0.000000000000	4.063226352364	0.311834973757
H	-0.441063917369	4.981399350087	-0.087820528359
C	1.175413862271	3.957163234461	0.949499973819
H	1.877064061297	4.764876330575	1.176091724331
C	2.466028709631	2.070400805060	1.946911973885
C	2.547654859472	2.202619946221	3.333954973889
C	3.693864365776	1.742185774751	3.958036973950
H	3.791666158092	1.862768339064	5.049664990863
C	4.720399521225	1.132691342061	3.254046974003
C	4.581611211209	0.979541223374	1.880907973996
H	5.375064809216	0.462115698892	1.317751050965
C	3.473575913947	1.457429702457	1.197853973937
C	1.418952355163	2.802912403424	4.126641973830
H	0.492908403410	2.218437944023	3.949485637785
H	1.261900773735	3.852819440819	3.807000641435
H	1.671507667656	2.780247820687	5.202437977293
C	5.976238919092	0.686326759073	3.955638974069
H	5.713982846832	0.251713457390	4.939963479068
H	6.646486838550	1.557891403025	4.103991978482
H	6.491218655470	-0.075816682777	3.340288947985
C	3.372727415766	1.333281366342	-0.294667026068
H	2.590129898536	0.588877343369	-0.552856373111
H	4.344465583510	1.003470130157	-0.702553563017
H	3.103714178658	2.316583290404	-0.728687850662
C	-1.798194499296	2.472571998450	-0.390876026341
C	-2.971268731901	2.484563466134	0.364093973596
C	-4.171001614404	2.241967897659	-0.301539026468
H	-5.112383071068	2.230963815303	0.271498454361
C	-4.206890695536	2.010345100313	-1.673080026470
C	-3.023051010530	1.985298459917	-2.369878026407
H	-3.046805682803	1.776698718015	-3.452572219766
C	-1.792672463040	2.212383805226	-1.759381026341
C	-2.952662104997	2.735361352555	1.841273973598

H	-2.445143566948	1.890602039470	2.353522570066
H	-3.990122391618	2.821044385297	2.212729531246
H	-2.405373827652	3.676851298623	2.049405550357
C	-5.530277361676	1.795190226467	-2.369878026541
H	-6.281137165391	1.446311718040	-1.635118877083
H	-5.407742551763	1.033758497201	-3.164191796632
H	-5.868848327324	2.750475702928	-2.820710279558
C	-0.505180872685	2.140335233718	-2.532890026273
H	0.044330636333	3.097120696709	-2.424573752411
H	-0.726957993613	1.958691242848	-3.602772496217
H	0.116053617960	1.309771772386	-2.138762219956
N	0.549777211387	-2.790533530812	0.254300973726
N	-1.319794002185	-2.611657429939	1.266095973825
C	-0.261007022017	-1.873317044033	0.844660973767
C	-0.000000000000	-4.063226352364	0.311834973757
H	0.441063917369	-4.981399350087	-0.087820528359
C	-1.175413862271	-3.957163234461	0.949499973819
H	-1.877064061297	-4.764876330575	1.176091724331
C	-2.466028709631	-2.070400805060	1.946911973885
C	-2.547654859472	-2.202619946221	3.333954973889
C	-3.693864365776	-1.742185774751	3.958036973950
H	-3.791666158092	-1.862768339064	5.049664990863
C	-4.720399521225	-1.132691342061	3.254046974003
C	-4.581611211209	-0.979541223374	1.880907973996
H	-5.375064809216	-0.462115698892	1.317751050965
C	-3.473575913947	-1.457429702457	1.197853973937
C	-1.418952355163	-2.802912403424	4.126641973830
H	-0.492908403410	-2.218437944023	3.949485637785
H	-1.261900773735	-3.852819440819	3.807000641435
H	-1.671507667656	-2.780247820687	5.202437977293
C	-5.976238919092	-0.686326759073	3.955638974069
H	-5.713982846832	-0.251713457390	4.939963479068
H	-6.646486838550	-1.557891403025	4.103991978482
H	-6.491218655470	0.075816682777	3.340288947985

C	-3.372727415766	-1.333281366342	-0.294667026068
H	-2.590129898536	-0.588877343369	-0.552856373111
H	-4.344465583510	-1.003470130157	-0.702553563017
H	-3.103714178658	-2.316583290404	-0.728687850662
C	1.798194499296	-2.472571998450	-0.390876026341
C	2.971268731901	-2.484563466134	0.364093973596
C	4.171001614404	-2.241967897659	-0.301539026468
H	5.112383071068	-2.230963815303	0.271498454361
C	4.206890695536	-2.010345100313	-1.673080026470
C	3.023051010530	-1.985298459917	-2.369878026407
H	3.046805682803	-1.776698718015	-3.452572219766
C	1.792672463040	-2.212383805226	-1.759381026341
C	2.952662104997	-2.735361352555	1.841273973598
H	2.445143566948	-1.890602039470	2.353522570066
H	3.990122391618	-2.821044385297	2.212729531246
H	2.405373827652	-3.676851298623	2.049405550357
C	5.530277361676	-1.795190226467	-2.369878026541
H	6.281137165391	-1.446311718040	-1.635118877083
H	5.407742551763	-1.033758497201	-3.164191796632
H	5.868848327324	-2.750475702928	-2.820710279558
C	0.505180872685	-2.140335233718	-2.532890026273
H	-0.044330636333	-3.097120696709	-2.424573752411
H	0.726957993613	-1.958691242848	-3.602772496217
H	-0.116053617960	-1.309771772386	-2.138762219956
F	-0.648390126382	0.937780566677	-6.022153026283
F	0.928340447723	0.648335458011	-7.625587026199
B	0.000000000000	0.000000000000	-6.806370026250
F	0.648390126382	-0.937780566677	-6.022153026283
F	-0.928340447723	-0.648335458011	-7.625587026199

100

8_2_dft_opt ! 2-A (C2): E(UPBE-D3(BJ)/def2SVP) = -3776.53430266

E(UPBE0-D3BJ/pcSseg-2) = -3779.38576352

Ni	-0.000000000000	0.000000000000	0.703737798292

N	0.000000000000	2.808595932695	-0.119331583922
N	1.194121450058	2.482577329125	1.654466427406
C	0.403804780198	1.837850387684	0.745219770429
C	0.530136498293	4.044103156726	0.231490958929
H	0.331162064445	4.949184396423	-0.349864666236
C	1.288625849940	3.840741424708	1.361710587477
H	1.883522587904	4.531788870913	1.965980495625
C	1.780623553382	1.743316458031	2.739127615682
C	1.101660098543	1.702740651214	3.975704432227
C	1.649816031428	0.902537316307	4.995016724973
H	1.134447506568	0.859703819147	5.967591812450
C	2.816455670918	0.142582845815	4.797956049942
C	3.446240277229	0.194525344302	3.538154014215
H	4.353108440067	-0.405938108089	3.361633500676
C	2.948552199333	0.985013167958	2.488492889138
C	-0.205240505496	2.427229033272	4.162903823165
H	-0.989936924442	1.973091569244	3.519819866537
H	-0.138188481176	3.497075921543	3.880759384725
H	-0.551255971681	2.362284747324	5.211182481157
C	3.376774606237	-0.725623501362	5.895735464857
H	2.755099418279	-0.682642893309	6.810537504941
H	4.406125679498	-0.414237777127	6.169778663020
H	3.439363797150	-1.786201213396	5.574304181136
C	3.589368630990	0.977251313504	1.125844092721
H	2.939646121843	0.445047670984	0.396288884317
H	4.569909052380	0.466510410877	1.145699020912
H	3.731149752453	2.001157002568	0.725817262238
C	-0.861780893276	2.471763707759	-1.225985679875
C	-2.260409001813	2.503576001700	-1.025210699342
C	-3.076285222572	2.076785253959	-2.087573794407
H	-4.170507206246	2.092518601623	-1.953382321353
C	-2.533124883534	1.618830873200	-3.306056929279
C	-1.132912445113	1.582216638717	-3.448693275589
H	-0.690983833301	1.171296200052	-4.366734275018

C	-0.272665573799	2.003627287974	-2.420733771320
C	-2.842858468441	2.923802014848	0.301287326577
H	-2.549500460269	2.216822183198	1.108851030991
H	-3.948139633979	2.950526969862	0.257792001456
H	-2.488336037343	3.926798283191	0.617439002890
C	-3.407437872723	1.173689780174	-4.448455932260
H	-4.345733044645	0.704194170106	-4.090471393954
H	-2.857834642201	0.471639498520	-5.105824416134
H	-3.694162227415	2.045168894272	-5.075740463822
C	1.221534575893	1.892141869707	-2.584593734555
H	1.743935944634	2.831819974880	-2.309915267675
H	1.464388756035	1.627118599465	-3.630272307539
H	1.628487072192	1.090690560740	-1.929472355362
N	-0.000000000000	-2.808595932695	-0.119331583922
N	-1.194121450058	-2.482577329125	1.654466427406
C	-0.403804780198	-1.837850387684	0.745219770429
C	-0.530136498293	-4.044103156726	0.231490958929
H	-0.331162064445	-4.949184396423	-0.349864666236
C	-1.288625849940	-3.840741424708	1.361710587477
H	-1.883522587904	-4.531788870913	1.965980495625
C	-1.780623553382	-1.743316458031	2.739127615682
C	-1.101660098543	-1.702740651214	3.975704432227
C	-1.649816031428	-0.902537316307	4.995016724973
H	-1.134447506568	-0.859703819147	5.967591812450
C	-2.816455670918	-0.142582845815	4.797956049942
C	-3.446240277229	-0.194525344302	3.538154014215
H	-4.353108440067	0.405938108089	3.361633500676
C	-2.948552199333	-0.985013167958	2.488492889138
C	0.205240505496	-2.427229033272	4.162903823165
H	0.989936924442	-1.973091569244	3.519819866537
H	0.138188481176	-3.497075921543	3.880759384725
H	0.551255971681	-2.362284747324	5.211182481157
C	-3.376774606237	0.725623501362	5.895735464857
H	-2.755099418279	0.682642893309	6.810537504941

H	-4.406125679498	0.414237777127	6.169778663020
H	-3.439363797150	1.786201213396	5.574304181136
C	-3.589368630990	-0.977251313504	1.125844092721
H	-2.939646121843	-0.445047670984	0.396288884317
H	-4.569909052380	-0.466510410877	1.145699020912
H	-3.731149752453	-2.001157002568	0.725817262238
C	0.861780893276	-2.471763707759	-1.225985679875
C	2.260409001813	-2.503576001700	-1.025210699342
C	3.076285222572	-2.076785253959	-2.087573794407
H	4.170507206246	-2.092518601623	-1.953382321353
C	2.533124883534	-1.618830873200	-3.306056929279
C	1.132912445113	-1.582216638717	-3.448693275589
H	0.690983833301	-1.171296200052	-4.366734275018
C	0.272665573799	-2.003627287974	-2.420733771320
C	2.842858468441	-2.923802014848	0.301287326577
H	2.549500460269	-2.216822183198	1.108851030991
H	3.948139633979	-2.950526969862	0.257792001456
H	2.488336037343	-3.926798283191	0.617439002890
C	3.407437872723	-1.173689780174	-4.448455932260
H	4.345733044645	-0.704194170106	-4.090471393954
H	2.857834642201	-0.471639498520	-5.105824416134
H	3.694162227415	-2.045168894272	-5.075740463822
C	-1.221534575893	-1.892141869707	-2.584593734555
H	-1.743935944634	-2.831819974880	-2.309915267675
H	-1.464388756035	-1.627118599465	-3.630272307539
H	-1.628487072192	-1.090690560740	-1.929472355362
F	-0.960815797250	-0.648710287067	-5.683253577631
F	-0.655090904442	0.958989114005	-7.315074288515
B	0.000000000000	-0.000000000000	-6.553263805350
F	0.960815797250	0.648710287067	-5.683253577631
F	0.655090904442	-0.958989114005	-7.315074288515

100

8_2_xrd_opt ! 2-A (C1): E(UPBE-D3(BJ)/def2SVP) = -3776.48642462

E(UPBE0-D3BJ/pcSseg-2) = -3779.37574802

Ni	0.742832996628	0.334090710521	0.151961683491
N	-1.033776718910	-1.904621996774	-0.468954267815
N	-2.093649537052	-0.354010391265	0.542626456859
C	-0.852387540600	-0.685021734746	0.102758293930
C	-2.357263943900	-2.312257659268	-0.382196598099
H	-2.776409346658	-3.249754588386	-0.762780637841
C	-3.028449548713	-1.341510850165	0.255474399762
H	-4.105613479431	-1.329455592351	0.503987818044
C	-2.414276965730	0.877686283634	1.213778092100
C	-2.542623138208	0.871521387762	2.603570259137
C	-2.925703389071	2.049996712110	3.219784209565
H	-3.061055689268	2.059933271334	4.313884762109
C	-3.141853950697	3.217390305571	2.504710128415
C	-2.960219483594	3.193857622487	1.128235282883
H	-3.092481479377	4.126423481153	0.555248223449
C	-2.616270736270	2.032326559446	0.453529988536
C	-2.251513625483	-0.366457183773	3.406899186355
H	-1.208871964860	-0.693378289449	3.214902991357
H	-2.953538847083	-1.171809780503	3.110111272543
H	-2.378775736257	-0.146042631726	4.482412743323
C	-3.605423135477	4.471076011930	3.198958664121
H	-3.086779324294	4.566389950045	4.173026837463
H	-4.700515383022	4.416671144720	3.367913912442
H	-3.371615591426	5.350009236149	2.567850701117
C	-2.483490198897	2.017294127238	-1.041577204705
H	-1.413660536685	1.902266418828	-1.317881185709
H	-2.871484263471	2.965656778990	-1.454779946234
H	-3.064207544723	1.168624310603	-1.454427900252
C	0.007019352866	-2.655638601324	-1.123642146801
C	0.773474980047	-3.545828011731	-0.371089665929
C	1.724989577346	-4.309023582417	-1.044462803194
H	2.355091482338	-5.010352729679	-0.473863242577
C	1.899477022978	-4.203336766604	-2.420851661534

C	1.137009793498	-3.295745051350	-3.115511426510
H	1.291364084453	-3.192107081665	-4.202635084474
C	0.176381710150	-2.499875881773	-2.497537590762
C	0.597552877002	-3.675765260481	1.111298361495
H	0.919294239777	-2.735126908741	1.605703238881
H	1.212948506456	-4.515553432019	1.482342025789
H	-0.469346617331	-3.868232497103	1.341206894307
C	2.909874604815	-5.078771882487	-3.125040148557
H	3.675920215777	-5.413774057555	-2.399901121817
H	3.394873216888	-4.501051443241	-3.935983746961
H	2.395342942181	-5.962340547326	-3.555457513880
C	-0.619066144299	-1.483922533927	-3.269705493953
H	-1.700984673233	-1.685921901000	-3.139185203764
H	-0.356868105346	-1.548081733546	-4.341761048738
H	-0.383915526644	-0.466827219526	-2.892525636608
N	2.494703860989	2.555487337308	-0.589697798234
N	3.591307858631	1.030585901475	0.421814726482
C	2.334746513366	1.350888390537	0.019019797029
C	3.820279741962	2.964584964827	-0.561415714828
H	4.224595650298	3.891211707195	-0.980335899923
C	4.514614490205	2.010041512478	0.075930219469
H	5.588507395097	2.003873157656	0.283175244850
C	3.936650940878	-0.183810930197	1.111960837995
C	4.115350915665	-0.142400642839	2.495573688687
C	4.521087206501	-1.305017760622	3.127217339123
H	4.696058415833	-1.287219370140	4.215547263624
C	4.711680829166	-2.490300059156	2.434657051021
C	4.480197316468	-2.501659460614	1.065528052518
H	4.591956836476	-3.448123000848	0.512238998029
C	4.111451669493	-1.357485109395	0.374460698880
C	3.853018307831	1.115720464754	3.277229584066
H	2.804135236042	1.438080824878	3.114987919850
H	4.543392459858	1.912897207896	2.934816850645
H	4.019310548431	0.922667007098	4.352522680793

C	5.200719898349	-3.726157994708	3.143248061989
H	4.717957418523	-3.796435730972	4.137311124320
H	6.301270933642	-3.667979732726	3.270918475295
H	4.944669044047	-4.620373745754	2.543814594036
C	3.924488163476	-1.380378312474	-1.114741340158
H	2.845287122362	-1.271914073448	-1.354866666254
H	4.297524212759	-2.338743573412	-1.517373578835
H	4.489603796321	-0.542506557687	-1.569949090869
C	1.430485937754	3.290109701779	-1.225047163316
C	0.691437975749	4.199483242173	-0.468030304063
C	-0.284241086885	4.945764776546	-1.125596367793
H	-0.893268993553	5.661333139156	-0.550762338730
C	-0.508519565941	4.805226482095	-2.491616138041
C	0.228651815652	3.879928813749	-3.190210273300
H	0.035038847098	3.748810449351	-4.267764713474
C	1.211442762911	3.099572649728	-2.587489838497
C	0.920986123334	4.366956304445	1.003216432605
H	0.617811876166	3.439260472775	1.532749075064
H	0.319299400450	5.215867168924	1.374758513307
H	1.995814301919	4.564779191243	1.189352778452
C	-1.544212995667	5.662955644884	-3.180681551353
H	-2.283316276930	6.016493612074	-2.437237432044
H	-2.057884008025	5.065246487571	-3.958286468288
H	-1.046015924122	6.535126131762	-3.651828924449
C	1.978809931130	2.063964087499	-3.361960173528
H	3.064877360265	2.268756070087	-3.275949639055
H	1.677929195426	2.100978520870	-4.424782323971
H	1.757958225305	1.056821168927	-2.950750442721
F	-7.472987547604	-2.551131052941	-0.877585244796
F	-5.282219137812	-3.170806648920	-0.758422591059
B	-6.430216324668	-2.876844365770	-0.016531466392
F	-6.155159393526	-1.816599432445	0.840847656770
F	-6.791606613864	-4.002947239600	0.729124742750

100

8_3_dft_opt ! 2-A (C1): E(UPBE-D3(BJ)/def2SVP) = -3776.54685202

E(UPBE0-D3BJ/pcSseg-2) = -3779.38535924

Ni	-0.222542230256	-0.311633236793	0.050106840176
N	1.432937654911	1.045501543391	2.105831645035
N	-0.453457777340	2.049186583617	1.776603509355
C	0.318716651783	1.020218123151	1.308493106621
C	1.357748609099	2.075353267006	3.043374327184
H	2.148227618848	2.251861703828	3.777224468669
C	0.164268226394	2.710404278120	2.833338641518
H	-0.297402167607	3.561558117333	3.339533806705
C	-1.725701032147	2.377500331905	1.199934280476
C	-1.917449027260	3.647494616831	0.613140803203
C	-3.157518291776	3.889302142383	-0.009133211598
H	-3.310829684556	4.864694706643	-0.497096847465
C	-4.180767979989	2.928814432392	-0.048661437587
C	-3.956535190627	1.689633426896	0.581065754119
H	-4.752977599617	0.929979095151	0.589006934730
C	-2.737243991831	1.386222529561	1.206933704873
C	-0.858020586358	4.713512072911	0.648472534392
H	0.157719768586	4.281032003093	0.620482941689
H	-0.954042732119	5.336729274293	1.564556776174
H	-0.943586428826	5.379534339517	-0.227946216652
C	-5.474133422079	3.195487951870	-0.777051376637
H	-5.417306244684	2.830748606300	-1.825885180767
H	-5.704713248560	4.277733035629	-0.823129503307
H	-6.329191032082	2.678131986827	-0.297256318055
C	-2.537664943146	0.051125019124	1.877286771402
H	-1.806692371344	-0.599631929152	1.322958202509
H	-3.482661687186	-0.521261448356	1.925366996794
H	-2.117950260040	0.157055660440	2.898291853622
C	2.532158152140	0.124889787848	2.007890847028
C	3.806741345239	0.616885893539	1.645974742020
C	4.870167100785	-0.304882666149	1.604368477197

H	5.868000007207	0.062695038616	1.317792049724
C	4.694760582988	-1.669891583295	1.881978557562
C	3.405268919846	-2.119060373508	2.221678586129
H	3.242155440096	-3.187269021136	2.438506408478
C	2.310979192383	-1.242455040154	2.302453052106
C	4.022743919010	2.061763722062	1.282661700784
H	3.255056847652	2.419668272018	0.564226548833
H	5.025999396877	2.203484733478	0.838347228328
H	3.964053939294	2.726902322663	2.169321744527
C	5.851884865784	-2.634481125868	1.817182596006
H	6.728693072763	-2.186575023281	1.310577243811
H	5.576817275550	-3.561058552781	1.272405082280
H	6.176164686171	-2.945325636489	2.833268017260
C	0.944922364354	-1.752812372442	2.676979509727
H	0.488448320222	-1.156838587367	3.493551725548
H	0.981999189037	-2.814380749624	2.986979722025
H	0.247642483960	-1.663285603332	1.805922509575
N	-1.666552469373	-2.699736764323	-1.184909080537
N	0.403035137387	-2.399077719453	-1.733426442593
C	-0.596092332267	-1.879662169453	-0.947412504248
C	-1.345386412683	-3.701395845652	-2.103283993329
H	-2.077524105603	-4.445757389195	-2.428920327041
C	-0.034095870306	-3.504355172091	-2.453334739503
H	0.608992559386	-4.039117611505	-3.157194112786
C	1.692817042342	-1.766049288032	-1.772162097760
C	1.738347483479	-0.378454447372	-2.059140758906
C	2.993239385634	0.256474227632	-2.043975266810
H	3.019044047598	1.335995780230	-2.261561765777
C	4.175090906759	-0.458194712916	-1.778158130303
C	4.082370637353	-1.828634945591	-1.477488568675
H	4.996861348316	-2.389930630456	-1.227759160011
C	2.850294899116	-2.505483036141	-1.447158680343
C	0.508767693844	0.426571771822	-2.392651017215
H	-0.012559085927	0.859601485312	-1.485213514029

H	-0.236953485170	-0.148424273571	-2.972890373549
H	0.790128547185	1.347183203081	-2.935966347455
C	5.509406643166	0.239837265724	-1.826946809857
H	5.462727162099	1.226926458121	-1.325895300042
H	5.814880374140	0.433014394897	-2.877325880050
H	6.307941518948	-0.361977606673	-1.350577875545
C	2.780821106043	-3.953337873469	-1.029608036258
H	1.915426065019	-4.141419936805	-0.361993164294
H	3.701006768128	-4.243111984492	-0.486951275861
H	2.679956073858	-4.645546182431	-1.892516612951
C	-2.955740264520	-2.537700207586	-0.574743687294
C	-3.854880451277	-1.591054862014	-1.114874111282
C	-5.123003466112	-1.488258141195	-0.517766122531
H	-5.843563971849	-0.766854116775	-0.935442374473
C	-5.491933003358	-2.267071031936	0.596577606218
C	-4.549807052052	-3.170397315251	1.122007988534
H	-4.813649066856	-3.773602552450	2.005317832272
C	-3.272736370280	-3.325368756468	0.551969528133
C	-3.443188558769	-0.689422099063	-2.247323190533
H	-2.698548038266	0.055389130627	-1.892769578093
H	-4.308747991031	-0.132912649810	-2.652258842145
H	-2.963845243840	-1.250813888503	-3.074426919342
C	-6.857138584633	-2.109556719695	1.217193410454
H	-7.004136292012	-1.078719966758	1.602717009522
H	-7.008928530354	-2.810636427003	2.059960023680
H	-7.660331711720	-2.289485054879	0.473160127570
C	-2.255022478880	-4.269179571417	1.138864255612
H	-1.980141309570	-5.076335813378	0.428432251623
H	-2.635683940174	-4.742588620763	2.063087104127
H	-1.313909030026	-3.732936367280	1.382564858176
F	1.595517964591	2.947865043231	-0.544388308800
F	1.968589082270	3.157327478529	-2.822287656207
B	1.191101572181	3.653286810816	-1.741255926725
F	-0.174345440149	3.363424466364	-1.966214151006

F	1.394364061299	5.018946381380	-1.566184720928

95

8_opt ! 2-A (D2): E(UPBE-D3(BJ)/def2SVP) = -3352.64147072

E(UPBE0-D3BJ/pcSseg-2) = -3354.97982167

Ni	0.000000000000	0.000000000000	0.000000000000
N	0.956793992233	0.501717969951	-2.721083611550
N	-0.956793992233	-0.501717969951	-2.721083611550
C	0.000000000000	0.000000000000	-1.886032476358
C	0.608548394797	0.318855016385	-4.055857432392
H	1.248569540445	0.653221318662	-4.877793145355
C	-0.608548394797	-0.318855016385	-4.055857432392
H	-1.248569540445	-0.653221318662	-4.877793145355
C	-2.149150403153	-1.128091776671	-2.213625286815
C	-2.134056901303	-2.521903125810	-1.976469741636
C	-3.303530958073	-3.106038170513	-1.462457028179
H	-3.311025361203	-4.189859060513	-1.265510080461
C	-4.461415661612	-2.347079383132	-1.197439540229
C	-4.421667246710	-0.961381957785	-1.435692845614
H	-5.313016064628	-0.351214376520	-1.224077159540
C	-3.271037254916	-0.320408421846	-1.929085004508
C	-0.889389141591	-3.332798999460	-2.226773516632
H	-0.059361997569	-2.993104489658	-1.570041906568
H	-0.528035583700	-3.227492240856	-3.270220884911
H	-1.065743218737	-4.406712215316	-2.031785151162
C	-5.711887600646	-3.009775390489	-0.680370098171
H	-5.478262536514	-3.795612567663	0.065296843768
H	-6.266651930092	-3.505055174332	-1.505530757320
H	-6.401399282511	-2.279802559973	-0.213857185397
C	-3.212479568018	1.176241661154	-2.085116642588
H	-2.571610834417	1.618826586093	-1.291347191275
H	-4.217001052930	1.628022985463	-1.990721199251
H	-2.779008397856	1.481604463275	-3.058451936683
C	2.149150403153	1.128091776671	-2.213625286815

C	3.271037254916	0.320408421846	-1.929085004508
C	4.421667246710	0.961381957785	-1.435692845614
H	5.313016064628	0.351214376520	-1.224077159540
C	4.461415661612	2.347079383132	-1.197439540229
C	3.303530958073	3.106038170513	-1.462457028179
H	3.311025361203	4.189859060513	-1.265510080461
C	2.134056901303	2.521903125810	-1.976469741636
C	3.212479568018	-1.176241661154	-2.085116642588
H	2.571610834417	-1.618826586093	-1.291347191275
H	4.217001052930	-1.628022985463	-1.990721199251
H	2.779008397856	-1.481604463275	-3.058451936683
C	5.711887600646	3.009775390489	-0.680370098171
H	6.401399282511	2.279802559973	-0.213857185397
H	5.478262536514	3.795612567663	0.065296843768
H	6.266651930092	3.505055174332	-1.505530757320
C	0.889389141591	3.332798999460	-2.226773516632
H	0.528035583700	3.227492240856	-3.270220884911
H	1.065743218737	4.406712215316	-2.031785151162
H	0.059361997569	2.993104489658	-1.570041906568
N	-0.956793992233	0.501717969951	2.721083611550
N	0.956793992233	-0.501717969951	2.721083611550
C	-0.000000000000	-0.000000000000	1.886032476358
C	-0.608548394797	0.318855016385	4.055857432392
H	-1.248569540445	0.653221318662	4.877793145355
C	0.608548394797	-0.318855016385	4.055857432392
H	1.248569540445	-0.653221318662	4.877793145355
C	2.149150403153	-1.128091776671	2.213625286815
C	2.134056901303	-2.521903125810	1.976469741636
C	3.303530958073	-3.106038170513	1.462457028179
H	3.311025361203	-4.189859060513	1.265510080461
C	4.461415661612	-2.347079383132	1.197439540229
C	4.421667246710	-0.961381957785	1.435692845614
H	5.313016064628	-0.351214376520	1.224077159540
C	3.271037254916	-0.320408421846	1.929085004508

C	0.889389141591	-3.332798999460	2.226773516632
H	0.059361997569	-2.993104489658	1.570041906568
H	0.528035583700	-3.227492240856	3.270220884911
H	1.065743218737	-4.406712215316	2.031785151162
C	5.711887600646	-3.009775390489	0.680370098171
H	5.478262536514	-3.795612567663	-0.065296843768
H	6.266651930092	-3.505055174332	1.505530757320
H	6.401399282511	-2.279802559973	0.213857185397
C	3.212479568018	1.176241661154	2.085116642588
H	2.571610834417	1.618826586093	1.291347191275
H	4.217001052930	1.628022985463	1.990721199251
H	2.779008397856	1.481604463275	3.058451936683
C	-2.149150403153	1.128091776671	2.213625286815
C	-3.271037254916	0.320408421846	1.929085004508
C	-4.421667246710	0.961381957785	1.435692845614
H	-5.313016064628	0.351214376520	1.224077159540
C	-4.461415661612	2.347079383132	1.197439540229
C	-3.303530958073	3.106038170513	1.462457028179
H	-3.311025361203	4.189859060513	1.265510080461
C	-2.134056901303	2.521903125810	1.976469741636
C	-3.212479568018	-1.176241661154	2.085116642588
H	-2.571610834417	-1.618826586093	1.291347191275
H	-4.217001052930	-1.628022985463	1.990721199251
H	-2.779008397856	-1.481604463275	3.058451936683
C	-5.711887600646	3.009775390489	0.680370098171
H	-6.401399282511	2.279802559973	0.213857185397
H	-5.478262536514	3.795612567663	-0.065296843768
H	-6.266651930092	3.505055174332	1.505530757320
C	-0.889389141591	3.332798999460	2.226773516632
H	-0.528035583700	3.227492240856	3.270220884911
H	-1.065743218737	4.406712215316	2.031785151162
H	-0.059361997569	2.993104489658	1.570041906568

95

8_xrd_opt ! 2-A (C2): E(UPBE-D3(BJ)/def2SVP) = -3352.61633747

E(UPBE0-D3BJ/pcSseg-2) = -3354.97845315

Ni	-0.000000000000	-0.000000000000	0.150103213092
N	-0.929599347614	2.687969014260	-0.531351786908
N	0.946769479734	2.768797264508	0.480443213092
C	0.000000000000	1.891412544373	0.059008213092
C	-0.560708246007	4.024352699921	-0.473817786908
H	-1.124424523591	4.873129443382	-0.873592478737
C	0.618096477116	4.081506505794	0.163847213092
H	1.201721600085	4.978545518566	0.390496739428
C	2.156729094636	2.390894540363	1.161259213092
C	2.219328623191	2.533112783443	2.548302213092
C	3.418110117447	2.235255791407	3.172384213092
H	3.498311288223	2.368139463386	4.263671462213
C	4.518931900594	1.773250065765	2.468394213092
C	4.402605480400	1.602412956690	1.095255213092
H	5.259900650966	1.199416853081	0.532078494268
C	3.239224353866	1.922825148907	0.412201213092
C	1.018586779612	2.971905903709	3.340989213092
H	0.182155245475	2.265315603995	3.163853577959
H	0.718178107005	3.990017371655	3.021372508389
H	1.271755552176	2.984304938975	4.416369311894
C	5.824352892201	1.504456522419	3.169986213092
H	5.624634813035	1.037937428647	4.154044048661
H	6.367780009689	2.459934790372	3.318302061886
H	6.439494623868	0.820762733373	2.554719908491
C	3.156472643448	1.785947892268	-1.080319786908
H	2.483998900562	0.940559751948	-1.338542973395
H	4.164513304686	1.593371258305	-1.488241418435
H	2.754253665054	2.722924415921	-1.514435468925
C	-2.122195430147	2.200773114417	-1.176528786908
C	-3.285701412802	2.050770518665	-0.421558786908
C	-4.440479050683	1.644937786694	-1.087191786908
H	-5.370906642444	1.504197166420	-0.514418383191

C	-4.444061796794	1.410578425175	-2.458732786908
C	-3.268091797606	1.549136336929	-3.155530786908
H	-3.262834662097	1.339308131882	-4.237947091479
C	-2.080821341654	1.943836208584	-2.545033786908
C	-3.301881858526	2.301736611152	1.055621213092
H	-2.682872873241	1.535376088470	1.567681797407
H	-4.340760134090	2.243461660652	1.426905104906
H	-2.889891141068	3.309400529266	1.263682151450
C	-5.725096900371	1.014860152460	-3.155530786908
H	-6.420240974867	0.565954694779	-2.421181875464
H	-5.498654745455	0.277605336803	-3.949863022452
H	-6.192196844401	1.914174629871	-3.606307789940
C	-0.795705024485	2.050145395070	-3.318542786908
H	-0.383613016285	3.073285066422	-3.210260626895
H	-0.989847257198	1.840118477696	-4.385967615432
H	-0.065524254189	1.312977754523	-2.924266238769
N	0.929599347614	-2.687969014260	-0.531351786908
N	-0.946769479734	-2.768797264508	0.480443213092
C	-0.000000000000	-1.891412544373	0.059008213092
C	0.560708246007	-4.024352699921	-0.473817786908
H	1.124424523591	-4.873129443382	-0.873592478737
C	-0.618096477116	-4.081506505794	0.163847213092
H	-1.201721600085	-4.978545518566	0.390496739428
C	-2.156729094636	-2.390894540363	1.161259213092
C	-2.219328623191	-2.533112783443	2.548302213092
C	-3.418110117447	-2.235255791407	3.172384213092
H	-3.498311288223	-2.368139463386	4.263671462213
C	-4.518931900594	-1.773250065765	2.468394213092
C	-4.402605480400	-1.602412956690	1.095255213092
H	-5.259900650966	-1.199416853081	0.532078494268
C	-3.239224353866	-1.922825148907	0.412201213092
C	-1.018586779612	-2.971905903709	3.340989213092
H	-0.182155245475	-2.265315603995	3.163853577959
H	-0.718178107005	-3.990017371655	3.021372508389

H	-1.271755552176	-2.984304938975	4.416369311894
C	-5.824352892201	-1.504456522419	3.169986213092
H	-5.624634813035	-1.037937428647	4.154044048661
H	-6.367780009689	-2.459934790372	3.318302061886
H	-6.439494623868	-0.820762733373	2.554719908491
C	-3.156472643448	-1.785947892268	-1.080319786908
H	-2.483998900562	-0.940559751948	-1.338542973395
H	-4.164513304686	-1.593371258305	-1.488241418435
H	-2.754253665054	-2.722924415921	-1.514435468925
C	2.122195430147	-2.200773114417	-1.176528786908
C	3.285701412802	-2.050770518665	-0.421558786908
C	4.440479050683	-1.644937786694	-1.087191786908
H	5.370906642444	-1.504197166420	-0.514418383191
C	4.444061796794	-1.410578425175	-2.458732786908
C	3.268091797606	-1.549136336929	-3.155530786908
H	3.262834662097	-1.339308131882	-4.237947091479
C	2.080821341654	-1.943836208584	-2.545033786908
C	3.301881858526	-2.301736611152	1.055621213092
H	2.682872873241	-1.535376088470	1.567681797407
H	4.340760134090	-2.243461660652	1.426905104906
H	2.889891141068	-3.309400529266	1.263682151450
C	5.725096900371	-1.014860152460	-3.155530786908
H	6.420240974867	-0.565954694779	-2.421181875464
H	5.498654745455	-0.277605336803	-3.949863022452
H	6.192196844401	-1.914174629871	-3.606307789940
C	0.795705024485	-2.050145395070	-3.318542786908
H	0.383613016285	-3.073285066422	-3.210260626895
H	0.989847257198	-1.840118477696	-4.385967615432
H	0.065524254189	-1.312977754523	-2.924266238769

Mechanistic investigations:

106

11_opt, 2-A (C2): E(UPBE0-D3BJ/def2SVP) = -4079.56616133

G_corr(298K) = 0.761335

E(COSMO-PBE0-D3BJ/def2TZVP) = -4082.54819524

Ni	-0.000000000000	-0.000000000000	0.389973880765
N	-0.000000000000	2.958687144856	0.012811277350
N	-1.277615030362	2.378774968735	1.622038637951
C	-0.407610019859	1.861768906942	0.713151475742
C	-0.600435535708	4.116354339455	0.473512840427
H	-0.389972103995	5.088992642270	0.038915954270
C	-1.414423817763	3.750940068337	1.494740877529
H	-2.067065968238	4.332855874198	2.139172409994
C	-1.866292041945	1.603237582315	2.662225396454
C	-1.167598732346	1.490238859554	3.872355066912
C	-1.755601310838	0.748261290983	4.897127366711
H	-1.227482748564	0.655194670657	5.850420769364
C	-2.989407475276	0.112964711877	4.729490871031
C	-3.651564331890	0.252837038592	3.506737610476
H	-4.621295820914	-0.232152690671	3.362790039820
C	-3.115228727231	1.004259555633	2.457872948876
C	0.179024877120	2.128503555568	4.029535706542
H	0.156816520998	3.196651490447	3.764496945507
H	0.909766947426	1.644334310564	3.361600388963
H	0.544666661929	2.031329762057	5.059706694036
C	-3.568321975097	-0.734235677009	5.824666865442
H	-3.285059792730	-0.359403616776	6.818948678689
H	-3.201088563284	-1.772002836773	5.749248243985
H	-4.665694588441	-0.772807781192	5.770942108267
C	-3.836554555384	1.170253423729	1.155233665567
H	-4.775737579806	0.601284701216	1.150732833850
H	-3.218912548935	0.835431608444	0.309006375132
H	-4.078313747486	2.228596798016	0.967352755529
C	0.931997107924	2.908522722468	-1.066172684586
C	2.255486238429	2.536047165616	-0.797605703173
C	3.131222114377	2.417365926676	-1.877734935298
H	4.160664004360	2.102267831896	-1.686747319666
C	2.722024796955	2.659821156287	-3.189945853285

C	1.401931203480	3.062174156254	-3.408914825702
H	1.061298146707	3.247940833025	-4.431515179752
C	0.485259895056	3.190116636578	-2.365723889538
C	2.706739107928	2.229542459291	0.598293184358
H	3.797596922370	2.111585946775	0.642780540302
H	2.237966879915	1.297750575261	0.956254497053
H	2.409284199185	3.019645252716	1.305124313195
C	3.651375491468	2.431625185545	-4.345053192605
H	3.471336249434	3.148805587649	-5.159195158609
H	3.495409871609	1.419817908188	-4.753082059734
H	4.704809733183	2.509335091736	-4.040433342441
C	-0.938333095515	3.576634374817	-2.641729400086
H	-1.128505999260	4.636524477461	-2.405068030165
H	-1.640848005413	2.975015783091	-2.048750051929
H	-1.179711729049	3.425515657148	-3.702478054488
C	0.000000000000	0.000000000000	-1.603139056150
C	-1.115451854255	0.322609852834	-2.356435161405
C	-1.147722963889	0.345555837500	-3.746400577118
C	0.000000000000	0.000000000000	-4.452975391526
F	-2.272417354145	0.675318491653	-1.744076956484
F	-2.252234327000	0.689016648367	-4.416149883274
F	0.000000000000	0.000000000000	-5.784175357280
C	1.115451854255	-0.322609852834	-2.356435161405
C	1.147722963889	-0.345555837500	-3.746400577118
F	2.272417354145	-0.675318491653	-1.744076956484
F	2.252234327000	-0.689016648367	-4.416149883274
N	-0.000000000000	-2.958687144856	0.012811277350
N	1.277615030362	-2.378774968735	1.622038637951
C	0.407610019859	-1.861768906942	0.713151475742
C	0.600435535708	-4.116354339455	0.473512840427
H	0.389972103995	-5.088992642270	0.038915954270
C	1.414423817763	-3.750940068337	1.494740877529
H	2.067065968238	-4.332855874198	2.139172409994
C	1.866292041945	-1.603237582315	2.662225396454

C	1.167598732346	-1.490238859554	3.872355066912
C	1.755601310838	-0.748261290983	4.897127366711
H	1.227482748564	-0.655194670657	5.850420769364
C	2.989407475276	-0.112964711877	4.729490871031
C	3.651564331890	-0.252837038592	3.506737610476
H	4.621295820914	0.232152690671	3.362790039820
C	3.115228727231	-1.004259555633	2.457872948876
C	-0.179024877120	-2.128503555568	4.029535706542
H	-0.156816520998	-3.196651490447	3.764496945507
H	-0.909766947426	-1.644334310564	3.361600388963
H	-0.544666661929	-2.031329762057	5.059706694036
C	3.568321975097	0.734235677009	5.824666865442
H	3.285059792730	0.359403616776	6.818948678689
H	3.201088563284	1.772002836773	5.749248243985
H	4.665694588441	0.772807781192	5.770942108267
C	3.836554555384	-1.170253423729	1.155233665567
H	4.775737579806	-0.601284701216	1.150732833850
H	3.218912548935	-0.835431608444	0.309006375132
H	4.078313747486	-2.228596798016	0.967352755529
C	-0.931997107924	-2.908522722468	-1.066172684586
C	-2.255486238429	-2.536047165616	-0.797605703173
C	-3.131222114377	-2.417365926676	-1.877734935298
H	-4.160664004360	-2.102267831896	-1.686747319666
C	-2.722024796955	-2.659821156287	-3.189945853285
C	-1.401931203480	-3.062174156254	-3.408914825702
H	-1.061298146707	-3.247940833025	-4.431515179752
C	-0.485259895056	-3.190116636578	-2.365723889538
C	-2.706739107928	-2.229542459291	0.598293184358
H	-3.797596922370	-2.111585946775	0.642780540302
H	-2.237966879915	-1.297750575261	0.956254497053
H	-2.409284199185	-3.019645252716	1.305124313195
C	-3.651375491468	-2.431625185545	-4.345053192605
H	-3.471336249434	-3.148805587649	-5.159195158609
H	-3.495409871609	-1.419817908188	-4.753082059734

H	-4.704809733183	-2.509335091736	-4.040433342441
C	0.938333095515	-3.576634374817	-2.641729400086
H	1.128505999260	-4.636524477461	-2.405068030165
H	1.640848005413	-2.975015783091	-2.048750051929
H	1.179711729049	-3.425515657148	-3.702478054488

96

12_opt, 2-A (C2): E(UPBE0-D3BJ/def2SVP) = -3452.70430100

G_corr(298K) = 0.721115

E(COSMO-PBE0-D3BJ/def2TZVP) = -3454.97056859

Ni	0.000000000000	0.000000000000	0.111170054569
N	0.635390425472	-2.913062509075	0.101393962979
N	-1.398138216216	-2.504311642279	-0.396699997435
C	-0.216293087780	-1.885877344321	-0.145203167735
C	-0.000000000000	-4.140576591977	0.012126333861
H	0.525645392674	-5.075489780242	0.184148826611
C	-1.293906152324	-3.881173733257	-0.303818855922
H	-2.142186938302	-4.540280170077	-0.464873952350
C	-2.608016134463	-1.796366831661	-0.660422160966
C	-2.993203750294	-1.597148033368	-1.992594370934
C	-4.207480116848	-0.953891684135	-2.232064486552
H	-4.525131099837	-0.791474761080	-3.266388701950
C	-5.023846191744	-0.518971203912	-1.183421202586
C	-4.579364120854	-0.699509289086	0.127591895207
H	-5.189247643469	-0.331959172867	0.956368003318
C	-3.367492049804	-1.329999300243	0.420869955191
C	-2.094482726023	-2.029969127829	-3.112057813932
H	-1.910945848865	-3.115822212185	-3.096013889037
H	-1.109879702342	-1.542355528156	-3.021076942239
H	-2.524539653568	-1.769610560292	-4.088545537579
C	-6.356422839951	0.110142346467	-1.469386598690
H	-6.295041262063	0.822971986308	-2.305940558542
H	-6.748179903281	0.642868292677	-0.592274964296
H	-7.098885463707	-0.654683983054	-1.751840188482

C	-2.871831740487	-1.473493578150	1.826929212532
H	-3.613957236495	-1.093514986910	2.541721190817
H	-1.925462942948	-0.916767914781	1.969681322911
H	-2.659666915096	-2.526401320661	2.073050978529
C	2.007263987686	-2.765142489905	0.469323309309
C	2.976895541683	-2.739600768915	-0.539176820012
C	4.318287326335	-2.710469346503	-0.154040085121
H	5.088547523572	-2.710694726724	-0.929335561863
C	4.693966040493	-2.674984601806	1.190269814281
C	3.691192637350	-2.649368110462	2.163390703437
H	3.968537827599	-2.600866105736	3.220538611167
C	2.335183042314	-2.696974162790	1.831577701669
C	2.573735505230	-2.696957443131	-1.981915154327
H	3.451694015514	-2.746138303831	-2.639830427987
H	2.038782318641	-1.755351614916	-2.189101940170
H	1.890892901720	-3.519406872411	-2.245834551470
C	6.141337286854	-2.628067728459	1.587596645978
H	6.342789766692	-3.278170493624	2.452121241056
H	6.440558294719	-1.606845484378	1.879093094240
H	6.797842325844	-2.942300549072	0.763768145374
C	1.262297865436	-2.623492298716	2.870933560680
H	0.534271267789	-3.443129590617	2.763965993798
H	0.710756406913	-1.667964489356	2.746953205875
H	1.690418724030	-2.670441113296	3.881451183498
F	0.000000000000	0.000000000000	2.068868089732
N	-0.635390425472	2.913062509075	0.101393962979
N	1.398138216216	2.504311642279	-0.396699997435
C	0.216293087780	1.885877344321	-0.145203167735
C	0.000000000000	4.140576591977	0.012126333861
H	-0.525645392674	5.075489780242	0.184148826611
C	1.293906152324	3.881173733257	-0.303818855922
H	2.142186938302	4.540280170077	-0.464873952350
C	2.608016134463	1.796366831661	-0.660422160966
C	2.993203750294	1.597148033368	-1.992594370934

C	4.207480116848	0.953891684135	-2.232064486552
H	4.525131099837	0.791474761080	-3.266388701950
C	5.023846191744	0.518971203912	-1.183421202586
C	4.579364120854	0.699509289086	0.127591895207
H	5.189247643469	0.331959172867	0.956368003318
C	3.367492049804	1.329999300243	0.420869955191
C	2.094482726023	2.029969127829	-3.112057813932
H	1.910945848865	3.115822212185	-3.096013889037
H	1.109879702342	1.542355528156	-3.021076942239
H	2.524539653568	1.769610560292	-4.088545537579
C	6.356422839951	-0.110142346467	-1.469386598690
H	6.295041262063	-0.822971986308	-2.305940558542
H	6.748179903281	-0.642868292677	-0.592274964296
H	7.098885463707	0.654683983054	-1.751840188482
C	2.871831740487	1.473493578150	1.826929212532
H	3.613957236495	1.093514986910	2.541721190817
H	1.925462942948	0.916767914781	1.969681322911
H	2.659666915096	2.526401320661	2.073050978529
C	-2.007263987686	2.765142489905	0.469323309309
C	-2.976895541683	2.739600768915	-0.539176820012
C	-4.318287326335	2.710469346503	-0.154040085121
H	-5.088547523572	2.710694726724	-0.929335561863
C	-4.693966040493	2.674984601806	1.190269814281
C	-3.691192637350	2.649368110462	2.163390703437
H	-3.968537827599	2.600866105736	3.220538611167
C	-2.335183042314	2.696974162790	1.831577701669
C	-2.573735505230	2.696957443131	-1.981915154327
H	-3.451694015514	2.746138303831	-2.639830427987
H	-2.038782318641	1.755351614916	-2.189101940170
H	-1.890892901720	3.519406872411	-2.245834551470
C	-6.141337286854	2.628067728459	1.587596645978
H	-6.342789766692	3.278170493624	2.452121241056
H	-6.440558294719	1.606845484378	1.879093094240
H	-6.797842325844	2.942300549072	0.763768145374

C	-1.262297865436	2.623492298716	2.870933560680
H	-0.534271267789	3.443129590617	2.763965993798
H	-0.710756406913	1.667964489356	2.746953205875
H	-1.690418724030	2.670441113296	3.881451183498

117

15_opt, 1-A (C2): E(RPBE0-D3BJ/def2SVP) = -4806.11009011

G_corr(298K) = 0.806949

E(COSMO-PBE0-D3BJ/def2TZVP) = -4809.93612329

Ni	-0.000000000000	-0.000000000000	0.000009331280
N	-2.859365381881	1.067721334988	-0.017947375132
N	-2.859273457123	-1.068052626985	0.017290793624
C	-2.003183388390	-0.000141138278	-0.000131644886
C	-4.185014988821	0.674225647766	-0.011691666772
H	-4.992014095587	1.400450156922	-0.024021370606
C	-4.184962963395	-0.674681053501	0.010467650531
H	-4.991893455487	-1.400985466382	0.022456458805
C	0.000000000000	0.000000000000	-1.955119367373
C	0.005474796885	1.164872813021	-2.708558514170
C	0.007229565670	1.196391440709	-4.100900006825
C	0.000000000000	0.000000000000	-4.811092230161
F	0.003521930292	2.355628291930	-2.097808713511
F	0.008631146611	2.357928907146	-4.754728632507
F	0.000000000000	0.000000000000	-6.137306002857
C	-0.005474796885	-1.164872813021	-2.708558514170
C	-0.007229565670	-1.196391440709	-4.100900006825
F	-0.003521930292	-2.355628291930	-2.097808713511
F	-0.008631146611	-2.357928907146	-4.754728632507
N	2.859365381881	-1.067721334988	-0.017947375132
N	2.859273457123	1.068052626985	0.017290793624
C	2.003183388390	0.000141138278	-0.000131644886
C	4.185014988821	-0.674225647766	-0.011691666772
H	4.992014095587	-1.400450156922	-0.024021370606
C	4.184962963395	0.674681053501	0.010467650531

H	4.991893455487	1.400985466382	0.022456458805
C	2.563828984733	2.469501544645	0.047852778059
C	2.528003432162	3.123223818466	1.288696903904
C	2.506420260082	3.176152137872	-1.159878264099
C	2.280102743412	4.494604278660	1.295955931179
C	2.253879606078	4.548744020951	-1.100790765018
C	2.119345637550	5.221244423350	0.113619453906
H	2.218000513465	5.010613134648	2.258384819696
H	2.172166616910	5.106975513756	-2.037597803716
C	2.564039584443	-2.469201275728	-0.048090214544
C	2.506728994147	-3.175488582351	1.159861678994
C	2.528133864861	-3.123293214459	-1.288731920900
C	2.254173030971	-4.548087002498	1.101208782960
C	2.280201151115	-4.494674068732	-1.295563360775
C	2.119537053379	-5.220951199786	-0.112997040180
H	2.172509796617	-5.106034581254	2.038188579597
H	2.218003997031	-5.010963776436	-2.257834705661
C	-2.564039584443	2.469201275728	-0.048090214544
C	-2.528133864861	3.123293214459	-1.288731920900
C	-2.506728994147	3.175488582351	1.159861678994
C	-2.280201151115	4.494674068732	-1.295563360775
C	-2.254173030971	4.548087002498	1.101208782960
C	-2.119537053379	5.220951199786	-0.112997040180
H	-2.218003997031	5.010963776436	-2.257834705661
H	-2.172509796617	5.106034581254	2.038188579597
C	-2.563828984733	-2.469501544645	0.047852778059
C	-2.528003432162	-3.123223818466	1.288696903904
C	-2.506420260082	-3.176152137872	-1.159878264099
C	-2.280102743412	-4.494604278660	1.295955931179
C	-2.253879606078	-4.548744020951	-1.100790765018
C	-2.119345637550	-5.221244423350	0.113619453906
H	-2.218000513465	-5.010613134648	2.258384819696
H	-2.172166616910	-5.106975513756	-2.037597803716
C	-2.834428499911	2.404791309769	-2.568643160935

H	-3.910668559507	2.481624420275	-2.797668808626
H	-2.283085684314	2.847990932386	-3.408304120671
H	-2.586660181894	1.337440933036	-2.518950702426
C	-1.865203343200	6.699329653112	-0.160138226981
H	-2.754585825190	7.241706700115	-0.520509822747
H	-1.611015481783	7.097958147399	0.831938386113
H	-1.039836326290	6.939065746941	-0.846786819657
C	-2.804393434975	2.518782781847	2.474370376257
H	-2.231790543192	2.985576567938	3.286376466883
H	-3.875506674603	2.626442603970	2.714952080803
H	-2.577578961623	1.445675344188	2.466357810854
C	2.803929485340	2.519906745971	-2.474662022849
H	2.577962617729	1.446619123326	-2.466728236504
H	2.230538435803	2.986394724038	-3.286287259786
H	3.874824757363	2.628504073828	-2.715783996754
C	1.864847071588	6.699575366684	0.161275346009
H	2.753497705213	7.241773106284	0.523692529780
H	1.612406961287	7.098855298083	-0.830984676472
H	1.038188203610	6.938765332244	0.846563626081
C	2.834406989560	2.404444574606	2.568429505425
H	3.910495191276	2.482153837190	2.797868951942
H	2.282408204205	2.846882242795	3.408061111699
H	2.587586454219	1.336889054013	2.518289659608
C	1.865203343200	-6.699329653112	-0.160138226981
H	1.611015481783	-7.097958147399	0.831938386113
H	1.039836326290	-6.939065746941	-0.846786819657
H	2.754585825190	-7.241706700115	-0.520509822747
C	2.834428499911	-2.404791309769	-2.568643160935
H	2.283085684314	-2.847990932386	-3.408304120671
H	2.586660181894	-1.337440933036	-2.518950702426
H	3.910668559507	-2.481624420275	-2.797668808626
C	2.804393434975	-2.518782781847	2.474370376257
H	3.875506674603	-2.626442603970	2.714952080803
H	2.577578961623	-1.445675344188	2.466357810854

H	2.231790543192	-2.985576567938	3.286376466883
C	-1.864847071588	-6.699575366684	0.161275346009
H	-1.038188203610	-6.938765332244	0.846563626081
H	-2.753497705213	-7.241773106284	0.523692529780
H	-1.612406961287	-7.098855298083	-0.830984676472
C	-2.803929485340	-2.519906745971	-2.474662022849
H	-3.874824757363	-2.628504073828	-2.715783996754
H	-2.577962617729	-1.446619123326	-2.466728236504
H	-2.230538435803	-2.986394724038	-3.286287259786
C	-2.834406989560	-2.404444574606	2.568429505425
H	-2.282408204205	-2.846882242795	3.408061111699
H	-2.587586454219	-1.336889054013	2.518289659608
H	-3.910495191276	-2.482153837190	2.797868951942
C	-0.000000000000	-0.000000000000	1.955110255179
C	0.005530904259	-1.164875351219	2.708557425057
C	0.007262746789	-1.196382403695	4.100898093315
F	0.008734012354	-2.357923959989	4.754727535041
F	-0.000000000000	-0.000000000000	6.137311953083
C	-0.000000000000	-0.000000000000	4.811095775655
C	-0.007262746789	1.196382403695	4.100898093315
F	-0.008734012354	2.357923959989	4.754727535041
F	-0.003777147288	2.355643145875	2.097840977479
C	-0.005530904259	1.164875351219	2.708557425057
F	0.003777147288	-2.355643145875	2.097840977479

55

1ipr_opt, 1-A (C2): E(RPBE0-D3BJ/def2SVP) = -2430.37665016

G_corr(298K) = 0.422587

E(COSMO-PBE0-D3BJ/def2TZVP) = -2431.58369186

Ni	0.000000000000	0.000000000000	-0.087971068025
N	-0.198665032840	2.756092386435	-0.996457457146
N	0.504140104538	2.606037249530	1.023706798384
C	0.108610879523	1.836737437239	-0.034554549672
C	-0.000000000000	4.047291209069	-0.553243481324

H	-0.181907009945	4.924771194091	-1.168136092458
C	0.440382474020	3.951873308385	0.730976454055
H	0.707847640694	4.731137612633	1.439796776268
N	0.198665032840	-2.756092386435	-0.996457457146
N	-0.504140104538	-2.606037249530	1.023706798384
C	-0.108610879523	-1.836737437239	-0.034554549672
C	0.000000000000	-4.047291209069	-0.553243481324
H	0.181907009945	-4.924771194091	-1.168136092458
C	-0.440382474020	-3.951873308385	0.730976454055
H	-0.707847640694	-4.731137612633	1.439796776268
C	0.878126147446	1.989433036260	2.287801991597
H	3.002466417347	2.220675437328	1.938428159061
C	-0.191629671596	2.212502976978	3.344345255047
C	2.265626351476	2.419458865235	2.730401499474
H	2.302986834556	3.493441642331	2.976482325058
H	-0.290079020066	3.278968096093	3.605052996603
H	2.566699329682	1.863207158595	3.630626904801
H	-1.164908971520	1.856201818796	2.974862640077
H	0.055697041765	1.661181947027	4.264462516139
H	0.891836860575	0.915524240932	2.016203909294
C	-0.624071221387	2.335461751728	-2.322981823128
H	-0.745755116657	1.243834181198	-2.189558523587
C	0.469172117458	2.584688520673	-3.349678240964
C	-1.960312610404	2.951619990003	-2.700423104283
H	-2.717508743660	2.731442127953	-1.933730160455
H	-1.889306790559	4.045950594820	-2.812569088326
H	-2.310235208169	2.542778022143	-3.659988320445
H	0.678813754639	3.660494147417	-3.465846566274
H	0.172168415486	2.189942672242	-4.333150653178
H	1.397013251162	2.082476759241	-3.037951263710
C	0.624071221387	-2.335461751728	-2.322981823128
H	0.745755116657	-1.243834181198	-2.189558523587
C	-0.469172117458	-2.584688520673	-3.349678240964
C	1.960312610404	-2.951619990003	-2.700423104283

H	2.717508743660	-2.731442127953	-1.933730160455
H	1.889306790559	-4.045950594820	-2.812569088326
H	2.310235208169	-2.542778022143	-3.659988320445
H	-0.678813754639	-3.660494147417	-3.465846566274
H	-0.172168415486	-2.189942672242	-4.333150653178
H	-1.397013251162	-2.082476759241	-3.037951263710
C	-0.878126147446	-1.989433036260	2.287801991597
H	-0.891836860575	-0.915524240932	2.016203909294
C	0.191629671596	-2.212502976978	3.344345255047
C	-2.265626351476	-2.419458865235	2.730401499474
H	-3.002466417347	-2.220675437328	1.938428159061
H	-2.302986834556	-3.493441642331	2.976482325058
H	-2.566699329682	-1.863207158595	3.630626904801
H	0.290079020066	-3.278968096093	3.605052996603
H	-0.055697041765	-1.661181947027	4.264462516139
H	1.164908971520	-1.856201818796	2.974862640077

95

1_opt, 1-A (C2): E(RPBE0-D3BJ/def2SVP) = -3352.95732260

G_corr(298K) = 0.712611

E(COSMO-PBE0-D3BJ/def2TZVP) = -3355.10776634

Ni	-0.000000000000	-0.000000000000	0.039881156756
N	-0.451331283814	2.599318201651	-1.045258683389
N	0.306309300121	2.755365745296	0.952420081206
C	-0.000000000000	1.829494922155	-0.005825111479
C	-0.432783207498	3.950048727104	-0.737785015705
H	-0.774598375459	4.710924123764	-1.433817978997
C	0.048198352376	4.049673693191	0.526898630705
H	0.214331924724	4.914747161428	1.162701632978
N	-0.306309300121	-2.755365745296	0.952420081206
N	0.451331283814	-2.599318201651	-1.045258683389
C	-0.000000000000	-1.829494922155	-0.005825111479
C	-0.048198352376	-4.049673693191	0.526898630705
H	-0.214331924724	-4.914747161428	1.162701632978

C	0.432783207498	-3.950048727104	-0.737785015705
H	0.774598375459	-4.710924123764	-1.433817978997
C	0.891780405046	-2.035342118162	-2.276705353502
C	2.110211352013	-1.337365629393	-2.316988055552
C	0.084355220567	-2.177239796471	-3.412344424524
C	2.516928662197	-0.808081353591	-3.542018267885
C	0.545526362758	-1.648074427960	-4.619239450407
C	1.759004655273	-0.965319146979	-4.705954946666
H	3.458067482474	-0.251701211773	-3.585277760735
H	-0.079280730111	-1.748199410328	-5.510969878211
C	-0.790572086398	-2.401992904664	2.243479399993
C	0.107416213810	-1.890222631623	3.193381795964
C	-2.150413232996	-2.578651677177	2.528788006323
C	-0.384577930277	-1.615707886374	4.469379892768
C	-2.599080045340	-2.270921690271	3.815445040055
C	-1.729598149935	-1.800015559035	4.802038910061
H	0.308135991377	-1.232280103189	5.223023963264
H	-3.660049616026	-2.398783090743	4.049470635049
C	-0.891780405046	2.035342118162	-2.276705353502
C	-2.110211352013	1.337365629393	-2.316988055552
C	-0.084355220567	2.177239796471	-3.412344424524
C	-2.516928662197	0.808081353591	-3.542018267885
C	-0.545526362758	1.648074427960	-4.619239450407
C	-1.759004655273	0.965319146979	-4.705954946666
H	-3.458067482474	0.251701211773	-3.585277760735
H	0.079280730111	1.748199410328	-5.510969878211
C	0.790572086398	2.401992904664	2.243479399993
C	2.150413232996	2.578651677177	2.528788006323
C	-0.107416213810	1.890222631623	3.193381795964
C	2.599080045340	2.270921690271	3.815445040055
C	0.384577930277	1.615707886374	4.469379892768
C	1.729598149935	1.800015559035	4.802038910061
H	3.660049616026	2.398783090743	4.049470635049
H	-0.308135991377	1.232280103189	5.223023963264

C	-2.893172391449	1.101882913906	-1.064935066357
H	-3.102426996529	2.039122698974	-0.526367282127
H	-3.841286507285	0.587507805979	-1.272087675385
H	-2.268904431840	0.481898085382	-0.384851927739
C	-2.239676303109	0.403749945957	-6.012422268610
H	-2.627346612166	-0.619537832658	-5.890726045976
H	-3.059520014592	1.010983982726	-6.430763991570
H	-1.434019303552	0.376601516748	-6.760103425844
C	1.267933652887	2.816662996727	-3.310277152245
H	1.823753608048	2.707977998193	-4.251452247802
H	1.209754792687	3.890248637911	-3.071426533223
H	1.849683797682	2.340185835822	-2.505150987268
C	-1.267933652887	-2.816662996727	-3.310277152245
H	-1.823753608048	-2.707977998193	-4.251452247802
H	-1.209754792687	-3.890248637911	-3.071426533223
H	-1.849683797682	-2.340185835822	-2.505150987268
C	2.239676303109	-0.403749945957	-6.012422268610
H	2.627346612166	0.619537832658	-5.890726045976
H	3.059520014592	-1.010983982726	-6.430763991570
H	1.434019303552	-0.376601516748	-6.760103425844
C	2.893172391449	-1.101882913906	-1.064935066357
H	3.102426996529	-2.039122698974	-0.526367282127
H	3.841286507285	-0.587507805979	-1.272087675385
H	2.268904431840	-0.481898085382	-0.384851927739
C	-2.229320816907	-1.447565961893	6.173495285728
H	-2.358800432403	-0.356621746065	6.278055807777
H	-3.202074610012	-1.914297628349	6.383815699838
H	-1.521322046695	-1.764260261632	6.954059606733
C	-3.093810688090	-3.037909453942	1.457389070049
H	-4.135240460931	-2.995965238743	1.803934406615
H	-2.993794592405	-2.400075542751	0.564366637827
H	-2.883555454835	-4.069738100975	1.134149931971
C	1.518746006485	-1.576091329056	2.810182954893
H	2.044553441781	-2.457542059451	2.410636539590

H	1.492819152957	-0.824551412392	1.996281855233
H	2.085094902000	-1.169407687717	3.658129797066
C	2.229320816907	1.447565961893	6.173495285728
H	2.358800432403	0.356621746065	6.278055807777
H	3.202074610012	1.914297628349	6.383815699838
H	1.521322046695	1.764260261632	6.954059606733
C	-1.518746006485	1.576091329056	2.810182954893
H	-2.044553441781	2.457542059451	2.410636539590
H	-1.492819152957	0.824551412392	1.996281855233
H	-2.085094902000	1.169407687717	3.658129797066
C	3.093810688090	3.037909453942	1.457389070049
H	2.993794592405	2.400075542751	0.564366637827
H	2.883555454835	4.069738100975	1.134149931971
H	4.135240460931	2.995965238743	1.803934406615

67

3ipr_opt, 1-A (C1): E(RPBE0-D3BJ/def2SVP) = -3256.70746852

G_corr(298K) = 0.469878

E(COSMO-PBE0-D3BJ/def2TZVP) = -3258.86887730

Ni	0.410328800331	-0.615219350931	0.338071962370
N	-2.231208321158	-1.156686965376	1.573335446154
N	-1.693908225237	-2.607437446868	0.092457719840
C	-1.265979065698	-1.471766247227	0.682206157814
C	-3.250311862583	-2.088103596707	1.549836649712
H	-4.125991075737	-2.024531046279	2.189130369416
C	-2.906534160538	-3.009292323781	0.611860816863
H	-3.426245816778	-3.902447811386	0.278232055275
C	-0.473900336491	0.911171512840	-0.366138829417
C	-1.140818870843	0.839169916707	-1.583869253417
C	-1.823935442800	1.906900970084	-2.161174619548
C	-1.848661825486	3.131611020476	-1.497291320247
F	-1.151553648960	-0.317245615381	-2.275734773457
F	-2.444443479593	1.778931973158	-3.327677826699
F	-2.490801728399	4.165784891591	-2.023220903943

C	-0.520668526206	2.155708544012	0.249044410237
C	-1.193876782402	3.257200906686	-0.274760055653
F	0.075708453883	2.342101335650	1.443292274572
F	-1.219052115248	4.417624376513	0.370023356770
N	2.811594467416	0.116064096375	-1.156532925698
N	2.958062868174	0.704375158284	0.899198183147
C	2.116252496793	0.162564690652	-0.001653451435
C	4.089854695384	0.608186485951	-0.980202860383
H	4.817793679639	0.669816924894	-1.782932900721
C	4.181645298105	0.983553900480	0.323191688840
H	5.004372304271	1.434391098422	0.869509154754
C	-0.919041929579	-3.321356958896	-0.923605727879
H	-0.137590829166	-2.603468860259	-1.205940242506
C	-1.774128661009	-3.623043677524	-2.142101837691
C	-0.241076358608	-4.536679694464	-0.314702023376
H	0.414764324898	-4.193949346088	0.497836679273
H	-0.977645477209	-5.261657875130	0.068077969734
H	0.372978590271	-5.043965845763	-1.073975913391
H	-2.569490469604	-4.352131416933	-1.919149967790
H	-1.146940836758	-4.056971634025	-2.934424155604
H	-2.234281857376	-2.703529507558	-2.530876588187
C	-2.182329406069	0.024299456820	2.431097016043
H	-1.223878666411	0.496448603024	2.177940585580
C	-2.156192853071	-0.371842311753	3.898547277604
C	-3.301721413944	0.992562369446	2.082165780745
H	-3.282036536248	1.231397665260	1.009060648974
H	-4.291780758425	0.577641040458	2.329988594026
H	-3.177425399132	1.930068654910	2.643408294874
H	-3.093039861850	-0.863655825449	4.205209510905
H	-2.027555662295	0.521623246267	4.526752634911
H	-1.322493976733	-1.060642248871	4.098460403803
C	2.303461351402	-0.607958971381	-2.323141028817
H	1.213847530957	-0.462187720618	-2.286130428734
C	2.596710649358	-2.093354555064	-2.150001513219

C	2.826429649310	-0.023564917956	-3.621444979254
H	2.622429993189	1.055503808769	-3.685681423699
H	3.909422739796	-0.185972054284	-3.740387846454
H	2.329409810746	-0.515639624848	-4.469456128292
H	3.679164580510	-2.288797985765	-2.216563909876
H	2.093693308043	-2.679078037404	-2.934482715991
H	2.240830496300	-2.419165118559	-1.158779746092
C	2.639792350545	0.756273576893	2.327006359179
H	1.541253093653	0.732310952514	2.350815319256
C	3.106707645617	2.057569053065	2.951926130635
C	3.166524606229	-0.497769667205	3.008496511476
H	2.753237993325	-1.380358951705	2.495541102579
H	4.267418852414	-0.534896412249	2.973956982089
H	2.857727798131	-0.515129190201	4.064707483509
H	4.205228430361	2.139808678199	2.964738255029
H	2.765065575339	2.108068517653	3.995717747587
H	2.690996865528	2.921013772758	2.412853326411
F	1.306427167010	-2.141665340810	0.903303777870

107

3_opt, 1-A (C1): E(RPBE0-D3BJ/def2SVP) = -4179.28932741

G_corr(298K) = 0.764620

E(COSMO-PBE0-D3BJ/def2TZVP) = -4182.38966793

Ni	0.000039025455	0.001624286137	-0.688392995659
N	0.520567914350	-2.881010895992	-0.558276714867
N	-1.534426537477	-2.588333636569	-1.065976295964
C	-0.408008615039	-1.907411889662	-0.730392575543
C	-0.000666635381	-4.138434482993	-0.798577323910
H	0.605023185169	-5.036555950718	-0.722349417436
C	-1.303403468425	-3.952939971285	-1.118764884142
H	-2.090786035143	-4.654630007399	-1.377385738409
C	-0.000060428570	-0.002753648047	1.210352245779
C	1.172249666606	0.056076840764	1.954223225756
C	1.200460131995	0.053023477427	3.346953624463

C	-0.000389949937	-0.009461654399	4.050855384552
F	2.353588113415	0.116016069923	1.333177728729
F	2.354383788699	0.097319161438	4.007182126103
F	-0.000541480551	-0.012597268928	5.378009176652
C	-1.172538622280	-0.065081457508	1.953673415857
C	-1.201080128182	-0.068609642518	3.346391382114
F	-2.353720823597	-0.122069076111	1.332055834125
F	-2.355160428511	-0.116036523691	4.006130441116
N	-0.520444263424	2.883644332300	-0.544916675049
N	1.534553921437	2.593242446662	-1.053922227771
C	0.408095340172	1.910818338909	-0.721552885419
C	0.000856163014	4.142151170738	-0.779327134649
H	-0.604800315535	5.039932742412	-0.698935842267
C	1.303602739824	3.958095011315	-1.100306041029
H	2.091029899175	4.660949260544	-1.355604390651
F	0.000065909715	0.006017870730	-2.582862392315
C	2.848828547035	2.055713660311	-1.236585350908
C	3.227872405045	1.584789346534	-2.500919885303
C	3.745736418713	2.119802963854	-0.159555871073
C	4.534547205803	1.109411370049	-2.645995593446
C	5.038080507996	1.633957606459	-0.359674955930
C	5.449108412796	1.118641228990	-1.591291399556
H	4.846701012369	0.731583411992	-3.623839691627
H	5.745513925240	1.665939269588	0.474335961721
C	-1.857156200044	2.653578316413	-0.095897689951
C	-2.823330895271	2.231413871869	-1.018044224443
C	-2.147484532380	2.876329057066	1.256596162538
C	-4.111974961434	2.003076072620	-0.534712598585
C	-3.453418875568	2.638341299295	1.686713894300
C	-4.442614468658	2.189764715062	0.809714347591
H	-4.877237806726	1.661270059159	-1.235182972123
H	-3.693804458218	2.777583094428	2.744328990738
C	1.857244569016	-2.653004442593	-0.108116997343
C	2.147494023416	-2.882085352975	1.243331845480

C	2.823465582430	-2.226524995405	-1.028226189432
C	3.453408491855	-2.646133461263	1.674630454222
C	4.112105585025	-2.000541585140	-0.543775666956
C	4.442674403175	-2.193540537910	0.799781172888
H	3.693733331904	-2.790315125904	2.731595958614
H	4.877422638020	-1.655541270070	-1.242618662284
C	-2.848711055312	-2.050013768564	-1.246209150764
C	-3.227612271903	-1.573010459089	-2.508304049779
C	-3.745745430552	-2.119321734388	-0.169608670088
C	-4.534268880815	-1.096935364636	-2.651237415076
C	-5.038063425153	-1.632504198254	-0.367526718820
C	-5.448942612186	-1.111232977128	-1.596685076998
H	-4.846303678609	-0.714383968783	-3.627281552443
H	-5.745588848138	-1.668485394053	0.466242755676
C	1.082266557686	-3.331984277594	2.199675877565
H	0.761493975246	-4.367455923068	2.001603848035
H	1.443697547126	-3.283709894138	3.235395620083
H	0.186856380597	-2.696133515975	2.120718718730
C	5.820388018110	-1.871309355979	1.297834916138
H	6.032813781590	-2.363650926606	2.257227355689
H	6.593565459696	-2.174617260295	0.576067827797
H	5.923213931634	-0.784263892071	1.452576738395
C	2.479162626580	-2.034872125025	-2.473932348525
H	3.357029072030	-1.694238487007	-3.038123856806
H	2.126209103478	-2.978338552243	-2.921967228045
H	1.669316202740	-1.297434267405	-2.604148346016
C	3.364355026918	2.734267353892	1.155797895995
H	2.283289278446	2.688409672135	1.336255532140
H	3.863672252686	2.216860712694	1.985694617004
H	3.662251103330	3.795476134217	1.189433737880
C	6.847398610054	0.602380733550	-1.771070346079
H	6.996241351346	0.178411267277	-2.773931193861
H	7.589236864706	1.404856928679	-1.631072922596
H	7.080041341637	-0.180720658778	-1.032342605576

C	2.276892014587	1.623086228925	-3.655614352821
H	1.974555890423	2.660373943052	-3.875816317848
H	2.744373751616	1.207527123331	-4.558532882967
H	1.358945293177	1.054432642320	-3.423847368303
C	-5.820300331599	1.865041457765	1.306217150664
H	-6.593468853199	2.171158094809	0.585630095824
H	-5.922805960644	0.777319022691	1.456358691480
H	-6.032999701620	2.353260625846	2.267654838449
C	-1.082301553679	3.321681391850	2.215118560872
H	-1.443932067515	3.268892902491	3.250547684597
H	-0.187065175932	2.685914597403	2.133501887632
H	-0.761187656132	4.357929737686	2.021716749288
C	-2.478951476721	2.046588103282	-2.464621816382
H	-2.125953788161	2.992160667698	-2.908160382697
H	-1.669113662652	1.309753139400	-2.598285674716
H	-3.356803830023	1.708685497844	-3.030474028724
C	-6.847214232838	-0.594114040828	-1.774133863034
H	-7.080134555730	0.185037094511	-1.031330400014
H	-6.995792101050	-0.164846759538	-2.774778552797
H	-7.589038995749	-1.397376448228	-1.638627395822
C	-3.364491735258	-2.740113180209	1.142809910753
H	-3.662476467837	-3.801446400694	1.171346829424
H	-2.283427473002	-2.695221116804	1.323533649728
H	-3.863808650080	-2.226648742581	1.975151835097
C	-2.276484933510	-1.605737059480	-3.663049089424
H	-2.743858308170	-1.185855651737	-4.564021648709
H	-1.358589048609	-1.038174300767	-3.428426357037
H	-1.974098767323	-2.641948688663	-3.888191262641

97

9_opt, 1-A (D2): E(RPBE0-D3BJ/def2SVP) = -3552.42704975

G_corr(298K) = 0.727457

E(COSMO-PBE0-D3BJ/def2TZVP) = -3554.80744600

Ni	0.000000000000	0.000000000000	0.000000000000

N	-1.044314715126	2.734911887226	0.221388494242
N	1.044314715126	2.734911887226	-0.221388494242
C	-0.000000000000	1.912641568761	0.000000000000
C	-0.663984337474	4.064635874499	0.139699993192
H	-1.371173068064	4.875723711104	0.286345606985
C	0.663984337474	4.064635874499	-0.139699993192
H	1.371173068064	4.875723711104	-0.286345606985
F	0.000000000000	0.000000000000	1.841943446129
N	1.044314715126	-2.734911887226	0.221388494242
N	-1.044314715126	-2.734911887226	-0.221388494242
C	-0.000000000000	-1.912641568761	-0.000000000000
C	0.663984337474	-4.064635874499	0.139699993192
H	1.371173068064	-4.875723711104	0.286345606985
C	-0.663984337474	-4.064635874499	-0.139699993192
H	-1.371173068064	-4.875723711104	-0.286345606985
F	0.000000000000	-0.000000000000	-1.841943446129
C	-2.376697573487	-2.299086036374	-0.496551176757
C	-2.784271744233	-2.191360803175	-1.833207711849
C	-3.224754515822	-2.015004223612	0.579332177591
C	-4.119088789428	-1.865537451928	-2.073776160046
C	-4.554353792547	-1.703352303578	0.283962942548
C	-5.023440320816	-1.647177856958	-1.029406944775
H	-4.461765842278	-1.784681701228	-3.109641786040
H	-5.239526257652	-1.495231101093	1.109374995577
C	2.376697573487	-2.299086036374	0.496551176757
C	3.224754515822	-2.015004223612	-0.579332177591
C	2.784271744233	-2.191360803175	1.833207711849
C	4.554353792547	-1.703352303578	-0.283962942548
C	4.119088789428	-1.865537451928	2.073776160046
C	5.023440320816	-1.647177856958	1.029406944775
H	5.239526257652	-1.495231101093	-1.109374995577
H	4.461765842278	-1.784681701228	3.109641786040
C	-2.376697573487	2.299086036374	0.496551176757
C	-2.784271744233	2.191360803175	1.833207711849

C	-3.224754515822	2.015004223612	-0.579332177591
C	-4.119088789428	1.865537451928	2.073776160046
C	-4.554353792547	1.703352303578	-0.283962942548
C	-5.023440320816	1.647177856958	1.029406944775
H	-4.461765842278	1.784681701228	3.109641786040
H	-5.239526257652	1.495231101093	-1.109374995577
C	2.376697573487	2.299086036374	-0.496551176757
C	2.784271744233	2.191360803175	-1.833207711849
C	3.224754515822	2.015004223612	0.579332177591
C	4.119088789428	1.865537451928	-2.073776160046
C	4.554353792547	1.703352303578	0.283962942548
C	5.023440320816	1.647177856958	-1.029406944775
H	4.461765842278	1.784681701228	-3.109641786040
H	5.239526257652	1.495231101093	1.109374995577
C	-1.790665575973	2.358416940438	2.941121965982
H	-1.289644382246	3.338541884377	2.900903059999
H	-2.272984771740	2.257928113945	3.922677910799
H	-1.013598698424	1.580668920239	2.832777072893
C	-6.463560493024	1.346072695711	1.328193990103
H	-6.924888535706	2.157769687229	1.912622824333
H	-7.049463907484	1.218014459173	0.407965732280
H	-6.565438615746	0.425448014746	1.925344783940
C	-2.699779522491	2.009550148474	-1.983253341064
H	-3.493625577508	1.750989496834	-2.696584480893
H	-2.285991489089	2.990959137987	-2.266347905440
H	-1.880748649837	1.275372550504	-2.081688510856
C	-2.699779522491	-2.009550148474	1.983253341064
H	-1.880748649837	-1.275372550504	2.081688510856
H	-3.493625577508	-1.750989496834	2.696584480893
H	-2.285991489089	-2.990959137987	2.266347905440
C	-6.463560493024	-1.346072695711	-1.328193990103
H	-6.924888535706	-2.157769687229	-1.912622824333
H	-7.049463907484	-1.218014459173	-0.407965732280
H	-6.565438615746	-0.425448014746	-1.925344783940

C	-1.790665575973	-2.358416940438	-2.941121965982
H	-1.289644382246	-3.338541884377	-2.900903059999
H	-2.272984771740	-2.257928113945	-3.922677910799
H	-1.013598698424	-1.580668920239	-2.832777072893
C	6.463560493024	-1.346072695711	1.328193990103
H	7.049463907484	-1.218014459173	0.407965732280
H	6.565438615746	-0.425448014746	1.925344783940
H	6.924888535706	-2.157769687229	1.912622824333
C	1.790665575973	-2.358416940438	2.941121965982
H	2.272984771740	-2.257928113945	3.922677910799
H	1.013598698424	-1.580668920239	2.832777072893
H	1.289644382246	-3.338541884377	2.900903059999
C	2.699779522491	-2.009550148474	-1.983253341064
H	2.285991489089	-2.990959137987	-2.266347905440
H	1.880748649837	-1.275372550504	-2.081688510856
H	3.493625577508	-1.750989496834	-2.696584480893
C	6.463560493024	1.346072695711	-1.328193990103
H	6.565438615746	0.425448014746	-1.925344783940
H	6.924888535706	2.157769687229	-1.912622824333
H	7.049463907484	1.218014459173	-0.407965732280
C	2.699779522491	2.009550148474	1.983253341064
H	2.285991489089	2.990959137987	2.266347905440
H	1.880748649837	1.275372550504	2.081688510856
H	3.493625577508	1.750989496834	2.696584480893
C	1.790665575973	2.358416940438	-2.941121965982
H	2.272984771740	2.257928113945	-3.922677910799
H	1.013598698424	1.580668920239	-2.832777072893
H	1.289644382246	3.338541884377	-2.900903059999

6

c2h4_opt, 1-AG (D2H): E(RPBE0-D3BJ/def2SVP) = -78.4273808015

G_corr(298K) = 0.030141

E(COSMO-PBE0-D3BJ/def2TZVP) = -78.5160389781

C	0.000000000000	-0.000000000000	0.665273210322

C	-0.000000000000	0.000000000000	-0.665273210322
H	0.000000000000	0.931026660321	1.240504091321
H	0.000000000000	-0.931026660321	1.240504091321
H	-0.000000000000	0.931026660321	-1.240504091321
H	-0.000000000000	-0.931026660321	-1.240504091321

11

c6f5_opt, 2-A1 (C2V): E(UPBE0-D3BJ/def2SVP) = -726.436723687

G_corr(298K) = 0.014196

E(COSMO-PBE0-D3BJ/def2TZVP) = -727.295126051

C	-0.000000000000	1.220458519396	-1.009376141361
C	0.000000000000	0.000000000000	-1.640445794057
C	-0.000000000000	1.215793151042	0.389776734186
C	-0.000000000000	-1.220458519396	-1.009376141361
C	0.000000000000	0.000000000000	1.076937149238
C	-0.000000000000	-1.215793151042	0.389776734186
F	0.000000000000	-2.367695437392	-1.661352982816
F	0.000000000000	-2.349506613281	1.064568380514
F	0.000000000000	-0.000000000000	2.395374177384
F	0.000000000000	2.349506613281	1.064568380514
F	0.000000000000	2.367695437392	-1.661352982816

12

c6f6_opt, 1-A1G (D6H): E(RPBE0-D3BJ/def2SVP) = -826.185296400

G_corr(298K) = 0.019443

E(COSMO-PBE0-D3BJ/def2TZVP) = -827.166644309

C	-0.000000000000	1.393784969313	0.000000000000
C	1.207053190838	0.696892484657	0.000000000000
C	-1.207053190838	0.696892484657	0.000000000000
C	1.207053190838	-0.696892484657	0.000000000000
C	-1.207053190838	-0.696892484657	0.000000000000
C	-0.000000000000	-1.393784969313	0.000000000000
F	2.349296691983	1.356367077523	-0.000000000000
F	2.349296691983	-1.356367077523	-0.000000000000

F	-0.000000000000	-2.712734155046	-0.000000000000
F	-2.349296691983	-1.356367077523	-0.000000000000
F	-2.349296691983	1.356367077523	-0.000000000000
F	0.000000000000	2.712734155046	-0.000000000000

61

I10_opt, 1-A (C1): E(RPBE0-D3BJ/def2SVP) = -2508.87543408

G_corr(298K) = 0.477311

E(COSMO-PBE0-D3BJ/def2TZVP) = -2510.15399632

Ni	-0.000055509651	-0.001369715317	1.252044225822
C	-0.614227675768	0.364424242389	3.067736185908
H	-0.605951594406	1.426029983277	3.351921260638
H	-1.542155765108	-0.139564634058	3.376389819061
C	0.613716295747	-0.374636800089	3.066261517841
H	0.605331018516	-1.437439607741	3.345957306393
H	1.541656493071	0.127970406469	3.377097521624
C	1.284878866569	-0.746699839524	0.067954463035
N	2.599273788919	-0.409213666576	-0.089575696882
C	3.196590149598	-1.119795728213	-1.112998805386
H	4.240157486855	-1.000249791007	-1.390840306637
N	1.089078572397	-1.689805493364	-0.901044310016
C	2.238276876167	-1.930362592677	-1.629974364730
H	2.288717169552	-2.648213384749	-2.444074020170
N	-1.089029221600	1.692346382390	-0.897180329425
N	-2.599212632868	0.409850461855	-0.088727091153
C	-1.284909680444	0.747188740234	0.069798272495
C	-2.238122588998	1.934301140057	-1.625827504806
H	-2.288498049024	2.653891806986	-2.438392401918
C	-3.196425070844	1.122513905902	-1.110756823130
H	-4.239927924576	1.003434916388	-1.389034701652
C	3.279439191881	0.512491569850	0.810835393222
H	2.454904912541	0.930491393526	1.409690170974
C	3.974597957120	1.629274082560	0.048845509918
C	4.211208141850	-0.242769985849	1.746201864087

H	3.649355618430	-1.016235771047	2.288624894291
H	5.032814311586	-0.726662819510	1.193515771301
H	4.655232714516	0.443494248822	2.482640362525
H	4.801681478013	1.249329411239	-0.572377946240
H	4.399820671996	2.358905014416	0.753414779392
H	3.269102711455	2.157669042022	-0.608446492757
C	-0.202018909451	-2.327042462091	-1.115041058404
H	-0.812390132889	-1.926076130911	-0.290487338739
C	-0.102329121022	-3.837024942524	-0.969356356259
C	-0.818668800889	-1.887680521080	-2.434670295025
H	-0.945440881267	-0.795649885226	-2.456355089955
H	-0.192784537077	-2.185016951163	-3.291500931270
H	-1.808810937661	-2.348167479068	-2.569267506792
H	0.510305055777	-4.285824164085	-1.767921754909
H	-1.102860291078	-4.291309373259	-1.024471920910
H	0.345103579948	-4.101930588775	-0.000442448731
C	0.202047270055	2.330050492239	-1.109940964916
H	0.812316277155	1.927850099076	-0.285918229129
C	0.102242099624	3.839794663355	-0.962046129282
C	0.818909083378	1.892670664746	-2.430155564122
H	0.945854369946	0.800697272400	-2.453326378019
H	0.192942058264	2.191054025412	-3.286560124476
H	1.808948015704	2.353561952811	-2.564118614780
H	-0.510136151475	4.289785794258	-1.760134536792
H	1.102781722881	4.294173914539	-1.016187345298
H	-0.345492515234	4.103297365101	0.007111943797
C	-3.279384260416	-0.513950280871	0.809521269302
H	-2.454868393662	-0.933348874176	1.407399271218
C	-3.974558956401	-1.628986254093	0.044965564648
C	-4.211137740690	0.239109361671	1.746655446479
H	-3.649253981595	1.011230796927	2.290975512592
H	-5.032709480866	0.724386264791	1.195130301087
H	-4.655205971071	-0.448907656680	2.481429796876
H	-4.801523011936	-1.247606920299	-0.575537476357

H	-4.399935986012	-2.360118345948	0.747883657126
H	-3.269038101391	-2.156039827975	-0.613378859274

66

I11cis_opt, 1-A (C1): E(RPBE0-D3BJ/def2SVP) = -3156.79676761

G_corr(298K) = 0.469245

E(COSMO-PBE0-D3BJ/def2TZVP) = -3158.88178454

Ni	0.184403121131	-0.271910471787	-0.561897732904
N	2.671756706812	-1.646807620686	-1.012332502596
N	3.071156872331	0.460870670119	-1.062566397808
C	2.074931123042	-0.436535711469	-0.907270663555
C	4.023229059655	-1.512670106700	-1.227109251744
H	4.694407195273	-2.357621473813	-1.353116271086
C	4.277173955811	-0.174769402481	-1.253576393787
H	5.211768695003	0.358455925111	-1.403166509278
C	-1.683823061774	-0.413014257167	-0.409254973109
C	-2.473903180886	0.113476442309	-1.425508097932
C	-3.857572602677	-0.026524511277	-1.459825945512
C	-4.481806213778	-0.745477314765	-0.438589775940
F	-1.895546442153	0.826032677077	-2.405644365336
F	-4.578645134566	0.494956063039	-2.433630620946
F	-5.787066233594	-0.904409147937	-0.451213550840
C	-2.342511632714	-1.123767881613	0.586646938955
C	-3.724433928472	-1.292681150526	0.599717189508
F	-1.645330402941	-1.663753983692	1.593528180428
F	-4.322433196951	-1.973708463879	1.558476245865
N	-0.147866417699	2.060983765699	1.065533265110
N	0.790382115389	0.435859313157	2.126142397794
C	0.269435072068	0.785005401764	0.932702689663
C	0.116885958624	2.516690627834	2.341324709979
H	-0.140356529586	3.518667909917	2.671933457687
C	0.708350608037	1.493770480189	3.009623156269
H	1.065414341344	1.433860136220	4.033394146809
C	2.875451110390	1.914467285322	-1.030471267455

H	1.782193372084	2.028027779849	-1.023263729216
C	3.423649415733	2.563082919933	-2.290574867906
C	3.442466667082	2.507191925363	0.249541338782
H	2.982938054790	2.050396833698	1.137615456824
H	4.532428156190	2.364389578789	0.309132844507
H	3.248259713897	3.588741678682	0.282609994768
H	4.521448817909	2.500310338359	-2.339632169814
H	3.157291567573	3.629488131508	-2.304228985318
H	3.006356768392	2.093901326664	-3.192981012979
C	1.922321665909	-2.909301332971	-0.979652034587
H	0.914152174324	-2.611962964140	-0.633122865878
C	2.513257027292	-3.876903144264	0.030236090114
C	1.802898719003	-3.497255032899	-2.376724052772
H	1.353036947275	-2.775674020368	-3.074128474548
H	2.788927120591	-3.788329249925	-2.770083608477
H	1.170765357268	-4.396131506169	-2.355275402537
H	3.511277095946	-4.223673882003	-0.278164196537
H	1.870155834400	-4.763852611116	0.114478405602
H	2.598669799793	-3.417607442280	1.024863637270
C	-0.881702300667	2.819839623483	0.043135812324
H	-0.827885309735	2.189731668416	-0.855788882073
C	-0.209271291504	4.152620759937	-0.241711338214
C	-2.340362150203	2.971555163054	0.447377952145
H	-2.785954926142	1.996257729132	0.686269033552
H	-2.447081415924	3.628224969761	1.324539525213
H	-2.911588285212	3.417499881628	-0.379084292359
H	-0.247973084551	4.824647056197	0.629043760119
H	-0.730602982397	4.656711615811	-1.067327638731
H	0.843326519435	4.025619027224	-0.531231590323
C	1.358375456837	-0.880162745471	2.443066064367
H	1.068065374075	-1.505487268637	1.585931130690
C	0.723014072350	-1.455037605639	3.697851579404
C	2.874873261299	-0.800209999436	2.526318321113
H	3.313919676194	-0.434994973046	1.587302510566

H	3.192152732650	-0.129701443494	3.339762752620
H	3.294363509410	-1.794039024406	2.738573584560
H	1.012690169133	-0.889951629439	4.596884407616
H	1.063194219420	-2.490407102118	3.840534471628
H	-0.371421179204	-1.460743580536	3.616143609716

66

I11trans_opt, 1-A (C1): E(RPBE0-D3BJ/def2SVP) = -3156.80114058

G_corr(298K) = 0.468204

E(COSMO-PBE0-D3BJ/def2TZVP) = -3158.88202577

Ni	-0.000023591690	-0.969835876533	0.000016967182
N	-2.679005164025	-0.639316002188	1.222496697725
N	-2.746375249303	-1.527268511585	-0.732044614340
C	-1.894228352691	-1.046728427225	0.201451815736
C	-4.004759767034	-0.862663028156	0.935178914333
H	-4.809669643925	-0.610093541738	1.619670103096
C	-4.048190212565	-1.429108967580	-0.302776342990
H	-4.896438047578	-1.762939931927	-0.893549377192
C	0.000019211774	0.876284560335	-0.000029450054
C	-0.552823196262	1.602319794771	-1.045375307353
C	-0.561200041411	2.995288449965	-1.067998922865
C	0.000086928937	3.695898782454	-0.000092560889
F	-1.080055475304	0.962044682913	-2.097891527506
F	-1.082674436190	3.651673786047	-2.086678456624
F	0.000119072111	5.012725225430	-0.000122389782
C	0.552896869893	1.602340012430	1.045283658565
C	0.561340957734	2.995309321106	1.067844777636
F	1.080102583113	0.962085834628	2.097826026965
F	1.082848640783	3.651715185057	2.086493990961
N	2.678977825323	-0.639510279461	-1.222473236054
N	2.746300282582	-1.527323341842	0.732132867316
C	1.894178328011	-1.046819831362	-0.201404543235
C	4.004722086709	-0.862884711986	-0.935128567294
H	4.809646571358	-0.610393904331	-1.619631608460

C	4.048122518062	-1.429239435240	0.302869486557
H	4.896353755293	-1.763054747110	0.893675159515
C	-2.305991600043	-2.092756286873	-2.012513217132
H	-1.232470877089	-1.838548893944	-2.055882551102
C	-3.009582000943	-1.418542370954	-3.177229965095
C	-2.460355930980	-3.605626561735	-2.009970944019
H	-1.926719741097	-4.060741394664	-1.162409290341
H	-3.519811982859	-3.896983510152	-1.943213042805
H	-2.056337916813	-4.028539589727	-2.940747528566
H	-4.083881282299	-1.657848319957	-3.193286452143
H	-2.582052276087	-1.775636609625	-4.124697262921
H	-2.889322747160	-0.328242084148	-3.130503987644
C	-2.179243789998	-0.007781093222	2.449939207768
H	-1.086295747283	-0.035185646559	2.341563548563
C	-2.571060236884	-0.819465345425	3.673479391001
C	-2.624025485341	1.444596484899	2.518747478790
H	-2.344241269253	1.983705168489	1.602425018620
H	-3.713428269542	1.528991323885	2.653257011928
H	-2.142014711269	1.942885434697	3.371851748448
H	-3.660654654699	-0.822590366263	3.830084175089
H	-2.111130306446	-0.382378209784	4.570989778264
H	-2.232140951189	-1.861958320797	3.584600313639
C	2.179249900595	-0.008048902300	-2.449967206612
H	1.086299963966	-0.035416239276	-2.341604553200
C	2.571060242996	-0.819831235209	-3.673444263155
C	2.624071742177	1.444311699897	-2.518873194726
H	2.344287718781	1.983493768063	-1.602593782059
H	3.713478832211	1.528667791549	-2.653372166423
H	2.142087318577	1.942552139118	-3.372020690622
H	3.660656662149	-0.822997411025	-3.830034390315
H	2.111154131296	-0.382795571400	-4.570991928522
H	2.232111170655	-1.862308570552	-3.584495202416
C	2.305884284993	-2.092692512921	2.012642522611
H	1.232368128114	-1.838459322915	2.055972144822

C	3.009466632809	-1.418389656860	3.177312403303
C	2.460218292160	-3.605566199191	2.010234085722
H	1.926587114778	-4.060743858462	1.162702997290
H	3.519669621214	-3.896950125207	1.943519001520
H	2.056176245434	-4.028390528002	2.941040557676
H	4.083761886328	-1.657710969850	3.193407374352
H	2.581915480827	-1.775394923523	4.124803515497
H	2.889224772072	-0.328091210802	3.130489947055

67

I12_opt, 1-A (C1): E(UPBE0-D3BJ/def2SVP) = -3256.59775952

G_corr(298K) = 0.465764

E_BS(COSMO-PBE0-D3BJ/def2TZVP) = -3258.75853309 <S**2> = 0.9948

E_T(COSMO-PBE0-D3BJ/def2TZVP) = -3258.75806890 <S**2> = 2.0162

C	4.749485769467	-1.228516583972	0.039540451859
C	4.988057333907	0.109058400247	0.101725450470
C	2.745281318431	-0.191740668731	0.065995386375
N	3.381352168173	-1.386148219221	0.024379943556
N	3.752166087557	0.719892269247	0.117891373817
Ni	0.854372905008	0.207445346814	-0.080262303038
C	-0.818093943719	1.189592549579	-0.024956216028
N	-1.520450870588	1.613973717267	-1.103462753850
N	-1.531674442011	1.637836646606	1.038241037860
C	-2.646849650387	2.311299443799	-0.726162242158
C	-2.655787833983	2.327401925585	0.633524675803
H	-3.364860680505	2.711756013746	-1.433763090011
H	-3.382413372256	2.744060964859	1.322548044438
H	5.441817377792	-2.065026550350	0.008533917985
H	5.925557103100	0.657116887305	0.135352932231
C	-1.316823914755	-2.077300364417	-0.654213813332
C	-1.314413310633	-2.070992627659	0.720775544103
C	-2.396767118669	-1.579788798904	-1.345779749840
C	-2.410915077712	-1.582852326404	1.427494479268
C	-3.488905275636	-1.044693865774	-0.664084815349

C	-3.485732831483	-1.036731808827	0.729710716230
F	0.377686805157	-1.243441918430	-1.354079652348
F	-2.421855569698	-1.560335550837	-2.681506151288
F	-4.515227176029	-0.510383018879	-1.321352258190
F	-4.502244571854	-0.496374067982	1.396864139920
F	-2.420656298344	-1.571861011158	2.760233028074
F	-0.266767344324	-2.516334298350	1.429561705870
C	-1.067377298081	1.352036868845	-2.477034532710
H	-0.683781843693	0.320374456466	-2.451178443815
C	0.080438818528	2.287556179378	-2.828609447986
C	-2.215605703620	1.439590104514	-3.464371293237
H	-3.055921558028	0.801404145894	-3.160432811635
H	-2.573731956520	2.474161674601	-3.590867497483
H	-1.870434441357	1.088016106073	-4.446522143767
H	-0.239534030990	3.341663637676	-2.800920070052
H	0.458269721513	2.062865390571	-3.836933179026
H	0.905132375622	2.147027754042	-2.113439619440
C	-1.100114244279	1.407178439383	2.420116560515
H	-0.746558893826	0.364828334871	2.438541862319
C	-2.254331918456	1.538660907659	3.396447341426
C	0.068327131585	2.319777842141	2.763009809864
H	0.901017733768	2.139962412804	2.066718177258
H	-0.225630171360	3.378890391058	2.694281352790
H	0.424748472571	2.120789708215	3.784506906263
H	-2.598947967140	2.581129832814	3.484167751330
H	-1.922255273594	1.216273646549	4.392989758702
H	-3.101463492137	0.903985841033	3.103657543691
C	2.674426904003	-2.665855647423	-0.075285674251
H	1.622206454420	-2.398903098322	0.072761902942
C	3.116696400895	-3.616369239630	1.023528981333
C	2.803141668712	-3.237068281204	-1.476637521214
H	2.356609829219	-2.533417650613	-2.191777682960
H	3.852899901298	-3.437357548643	-1.747375342828
H	2.245059203660	-4.182317505400	-1.544478534063

H	4.161073815140	-3.945817693104	0.897760002584
H	2.481766304726	-4.513703682693	1.008735933385
H	3.015534338289	-3.146161906606	2.012808728416
C	3.508149789899	2.157375064802	0.150885644131
H	2.406173349142	2.220995476984	0.143370473376
C	4.035459895299	2.830273114555	-1.106237923157
C	4.036146910739	2.776758274508	1.434630114204
H	3.619174010552	2.264357995930	2.313981462392
H	5.134750558964	2.717557559182	1.491275757107
H	3.756030253553	3.839063696752	1.487458318184
H	5.133621875582	2.769241236993	-1.168640611694
H	3.757843424150	3.894537934867	-1.113020891905
H	3.612270111733	2.355969306808	-2.003662497588

101

I13_opt, 1-A (C1): E(RPBE0-D3BJ/def2SVP) = -3431.43385053

G_corr(298K) = 0.771795

E(COSMO-PBE0-D3BJ/def2TZVP) = -3433.65940567

Ni	-0.000180039539	-0.000016157545	-0.872032005204
C	0.197982093468	0.680849095351	-2.708387213215
H	-0.498082400688	1.465450305351	-3.025928640538
H	1.246454490895	0.921335266696	-2.930196212145
C	-0.199205413897	-0.681450114247	-2.708056541075
H	0.496690836411	-1.466169148543	-3.025692294149
H	-1.247807890237	-0.922046238228	-2.929104910691
C	0.055023872660	-1.732179084260	-0.099432115009
N	-0.978728470678	-2.640309816583	-0.087506351000
C	-0.560745559796	-3.927258317996	0.208968476086
H	-1.252328497375	-4.763551129391	0.253573153516
N	1.132786659261	-2.525258794268	0.229581941070
C	0.772690023025	-3.857330408380	0.406701020859
H	1.504727802334	-4.619095487292	0.658939698246
C	2.469313691014	-2.127105658501	0.528293666139
C	3.422268285865	-1.999233652798	-0.491135894666

C	4.759678733248	-1.832630657860	-0.121851157415
H	5.512106827276	-1.758094864329	-0.912199133108
C	5.161390851038	-1.778257926533	1.212985130098
C	4.177577523841	-1.863271338330	2.201995265101
H	4.466592171298	-1.804897565516	3.255845978057
C	2.831064501030	-2.041936857796	1.883042960809
C	3.027814361830	-2.033678947914	-1.933086953874
H	3.897323424256	-2.222027622185	-2.577172966637
H	2.584967711983	-1.067733639410	-2.221876025332
H	2.264470630735	-2.800146316333	-2.127566609256
C	1.788563872587	-2.157191631235	2.955414257716
H	1.432324722181	-3.194300108076	3.064686195948
H	0.906349346564	-1.546546425653	2.711660224306
H	2.185984411156	-1.831887977108	3.926520351125
C	6.604499594253	-1.590956558041	1.580533922494
H	7.272680600382	-1.922642323560	0.772955592572
H	6.864493582560	-2.148116774566	2.492569068388
H	6.830920629105	-0.528418731501	1.773721640253
C	-2.346441525537	-2.313379414839	-0.287593136630
C	-3.031166948768	-1.670006095420	0.756075087968
C	-4.392271962957	-1.426792850256	0.595584241742
H	-4.935055684481	-0.941978575865	1.409536073418
C	-5.073982798700	-1.790566061137	-0.570789914822
C	-4.360117209365	-2.431565554323	-1.582877791440
H	-4.879547130166	-2.732437281170	-2.497655900095
C	-2.996903903299	-2.720621601250	-1.458372381814
C	-2.300769002949	-1.290513182086	2.007055901739
H	-1.820595382756	-2.169017232288	2.467553390328
H	-2.978638457633	-0.832145467013	2.737974954217
H	-1.497523033629	-0.577359537098	1.762996907258
C	-6.534693932659	-1.483610182909	-0.727198852015
H	-6.692383370461	-0.410081162140	-0.922225839704
H	-7.097566924687	-1.729766939732	0.186241811960
H	-6.979356850776	-2.041984822434	-1.562995956152

C	-2.277083648109	-3.485969711570	-2.529490937352
H	-1.214351478511	-3.217197291977	-2.572850567582
H	-2.724879483106	-3.295722312172	-3.515080083093
H	-2.337097126771	-4.572096094636	-2.346047895469
C	2.346527730748	2.313010342013	-0.287740367939
N	-1.132673171950	2.525472930313	0.229541273660
C	2.996811424574	2.719999682313	-1.458720928651
N	0.978858037479	2.640070720988	-0.087563545534
C	4.360019223146	2.431037019056	-1.583312378736
H	4.879317552750	2.731714176585	-2.498227984666
C	5.074087554004	1.790441943109	-0.571087089622
C	4.392554253577	1.426907590184	0.595450029059
H	4.935481474003	0.942417513382	1.409506387374
C	3.031413005538	1.669961564227	0.755997188396
C	2.276726996724	3.485097611106	-2.529831952475
H	2.335525723963	4.571197973293	-2.345846606984
H	1.214271481725	3.215297350030	-2.573736782749
H	2.725160362784	3.295760247955	-3.515301360499
C	6.534806639537	1.483621524777	-0.727672854291
H	6.979647747720	2.043174487002	-1.562589744298
H	6.692470804291	0.410368973781	-0.924263926077
H	7.097548718099	1.728414647906	0.186208783677
C	2.301141068951	1.290656138590	2.007112179141
H	2.979018116456	0.832112753054	2.737916608481
H	1.497683918963	0.577694609229	1.763199407802
H	1.821276482634	2.169283552670	2.467707715567
C	-0.055093946632	1.732163203631	-0.099509530893
C	-0.772277131796	3.857433883869	0.406843414442
H	-1.504146100777	4.619329565468	0.659172317887
C	0.561159750597	3.927091872847	0.209020718605
H	1.252923441454	4.763234111135	0.253663395900
C	-2.469247180133	2.127476169315	0.528222123914
C	-3.422233544641	1.999826987590	-0.491188373161
C	-4.759640643088	1.833124644978	-0.121872653682

H	-5.512108318742	1.758697562720	-0.912192106448
C	-5.161299885337	1.778497150746	1.212952953118
C	-4.177448114748	1.863355145612	2.201953422761
H	-4.466415376689	1.804753784083	3.255805762971
C	-2.830960495330	2.042066851389	1.882982548958
C	-1.788420354003	2.157148217921	2.955335753927
H	-0.906057939402	1.546850026680	2.711278750913
H	-2.185699176766	1.831336833868	3.926331066765
H	-1.432480084079	3.194318606949	3.065028757160
C	-3.027968488703	2.034550346930	-1.933191926528
H	-2.262783743406	2.799273313093	-2.127160324425
H	-3.897155626947	2.225580883860	-2.576936200030
H	-2.587636229167	1.067734985547	-2.222960328953
C	-6.604406636500	1.591261315789	1.580574634111
H	-6.830389313715	0.529060818211	1.776050867179
H	-7.272558054928	1.920971339446	0.772167685246
H	-6.864849953637	2.150289546376	2.491355059646

107

I14_opt, 1-A (C1): E(RPBE0-D3BJ/def2SVP) = -4179.17444882

G_corr(298K) = 0.762791

E(COSMO-PBE0-D3BJ/def2TZVP) = -4182.27476012

C	1.972760264457	3.083789153971	2.160109637698
C	0.963264098072	3.773305644236	1.583654177965
C	0.818027439150	1.610490634860	0.849943137373
N	1.879696352270	1.782077231067	1.700287346773
N	0.275789441987	2.868322425689	0.792244584127
Ni	0.121925748937	0.039983034885	-0.012164709570
C	-0.166320183407	-1.156670907501	-1.512061808848
N	0.801150403530	-1.544630752183	-2.397243324138
N	-1.304375562836	-1.690174246804	-2.062621638149
C	0.287584541922	-2.281425482438	-3.450618788598
C	-1.044582692302	-2.363269675116	-3.242938119760
H	0.918590100651	-2.671377711819	-4.243484244089

H	-1.831346106001	-2.830096932140	-3.826127879787
H	2.762280928867	3.395116446861	2.837665871842
H	0.672801268963	4.817771068891	1.648206487616
C	-2.626820391416	-1.612259199258	-1.529752712461
C	-3.315758219523	-2.800563927727	-1.241173248404
C	-3.228234917173	-0.355355625731	-1.369737068695
C	-4.614548323731	-2.696255096893	-0.736902990189
C	-4.515951035613	-0.306197335504	-0.837223626136
C	-5.224409223437	-1.463290691382	-0.505006192384
H	-5.149113234633	-3.614435319864	-0.480045349729
H	-4.981373556067	0.672493535039	-0.689124767924
C	2.182780148645	-1.184769388969	-2.389206596815
C	2.629389490555	-0.293550424093	-3.378421915264
C	3.073065306137	-1.827372841449	-1.514937445799
C	4.002973706428	-0.067633695158	-3.492036374268
C	4.436463407768	-1.583556473212	-1.687034992450
C	4.923395003770	-0.715751380821	-2.666886222504
H	4.360940095480	0.630003014546	-4.254814328740
H	5.139921313410	-2.097070626149	-1.026846839674
C	2.949196279097	0.866327268936	1.953671232940
C	3.069920872267	0.240793154284	3.200653123097
C	3.938266996109	0.742998828224	0.966454756178
C	4.174249447338	-0.590007771127	3.407559385571
C	5.031790465655	-0.081832098769	1.229056363180
C	5.157562198275	-0.775325513492	2.435089466640
H	4.266811266676	-1.104937457026	4.368375112463
H	5.813549326313	-0.173800064816	0.470756621046
C	-0.774490601452	3.312815940268	-0.067051178274
C	-0.481625001463	3.482441603354	-1.432684936753
C	-2.012030608658	3.693779626776	0.469635175645
C	-1.461131138189	4.030547099097	-2.257885140565
C	-2.959249452525	4.245724184430	-0.401366777833
C	-2.708375873157	4.422280678226	-1.761462522989
H	-1.239675764979	4.161824451149	-3.321033874544

H	-3.927015947816	4.549876045962	0.008086300713
C	-2.525051676640	0.893593871758	-1.784196284859
H	-1.614493048715	1.047284788185	-1.179486780353
H	-2.193278452782	0.838378072679	-2.833374573483
H	-3.169664221506	1.771527788713	-1.663691791885
C	-2.689632410950	-4.153273981154	-1.427636602058
H	-2.715714121570	-4.479614919299	-2.479404375091
H	-1.639346845332	-4.154154879260	-1.104989993280
H	-3.226091117796	-4.904010381358	-0.834852384762
C	-6.581712405138	-1.376473357363	0.125422273991
H	-7.169249407089	-2.289141355824	-0.048611723969
H	-6.477332566297	-1.251840593767	1.215144306301
H	-7.152366853685	-0.517464007165	-0.256917568399
C	2.585240727633	-2.723097702217	-0.423281272073
H	1.815268044464	-3.422463705502	-0.777480668949
H	3.414634136873	-3.294015516048	0.014774888911
H	2.116567800689	-2.129451007740	0.376963524593
C	1.667844678630	0.387043005882	-4.309623011608
H	1.444427111038	-0.238235490767	-5.189342512654
H	0.709414070878	0.602021412556	-3.817329579075
H	2.090360646383	1.332364750755	-4.677529961068
C	6.394828811214	-0.447681052559	-2.787731117212
H	6.9884883116050	-1.340067464952	-2.541397453600
H	6.667213600602	-0.121341679019	-3.801583708807
H	6.707095427289	0.351317163464	-2.092813919863
C	0.843000619888	3.058119831106	-1.984361439471
H	1.677828606284	3.529112424490	-1.443614113733
H	0.968384407016	1.969015610650	-1.860629496078
H	0.928085196160	3.308939300621	-3.049730021294
C	-2.356275639133	3.507647106122	1.916491435307
H	-1.478767571590	3.558865722511	2.572049270526
H	-3.084750430106	4.264183416126	2.240000311600
H	-2.808533683723	2.519138929233	2.083308429293
C	-3.757338271493	4.979097266600	-2.678631409100

H	-3.316996389052	5.633843116378	-3.445123732012
H	-4.282817073092	4.166878450262	-3.208797701984
H	-4.513651082840	5.554960302641	-2.126833996631
C	2.079972221680	0.462464123080	4.304161468886
H	1.121428666617	0.832266517295	3.927521979155
H	1.893220132892	-0.475375710360	4.845824478686
H	2.470761858055	1.192163337295	5.033300289621
C	3.829686350998	1.501200876355	-0.319319283486
H	3.575077921571	2.556925150299	-0.139529686968
H	4.770772944533	1.461880666858	-0.880956715612
H	3.034803588768	1.078935911628	-0.952669545205
C	6.311157703458	-1.707008596108	2.668320949248
H	6.561581969928	-1.783877296752	3.736251958144
H	6.069010808951	-2.724263694419	2.316572484287
H	7.210843083790	-1.379870174222	2.126542919731
C	-0.400968975980	-0.954900760296	1.596002304952
C	-1.293088088491	-0.098934764886	2.330626469347
C	-0.983095253808	-2.248446271660	1.338685860284
C	-2.627571091134	-0.395637284586	2.550559163553
F	-0.829507712763	1.033981472251	2.880801623970
C	-2.284460701911	-2.581643768235	1.648064605072
F	-0.197857850690	-3.198071286763	0.815960722213
C	-3.144587199161	-1.645747676968	2.225599495850
F	-3.420348540871	0.507077864626	3.131291947081
F	-2.731944731554	-3.814464234203	1.421908107122
F	-4.417834115720	-1.950841815884	2.473009118718
F	0.880937507760	-1.096352211733	2.156497227436

107

I15_opt, 1-A (C1): E(UPBE0-D3BJ/def2SVP) = -4179.17139544

G_corr(298K) = 0.754996

E_BS(COSMO-PBE0-D3BJ/def2TZVP) = -4182.28327932 <S**2> = 0.9775

E_T(COSMO-PBE0-D3BJ/def2TZVP) = -4182.28123228 <S**2> = 2.0201

C	-2.414969995466	1.400768387726	-3.586675100091

C	-2.606600209892	0.060178319498	-3.690582243691
C	-1.662758469006	0.457808034381	-1.663930248836
N	-1.843322466308	1.616332342006	-2.345167898057
N	-2.145955530738	-0.488898121870	-2.507116285052
Ni	-0.684647646409	0.240257239993	-0.013472279887
C	-0.266446443628	0.188480340294	1.869112727466
N	-1.223221022261	-0.071664855398	2.800387425453
N	0.838159954325	0.452204725536	2.618494606916
C	-0.741321345383	0.032746487418	4.092640168345
C	0.566278936677	0.369789930721	3.974124715940
H	-1.365044815725	-0.141571747385	4.964587937355
H	1.326137618410	0.565579132754	4.724771517335
H	-2.628582800740	2.213047564321	-4.275568966211
H	-3.017751714310	-0.546560192083	-4.492363251697
C	2.737005798250	-0.746429576335	-1.025757289298
C	3.941965756424	-0.243083089254	-1.439025460200
C	2.647257116488	-1.914770881659	-0.316909163884
C	5.110717349398	-0.961199028696	-1.180822591757
C	3.802062930896	-2.641340394213	-0.029747654545
C	5.035618024108	-2.147573308264	-0.452460835874
F	1.074669058949	0.506611151229	-0.851921278046
F	1.474357182388	-2.404281966353	0.107728585428
F	3.756077717987	-3.767158077560	0.678336852645
F	6.147084869318	-2.809417935414	-0.153585528148
F	6.300649803535	-0.511171724514	-1.570014994394
F	4.045227917656	0.915706765700	-2.093117660126
C	-1.500599103362	2.891949960356	-1.804699123843
C	-0.265185932932	3.465924075519	-2.133157774352
C	-2.418026778115	3.515591329896	-0.948425594109
C	0.026206135987	4.717344161795	-1.584767561804
C	-2.068891353529	4.754980433642	-0.409667818275
C	-0.852903485077	5.371815544126	-0.718868534489
H	0.986388618091	5.181896400688	-1.827115352361
H	-2.769568586806	5.254065974161	0.266023151347

C	-0.476071323780	6.684974924734	-0.097007295081
H	0.109127914675	7.306878697645	-0.790051784377
H	-1.362274334028	7.256386857060	0.214093588953
H	0.144854776924	6.525736464248	0.800901647551
C	-3.717131191528	2.847883283137	-0.609178303013
H	-4.335820122966	3.490018967584	0.031910948510
H	-4.295230970144	2.604078176306	-1.514385288961
H	-3.540491344887	1.896588716498	-0.081445481377
C	0.732980587142	2.717550559662	-2.958501545042
H	1.089316812650	1.850801388846	-2.371182550736
H	0.294483670797	2.330770158033	-3.890942295105
H	1.591816253372	3.352181344690	-3.213898478483
C	-2.155470401671	-1.872703868942	-2.161808072228
C	-3.338150061750	-2.432417394602	-1.665695639130
C	-0.970391569558	-2.608249987469	-2.298442351643
C	-3.307958102160	-3.772303516826	-1.273328581099
C	-0.985605738887	-3.937245679326	-1.871401769254
C	-2.138404012594	-4.533322699205	-1.351719854861
H	-4.222484265161	-4.229824815190	-0.884608295265
H	-0.063938820160	-4.520742011093	-1.945651466176
C	-2.107159178599	-5.950579555057	-0.858539862598
H	-3.117050813027	-6.379587389780	-0.791117716045
H	-1.503194471006	-6.593579395013	-1.515572950287
H	-1.655639943269	-6.002840974781	0.146557888787
C	0.256459039575	-1.971879903051	-2.870305447227
H	1.070272355675	-2.701901456176	-2.967410887306
H	0.053119674759	-1.545838086988	-3.865650715787
H	0.607661508334	-1.143612727221	-2.232025918176
C	-4.578640402517	-1.599884643966	-1.543874332939
H	-4.395589078724	-0.726361657637	-0.898635181679
H	-4.910836199552	-1.217692251447	-2.521770581542
H	-5.401730945056	-2.180494346734	-1.107735055604
C	-2.572496970297	-0.371081964089	2.455442639160
C	-3.526007182153	0.656288611510	2.528249838496

C	-2.903517785028	-1.673976438775	2.063395237314
C	-4.850758263460	0.338633755099	2.230368302896
C	-4.247221173998	-1.944614782901	1.790534764516
C	-5.232324628868	-0.959492723304	1.873149415396
H	-5.606558275622	1.128104425055	2.278621924638
H	-4.525373877179	-2.959208829973	1.493966378352
C	-6.666052057416	-1.268297756878	1.554075405650
H	-7.339837933325	-0.913468482388	2.348985205601
H	-6.977923961485	-0.771312477209	0.620785893489
H	-6.828077196259	-2.347873410053	1.427944861542
C	-1.845310504029	-2.720700442037	1.898574887959
H	-2.289632852196	-3.715523895624	1.766727894647
H	-1.234595659101	-2.497217923331	1.007522584651
H	-1.156472057619	-2.746028668441	2.756043774732
C	-3.109474718766	2.054343500811	2.875989581641
H	-2.687158678221	2.117028220574	3.890940836824
H	-2.326762554861	2.407246669358	2.184283591102
H	-3.961138822432	2.744949638372	2.817684888615
C	2.132823970674	0.790377224675	2.117566488800
C	2.314010946071	2.015910360905	1.462192283861
C	3.189696199465	-0.102659576670	2.336528432302
C	3.597495956020	2.328497471735	1.018378260548
C	4.461282301497	0.269929560421	1.893966315815
C	4.683858053243	1.474502429206	1.225754261835
H	3.751700648791	3.271217207941	0.486038448651
H	5.296468830812	-0.418130723807	2.052445522735
C	6.040483294713	1.830600053011	0.693448750719
H	6.326147179800	2.855853925416	0.974687450743
H	6.814252640703	1.142818046098	1.061525223430
H	6.046203065401	1.776902435782	-0.406291547137
C	2.967904410702	-1.435773968188	2.991673381943
H	3.830972732662	-2.095030638655	2.830714384015
H	2.815852274865	-1.348335474761	4.079408248768
H	2.079816731959	-1.935924326414	2.577720225923

C	1.166350219232	2.943251439042	1.218342476445
H	0.522577386354	2.525075871506	0.429043683229
H	0.543612995190	3.073534467676	2.116630560774
H	1.518234150789	3.929051884392	0.887810870489

202

I16_opt, 1-A (C1): E(RPBE0-D3BJ/def2SVP) = -7532.18608928

G_corr(298K) = 1.521544

E(COSMO-PBE0-D3BJ/def2TZVP) = -7537.40286553

Ni	2.616723092879	0.396105471341	-0.037940882956
N	4.240214343948	-0.175011012686	-2.402761564410
N	4.496179887214	1.914087652920	-2.051659500877
C	3.993446771949	0.782686649530	-1.447739774725
C	4.840630160558	0.340399472019	-3.537495147029
H	5.103422941536	-0.288351230283	-4.382648765570
C	5.003818252654	1.660448021415	-3.316424991885
H	5.440794753819	2.445949649542	-3.925793618307
N	2.557984013948	0.521469477902	2.891060860430
N	3.140157414640	-1.488972825769	2.440194698524
C	2.864910640949	-0.296198888823	1.818966995630
C	2.626867439347	-0.140770855611	4.101206096106
H	2.427895028324	0.350490912498	5.047288878066
C	2.983430504094	-1.410881801694	3.819219584860
H	3.155930528671	-2.269733982344	4.461062934618
C	4.065232122958	-1.586890681099	-2.287380883171
C	4.978173222625	-2.293648078384	-1.492195035768
C	3.129738449028	-2.243952126459	-3.100060910843
C	4.910316568589	-3.686063105322	-1.489252404555
C	3.081661858409	-3.637965594638	-3.037273841351
C	3.958971510385	-4.376752468167	-2.241868809279
H	5.625078583159	-4.242184679763	-0.880486419044
H	2.340411145663	-4.161184359406	-3.649229070467
C	3.876833065906	-5.875345449589	-2.209372506887
H	3.920652971200	-6.302330518257	-3.223629799701

H	4.694558809857	-6.312268964432	-1.620030658500
H	2.926749030078	-6.208689758896	-1.760799562463
C	2.241619042244	-1.501132432260	-4.052306159221
H	1.318067860817	-2.067586355317	-4.234818515973
H	1.969509294032	-0.514590749506	-3.664044453482
H	2.742400177924	-1.362979360409	-5.025775788455
C	6.021849059125	-1.561845190758	-0.706214285934
H	6.626622575712	-0.911956214898	-1.359052199492
H	5.544858834903	-0.910446824917	0.041550527087
H	6.689284622338	-2.263128152663	-0.189975980248
C	4.677325898895	3.218276003772	-1.497759935566
C	5.819292102483	3.450847780470	-0.713721628491
C	3.850621477360	4.273611897223	-1.911472027788
C	6.034178283933	4.739625992433	-0.222817152114
C	4.101364412360	5.545401593208	-1.386929138875
C	5.168350603150	5.795404557480	-0.523003873918
H	6.910020967757	4.924916002299	0.406107529804
H	3.443548789199	6.368778942313	-1.679757564733
C	5.371484359220	7.153930831769	0.080711179463
H	4.853214884381	7.227781564459	1.052087965398
H	6.435761054037	7.361888256042	0.263821364331
H	4.969769877322	7.948121612615	-0.564883729142
C	2.816303286812	4.088955315259	-2.978838543919
H	2.011253754191	4.827844846080	-2.877680484054
H	3.281405488853	4.235704491881	-3.968928960328
H	2.376839383299	3.087344104634	-2.958636838356
C	6.827137895695	2.364160076439	-0.482855003014
H	6.351003886447	1.406872934941	-0.235645748528
H	7.422291279090	2.197288090244	-1.396206347221
H	7.520154228972	2.631793827946	0.326219362226
C	2.270417852788	1.907407217309	2.748379479698
C	1.094953508299	2.479342922475	3.259664084982
C	3.235156781236	2.694693873154	2.102820058424
C	0.795097377670	3.784607933891	2.864366529565

C	2.881596416848	3.984438187464	1.710120868348
C	1.631324443913	4.520417107854	2.022862094226
H	-0.142623278059	4.227147703139	3.208496802576
H	3.602386532178	4.578454733050	1.145400810502
C	1.187991189721	5.834007629118	1.453977280032
H	0.554523341447	6.395414379324	2.157004870832
H	2.042687006091	6.464874856431	1.169229323474
H	0.593352359998	5.648064731010	0.544096786718
C	4.632800543938	2.183492343876	1.936594536990
H	5.330727488910	3.022187660486	1.836225307114
H	4.929080641882	1.580870654621	2.808483891621
H	4.714999034168	1.536315019100	1.052011297046
C	0.226824268310	1.787240739466	4.267748337420
H	0.156807131395	0.709517132977	4.090739794483
H	0.633067449018	1.942284280213	5.282476703330
H	-0.791024674517	2.194530549018	4.254117277255
C	3.758423743096	-2.680227057126	1.939530070737
C	2.992906762227	-3.711079935201	1.379858304833
C	5.120506851551	-2.859508033263	2.240949946103
C	3.607068422940	-4.953737345490	1.194797492854
C	5.693486475868	-4.110929729246	2.013219690709
C	4.943934597481	-5.181336589842	1.517036778827
H	3.007865475748	-5.771258245497	0.784954907463
H	6.751813066736	-4.256781955691	2.250510433422
C	5.580990524690	-6.522012543180	1.294039318628
H	6.188748726258	-6.828859913394	2.158838030712
H	4.827666491455	-7.300963218485	1.110956176785
H	6.254763501888	-6.501081462729	0.420493703585
C	5.935096060784	-1.741978952204	2.824543522846
H	7.008203765617	-1.920625156499	2.670331990011
H	5.671298024474	-0.776212270247	2.370565687262
H	5.764223434680	-1.639706065890	3.908443455619
C	1.575172666483	-3.491056140609	0.978257902336
H	1.029466751127	-2.872109732656	1.698735022388

H	1.537460692889	-2.940298805607	0.026026563641
H	1.040311406290	-4.441701740300	0.848145599467
Ni	-2.554719731700	-0.075626222170	-0.126721137294
N	-3.486398787219	-0.958679330668	2.483522098992
N	-4.232220985436	1.020080282058	2.177235003244
C	-3.613579775988	-0.001335573216	1.504691303636
C	-3.983905383600	-0.533713699750	3.703315309364
H	-3.969750607586	-1.172165517624	4.581355992405
C	-4.455769805953	0.715595250166	3.512158019611
H	-4.945577874101	1.413328097585	4.184754616159
C	-1.116044679732	0.852030012978	0.693081572962
C	-0.649419886646	2.123836444510	0.136561908633
C	0.442562547049	2.236783635258	-0.644084616960
C	1.218043762811	1.094827101440	-1.132199337171
F	-1.273132774929	3.243189150293	0.533071997951
F	0.786338354092	3.451540854825	-1.099391276886
F	1.466644111366	1.206412149158	-2.503158864855
C	-0.487450214749	-0.292725253048	0.097578108954
C	0.766785843971	-0.172258630174	-0.627854276119
F	-0.506301133716	-1.418206933458	0.902656524186
F	1.016916860989	-1.243907313848	-1.461263464253
N	-3.708142758349	0.136208046615	-2.758845094770
N	-3.596966307544	-1.973435877541	-2.457536846895
C	-3.355929973186	-0.785776347276	-1.804065295179
C	-4.098161296138	-0.438332206939	-3.950934936113
H	-4.371886543554	0.156994056183	-4.817027401339
C	-4.025570764966	-1.772923281616	-3.764312976899
H	-4.225783293395	-2.605339512699	-4.431857040519
F	-1.100696416581	0.933188782800	2.097443466746
C	-3.047583104704	-2.309783198720	2.331088024259
C	-3.902404132607	-3.206756831714	1.674079249841
C	-1.880014137877	-2.744524117999	2.972350543402
C	-3.530468263889	-4.548356205386	1.608800090561
C	-1.545422085381	-4.096839021905	2.871795311334

C	-2.346120173127	-5.010956361397	2.187821148702
H	-4.192996281885	-5.249394922501	1.096993859539
H	-0.624382369345	-4.442744601130	3.349628102522
C	-1.939159222969	-6.449754366031	2.065298116962
H	-1.205717544889	-6.729702350602	2.834924148004
H	-2.803995705011	-7.124437897308	2.152986660854
H	-1.478396109370	-6.641438101357	1.081990373138
C	-1.019420542453	-1.807033259045	3.761277457817
H	-0.937578559790	-0.841575967414	3.251303806488
H	-1.435244897446	-1.627696649994	4.767026867338
H	-0.009434255400	-2.219604003232	3.891398311354
C	-5.201768890798	-2.730033549433	1.104954213499
H	-5.786735002926	-2.183096233462	1.861068479965
H	-5.022181101971	-2.031357477670	0.274351502383
H	-5.801680706428	-3.571896885989	0.738499220929
C	-4.810975139698	2.209445909067	1.640778783608
C	-6.063608058062	2.097373612253	1.015729198784
C	-4.248189074047	3.455542680636	1.939568094948
C	-6.725395472416	3.267792640672	0.648984836032
C	-4.932865657839	4.600235417262	1.517979342356
C	-6.168009451154	4.530354742687	0.875479464735
H	-7.713001896511	3.192889202158	0.183880821569
H	-4.493441073001	5.579226937204	1.730459189059
C	-6.875517968215	5.773670562608	0.422183010864
H	-7.967774840129	5.669196638722	0.500315710473
H	-6.569979297624	6.650612289518	1.010503846487
H	-6.646014410237	5.995255519313	-0.634552190931
C	-3.014207333847	3.576284231917	2.779101510293
H	-2.460404764234	4.489550904329	2.525600535513
H	-3.287232486898	3.636287010348	3.846831312611
H	-2.346547212300	2.719025321641	2.645132730446
C	-6.685556903113	0.748005707556	0.823683511257
H	-6.017629753484	0.092061171995	0.247041068167
H	-6.850760216610	0.252114921479	1.794055999215

H	-7.651103935411	0.822887285849	0.306466399031
C	-3.614465432445	1.544687293709	-2.571688700925
C	-2.367497287131	2.147796678791	-2.763866004251
C	-4.765150777814	2.287300699159	-2.292174704146
C	-2.248858801387	3.506132276524	-2.478122756980
C	-4.605500612227	3.650413171548	-2.034444351406
C	-3.350438229646	4.261734261897	-2.066641303460
H	-1.266538669739	3.977856751018	-2.559717708041
H	-5.486972450569	4.245407480658	-1.788728450927
C	-3.174124219031	5.690219949097	-1.646699052296
H	-2.465514219451	6.222548253222	-2.298787603217
H	-4.127216640041	6.238449864128	-1.646702975801
H	-2.766743505451	5.726270816777	-0.622571302523
C	-6.119507831372	1.649492550525	-2.359737211796
H	-6.875500525433	2.269621488463	-1.863552678696
H	-6.432424935497	1.517812994356	-3.409122688839
H	-6.122921108332	0.652505389848	-1.900161677092
C	-1.237993081661	1.336764664095	-3.305601287049
H	-0.959948496387	0.533277117349	-2.608068130518
H	-1.526149040212	0.854466446755	-4.253618305702
H	-0.355540787956	1.955443938420	-3.485453214297
C	-3.554098310465	-3.319648059941	-1.978225679733
C	-2.326974760638	-3.962615075326	-1.769469583913
C	-4.771533288201	-4.022008433390	-1.908029951092
C	-2.343643163883	-5.335282853934	-1.502507256102
C	-4.733090346052	-5.389747216223	-1.636030018749
C	-3.526967799743	-6.069540022588	-1.443889812860
H	-1.386561108099	-5.844413820828	-1.356723433562
H	-5.676534021892	-5.941765042234	-1.582232185386
C	-3.519381672181	-7.542080581353	-1.154305181463
H	-4.177179333535	-8.091176407976	-1.845126347052
H	-2.508045745307	-7.964292308508	-1.236764551333
H	-3.880337440566	-7.747088172914	-0.132782913302
C	-6.089572892603	-3.340832640816	-2.145125353225

H	-6.898197265881	-3.872014039961	-1.623615194338
H	-6.077065621945	-2.298575457311	-1.797807831928
H	-6.348988319483	-3.318019058965	-3.215713659500
C	-1.041324668166	-3.212671467047	-1.828521815906
H	-0.879072231256	-2.672698491356	-0.886676909831
H	-0.187048694870	-3.885899504635	-1.980746844096
H	-1.039579467852	-2.450945083485	-2.619181873723

40

I17_opt, (C2): E(RPBE0-D3BJ/def2SVP) = -2795.35822118

G_corr(298K) = 0.241303

E(COSMO-PBE0-D3BJ/def2TZVP) = -2797.02978219

Ni	0.000000000192	0.000000000131	0.467214929120
N	0.802981940838	-0.709268953211	-2.247031553207
N	-0.802981941066	0.709268953221	-2.247031553162
C	-0.000000000029	0.000000000054	-1.410008754821
C	0.513150414932	-0.445497880692	-3.569447964853
H	1.043685720806	-0.902179387784	-4.399029847666
C	-0.513150414652	0.445497881338	-3.569447964828
H	-1.043685720333	0.902179388706	-4.399029847617
C	-1.233948939679	0.689142314034	1.952294425410
C	-0.019857599805	1.413020393458	1.949308591857
C	1.214639887109	0.723485989711	1.951888300970
C	1.233948940437	-0.689142313676	1.952294425200
F	-0.042888359626	2.742401773668	1.973290131615
F	2.357970835177	1.401678393951	1.971722348735
F	2.394925665864	-1.340219258010	1.971971357915
C	-1.214639886333	-0.723485989353	1.951888301216
C	0.019857600572	-1.413020393113	1.949308591963
F	-2.357970834400	-1.401678393592	1.971722349277
F	0.042888360402	-2.742401773311	1.973290131818
C	-1.881754324386	1.560180643578	-1.746360993465
H	-1.506744796463	1.926011558057	-0.777485164604
C	-3.134577880468	0.736476104630	-1.487372685043

C	-2.127575753185	2.748209240228	-2.658898862461
H	-1.196667895881	3.299333248367	-2.857644877437
H	-2.568892655818	2.445358732841	-3.621725902593
H	-2.837117097156	3.437238285515	-2.179334047340
H	-3.517304971636	0.293768074636	-2.420653233972
H	-3.923901147306	1.366350649089	-1.051364622076
H	-2.912535387801	-0.073975483403	-0.778220171213
C	1.881754323764	-1.560180643980	-1.746360993406
H	1.506744795273	-1.926011558822	-0.777485164900
C	3.134577879766	-0.736476105272	-1.487372683883
C	2.127575752960	-2.748209240280	-2.658898862773
H	1.196667895704	-3.299333248201	-2.857644878552
H	2.568892656227	-2.445358732539	-3.621725902500
H	2.837117096541	-3.437238285888	-2.179334047530
H	3.517304971515	-0.293768074872	-2.420653232385
H	3.923901146279	-1.366350650033	-1.051364620763
H	2.912535386767	0.073975482442	-0.778220169781
F	-2.394925665103	1.340219258375	1.971971358343

60

I18_opt, 1-A1 (C2V): E(RPBE0-D3BJ/def2SVP) = -3256.64445716

G_corr(298K) = 0.380893

E(COSMO-PBE0-D3BJ/def2TZVP) = -3258.79051818

Ni	-0.000000000000	-0.000000000000	0.631595013340
N	0.000000000000	1.068527002000	-2.063294555660
N	-0.000000000000	-1.068527002000	-2.063294555660
C	-0.000000000000	0.000000000000	-1.223639993660
C	0.000000000000	0.679685996000	-3.391978653660
H	0.000000000000	1.396223495000	-4.208496213660
C	-0.000000000000	-0.679685996000	-3.391978653660
H	-0.000000000000	-1.396223495000	-4.208496213660
C	0.000000000000	-2.402241008000	-1.557825533660
C	-1.226143565000	-3.017938007000	-1.276398017660
C	1.226143565000	-3.017938007000	-1.276398017660

C	-1.200545813000	-4.301336004000	-0.729263239660
C	1.200545813000	-4.301336004000	-0.729263239660
C	0.000000000000	-4.958499501000	-0.448674976660
H	-2.149148114000	-4.793760502000	-0.497444228660
H	2.149148114000	-4.793760502000	-0.497444228660
C	0.000000000000	2.402241008000	-1.557825533660
C	-1.226143565000	3.017938007000	-1.276398017660
C	1.226143565000	3.017938007000	-1.276398017660
C	-1.200545813000	4.301336004000	-0.729263239660
C	1.200545813000	4.301336004000	-0.729263239660
C	0.000000000000	4.958499501000	-0.448674976660
H	-2.149148114000	4.793760502000	-0.497444228660
H	2.149148114000	4.793760502000	-0.497444228660
C	2.514991399000	-2.279640009000	-1.476247781660
H	2.608169986000	-1.487018993000	-0.714859805660
H	3.377264853000	-2.952232247000	-1.376912021660
H	2.565084683000	-1.787268038000	-2.458722531660
C	0.000000000000	-6.315796491000	0.191624053340
H	-0.891208327000	-6.895735534000	-0.088188459660
H	0.891208327000	-6.895735534000	-0.088188459660
H	0.000000000000	-6.223354892000	1.290552605340
C	-2.514991399000	-2.279640009000	-1.476247781660
H	-3.377264853000	-2.952232247000	-1.376912021660
H	-2.608169986000	-1.487018993000	-0.714859805660
H	-2.565084683000	-1.787268038000	-2.458722531660
C	0.000000000000	6.315796491000	0.191624053340
H	-0.891208327000	6.895735534000	-0.088188459660
H	0.000000000000	6.223354892000	1.290552605340
H	0.891208327000	6.895735534000	-0.088188459660
C	2.514991399000	2.279640009000	-1.476247781660
H	3.377264853000	2.952232247000	-1.376912021660
H	2.608169986000	1.487018993000	-0.714859805660
H	2.565084683000	1.787268038000	-2.458722531660
C	-2.514991399000	2.279640009000	-1.476247781660

H	-2.565084683000	1.787268038000	-2.458722531660
H	-2.608169986000	1.487018993000	-0.714859805660
H	-3.377264853000	2.952232247000	-1.376912021660
C	-1.408736936000	0.000000000000	2.102898404340
C	-0.706350470000	1.228227854000	2.113345652340
C	-0.706350470000	-1.228227854000	2.113345652340
C	0.706350470000	1.228227854000	2.113345652340
C	0.706350470000	-1.228227854000	2.113345652340
C	1.408736936000	-0.000000000000	2.102898404340
F	2.740708886000	-0.000000000000	2.101192903340
F	1.376256443000	2.375689692000	2.144311896340
F	-1.376256443000	2.375689692000	2.144311896340
F	-2.740708886000	0.000000000000	2.101192903340
F	-1.376256443000	-2.375689692000	2.144311896340
F	1.376256443000	-2.375689692000	2.144311896340

72

I19_opt, 1-A (C1): E(RPBE0-D3BJ/def2SVP) = -4082.84398950

G_corr(298K) = 0.429722

E(COSMO-PBE0-D3BJ/def2TZVP) = -4085.95313558

Ni	0.044841348147	0.419445739460	0.057941441160
N	1.216656511631	-1.785543751304	-1.607873626918
N	-0.919850406392	-1.810657163522	-1.682250502859
C	0.122854553500	-1.158821699381	-1.093722673570
C	0.868578262834	-2.791015062166	-2.490154867080
H	1.614721248576	-3.401051706576	-2.990861329592
C	-0.485442543929	-2.808501094609	-2.535635977836
H	-1.179783275683	-3.439134945126	-3.083100604946
C	-2.298762076044	-1.605754192787	-1.361236181183
C	-3.134236766797	-0.938996634568	-2.264371114774
C	-2.780735173688	-2.141675413321	-0.157206530377
C	-4.464025618088	-0.737419750799	-1.887946571169
C	-4.110192720343	-1.896089670654	0.183307465512
C	-4.963414936006	-1.180866690140	-0.661941798269

H	-5.125370471136	-0.199223425901	-2.572656092903
H	-4.488630438606	-2.280874261402	1.133755852591
C	2.550681529446	-1.570062957259	-1.139856111255
C	3.419691074780	-0.722411677560	-1.834220750760
C	2.952547925370	-2.280449825051	0.002559390455
C	4.674679921492	-0.483902760326	-1.266821656251
C	4.212541602716	-2.007435231506	0.529481014627
C	5.075003487700	-1.086092707559	-0.073220581988
H	5.358263278511	0.199174241804	-1.778640616133
H	4.527889228890	-2.528629316677	1.437144970942
C	-1.907257247331	-3.007355225898	0.697822272243
H	-0.923617450461	-2.546495632551	0.850853317991
H	-2.360772083142	-3.192685250958	1.678392875400
H	-1.732069265326	-3.981117236915	0.211219734527
C	-6.374347629023	-0.881759941607	-0.249570393342
H	-7.021432984151	-0.700854588964	-1.119518044517
H	-6.806466426628	-1.704346450542	0.338675710103
H	-6.407751028639	0.022294226372	0.380914223476
C	-2.625930553518	-0.456354512255	-3.589538132260
H	-3.350699442459	0.221683556106	-4.059261269772
H	-1.672904719521	0.076454490855	-3.483326814442
H	-2.457361286118	-1.295929978975	-4.283651034042
C	6.394574026092	-0.750422012147	0.555915888042
H	7.111571184336	-0.367245390158	-0.183903359943
H	6.264982489828	0.029092889163	1.325023630543
H	6.841162054685	-1.625059840919	1.051025373416
C	2.074012030853	-3.347630984493	0.584110474477
H	2.514018503581	-3.769868197684	1.494569364159
H	1.075355772587	-2.966697541370	0.836889661470
H	1.925097272030	-4.164538518298	-0.140166733618
C	3.061998800319	-0.157246468674	-3.175785932974
H	2.002891694635	0.113496895653	-3.243888511347
H	3.660534085175	0.735275208552	-3.398612767431
H	3.266531827400	-0.900977787380	-3.964515258728

C	-0.037487206380	1.074123669293	1.861618744150
C	1.024920626388	0.101601877378	1.878646903206
C	-1.331228856938	0.564276159978	2.220471732825
C	0.884575358248	-1.079862133437	2.659006970240
C	-1.480377917985	-0.661362985114	2.823966433969
C	-0.357758493889	-1.483592684680	3.078479864154
F	-0.538514570508	-2.650559610571	3.685682377611
F	1.963975913080	-1.782564751524	2.967555165556
F	2.275955392913	0.575938677545	1.757549443584
F	0.237816520534	2.351214373731	2.222663699804
F	-2.377857741828	1.371098429896	2.092681823812
F	-2.685068226613	-1.124582672693	3.131967883117
C	1.123017430261	3.587684779561	-0.234657924999
F	2.189589316443	4.270325999399	0.138317417170
C	-0.142786725605	4.004703143710	0.209931022677
F	-0.235492638716	5.071191481585	0.979710949994
C	-1.261348260739	3.265861529736	-0.109969276139
F	-2.456354004187	3.645045540612	0.299366523280
C	-1.128516093332	2.067316055296	-0.842607741329
F	-2.245580935917	1.563258849545	-1.361710332134
C	0.120553577192	1.694583147752	-1.419452456617
F	0.136767771710	1.187951510163	-2.674271614265
C	1.259212345578	2.450102930094	-1.004421932760
F	2.447771797318	2.148921994528	-1.504594576543

67

I1_opt, 1-A (C1): E(RPBE0-D3BJ/def2SVP) = -3256.64407812

G_corr(298K) = 0.469836

E(COSMO-PBE0-D3BJ/def2TZVP) = -3258.80356666

Ni	-0.180851126249	0.042948886546	-0.318365321958
N	1.509624785953	2.081281307668	1.035442066252
N	-0.277472259363	2.915472720428	0.205060357967
C	0.371637416606	1.735112038757	0.377580342219
C	1.570605825922	3.442650370060	1.260044148050

H	2.399476896996	3.922522900155	1.772625853717
C	0.440212910442	3.973283638914	0.726681893006
H	0.100585654113	5.003801551415	0.680891058504
C	0.110035878260	-1.352854006510	-1.546719076663
C	0.806865006081	-2.412192102960	-0.866230988813
C	2.153674371998	-2.392525009441	-0.663204570425
C	2.957900560596	-1.336493113598	-1.201448058179
F	0.075023263731	-3.430392650229	-0.402253946400
F	2.748908367389	-3.295595003907	0.116972270090
F	4.276509615792	-1.381183679416	-0.999180665196
C	0.927023427624	-0.178568164669	-1.890325782494
C	2.363254786335	-0.309800415202	-1.866075939517
F	0.470559060294	0.567733705316	-2.945593574565
F	3.094302391963	0.649442197283	-2.432361729206
N	-2.942101754372	-0.882525504676	0.230008199711
N	-1.659280732812	-1.126056870876	1.924884983174
C	-1.674368521366	-0.643812847586	0.655297110044
C	-3.696117343095	-1.501708200031	1.207185363557
H	-4.736324516512	-1.780715747062	1.065788893598
C	-2.881157381198	-1.661463633094	2.280846346214
H	-3.074158957618	-2.110660261050	3.250757036866
C	-1.518459614794	3.006303229987	-0.555233219498
H	-1.880587382315	1.965711713541	-0.567475026789
C	-1.239615083586	3.431798104698	-1.988804837794
C	-2.539535570754	3.881477996974	0.150923611099
H	-2.707358361033	3.538144114690	1.182445772293
H	-2.225365072814	4.936820424048	0.181888585914
H	-3.498461627913	3.845097295970	-0.386239542501
H	-0.816391603011	4.448381146307	-2.027271004725
H	-2.170372257206	3.428482425627	-2.575908802772
H	-0.531392709780	2.733804107385	-2.457247170526
C	2.546155046325	1.121907424205	1.405083053010
H	2.162371267741	0.165556793591	1.015297436244
C	2.692654563257	1.029658647560	2.916360089292

C	3.861780610320	1.435805475231	0.710319812307
H	3.720563862082	1.533435755531	-0.373411835539
H	4.305853849999	2.368403557899	1.093674058491
H	4.579121626584	0.621158206687	0.882636367069
H	3.090097811963	1.966561958097	3.337892756580
H	3.391828557296	0.222630595548	3.179895027858
H	1.726275372698	0.824612201998	3.397054619182
C	-3.413999406324	-0.558048176937	-1.113138125263
H	-2.516283258194	-0.170820830519	-1.619217044364
C	-4.482652618081	0.523950087610	-1.065234419283
C	-3.868150808544	-1.810918238926	-1.844424968599
H	-3.062218255931	-2.555944633275	-1.850049376485
H	-4.766022168996	-2.249605489985	-1.380388517715
H	-4.114765837723	-1.566442103278	-2.887766234683
H	-5.398214705034	0.163620515080	-0.569644941523
H	-4.754860979132	0.834677533792	-2.084643467772
H	-4.126039594768	1.407583086134	-0.516607756290
C	-0.461505826060	-1.112238676810	2.755738039502
H	0.235524922354	-0.489518940130	2.173582168378
C	0.131959485525	-2.505670024545	2.893270618684
C	-0.734894037207	-0.439855132195	4.091526575386
H	-1.130396229052	0.576023333204	3.944730379896
H	-1.459237688844	-1.010365706765	4.694066952081
H	0.192543705097	-0.370871196579	4.678453089549
H	-0.547980932259	-3.178233032720	3.440447730179
H	1.080197482325	-2.460007252499	3.449325404215
H	0.330645139770	-2.943998000713	1.906307548461
F	-0.853084345997	-1.769383684844	-2.433752615540

60

I20_opt, 1-A (C1): E(RPBE0-D3BJ/def2SVP) = -3256.64633437

G_corr(298K) = 0.380845

E(COSMO-PBE0-D3BJ/def2TZVP) = -3258.81034019

Ni	-0.003428493503	0.198475167219	-0.023820589323

N	-2.539955117123	-1.477522435021	-0.072932588531
N	-0.716096152397	-2.592340629154	-0.149915432168
C	-1.199789873290	-1.339244215762	-0.011475863218
C	-2.894499630172	-2.801331695902	-0.254645681783
H	-3.931866776569	-3.114106571981	-0.333333932261
C	-1.733858551404	-3.510702515249	-0.303242406034
H	-1.544646904714	-4.572473127798	-0.433310964994
C	0.693071044542	-2.846741070756	-0.123118062640
C	1.302581923647	-3.088782580941	1.115254876333
C	1.417389542748	-2.758498877154	-1.319287958895
C	2.688419170528	-3.238252064902	1.137670194763
C	2.802552842835	-2.911905074612	-1.239530854775
C	3.455808461972	-3.133696811934	-0.025479861033
H	3.185708291802	-3.410636159715	2.096095280634
H	3.391184616566	-2.826106583342	-2.156950867172
C	-3.460138221197	-0.388577825822	0.053744195655
C	-3.963397881319	-0.086397919452	1.325144016990
C	-3.809234109542	0.339217746876	-1.091804480709
C	-4.857266983714	0.980301391348	1.432902436911
C	-4.706541745590	1.396356527489	-0.928631866470
C	-5.238819994592	1.733087039998	0.319015483047
H	-5.261149845125	1.233238772859	2.417146053906
H	-4.990627735972	1.980224461576	-1.808497544202
C	0.736384544876	-2.431591913117	-2.612097962723
H	0.387466660965	-1.383879155271	-2.582718756119
H	1.425302278914	-2.552073315110	-3.458330969237
H	-0.142709902879	-3.070749437248	-2.787055084031
C	4.952666256760	-3.177813987328	0.041169336787
H	5.342634021679	-2.160058574112	0.210884305603
H	5.306966468049	-3.810036532556	0.867885667012
H	5.392501705848	-3.548856576739	-0.895407656416
C	0.491840019781	-3.134153171361	2.377160788666
H	1.119729632805	-3.399061247656	3.237773035798
H	0.030996483669	-2.154524122224	2.586037601769

H	-0.330906403616	-3.863099311499	2.314372517777
C	-6.170077465847	2.901206803086	0.460950613346
H	-6.767233565645	2.834320987727	1.381217704251
H	-5.603371551131	3.846134917275	0.501361848454
H	-6.859043448840	2.972641661626	-0.393352079010
C	-3.200120046762	0.017578413351	-2.420645324815
H	-3.654902435626	0.621156896674	-3.216865268948
H	-2.115231591073	0.223309045501	-2.386630021440
H	-3.325665824673	-1.045924493282	-2.678390109175
C	-3.532959815965	-0.870281659498	2.530055604052
H	-3.766929719893	-1.941742919514	2.428873912786
H	-2.443738793414	-0.797273901312	2.680692042545
H	-4.026166602143	-0.498602112434	3.437678971080
C	2.101499632265	3.851244646898	-0.043425722227
C	1.086612400422	2.897943690086	-0.095774826543
C	3.410739089021	3.438794621508	0.204862867944
C	1.322874708966	1.549766995818	0.138419837357
C	3.686432212120	2.091953502252	0.432178772676
C	2.639001442290	1.177018722091	0.372468555213
F	2.943050247636	-0.113193540379	0.581458595377
F	-0.197986904962	0.429336344177	-1.762926486089
F	-0.146327133109	3.334658552071	-0.348875449699
F	1.851776079232	5.138991223687	-0.244254787049
F	4.387269780805	4.331721544492	0.251623424967
F	4.933667242116	1.704143860794	0.674832114724

67

I2_opt, 1-A (C1): E(RPBE0-D3BJ/def2SVP) = -3256.69193152

G_corr(298K) = 0.470750

E(COSMO-PBE0-D3BJ/def2TZVP) = -3258.86837957

Ni	0.196410573153	-0.264453861634	-0.571582838363
N	2.791461428060	-1.557533787927	-0.824287279521
N	3.056084327639	0.564555369202	-0.947611258777
C	2.110935705947	-0.388775999359	-0.776041166337

C	4.138314147482	-1.339711900106	-1.020626349844
H	4.861195385895	-2.146578121316	-1.098228806768
C	4.310543295133	0.005631632115	-1.097420924909
H	5.208985740764	0.595070208822	-1.255048610107
C	-1.715856778510	-0.395780507720	-0.394767926427
C	-2.587410152849	0.108036778568	-1.347718446627
C	-3.966171639267	-0.081992973308	-1.295304404004
C	-4.505646433672	-0.856873077601	-0.270060562298
F	-2.129491250624	0.891740835737	-2.337852186190
F	-4.773310918329	0.452976717136	-2.205933268771
F	-5.815414716466	-1.062320168516	-0.206161740676
C	-2.292010450101	-1.165142633776	0.600599649407
C	-3.663177909980	-1.394438171979	0.699902080203
F	-1.520598422073	-1.717019775227	1.560133825727
F	-4.174071777183	-2.131590774772	1.681471311876
N	-0.215614064283	2.134280922921	1.036317240438
N	0.788284152009	0.591920746644	2.137988254657
C	0.259642722741	0.868320958337	0.921037737194
C	0.011968528339	2.637759089092	2.301119270935
H	-0.299926322341	3.632736621950	2.604558349112
C	0.648710032557	1.662922198418	2.998464203009
H	0.999681855795	1.644951174369	4.025854082945
C	2.755263533394	1.990731292659	-1.014552345455
H	1.657606091890	2.020080114669	-0.983730251188
C	3.214601041575	2.581471105782	-2.338000927329
C	3.305708701729	2.724099060782	0.198701198300
H	2.900451281295	2.301326622454	1.129255185811
H	4.404906382577	2.664825032612	0.238879110076
H	3.030210730455	3.788151287950	0.159471523601
H	4.312327008366	2.582078049202	-2.427618946042
H	2.875152524951	3.624329364527	-2.422004386573
H	2.796055856215	2.012952451073	-3.180767438700
C	2.155395321378	-2.880213661388	-0.785101333098
H	1.096363358193	-2.659708061067	-0.592085264412

C	2.747646226912	-3.736773260172	0.321007322194
C	2.215307824063	-3.523941509621	-2.159877182644
H	1.692370604396	-2.866177133125	-2.866466956211
H	3.252337788025	-3.699436965061	-2.490185659426
H	1.695363653968	-4.492930037969	-2.139938224075
H	3.805024675085	-3.984026652178	0.133117134196
H	2.195408083017	-4.685020873207	0.388026159498
H	2.681548029015	-3.236360014887	1.298064037125
C	-0.977656009812	2.818820960469	-0.009101162031
H	-0.921531680083	2.140329190563	-0.871688308604
C	-0.346853286999	4.152806093100	-0.375918530314
C	-2.436510423532	2.957544562479	0.401001255230
H	-2.841207219641	1.986997147834	0.718014022982
H	-2.557294331819	3.678150280578	1.225811472325
H	-3.032190200876	3.310810581161	-0.452833111292
H	-0.372724294838	4.862901168776	0.465811853002
H	-0.902650781351	4.606426169002	-1.208823667138
H	0.699077618756	4.033051974183	-0.691118822544
C	1.398636651675	-0.689998001429	2.488344841524
H	1.170446499484	-1.329692373045	1.623755898807
C	0.738281579704	-1.287720350423	3.719967590377
C	2.906374313941	-0.545557495077	2.630558344767
H	3.359102044855	-0.166082565815	1.703701148900
H	3.164236069968	0.142791087696	3.451228124818
H	3.362184614354	-1.519952740153	2.858832165615
H	0.949326310825	-0.696809804235	4.625387943860
H	1.125648967414	-2.302589218828	3.891004115010
H	-0.348107973125	-1.353973635245	3.578063706381
F	0.077708798583	-1.383100178853	-2.005891297003

67

I3_opt, 1-A (C1): E(RPBE0-D3BJ/def2SVP) = -3256.64366792

G_corr(298K) = 0.467651

E(COSMO-PBE0-D3BJ/def2TZVP) = -3258.80280246

Ni	0.548901105705	-0.027805157138	-0.255770801068
C	-1.255845162776	0.499862312894	-0.083651840727
F	1.179821851391	2.054266616307	-2.059648162413
C	1.423902676852	1.489273440334	-0.833384407810
C	0.320547307294	-1.840537373959	0.350949235178
N	2.383599388311	0.381770009341	-0.846985182976
C	3.420648459047	0.718084733451	0.073409364870
N	1.988429092763	2.439648443601	0.053876054729
C	3.132431814109	1.920934907955	0.611089539754
N	0.829199442609	-2.362954010202	1.491978581622
C	0.385308544963	-3.653070931971	1.698109649579
N	-0.448810057821	-2.831346461829	-0.158825904498
C	-0.426545850581	-3.950150809326	0.648977161348
H	3.672543707652	2.450916297603	1.393772880617
H	4.270763182369	0.064700146934	0.244968541513
H	0.670941289295	-4.250591611774	2.559184018109
H	-0.980748656831	-4.856596130674	0.422451540905
C	-2.060670999562	0.710007296064	-1.196525827117
C	-3.415571307507	1.023747635025	-1.120787618055
C	-4.014263356725	1.142742955797	0.132159196610
C	-3.249534344720	0.936052691769	1.278683734756
C	-1.900989447133	0.616253846358	1.139008153656
F	-3.816820918038	1.042691975910	2.475875428004
F	-5.303050053825	1.445699516773	0.232701290330
F	-4.147889770855	1.199519563320	-2.215877972754
F	-1.546447941221	0.564918144592	-2.431387638349
F	-1.213904600212	0.409354158561	2.282935255821
C	2.732639429322	-0.249408658493	-2.135128001545
H	1.789590507239	-0.271227538106	-2.703492410403
C	1.181559442953	3.516243802248	0.607191773044
H	0.296511565959	3.559521568849	-0.046384289589
C	1.720123881501	-1.606769221027	2.368208372911
H	1.770322813372	-0.613856184114	1.892584812208
C	-1.228458408053	-2.681608953192	-1.386219890159

H	-0.985209535422	-1.667011964417	-1.731703355430
C	0.714352012773	3.206036164616	2.024217940160
H	0.023251989363	3.982727415183	2.384726187844
H	0.193824060619	2.239749269275	2.060144912929
H	1.567576548179	3.168548763159	2.721102213281
C	1.916863610080	4.845188407475	0.506002759063
H	2.214514973010	5.043301527334	-0.533553910490
H	1.276783732412	5.669962261592	0.852963740417
H	2.825707601258	4.846962693804	1.129651449767
C	3.191949539112	-1.678781391075	-1.904571432926
H	2.435297584127	-2.242302191312	-1.340059921383
H	3.360826547467	-2.182015161049	-2.867806684718
H	4.141009450854	-1.713247947089	-1.345970536879
C	3.746775649309	0.579680181695	-2.910504381358
H	3.931880817442	0.127270000327	-3.896129389544
H	3.372546636364	1.600792092724	-3.062867465997
H	4.706294598679	0.634848592450	-2.373605830631
C	-2.718697948554	-2.739444074763	-1.089784385746
H	-2.983724388633	-2.006897134545	-0.314056166582
H	-3.290964236560	-2.496390239926	-1.996772144759
H	-3.029003738993	-3.740654587362	-0.750044691303
C	-0.781178757387	-3.681921966686	-2.439985788877
H	0.298626251687	-3.593036329921	-2.630627550196
H	-0.993468021914	-4.719085224838	-2.134727562096
H	-1.313674933847	-3.496261688938	-3.383931191383
C	1.120504150073	-1.453659864091	3.755994570319
H	1.756036268751	-0.797715844965	4.368713827786
H	0.119824982149	-1.005976664781	3.691270177764
H	1.044547252444	-2.422778645795	4.274621296115
C	3.113589023747	-2.214468515617	2.373934161599
H	3.803034455383	-1.575153447968	2.944408225531
H	3.117827807461	-3.213568012650	2.838473509762
H	3.498536013916	-2.309985017587	1.348237317717

56

I4_opt, 2-A (C2): E(UPBE0-D3BJ/def2SVP) = -2530.11844339

G_corr(298K) = 0.425268

E(COSMO-PBE0-D3BJ/def2TZVP) = -2531.44348269

Ni	0.000000000000	0.000000000000	0.305157239274
N	0.241568579968	2.929524176071	0.807296299660
N	-0.518530350060	2.524832753063	-1.153100020037
C	-0.076475093995	1.905972805967	-0.023300350724
C	0.000000000000	4.151178856183	0.217345860921
H	0.181190843520	5.096755856157	0.720149810201
C	-0.476762493241	3.897764651898	-1.030354064959
H	-0.784094279868	4.578537907513	-1.819348433015
N	-0.241568579968	-2.929524176071	0.807296299660
N	0.518530350060	-2.524832753063	-1.153100020037
C	0.076475093995	-1.905972805967	-0.023300350724
C	-0.000000000000	-4.151178856183	0.217345860921
H	-0.181190843520	-5.096755856157	0.720149810201
C	0.476762493241	-3.897764651898	-1.030354064959
H	0.784094279868	-4.578537907513	-1.819348433015
C	-0.955362199291	1.786166750549	-2.329744663192
H	-0.942364894499	0.738334718650	-1.982767009541
C	0.035292029281	1.942851930503	-3.473223340652
C	-2.379794692992	2.149434739604	-2.715896258626
H	-3.058130385710	2.014798818013	-1.861070447937
H	-2.456279723768	3.194046484605	-3.057518921434
H	-2.725361354598	1.505299473207	-3.537930714692
H	0.085948362617	2.986705718273	-3.821619556633
H	-0.262783716329	1.317544638806	-4.327972815837
H	1.043967208096	1.641159867838	-3.153640647040
C	0.696472238348	2.733406556799	2.187628835839
H	0.926303168406	1.656811300792	2.248047170039
C	-0.458053895034	2.954513634919	3.151028162070
C	1.918741444403	3.585053248023	2.481479652414
H	2.713149428737	3.406090320439	1.741273994313

H	1.691327516100	4.664241223112	2.490892829179
H	2.315482194411	3.327418485444	3.474196324086
H	-0.890712311136	3.965405094792	3.065045418312
H	-0.112580722306	2.813395435925	4.185909520441
H	-1.225052317633	2.194270737665	2.950581147097
C	-0.696472238348	-2.733406556799	2.187628835839
H	-0.926303168406	-1.656811300792	2.248047170039
C	0.458053895034	-2.954513634919	3.151028162070
C	-1.918741444403	-3.585053248023	2.481479652414
H	-2.713149428737	-3.406090320439	1.741273994313
H	-1.691327516100	-4.664241223112	2.490892829179
H	-2.315482194411	-3.327418485444	3.474196324086
H	0.890712311136	-3.965405094792	3.065045418312
H	0.112580722306	-2.813395435925	4.185909520441
H	1.225052317633	-2.194270737665	2.950581147097
C	0.955362199291	-1.786166750549	-2.329744663192
H	0.942364894499	-0.738334718650	-1.982767009541
C	-0.035292029281	-1.942851930503	-3.473223340652
C	2.379794692992	-2.149434739604	-2.715896258626
H	3.058130385710	-2.014798818013	-1.861070447937
H	2.456279723768	-3.194046484605	-3.057518921434
H	2.725361354598	-1.505299473207	-3.537930714692
H	-0.085948362617	-2.986705718273	-3.821619556633
H	0.262783716329	-1.317544638806	-4.327972815837
H	-1.043967208096	-1.641159867838	-3.153640647040
F	0.000000000000	0.000000000000	2.232092005940

107

I5_opt, 1-A (C1): E(RPBE0-D3BJ/def2SVP) = -4179.18912062

G_corr(298K) = 0.767053

E(COSMO-PBE0-D3BJ/def2TZVP) = -4182.29095797

Ni	0.057508622704	0.119618574190	-0.329947834738
N	-0.914802974270	-2.507507243452	-1.107382894714
N	1.137256897921	-2.763712148425	-0.576957922723

C	0.130751685251	-1.832352731679	-0.531089224968
C	-0.565778426744	-3.786268305144	-1.504261938873
H	-1.280166312156	-4.454478857168	-1.975501006965
C	0.731067631966	-3.949260309267	-1.168919770867
H	1.406424353820	-4.792109822919	-1.282254153087
C	0.820572964818	0.437557790272	-2.028734948235
C	2.081273463021	1.150684558913	-1.943153241062
C	2.167897955005	2.506309395094	-2.077091639802
C	1.005773471619	3.315212908238	-2.181574192264
F	3.214866479484	0.449969661113	-1.866453832184
F	3.346412784796	3.124666510417	-2.035119689602
F	1.147651817039	4.636394744648	-2.278925608496
C	-0.342586053289	1.310676551973	-1.956783888145
C	-0.220560925579	2.719875234924	-2.158994585700
F	-1.507473496739	0.809312166840	-2.465357752626
F	-1.320684830540	3.450432361820	-2.320038965868
N	0.462201459463	1.982230792943	1.869941025014
N	-1.643883447855	1.642180161362	1.845789692312
C	-0.488736877587	1.206663236790	1.254808481408
C	-0.077554133294	2.865202749125	2.784142985520
H	0.537335736453	3.553118034425	3.356352002706
C	-1.411584276196	2.654914270478	2.767136645895
H	-2.223312030848	3.119954509819	3.318737492549
F	0.842534707439	-0.544510996743	-3.020803641872
C	-2.276666664670	-2.086071623421	-1.199371891120
C	-3.058804618378	-2.152466824829	-0.035557531227
C	-2.836162382142	-1.807529750073	-2.452030677400
C	-4.424203888860	-1.901831993982	-0.145151152157
C	-4.207222802629	-1.539319042671	-2.504978484241
C	-5.017458446867	-1.585973821927	-1.371344857984
H	-5.041466143921	-1.970829632054	0.752647713909
H	-4.655602266916	-1.307540704086	-3.475395504003
C	-6.487775078080	-1.301737532471	-1.462512334914
H	-6.858709933167	-1.417125899569	-2.490710479201

H	-7.068686285455	-1.971823216230	-0.811102960170
H	-6.708782522102	-0.269857516653	-1.144391974084
C	-2.022511117718	-1.849627883891	-3.709687486903
H	-2.491862889254	-1.237009229489	-4.491333140832
H	-1.004078759873	-1.481153300855	-3.546744683522
H	-1.951664143929	-2.880478291978	-4.096333558080
C	-2.435545933441	-2.535955377892	1.271041324896
H	-1.931221595048	-3.513101613964	1.198731793548
H	-1.669617038820	-1.799781149821	1.557625125364
H	-3.189262860541	-2.588517832642	2.066496170065
C	2.430652005408	-2.700600362798	0.026609236308
C	2.541462927592	-3.078877620202	1.375420147747
C	3.561616966000	-2.474401437079	-0.768341413235
C	3.812320097360	-3.114660364445	1.948027024291
C	4.813006565660	-2.502524929393	-0.142727634250
C	4.960405393709	-2.807050629181	1.209937980143
H	3.909807393935	-3.404250341108	2.998761668024
H	5.702651750709	-2.304110800000	-0.747252406940
C	6.310467805768	-2.799472515070	1.865084084421
H	6.424978356849	-3.641279401583	2.564189400292
H	7.120056966297	-2.853917647427	1.123942837652
H	6.459815654215	-1.874294621833	2.447536620440
C	3.462902366739	-2.342062492788	-2.257492762097
H	4.297945125855	-1.751796318287	-2.655706483216
H	3.512653075637	-3.343562710029	-2.718578378067
H	2.526496440364	-1.873295706511	-2.576281383260
C	1.334866447902	-3.526456954309	2.146242422572
H	0.477642135576	-2.858263872385	1.992084494331
H	1.017989410570	-4.529085820034	1.813925744374
H	1.549058781460	-3.581455282436	3.222022338416
C	1.863201956747	1.842735255779	1.656832604977
C	2.591239013708	2.873217591118	1.051317190696
C	2.479851209049	0.673752030718	2.124247094316
C	3.949210194503	2.649574917304	0.808566002584

C	3.831505259157	0.490408693270	1.846134005438
C	4.573686802055	1.451567790990	1.154890503573
H	4.524457274779	3.426984654927	0.300634608692
H	4.312504858569	-0.433267456049	2.172045228946
C	5.994412286013	1.182119301873	0.759555605520
H	6.576905305079	2.109937127037	0.666920941901
H	6.500704083682	0.522314803908	1.479115256543
H	6.015249460371	0.678108870454	-0.221266490986
C	1.697754159353	-0.317467275480	2.927894901938
H	2.358171270022	-1.074368992296	3.366301648209
H	1.145199961769	0.182372670127	3.739180648153
H	0.955034912524	-0.818173565883	2.290541628823
C	1.960925308499	4.188432731720	0.701733860064
H	0.897924051420	4.081532884694	0.448714122010
H	2.027371535580	4.890407743460	1.550114409992
H	2.469620327271	4.655050112532	-0.150558282134
C	-2.969520221625	1.111809405557	1.762498785838
C	-3.858417311916	1.579902551738	0.787716852512
C	-3.393936948260	0.281613838448	2.813895036453
C	-5.202774106655	1.213389349788	0.903855316451
C	-4.742501650959	-0.067841815092	2.876249295056
C	-5.667189534069	0.402923362533	1.938973168057
H	-5.910116460230	1.595898536681	0.162713786415
H	-5.083505045373	-0.710361432608	3.693719985619
C	-7.113224274859	0.012963253418	2.030785259868
H	-7.468712048835	0.023986526156	3.071834410719
H	-7.751446257062	0.686775346405	1.442046875910
H	-7.271847642455	-1.008224453223	1.644962842886
C	-2.429787600139	-0.179371570336	3.867522583732
H	-2.831345879156	-1.042842213478	4.415201380349
H	-1.460933549443	-0.460577636063	3.430925164725
H	-2.226935969745	0.617691223586	4.601357596170
C	-3.396712136789	2.443979282352	-0.339503726335
H	-2.989189997525	1.823075222985	-1.150197603352

H	-4.225684491355	3.034359998737	-0.752516464195
H	-2.594911392375	3.129508691615	-0.035043607373

107

I6_opt, 1-A (C1): E(RPBE0-D3BJ/def2SVP) = -4179.24450471

G_corr(298K) = 0.766549

E(COSMO-PBE0-D3BJ/def2TZVP) = -4182.35125623

Ni	-0.151065295430	-0.086445360175	-0.517775779668
N	2.375889352415	1.126081737425	-1.710885662546
N	0.808878331047	2.542853810329	-1.374526250078
C	1.227603173601	1.302440006578	-0.997902305059
C	2.618564183578	2.199883670964	-2.553188996820
H	3.476334350000	2.218437214286	-3.218424677277
C	1.632248372741	3.096258847277	-2.336513501055
H	1.434449438050	4.072723580793	-2.768309032850
C	-1.658592998588	-1.283277592826	-0.750663760928
C	-2.843829207183	-0.688820051479	-1.175051973131
C	-3.973142625484	-1.408031810330	-1.542202055308
C	-3.932806937453	-2.799642077011	-1.524476226290
F	-2.964439743532	0.645109996249	-1.192294625571
F	-5.103968844810	-0.790936836278	-1.879704056274
F	-5.018041200942	-3.503594298412	-1.829977488013
C	-1.647923875369	-2.666036207566	-0.796376510112
C	-2.758603673566	-3.436131065185	-1.143289781557
F	-0.548613487686	-3.361228099227	-0.443977088950
F	-2.709002038991	-4.765670052834	-1.099262326032
N	-1.146562632059	-0.450786964437	2.240441206593
N	0.994000780004	-0.380231044007	2.246289241802
C	-0.078096606035	-0.310620821655	1.394103323354
C	-0.753995684509	-0.590298564676	3.555981065788
H	-1.478795200417	-0.707551418867	4.355677009785
C	0.593655537760	-0.559282160893	3.561649151753
H	1.314824023003	-0.661475235687	4.365999269300
F	-0.339365962673	0.094257317203	-2.356043485270

C	2.403677476233	-0.443564138629	1.992802918303
C	3.236644977187	0.504145307144	2.604706834603
C	2.931249386543	-1.598585715491	1.386816374443
C	4.616893534684	0.276043665652	2.591859766249
C	4.309827181301	-1.786981008358	1.420944335193
C	5.171572690938	-0.869709307615	2.027856603469
H	5.271768984968	1.013665459461	3.065740427006
H	4.720936316902	-2.688984096127	0.961931166525
C	-2.539725952470	-0.575257339364	1.930088137189
C	-3.300189151324	0.571921829056	1.698401334598
C	-3.111005234488	-1.858256198966	1.973671870256
C	-4.661172233580	0.407931830749	1.426804196567
C	-4.470942074952	-1.968467996040	1.696228786167
C	-5.259578936088	-0.851074653585	1.399703024826
H	-5.261621996851	1.294205998013	1.205372384224
H	-4.925499097277	-2.963203408883	1.684279362437
C	3.383646081085	0.111442822001	-1.603939632971
C	3.233729844244	-1.154315108611	-2.191024222980
C	4.607405127990	0.521466630289	-1.039974613747
C	4.345796024776	-2.005812619853	-2.178022637056
C	5.691588982714	-0.351526015282	-1.096379076771
C	5.581014378338	-1.625159419530	-1.657310828265
H	4.238239582186	-2.995558719055	-2.632283680060
H	6.647773247932	-0.029553845695	-0.675315715016
C	-0.318346674997	3.270571156985	-0.878132756855
C	-0.161073389556	3.969622173519	0.328509167282
C	-1.474058371947	3.394116261502	-1.663108356050
C	-1.210899748765	4.771871734312	0.771997388529
C	-2.503808561904	4.200543810619	-1.166157537698
C	-2.396288036197	4.892330497768	0.040463881757
H	-1.099938932252	5.315638794354	1.714581649616
H	-3.420584505104	4.289531869632	-1.755576650254
C	1.971367791862	-1.615492803984	-2.849985223559
H	2.203176960074	-2.094243461716	-3.813088550131

H	1.468474228025	-2.374976918841	-2.228810042025
H	1.236931909755	-0.812955317987	-2.994502240517
C	6.745699847496	-2.571787099455	-1.665879788156
H	6.790460412321	-3.149011252246	-2.601189007244
H	7.700350663467	-2.040339340966	-1.544217259274
H	6.667284759294	-3.302237091470	-0.842318779900
C	4.762729256004	1.871109775003	-0.400158439975
H	5.627301975263	1.876019886578	0.276722881170
H	4.915116062578	2.667618237626	-1.145878209043
H	3.869888131993	2.139427575332	0.181844106514
C	2.032790782513	-2.622997974500	0.778716900562
H	1.386719567302	-2.179240057298	0.011401210563
H	2.611034885714	-3.434321138945	0.318808422917
H	1.355605532238	-3.060200913830	1.529754670566
C	6.647640209807	-1.128388736901	2.100591469369
H	7.226121485743	-0.195545256457	2.026464208462
H	6.915052668056	-1.600911013391	3.060461085149
H	6.978700142146	-1.802985966538	1.299004881854
C	2.710188122547	1.707739665694	3.333473527460
H	2.852473580401	1.598356884452	4.421017683840
H	3.257084185141	2.612638327964	3.028818183622
H	1.642711022294	1.877387192141	3.155598252225
C	-6.695716834490	-1.025510352806	1.005855799042
H	-7.248914167321	-0.077271546112	1.054687135838
H	-6.753273883410	-1.392183592566	-0.031677486396
H	-7.206073827403	-1.760395891711	1.646179206303
C	-2.292033612220	-3.074293446014	2.291018909743
H	-2.782274100266	-3.980517147198	1.910311188282
H	-1.292440537151	-3.022110199198	1.839873406934
H	-2.158023450232	-3.196845949350	3.378697909549
C	-2.659287046712	1.920556672900	1.680081434190
H	-1.987289212330	2.071090528669	2.538393337256
H	-2.055469498024	2.039337120207	0.768989986361
H	-3.414531562152	2.716062011477	1.685473731466

C	-3.538163352902	5.714524933388	0.561525009256
H	-4.183295403816	5.111484343334	1.223324796718
H	-3.183780910049	6.573982447035	1.149124224636
H	-4.171266626959	6.090802430892	-0.254565739620
C	-1.593736939126	2.719582265699	-2.992754520078
H	-1.044727498309	3.286043937728	-3.764202001729
H	-1.172993016393	1.702254515531	-2.948142222972
H	-2.645581402561	2.664467225746	-3.302551364020
C	1.118947510951	3.861561929653	1.096908355162
H	1.043404767929	4.362869371107	2.071115363929
H	1.383765884812	2.808954791406	1.259793857928
H	1.956882730674	4.315152904904	0.541834444493

107

I7_opt, 1-A (C1): E(UPBE0-D3BJ/def2SVP) = -4179.16437659

G_corr(298K) = 0.757385

E(COSMO-PBE0-D3BJ/def2TZVP) = -4182.28003714

C	-1.168057573519	3.558432655458	-2.307142623279
C	-2.169209160639	2.749693540942	-2.728598134720
C	-1.190100785127	1.731451672577	-0.924118801976
N	-0.584481103717	2.928975517096	-1.218537413740
N	-2.172028309699	1.653172471977	-1.882249276916
Ni	-0.853022079087	0.401526266079	0.304129122478
C	-0.750125700666	-0.888827177944	1.619815365772
N	-1.850151507996	-1.435147202025	2.229420734231
N	0.289505185449	-1.440060640832	2.336611663964
C	-1.514524441857	-2.263300077660	3.287426952029
C	-0.163250352787	-2.266511080637	3.356287192341
H	-2.265285051755	-2.756268547206	3.898476904843
H	0.515345472568	-2.762449764233	4.043620713893
H	-0.817866715865	4.517207582620	-2.678477852841
H	-2.870747316851	2.849731508277	-3.551792622904
C	2.832577175795	-0.711144149911	-1.262102495805
C	4.147414586733	-0.257872530926	-1.319036382781

C	2.565923835013	-2.071130155918	-1.397174372950
C	5.190237768207	-1.154331388071	-1.534231059407
C	3.608482536892	-2.970471659203	-1.603127376293
C	4.923366669634	-2.515168598282	-1.657417236353
F	1.850721543411	0.135251671368	-1.041733889574
F	1.327667961787	-2.518421979376	-1.313293906205
F	3.354015827282	-4.264574697790	-1.700110737650
F	5.915145368909	-3.371567107243	-1.825557231511
F	6.437490630561	-0.718943745234	-1.574622486294
F	4.417917086191	1.025693764099	-1.153412861810
C	0.553215749304	3.437977420954	-0.531813956743
C	1.810793760235	3.362336094974	-1.147031770379
C	0.381738768411	4.035186704762	0.725421063717
C	2.914076477487	3.861509680061	-0.450804363941
C	1.510793351002	4.535277794126	1.374577170408
C	2.787016887492	4.443871291872	0.811757794057
H	3.903571020670	3.785640317016	-0.909844593779
H	1.388717816311	5.000668028602	2.357241447985
C	3.993858375448	4.927155609143	1.562903072078
H	4.382325774492	4.138933966300	2.230533292972
H	4.809855783576	5.207816096708	0.881550503644
H	3.756974178090	5.796897951007	2.193095196125
C	-0.976272528146	4.102940358583	1.349801818975
H	-0.949662636560	4.639500982897	2.307700293746
H	-1.700164783242	4.600953850945	0.685853851847
H	-1.358296778926	3.083291947760	1.518251533666
C	1.965360229119	2.782515681620	-2.521727804530
H	1.315978407721	1.907550028840	-2.658920744578
H	1.689752348112	3.516612334229	-3.296549170424
H	3.005440707575	2.481262706057	-2.701704128323
C	-3.088110619400	0.569732847660	-2.003427141670
C	-4.447072379826	0.809504949219	-1.760153051124
C	-2.624230005086	-0.691682067966	-2.413953653755
C	-5.353638604275	-0.224870633370	-2.002059412662

C	-3.567179619459	-1.698954587073	-2.617200528536
C	-4.936448627090	-1.481327680097	-2.439487831746
H	-6.416148946559	-0.047169911551	-1.814667608242
H	-3.216149644961	-2.686948451349	-2.929804234647
C	-5.924743934002	-2.581222936213	-2.697801772946
H	-6.919535775605	-2.330692468124	-2.302200847950
H	-6.035928525200	-2.773481801654	-3.777761410303
H	-5.602358279614	-3.527451430433	-2.235610714809
C	-1.161048139320	-0.956596962915	-2.555384023499
H	-0.966281881275	-1.973928381934	-2.919700412367
H	-0.675082617796	-0.232436893001	-3.228072464814
H	-0.687148428628	-0.822497915829	-1.558706351086
C	-4.907259720319	2.124996979783	-1.204979947636
H	-4.248450574081	2.443622142249	-0.382710254401
H	-4.896009906392	2.929354650510	-1.957856935473
H	-5.931787589028	2.043741973145	-0.817147240061
C	-3.196281135450	-1.147966829202	1.862262982125
C	-3.782974437567	0.051006556108	2.295417692178
C	-3.915972167006	-2.105177084923	1.138403047342
C	-5.131750698166	0.258920505276	2.009547213702
C	-5.269621721342	-1.863470730457	0.896849521166
C	-5.896678916865	-0.694970705993	1.330571086904
H	-5.598954084252	1.194763756171	2.330797316856
H	-5.844364391049	-2.603264425802	0.333396867994
C	-7.355776646681	-0.457656289189	1.069279199946
H	-7.946948023895	-0.545530380358	1.995793912845
H	-7.533500044130	0.553433606134	0.670714963270
H	-7.759377470357	-1.183226906104	0.348670398251
C	-3.224966096958	-3.322591253285	0.603116374338
H	-3.893699053499	-3.899823580595	-0.049472209000
H	-2.340630391015	-3.021533211464	0.019266418947
H	-2.869657618142	-3.987724927321	1.406343850702
C	-2.940178277460	1.083428643675	2.972444710653
H	-2.486880396554	0.702461734947	3.901178425733

H	-2.105422922078	1.326174671295	2.284945233439
H	-3.513680095510	1.991525247414	3.201549599305
C	1.671144623301	-1.189408303714	2.111909006737
C	2.170109573506	0.118848971799	2.266597477805
C	2.518826856854	-2.264456336624	1.804100638061
C	3.536783959809	0.323968143135	2.079104929919
C	3.890123242980	-2.013146307894	1.680467218421
C	4.417794478123	-0.728406489466	1.806128398749
H	3.926443448062	1.342011308716	2.168970488499
H	4.559229146901	-2.848721157402	1.453292825075
C	5.888568999518	-0.469013602362	1.651998308053
H	6.337764656151	-0.151700241613	2.607013070833
H	6.425200750793	-1.365921471790	1.311877447577
H	6.078443276383	0.333978223203	0.923635663559
C	1.985237223134	-3.651910885541	1.586822893973
H	2.636220415729	-4.214649502736	0.903010934965
H	1.927336826721	-4.224432324486	2.526749960195
H	0.974730245485	-3.626240272910	1.157003859649
C	1.258217229119	1.246828617885	2.614556898221
H	0.478327929865	1.343374944789	1.824344520100
H	0.712959483297	1.041916981318	3.549836284025
H	1.806344448577	2.191420511917	2.709762941063

107

I8_opt, 1-A (C1): E(RPBE0-D3BJ/def2SVP) = -4179.19806241

G_corr(298K) = 0.764752

E(COSMO-PBE0-D3BJ/def2TZVP) = -4182.31879974

Ni	0.601938228724	-0.326588550161	0.142069116671
N	0.452961214506	1.328067424827	2.629648051463
N	0.272133551305	-0.785808263090	2.930055677625
C	0.397840646235	0.155470707540	1.970389143025
C	0.315871380047	1.127920085440	3.988858349839
H	0.298887380390	1.955944873467	4.689902701998
C	0.195843923343	-0.208646719527	4.179015816357

H	0.058882020664	-0.790758999458	5.083353120031
C	-1.027618178131	0.538037916141	-0.106196227756
C	-2.183656396756	-0.083350892901	0.348889126305
C	-3.428670358492	0.525755138072	0.252073746527
C	-3.522167932829	1.804824638841	-0.277566000522
F	-2.135584716507	-1.348037217451	0.755414643981
F	-4.524712665256	-0.100025449725	0.656784335469
F	-4.705690017504	2.403428245856	-0.376725036218
C	-1.151570327360	1.808968078721	-0.640995957390
C	-2.379033144880	2.451314539234	-0.742205923698
F	-0.078397608581	2.450070958767	-1.138498185551
F	-2.471568206826	3.667193100371	-1.283443962937
N	-0.058340682271	-0.993545806955	-2.729929380134
N	2.034594818431	-0.711777501496	-2.407910086920
C	0.841816062573	-0.720577065682	-1.748829591558
C	0.555418420011	-1.150975955514	-3.956459462576
H	-0.014514000198	-1.378866267480	-4.852110024490
C	1.882745703472	-0.971122357339	-3.756247410674
H	2.728976404225	-0.999325219913	-4.436378470592
F	-2.254493486233	0.314241175741	2.716453792445
C	0.597216327754	2.601453203089	2.000456561038
C	1.850564900273	2.937208521888	1.472038188626
C	-0.511280089019	3.460051977234	1.920574782914
C	1.972316647893	4.147204172177	0.787676980234
C	-0.336076285864	4.650127473515	1.207520301681
C	0.880544645251	5.001884059086	0.620636996661
H	2.943517681648	4.423398013628	0.366558439710
H	-1.196583114917	5.315679800854	1.095965299896
C	0.996327313962	6.253590908207	-0.198314805228
H	0.455450570958	7.092331997571	0.264516499370
H	2.044600334390	6.554273479369	-0.338572735945
H	0.556048964418	6.095811845858	-1.197034101337
C	-1.816366994846	3.128112915396	2.571433889200
H	-2.636915783445	3.653398410440	2.061065569218

H	-2.031340362346	2.026905288387	2.607150438936
H	-1.809041607319	3.488451382742	3.615914835996
C	3.025800377282	2.018488155686	1.632839579884
H	3.100731961303	1.634535520516	2.661659554297
H	2.947931397391	1.142409964830	0.969973394260
H	3.963836749131	2.533165937277	1.384414028933
C	0.170326477833	-2.162258591354	2.598106959631
C	1.182077573775	-2.701437064629	1.789470301894
C	-0.951832133471	-2.909815532519	2.991412924293
C	1.075303850798	-4.032238786197	1.382159444773
C	-1.006414167436	-4.238346799429	2.554032747503
C	-0.018580752905	-4.816989557127	1.754193638749
H	1.866332233639	-4.465650726974	0.763395795380
H	-1.877580711924	-4.835228740855	2.839227949547
C	-0.152354889730	-6.230955792996	1.268721937621
H	-0.765940006330	-6.273001919600	0.352844752326
H	0.825991877785	-6.671803296289	1.028445183811
H	-0.642782979573	-6.870277032604	2.016978835417
C	-2.065659782457	-2.297435733144	3.776623785087
H	-2.965163334248	-2.924585587267	3.701481070587
H	-1.807461175612	-2.230429465408	4.847382022724
H	-2.281104830462	-1.254749093667	3.402431032859
C	2.364072151495	-1.874729776056	1.372682395616
H	2.225932578416	-1.262574765722	0.440722021411
H	2.694947451048	-1.182132396046	2.156674920861
H	3.210123646363	-2.515832088328	1.091320686345
C	-1.479606359938	-1.110457839034	-2.597975973135
C	-2.279002209392	-0.077917335654	-3.113655690124
C	-2.018563909023	-2.261104823483	-2.014341897791
C	-3.658865192164	-0.205974466212	-2.977702363428
C	-3.409084963508	-2.339016896346	-1.906912888529
C	-4.243088944538	-1.318272719419	-2.362431571053
H	-4.299019581150	0.605691433131	-3.334524674823
H	-3.848611956684	-3.214747169847	-1.422503586072

C	-5.724752593179	-1.368224575173	-2.141748964888
H	-6.278184726260	-0.968163215037	-3.004047599259
H	-6.073024826960	-2.392182402730	-1.947285298236
H	-5.983267677641	-0.754857741037	-1.263660957286
C	-1.144005355370	-3.343832137712	-1.460890510088
H	-1.704828103668	-4.283035277903	-1.361310270942
H	-0.263654372603	-3.531093933173	-2.094003412912
H	-0.781818270017	-3.070409436952	-0.457791711648
C	-1.680279073788	1.131745040720	-3.770473328201
H	-0.759919351793	1.458260696131	-3.267744429983
H	-1.430468843693	0.937325712738	-4.827115873013
H	-2.386889044479	1.972114591788	-3.745634103358
C	3.314787355128	-0.516621534899	-1.807061004363
C	3.801808514389	0.786116003392	-1.635883710878
C	4.066608742932	-1.651160461658	-1.467458180215
C	5.069263764015	0.929448253281	-1.066337110903
C	5.322617418329	-1.452724356499	-0.893314430891
C	5.837300148732	-0.170449172266	-0.677023120688
H	5.466334988535	1.938102619777	-0.922848167637
H	5.918073541325	-2.325477082699	-0.610669482944
C	7.172175963619	0.021551416638	-0.019709264903
H	7.833357115078	-0.840841776585	-0.184988747667
H	7.679451104522	0.922692632200	-0.392953292825
H	7.054059398662	0.141457793623	1.070184293580
C	3.528054382476	-3.029090677921	-1.716379771393
H	3.508925084235	-3.262786863976	-2.792966486246
H	4.146399186683	-3.789723340329	-1.221089211285
H	2.493163897360	-3.130989930969	-1.355078165854
C	2.987017892430	1.974993711345	-2.040222547964
H	2.593752451683	1.865013391109	-3.062276327830
H	2.114629377459	2.110239802140	-1.383435859239
H	3.586941560280	2.893173923022	-1.997214173528

107

I9_opt, 1-A (C1): E(RPBE0-D3BJ/def2SVP) = -4179.19801767

G_corr(298K) = 0.762999

E(COSMO-PBE0-D3BJ/def2TZVP) = -4182.29640252

Ni	0.290332192968	-0.162697896845	0.284051389984
N	0.586955334903	1.472543505303	2.585202737035
N	1.318169697899	-0.626511570602	2.658893043462
C	0.225623471736	0.153143551254	2.150847687996
C	1.626252664277	1.402356991554	3.506936766368
H	1.941272814780	2.275403219326	4.072788029854
C	2.073614300985	0.132411573513	3.559506877758
H	2.836713009050	-0.320686585111	4.186698621336
C	-1.519619423804	0.252990340845	0.157493497208
C	-2.516801733271	-0.586178460762	0.645884108376
C	-3.867883201919	-0.265414076086	0.589408198552
C	-4.265082881384	0.934596166302	0.003407434154
F	-2.209557481714	-1.776323642963	1.168108774412
F	-4.792499824814	-1.107190492743	1.045524220726
F	-5.554110603858	1.239970709758	-0.088581452729
C	-1.957165164073	1.429461530387	-0.430357555902
C	-3.298640054762	1.794408892071	-0.506478172157
F	-1.081659894290	2.276747983405	-0.993258077044
F	-3.662583905347	2.936328502336	-1.084515512293
N	-0.261351411978	-1.107715591917	-2.505048435604
N	1.660050282009	-0.185863383721	-2.468614918529
C	0.620824986167	-0.515683651687	-1.648237346320
C	0.213585616848	-1.145128319573	-3.800690890155
H	-0.360139996962	-1.590805352821	-4.607514581376
C	1.430808652201	-0.557473455505	-3.780817902890
H	2.157008045019	-0.367127136909	-4.565631393144
F	-0.954461323260	-0.220462931661	2.847000305307
C	0.115038793848	2.675750826751	2.004962599732
C	1.008531765938	3.452571920989	1.243340637202
C	-1.216552844017	3.094967371200	2.188475818208
C	0.538733701123	4.610675895464	0.622069700759

C	-1.645256260505	4.246482170476	1.524289416365
C	-0.794825577584	5.008029650674	0.722675611946
H	1.231173407091	5.201945703099	0.015202724827
H	-2.688116249935	4.556665501514	1.640465577899
C	-1.319615166102	6.190767192821	-0.036129327250
H	-1.955123110532	6.829512321300	0.596041486929
H	-0.506290140019	6.809137059050	-0.441872849045
H	-1.940490265650	5.851524248735	-0.881934689641
C	-2.177945197303	2.349135976134	3.061961934739
H	-2.949006835010	3.030127202397	3.448855376940
H	-2.679589534635	1.545023952791	2.503971784351
H	-1.663764264236	1.859693690499	3.898385035842
C	2.430839118907	3.018810411393	1.056740498249
H	3.010892611854	3.063222096990	1.990297050731
H	2.481542953714	1.973780227568	0.712035382074
H	2.938769981664	3.647337215180	0.314913190046
C	1.624275451315	-1.960144609264	2.282852694569
C	2.816436669443	-2.188510218108	1.563240152395
C	0.815238196427	-3.043086728408	2.681897632671
C	3.133101063473	-3.488455626981	1.169070777945
C	1.166307908758	-4.324351094195	2.242515472284
C	2.302903789197	-4.570456757922	1.473122196782
H	4.056309329596	-3.660279725284	0.608132380277
H	0.531296954959	-5.164931806871	2.538347120277
C	2.619683117298	-5.951760723852	0.979438702180
H	3.702110331956	-6.099226183983	0.852447757723
H	2.246170972875	-6.722832442591	1.668868750806
H	2.147194004273	-6.134851325248	-0.000706377562
C	-0.366833420549	-2.876517349231	3.586317754177
H	-0.540009122191	-3.802244417766	4.153136559857
H	-0.218063284042	-2.041810447443	4.282990896800
H	-1.274412147941	-2.636643954809	3.017360868801
C	3.733159595096	-1.048955465325	1.237270025347
H	3.203086209290	-0.244095440003	0.703631423711

H	4.149386409956	-0.585500991663	2.143460111730
H	4.569803917169	-1.378367709223	0.610899945092
C	-1.548020145441	-1.650242549065	-2.199400142812
C	-2.681488999599	-0.985518640772	-2.695390603910
C	-1.632556151397	-2.854995617773	-1.493755464806
C	-3.927325570597	-1.552149197654	-2.435135805558
C	-2.906149065863	-3.381767524772	-1.265960255003
C	-4.062458493244	-2.744317094607	-1.715672076557
H	-4.824137417096	-1.033504285835	-2.786039764048
H	-2.993504110704	-4.310991227710	-0.696798860377
C	-5.423093317576	-3.286389646543	-1.393773195488
H	-6.114308344966	-3.176629007042	-2.242457175091
H	-5.381215514432	-4.348627656090	-1.115173480702
H	-5.850753100768	-2.732781837595	-0.542709741427
C	-0.409210311715	-3.527239262470	-0.950419518758
H	-0.627983281293	-4.561405270559	-0.653007167964
H	0.414367311304	-3.539483326036	-1.680132487202
H	-0.041073879060	-2.999574268184	-0.055615495471
C	-2.568831391954	0.300127421270	-3.463024443033
H	-1.780762141072	0.949855202406	-3.058039995408
H	-2.333906106488	0.121960925805	-4.525542518744
H	-3.515228020620	0.856235039180	-3.423496408599
C	2.904956831997	0.417514337378	-2.114095491144
C	3.006711606435	1.814308785410	-2.084070364837
C	4.020246635587	-0.418418952033	-1.964057947805
C	4.267587317571	2.363955714265	-1.841580691351
C	5.256882954541	0.180140235809	-1.721036980634
C	5.398922285715	1.568382723694	-1.645256069074
H	4.367808548161	3.452639351879	-1.815239167379
H	6.135518622091	-0.457979888128	-1.590106841230
C	6.729978077837	2.190040333752	-1.340146575401
H	7.561027043612	1.551535522088	-1.672156621585
H	6.836122294473	3.173197318073	-1.820822959334
H	6.846762225445	2.343174821224	-0.254275370126

C	3.877434587852	-1.907214186303	-2.071480877274
H	4.809803049040	-2.413454157016	-1.787805325897
H	3.069945104655	-2.280696387097	-1.423528514184
H	3.629409972283	-2.213823381149	-3.100261593126
C	1.806533278634	2.677127046392	-2.318113700946
H	1.292162132917	2.401586770017	-3.251913442938
H	1.066888724973	2.568950960637	-1.512158142646
H	2.089971062796	3.735858002004	-2.380828122556

27

iprim_opt, 1-A1 (C2V): E(RPBE0-D3BJ/def2SVP) = -461.204041072

G_corr(298K) = 0.202775

E(COSMO-PBE0-D3BJ/def2TZVP) = -461.704022802

N	0.000000000000	1.060062497000	0.073960463690
N	-0.000000000000	-1.060062497000	0.073960463690
C	0.000000000000	0.000000000000	0.926634042690
C	0.000000000000	0.679041998000	-1.255528157310
H	-0.000000000000	1.380298497000	-2.086476743310
C	-0.000000000000	-0.679041998000	-1.255528157310
H	-0.000000000000	-1.380298497000	-2.086476743310
C	-0.000000000000	2.439424497000	0.543388513690
H	-0.000000000000	2.336730489000	1.638522558690
C	-1.267479055000	3.165588251000	0.115148759690
C	1.267479055000	3.165588251000	0.115148759690
H	2.159520583000	2.614844998000	0.446988763690
H	1.319232058000	3.276243010000	-0.979928027310
H	1.298829314000	4.174129750000	0.553630041690
H	-1.319232058000	3.276243010000	-0.979928027310
H	-1.298829314000	4.174129750000	0.553630041690
H	-2.159520583000	2.614844998000	0.446988763690
C	-0.000000000000	-2.439424497000	0.543388513690
H	-0.000000000000	-2.336730489000	1.638522558690
C	1.267479055000	-3.165588251000	0.115148759690
C	-1.267479055000	-3.165588251000	0.115148759690

H	-2.159520583000	-2.614844998000	0.446988763690
H	-1.319232058000	-3.276243010000	-0.979928027310
H	-1.298829314000	-4.174129750000	0.553630041690
H	1.319232058000	-3.276243010000	-0.979928027310
H	1.298829314000	-4.174129750000	0.553630041690
H	2.159520583000	-2.614844998000	0.446988763690

47

mesim_opt, 1-A (C2): E(RPBE0-D3BJ/def2SVP) = -922.480551956

G_corr(298K) = 0.341913

E(COSMO-PBE0-D3BJ/def2TZVP) = -923.457565336

N	0.753691980429	-0.741170763190	-0.589901584801
N	-0.753691980429	0.741170763190	-0.589901584801
C	0.000000000000	0.000000000000	0.270298061986
C	0.483710293373	-0.474969130943	-1.924944381616
H	0.998374465088	-0.976064974378	-2.740741975750
C	-0.483710293373	0.474969130943	-1.924944381616
H	-0.998374465088	0.976064974378	-2.740741975750
C	-1.722449602359	1.694093784515	-0.158400809571
C	-1.380567697597	3.051943030865	-0.140915830846
C	-2.991697768345	1.245021124505	0.230721869713
C	-2.353205241310	3.969346328118	0.264287093735
C	-3.930258914735	2.197861493446	0.629880876967
C	-3.632038061160	3.563483017434	0.652143129132
H	-2.098986423346	5.033186875529	0.282946902820
H	-4.925776736460	1.860981303852	0.933858967101
C	1.722449602359	-1.694093784515	-0.158400809571
C	2.991697768345	-1.245021124505	0.230721869713
C	1.380567697597	-3.051943030865	-0.140915830846
C	3.930258914735	-2.197861493446	0.629880876967
C	2.353205241310	-3.969346328118	0.264287093735
C	3.632038061160	-3.563483017434	0.652143129132
H	4.925776736460	-1.860981303852	0.933858967101
H	2.098986423346	-5.033186875529	0.282946902820

C	-3.310318924468	-0.219704335797	0.233899668842
H	-2.666648306062	-0.747887613748	0.954861458867
H	-4.360898314097	-0.396295903596	0.501031388036
H	-3.119701844631	-0.675511539863	-0.750436755280
C	-4.651897319249	4.562647310357	1.116949940897
H	-4.434251951861	5.570197812256	0.734853803755
H	-5.665668934505	4.284354270526	0.792999162280
H	-4.667948694101	4.624180574879	2.217969765600
C	-0.000000000000	3.494847513148	-0.525091416162
H	0.212052532227	3.296555599908	-1.587794449071
H	0.133058145315	4.570430862235	-0.347368473824
H	0.758100758411	2.946079392692	0.055056266568
C	4.651897319249	-4.562647310357	1.116949940897
H	5.665668934505	-4.284354270526	0.792999162280
H	4.667948694101	-4.624180574879	2.217969765600
H	4.434251951861	-5.570197812256	0.734853803755
C	-0.000000000000	-3.494847513148	-0.525091416162
H	-0.133058145315	-4.570430862235	-0.347368473824
H	-0.758100758411	-2.946079392692	0.055056266568
H	-0.212052532227	-3.296555599908	-1.587794449071
C	3.310318924468	0.219704335797	0.233899668842
H	3.119701844631	0.675511539863	-0.750436755280
H	2.666648306062	0.747887613748	0.954861458867
H	4.360898314097	0.396295903596	0.501031388036

108

TS10_opt, 2-A (C1): E(UPBE0-D3BJ/def2SVP) = -4278.86878208

G_corr(298K) = 0.756329

E(COSMO-PBE0-D3BJ/def2TZVP) = -4282.09489626

C	0.036589365241	3.030817260167	-3.365463234230
C	-1.076187309878	2.305259305499	-3.640161015308
C	-0.295164521988	1.620822410350	-1.629734603815
N	0.493714771053	2.597490118729	-2.131731791478
N	-1.254553668839	1.451610177908	-2.565951066314

Ni	-0.291679677015	0.771897406690	0.116153377198
C	-0.350956288713	-0.017709418829	1.901158386072
N	-1.414935234645	-0.078724653430	2.737357166103
N	0.704036124030	-0.340438299716	2.688437833567
C	-1.033318707823	-0.432201003436	4.019805932822
C	0.312105932302	-0.592337493616	3.990740245597
H	-1.755290595718	-0.538123854682	4.824190176263
H	1.023701415841	-0.853564381609	4.768329923193
H	0.547233671165	3.808816314767	-3.925504410105
H	-1.754568681575	2.316551481078	-4.488240458193
C	1.058587365239	-2.314199633258	-1.173568277036
C	2.413739739935	-2.576230657692	-1.057327020981
C	0.139682820769	-3.330946382927	-0.975784006433
C	2.855369189205	-3.857618517453	-0.753018854279
C	0.565180903206	-4.619370173156	-0.669407238449
C	1.928619183183	-4.873633158019	-0.517944840199
F	0.593394707078	-0.847852010380	-0.783875132276
F	-1.169346607196	-3.126818283616	-1.177412828268
F	-0.306989986967	-5.609088271082	-0.516481954636
F	2.345554285828	-6.076715137076	-0.144168259528
F	4.154917966378	-4.124597945556	-0.648513540431
F	3.327389585474	-1.635873644964	-1.314332173871
C	1.649103121312	3.144481755824	-1.490816157685
C	2.883898335268	2.513975433679	-1.686449640176
C	1.511319437657	4.318657730235	-0.737898648032
C	4.010245689007	3.098836914011	-1.104518921629
C	2.669196520256	4.863999086682	-0.178600724507
C	3.923329074941	4.270848129018	-0.348841374064
H	4.982897625326	2.620155791766	-1.249525358650
H	2.583097560511	5.778086244511	0.415806019569
C	5.142898773457	4.850770152281	0.306851673467
H	5.287357303974	4.420815902880	1.313072420413
H	6.053408867828	4.637885631150	-0.271762387966
H	5.058343333294	5.940166526580	0.428874845113

C	0.161356216399	4.921955323798	-0.506304485063
H	0.238838090508	5.845145727677	0.083350320511
H	-0.348751385500	5.159285954974	-1.453295498284
H	-0.461660436277	4.188518348488	0.036470210515
C	2.973812065721	1.246500539989	-2.477013582892
H	2.396116459584	0.448498575695	-1.989872514452
H	2.564334278290	1.372927832910	-3.491740882086
H	4.013152296839	0.904442256188	-2.561730460001
C	-2.352939278510	0.549127858087	-2.419258293718
C	-3.474401978930	0.977867005731	-1.697201337138
C	-2.275273614466	-0.715636152252	-3.017946772525
C	-4.548673223862	0.091239831223	-1.597639351180
C	-3.365375294956	-1.571743695417	-2.864343486059
C	-4.509385768798	-1.185584575679	-2.160165548081
H	-5.437854680078	0.409943233172	-1.050271698664
H	-3.312775616572	-2.572303169679	-3.301856162413
C	-5.653651407660	-2.140533771412	-1.982327146735
H	-6.561334070552	-1.624251551730	-1.638955946441
H	-5.891405745809	-2.667495016611	-2.918561732773
H	-5.404633432031	-2.911672885202	-1.233956830225
C	-1.046665786191	-1.135807169114	-3.764208149418
H	-1.158573313731	-2.152112611360	-4.163566671879
H	-0.826067165874	-0.457917989193	-4.603665462054
H	-0.171256618383	-1.124099993381	-3.096425678669
C	-3.503134383367	2.322671169895	-1.036835610257
H	-2.716901614958	2.394476479769	-0.264223097261
H	-3.316586498644	3.132186858042	-1.760827833358
H	-4.475917237444	2.500009362608	-0.559789468405
C	-2.779691121147	0.176513777441	2.394876254217
C	-3.355979286139	1.396243377845	2.781178744324
C	-3.513886085969	-0.834607263865	1.764816950521
C	-4.714341825367	1.578891110512	2.519469554495
C	-4.873698216139	-0.607112204918	1.541897406391
C	-5.490393472026	0.589492709895	1.908377637858

H	-5.178968292040	2.527077202377	2.805147092354
H	-5.463170332069	-1.390978980419	1.059015477574
C	-6.942456590594	0.829679268652	1.613106234082
H	-7.445003206762	1.346508873692	2.444220089636
H	-7.061807812099	1.466814256152	0.719890123259
H	-7.477066244133	-0.111354514596	1.420434750485
C	-2.855173568285	-2.099993324806	1.311807265263
H	-3.599129582774	-2.842715748007	0.994916983556
H	-2.193719929247	-1.906204931107	0.454952355765
H	-2.233069053067	-2.546720271085	2.102691770027
C	-2.524399142034	2.474418987802	3.403213608590
H	-2.060948484211	2.147560501799	4.347322628954
H	-1.718089312598	2.733206584467	2.696156016695
H	-3.131114579978	3.365798745507	3.611578860839
C	2.056915823071	-0.402527676292	2.236870138365
C	2.735751795783	0.790441381158	1.962468315054
C	2.658885054001	-1.662308050865	2.101547424498
C	4.045710895409	0.686602613778	1.484606221076
C	3.974046607172	-1.707763510883	1.644586897912
C	4.673509371642	-0.545664911973	1.305520673113
H	4.584256133650	1.605468307974	1.241501770510
H	4.450979295313	-2.680971593587	1.501947406777
C	6.049685136968	-0.643038256645	0.717872764492
H	6.510400639537	0.347062623218	0.594019646643
H	6.715703529176	-1.256976476485	1.343532319668
H	5.999422346726	-1.124532597940	-0.272185640261
C	1.897277410661	-2.919374508585	2.400984156866
H	2.491783980905	-3.806750358681	2.148258417875
H	1.618590042988	-2.994143527619	3.463437047237
H	0.960960349945	-2.956729393732	1.821841421657
C	2.099942968653	2.127076685726	2.201800149641
H	1.167760641695	2.265401369236	1.630634298178
H	1.831596396724	2.244882594642	3.264665837204
H	2.787767985982	2.936172595440	1.926587706750

F	-0.899643600610	2.386009184795	0.864713090260

67

TS11_opt, 1-A (C1): E(RPBE0-D3BJ/def2SVP) = -3256.59384729

G_corr(298K) = 0.465236

E(COSMO-PBE0-D3BJ/def2TZVP) = -3258.75633899

Ni	-0.561127727820	0.070732793227	-0.121139684005
N	-3.500453263189	0.767421861221	-0.291899343488
N	-3.206204232670	-1.347797210639	-0.389175022067
C	-2.531146885871	-0.179623472526	-0.278349093210
C	-4.754122583003	0.206870506169	-0.409181939436
H	-5.669734893978	0.791056833590	-0.442003118511
C	-4.565176687024	-1.139709091789	-0.472314904114
H	-5.286430821838	-1.946434654300	-0.570916344124
C	0.831364580492	-0.644136281262	-1.265912767331
C	1.540660989790	-1.780306941631	-0.768422085079
C	2.915537660497	-1.804425310184	-0.643965521110
C	3.704946910378	-0.777803943407	-1.163783122217
F	0.827495881887	-2.766381916204	-0.172734198304
F	3.503355038091	-2.829768024141	-0.024436398560
F	5.038029310378	-0.839115076801	-1.121460266642
C	1.675935659418	0.312878545352	-1.910552364269
C	3.049261395681	0.286682944940	-1.789996494219
F	1.094074172919	1.409627861254	-2.437496466558
F	3.775098638425	1.292883527948	-2.282714290350
N	1.134622454619	0.081952691485	2.202435145157
N	1.248339017034	1.964067427762	1.180758474544
C	0.658306491845	0.742033681177	1.114290721141
C	1.993271722458	0.875199487995	2.935872582814
H	2.493848757310	0.531780635271	3.836327609228
C	2.066250542369	2.066652496301	2.289034552878
H	2.643300361201	2.957667412402	2.518439083824
C	-2.530849846500	-2.644851834338	-0.421247079564
H	-1.464909532509	-2.389418338641	-0.352553609872

C	-2.756360378116	-3.344510900153	-1.750239421480
C	-2.917575833952	-3.483660529198	0.785777868436
H	-2.723742974078	-2.933493684096	1.718831662321
H	-3.982437631185	-3.766413298786	0.765562101578
H	-2.324753008013	-4.409586742258	0.802881372276
H	-3.816644920452	-3.601280655338	-1.906581830747
H	-2.174592978531	-4.276983751176	-1.782028569682
H	-2.416234800739	-2.703292650406	-2.575054189340
C	-3.193783948844	2.191811937709	-0.211966376463
H	-2.098842763555	2.198305381431	-0.084535322226
C	-3.836634601243	2.822869876062	1.011949949485
C	-3.538862879943	2.899228130083	-1.511963650173
H	-3.031975573752	2.415097030334	-2.359167660684
H	-4.623781761244	2.889169875966	-1.703406904355
H	-3.215475806359	3.949756327784	-1.469322554571
H	-4.936246427354	2.811699113170	0.946483936208
H	-3.520518141560	3.871978949504	1.107299580631
H	-3.539055907113	2.286773322878	1.925121790400
C	0.664082597281	-1.247798850870	2.582070745093
H	0.185087601666	-1.625846451302	1.667969369278
C	-0.392259697271	-1.137984375833	3.671933344782
C	1.812636960741	-2.172373997146	2.942346033219
H	2.535451697448	-2.248655483336	2.119381411026
H	2.340397316748	-1.840261314328	3.850706788680
H	1.422692918720	-3.181322557368	3.139307344955
H	0.026750713873	-0.714003972285	4.598486175662
H	-0.802784636111	-2.131061636634	3.908440318686
H	-1.217562175934	-0.492681553973	3.335215213955
C	0.948631122322	3.047598586428	0.249336989413
H	0.392385065469	2.550776112062	-0.559385534132
C	2.210943676806	3.654379892099	-0.337883610143
C	0.050030751795	4.080590741036	0.916016464310
H	-0.843148517315	3.602957188745	1.345169137107
H	0.577126520808	4.605572572500	1.728494765303

H	-0.274640694851	4.834573138141	0.183432979324
H	2.820991937191	4.152416366933	0.432571245703
H	1.940675266999	4.413132321166	-1.086381809481
H	2.824000019296	2.894650160494	-0.838626111378
F	-0.337194373088	-1.013282525322	-2.083401367421

67

TS12_opt, 1-A (C1): E(RPBE0-D3BJ/def2SVP) = -3256.64002479

G_corr(298K) = 0.468773

E(COSMO-PBE0-D3BJ/def2TZVP) = -3258.80386198

Ni	0.292060355683	0.042962159939	-0.626248315500
N	1.763788691736	-2.637562324098	-0.507678270531
N	3.159816850459	-1.142998551257	0.102916049905
C	1.850635224201	-1.327512410569	-0.183410906136
C	2.992195935184	-3.261749744174	-0.441190565915
H	3.138956909030	-4.316263430857	-0.655125116108
C	3.881879840296	-2.310939873547	-0.052663792363
H	4.949123649611	-2.383066613582	0.133717605814
C	-1.555686975245	-0.155298470332	-0.233980995030
C	-2.499498009173	0.594550846318	-0.931463610565
C	-3.874812509454	0.407729366876	-0.808803583065
C	-4.353538736556	-0.592799344750	0.033503750668
F	-2.111668081105	1.559981661464	-1.768100001974
F	-4.732363404804	1.155941593991	-1.489883463156
F	-5.656084652425	-0.793877132890	0.160109143481
C	-2.083769361156	-1.148438281710	0.586714666365
C	-3.449529706745	-1.379678887183	0.740937040231
F	-1.278012802356	-1.964855164227	1.291294185129
F	-3.895052329962	-2.339601399311	1.542294878337
N	0.445950266156	2.760003675504	0.125197924617
N	0.338032786319	1.497295159881	1.867749790245
C	0.311550265941	1.469881529830	0.514380851237
C	0.553264405588	3.585400238138	1.225146258763
H	0.669166419828	4.661639951044	1.138996006791

C	0.481973144644	2.791186875143	2.324447840840
H	0.521040253863	3.040579358913	3.380681711673
C	3.736168251080	0.194145790664	0.238319225075
H	2.930208392777	0.801398754191	0.678869605613
C	4.045899880024	0.737122302037	-1.150417796035
C	4.927632097688	0.204415544461	1.179559216786
H	4.682015604955	-0.250130138956	2.151378022756
H	5.790241298561	-0.332016583104	0.754408532806
H	5.247769311757	1.241183810162	1.356387207966
H	4.808302784937	0.113733726890	-1.644626689710
H	4.440091314592	1.762630870772	-1.084473913330
H	3.124309509738	0.726671548829	-1.758599873896
C	0.547772480091	-3.189572627149	-1.106264913752
H	-0.257938235906	-2.573850738077	-0.683079870836
C	0.312913994336	-4.628582979912	-0.688160145826
C	0.585171021873	-2.977278183875	-2.613974356612
H	0.770393701273	-1.913456952799	-2.836962361305
H	1.384731155323	-3.579153765128	-3.075225468054
H	-0.373006624141	-3.274636826292	-3.065713980780
H	1.073461890117	-5.308777741287	-1.103548671120
H	-0.663844575117	-4.965233304591	-1.063695196397
H	0.310031397610	-4.726572725547	0.407301362263
C	0.511507116037	3.215607773502	-1.271323183921
H	0.413525535618	2.293740434857	-1.869715390793
C	1.878729313093	3.813331341398	-1.561448894152
C	-0.618962354695	4.186897026394	-1.567805837235
H	-1.592759382281	3.746403754364	-1.321911242131
H	-0.501735222790	5.132357128346	-1.012923460713
H	-0.619754942706	4.430042515842	-2.640099493525
H	2.067141861060	4.725927864371	-0.972285720788
H	1.942594021738	4.080572439070	-2.625916233691
H	2.671585196046	3.084328177554	-1.353230386912
C	0.238123640593	0.308353795102	2.711090058363
H	0.216391291859	-0.519756215638	1.991046846189

C	-1.058716686156	0.318491358914	3.503965137716
C	1.472919644329	0.156146725690	3.584347300157
H	2.384068858807	0.123799855818	2.970450069949
H	1.570204813522	0.977937331138	4.311592205228
H	1.411754492088	-0.784233999278	4.150800763188
H	-1.064739815918	1.116661534433	4.263728497700
H	-1.191513857378	-0.643233573705	4.019399648216
H	-1.919069765252	0.469555297700	2.836887621609
F	1.065793619550	0.430483084135	-2.352367503693

66

TS13_opt, 1-A (C1): E(RPBE0-D3BJ/def2SVP) = -3156.78348489

G_corr(298K) = 0.466374

E(COSMO-PBE0-D3BJ/def2TZVP) = -3158.86592104

Ni	0.475434312990	0.092297010101	-0.049412058746
N	3.186293036885	-0.025262088369	1.337855965291
N	2.915295487415	-1.655212401110	-0.030322016927
C	2.314166741990	-0.536326917699	0.437679703227
C	4.306377616069	-0.808780569561	1.437527097867
H	5.133244323660	-0.586973493814	2.106755856190
C	4.135055366204	-1.844491945108	0.564047815252
H	4.788372064619	-2.681966770199	0.334227181667
C	-1.207815371981	-0.167763250077	0.660999543655
C	-1.610761354598	-1.494344534061	0.785431938712
C	-2.789201299210	-1.866877976801	1.424629719943
C	-3.603779153059	-0.873473955279	1.969300439487
F	-0.854968058269	-2.472461610792	0.260431736500
F	-3.137235836427	-3.135099714894	1.524153897542
F	-4.719335929536	-1.200243488232	2.584921000861
C	-2.045748812476	0.796990755194	1.208214402251
C	-3.232591893231	0.466996884047	1.860223230981
F	-1.733334603103	2.092531172519	1.123771156767
F	-3.999819137151	1.405281608203	2.379065301421
N	-1.091503885946	0.510571191961	-2.436805674105

N	-0.298699041621	2.324808139009	-1.584183007621
C	-0.382526588548	0.991217362717	-1.396119149787
C	-1.437276864567	1.536860852899	-3.289417361404
H	-2.007988370557	1.378891558712	-4.199993140413
C	-0.934656503328	2.681250313742	-2.753056545604
H	-0.985123330327	3.705662682965	-3.109217361200
C	2.311296934700	-2.533310945907	-1.040310836503
H	1.344012899476	-2.052254986789	-1.254536058999
C	2.048986117034	-3.914639064885	-0.465933845592
C	3.147037056710	-2.547002778193	-2.309429593896
H	3.305996531549	-1.527327651045	-2.689907906247
H	4.131116744032	-3.011479469495	-2.142851940228
H	2.634886518861	-3.131187112441	-3.087273462317
H	2.986125606816	-4.433024964078	-0.211088616897
H	1.520205369988	-4.530972687949	-1.206703809191
H	1.422753979418	-3.850749440271	0.434503007663
C	2.911990176104	1.176485532367	2.134371789657
H	1.998271646025	1.588029787404	1.676731381200
C	4.034194642612	2.190956060283	1.994109554609
C	2.609531461569	0.802732219423	3.576522848702
H	1.778478494692	0.084631824409	3.629680965721
H	3.487632720222	0.355351232604	4.067130931694
H	2.327924724982	1.699283243544	4.146826819610
H	4.966640064795	1.834065657729	2.457616219061
H	3.755440386750	3.124897068944	2.502300069348
H	4.236931976944	2.418271660640	0.937613938349
C	-1.444426900153	-0.901511604408	-2.625740160541
H	-0.993976809317	-1.410966797427	-1.763862620176
C	-0.812967038146	-1.445994158412	-3.896757508390
C	-2.951192823235	-1.088902063110	-2.562510672932
H	-3.357414090687	-0.677794400150	-1.627091792494
H	-3.458463027556	-0.599162270422	-3.407903282891
H	-3.195620176029	-2.159813122771	-2.601968463197
H	-1.226054214272	-0.965927725813	-4.797040298140

H	-1.011175387722	-2.524220117299	-3.977446814241
H	0.276634987334	-1.293212147442	-3.894611136602
C	0.441998729663	3.222918988450	-0.688598790408
H	0.464116053309	2.680363049976	0.268895448382
C	-0.308668129401	4.524375832968	-0.476531985880
C	1.863540864393	3.415394976921	-1.193793077626
H	2.380234100576	2.448136839981	-1.288969481204
H	1.873503209567	3.911480277814	-2.176291045154
H	2.432082388128	4.043738614868	-0.492988852223
H	-0.334328806906	5.138572834924	-1.389597005011
H	0.198284677746	5.117002807634	0.297605701556
H	-1.337740967265	4.333372047709	-0.143068227686

60

TS14_opt, 1-A (C1): E(RPBE0-D3BJ/def2SVP) = -3256.56925955

G_corr(298K) = 0.378217

E(COSMO-PBE0-D3BJ/def2TZVP) = -3258.73168720

Ni	0.000113825237	0.162928922162	-0.301242970277
N	-1.070795742350	-2.555311017181	-0.011384416252
N	1.069107159174	-2.555916396629	-0.011387670283
C	-0.000605143979	-1.718159730200	-0.020754745647
C	-0.680721365825	-3.882964491359	-0.006358311425
H	-1.394766469424	-4.701351507821	-0.019144197642
C	0.678295799918	-3.883348125147	-0.006354196546
H	1.391883325810	-4.702134686975	-0.019137544516
C	2.403343969373	-2.051996749097	0.013920506709
C	3.141286981096	-2.160626405903	1.201604810587
C	2.908179049998	-1.399168006806	-1.120570111116
C	4.419783029908	-1.602192053473	1.230998893466
C	4.188110499519	-0.848559115881	-1.033950829437
C	4.957721900633	-0.935033351607	0.127574419049
H	5.004477053689	-1.673458367109	2.152510367355
H	4.592303180284	-0.328040641222	-1.906638433336
C	-2.404768041127	-2.050701014253	0.013986450730

C	-3.142849472034	-2.159279644585	1.201589643678
C	-2.909208938604	-1.397315105986	-1.120363716037
C	-4.421093222014	-1.600263336737	1.231026158986
C	-4.188883727484	-0.846119609563	-1.033694730484
C	-4.958641734068	-0.932570612778	0.127738751746
H	-5.005893349075	-1.671507859631	2.152472036093
H	-4.592759761969	-0.325178133581	-1.906275947850
C	2.110469929266	-1.260382218027	-2.383375752815
H	1.506398109458	-0.338153809081	-2.372298908080
H	2.777685135940	-1.202454048680	-3.254759862150
H	1.420619766152	-2.103193612666	-2.528550847586
C	6.311029722007	-0.290704776594	0.196864451498
H	6.213926866082	0.795683633423	0.357900331916
H	6.911950058437	-0.696332968356	1.022834078498
H	6.872103492195	-0.430750031539	-0.738889341967
C	2.558622736167	-2.821120489667	2.416633070695
H	3.178350983480	-2.624941426862	3.301641352112
H	1.541267784655	-2.451140168022	2.617889774414
H	2.482918571389	-3.913838993162	2.298406811117
C	-6.311675435748	-0.287669505120	0.197078659130
H	-6.912883611201	-0.693349825501	1.022812235267
H	-6.214161207106	0.798623110329	0.358521188749
H	-6.872665540587	-0.427146180937	-0.738809040022
C	-2.111332733864	-1.258585716992	-2.383067655623
H	-2.778460078005	-1.199504546426	-3.254438776857
H	-1.506338410956	-0.336967143536	-2.371478901655
H	-1.422295865687	-2.101987354349	-2.528695980168
C	-2.560607827257	-2.820336276609	2.416517202584
H	-2.485354200357	-3.913056240903	2.298031414604
H	-1.543119542863	-2.450843791799	2.617994461434
H	-3.180362066762	-2.624116667805	3.301498535388
C	-1.209337466623	3.738261444772	0.425214050373
C	-1.211234784347	2.541571570488	-0.274564771345
C	0.001420888333	4.336703055182	0.786891149736

C	0.000823347175	1.910906914584	-0.655616638500
C	1.211884893279	3.737336607843	0.425770879844
C	1.213190702469	2.540640374584	-0.274006101994
F	2.374522151617	2.008464233470	-0.660627924381
F	0.000939204491	1.333637664205	-2.120620630746
F	-2.372796420207	2.010252129782	-0.661679846983
F	-2.349609464369	4.308345967890	0.790277483523
F	0.001694175063	5.470716456869	1.475361270363
F	2.352426732851	4.306559275775	0.791336730594

67

TS1_opt, 1-A (C1): E(RPBE0-D3BJ/def2SVP) = -3256.59765184

G_corr(298K) = 0.466554

E(COSMO-PBE0-D3BJ/def2TZVP) = -3258.76364202

Ni	0.173771838297	-0.680385492171	-0.307000472405
F	0.242536979991	-0.691804407656	2.044712320907
C	-0.196112669469	0.645520370856	0.976202628030
C	2.064248583826	-0.457533943624	-0.582548646079
C	-1.601535762959	-1.299662138546	-0.607987868025
N	-2.139257692409	-2.417950437698	-0.068844718422
C	-3.399252818161	-2.653073683089	-0.569667227221
N	-2.548992965303	-0.833858339537	-1.453209951857
C	-3.662475670451	-1.645583391477	-1.446813094340
N	2.654562477922	0.300333501425	-1.539010151370
C	4.025456382113	0.186885332711	-1.491264381046
N	3.093396750249	-1.060092216864	0.056262393645
C	4.303073504853	-0.678061813799	-0.477786911064
H	-4.546649666995	-1.448352447086	-2.045987209604
H	-4.013687974618	-3.494849090411	-0.266674259282
H	4.690913821509	0.718447535476	-2.165657258292
H	5.256723749308	-1.039760919407	-0.103873842511
C	0.708348334569	1.723354278543	1.145160356502
C	0.323739156687	3.034719211933	1.396543322735
C	-1.013613176590	3.361059192957	1.590298084631

C	-1.935686990101	2.318753510176	1.546671923323
C	-1.528438228733	1.017165845859	1.293523127092
C	-1.434510530039	-3.162406911205	0.982359337834
H	-0.912612456634	-2.379928534196	1.558923211291
C	-2.415063593253	0.437786700220	-2.158845687899
H	-1.467403134237	0.842054412109	-1.768454178319
C	1.896457622835	1.198773101866	-2.404677798784
H	0.854978220990	1.014826997049	-2.091889111775
C	2.913396359098	-1.898032675945	1.242985052688
H	1.823360385499	-1.916674370056	1.389221898308
C	-0.397946502169	-4.087011485436	0.362324709335
H	0.301239169655	-3.499183502766	-0.252856506670
H	0.183691390424	-4.592583547419	1.147437264680
H	-0.866806572639	-4.853836476879	-0.274996942120
C	-2.406132091142	-3.882314722468	1.897106637027
H	-2.926522027668	-4.711326868663	1.390266307550
H	-1.853329490344	-4.312161098026	2.744262901599
H	-3.154806638813	-3.185963809965	2.302140144126
C	-3.537799572313	1.390075086796	-1.777559664281
H	-3.352042069221	2.382060447119	-2.214258130688
H	-4.513488394751	1.039596620316	-2.150016937094
H	-3.600185229686	1.499713172061	-0.686199779072
C	-2.283428008644	0.219237180692	-3.657358911704
H	-2.110971350681	1.177964302041	-4.168517771974
H	-1.438241936106	-0.449394733128	-3.878737683401
H	-3.196851071518	-0.227530948638	-4.081275278931
C	3.502705967410	-1.214243029808	2.465475442714
H	4.599256811771	-1.121527591313	2.400576722911
H	3.060813600510	-0.215343205543	2.574413224609
H	3.262796552674	-1.795628717282	3.367355338355
C	3.449908873914	-3.299148633466	1.004534062098
H	4.543067518662	-3.302901944247	0.863866827883
H	3.226281733399	-3.937917177112	1.871574668780
H	2.987774364262	-3.753608096660	0.115450794665

C	2.248011366808	2.651149684809	-2.121420157868
H	1.579491686871	3.318072682026	-2.685289308329
H	2.144907444323	2.874585231972	-1.051143276697
H	3.282081227945	2.881013121403	-2.424525414147
C	2.049891487596	0.811864684966	-3.866375721373
H	3.086520906362	0.945540747977	-4.214705409474
H	1.766573747213	-0.239442858254	-4.023491193846
H	1.404093206772	1.442538690160	-4.495029050794
F	1.250375508653	3.990923523399	1.429736320944
F	2.036170120224	1.541829589168	1.029489417926
F	-2.505824534150	0.098834185643	1.327788641584
F	-3.230065852107	2.574989609757	1.732835233761
F	-1.395857905264	4.613853909213	1.824598928840

67

TS2_opt, 1-A (C1): E(RPBE0-D3BJ/def2SVP) = -3256.59187453

G_corr(298K) = 0.469433

E(COSMO-PBE0-D3BJ/def2TZVP) = -3258.76553132

Ni	0.525795606429	-0.232823558409	-0.666171009754
C	-0.282355134850	0.655092181497	0.777919712654
F	1.343027504379	1.344833713683	1.549504968178
C	2.326689779851	0.292221542182	-0.363670900838
C	-1.074209267323	-1.190516897317	-1.245604803025
N	3.006945927113	1.343594552667	-0.869141568163
C	4.319216910395	1.327217349819	-0.445785735803
N	3.240973484377	-0.414325460807	0.340434998164
C	4.467065993339	0.214010688405	0.318730544061
N	-1.193963331253	-2.536770550800	-1.118822089094
C	-2.424150605041	-2.974623325349	-1.553677239544
N	-2.253865389979	-0.798103164433	-1.779883937621
C	-3.096543673125	-1.869529934685	-1.972612225913
H	5.338339709417	-0.173276343104	0.838799971311
H	5.036905158262	2.095040859511	-0.718879319590
H	-2.727414412827	-4.017284408644	-1.528408996116

H	-4.097411029903	-1.767250785637	-2.381813191470
C	-1.029021209331	1.809048429487	0.509816067342
C	-2.134771374720	2.196987146955	1.252068159333
C	-2.610643151240	1.378595874343	2.274200923924
C	-1.930949647584	0.190102901124	2.541761699072
C	-0.829628275331	-0.161462419513	1.777466214082
F	-2.379462878898	-0.625582962346	3.492343048461
F	-3.681986263087	1.727306803295	2.985441370968
F	-2.796185046856	3.309136830949	0.936553670274
F	-0.669727183999	2.620729935438	-0.512012983119
F	-0.245212538310	-1.348689538454	2.057754879692
C	2.336405659990	2.491953533242	-1.475953508489
H	1.321071834669	2.124667706677	-1.685853126688
C	2.865616658110	-1.495110623484	1.248519144634
H	1.803385832380	-1.665971001658	1.022825793797
C	-0.125030005389	-3.392456165583	-0.604807100142
H	0.659371933400	-2.674609851445	-0.315239772461
C	-2.581440257455	0.586982056945	-2.119524405539
H	-1.737306910807	1.169177268020	-1.732783627131
C	3.655766639090	-2.761084825285	0.966547606043
H	3.299064262659	-3.577096102913	1.612479571881
H	3.547670123442	-3.074735367138	-0.083563367753
H	4.729534551974	-2.625337588576	1.172527630490
C	2.936536026029	-1.008482519065	2.687186986562
H	2.373746393416	-0.063151282776	2.746696151264
H	2.487029538095	-1.753262856822	3.360159216972
H	3.979155577175	-0.842712387095	3.004332822046
C	3.004674178899	2.906737451962	-2.774910570801
H	3.057688482371	2.067834080213	-3.485307640463
H	2.433801944411	3.721771685656	-3.243099105767
H	4.027249682342	3.280162487496	-2.605481788010
C	2.225766118502	3.604736808130	-0.443752765016
H	1.550760058163	4.394564876970	-0.803362584404
H	1.830505651425	3.170389374977	0.488255003447

H	3.211967386856	4.055927131727	-0.245548362628
C	-3.849113418339	1.034374272229	-1.409371849571
H	-3.807502406739	0.775891835117	-0.342392135329
H	-3.951846440659	2.125921518383	-1.483604427311
H	-4.749356203050	0.576588619302	-1.849811537521
C	-2.645083686210	0.766470765105	-3.628653887306
H	-1.700947737465	0.457577685794	-4.101048461926
H	-3.463569083622	0.178009112501	-4.073118915412
H	-2.821940488395	1.824248764761	-3.871421792127
C	-0.572430830369	-4.152172226538	0.631511226858
H	0.277441475201	-4.709277631022	1.052260088970
H	-0.941400388136	-3.457144936773	1.396859567936
H	-1.362638835181	-4.881813147973	0.393736937786
C	0.421271214541	-4.291151998139	-1.703214992593
H	1.286340704862	-4.858288828509	-1.329681860999
H	-0.333677738498	-5.017256774078	-2.043866805460
H	0.745155369259	-3.698265155795	-2.571363877222

67

TS3_opt, 1-A (C1): E(RPBE0-D3BJ/def2SVP) = -3256.61599781

G_corr(298K) = 0.466626

E(COSMO-PBE0-D3BJ/def2TZVP) = -3258.77865319

Ni	0.673916651637	0.364299236771	0.081378047759
C	-0.960552445213	-0.491342105235	-0.144491615314
F	0.486211804100	2.328788465768	-1.525360964400
C	-0.017478584490	2.083767918590	-0.084932618904
C	1.784481254798	-1.216100901386	0.190120679166
N	0.851289361800	2.768746058272	0.777092307781
C	0.236998185460	3.922753641144	1.267436563736
N	-1.235508378549	2.725497211543	0.100904149958
C	-1.045617835945	3.885563997806	0.847559910456
N	1.778202827686	-2.234207600563	1.078881232688
C	2.636716048463	-3.239970833558	0.688813326958
N	2.663391863394	-1.598405740400	-0.765012329079

C	3.202108162489	-2.834478679530	-0.481023536280
H	-1.862495147734	4.566617928307	1.069155777877
H	0.737628312581	4.625371873805	1.927633956696
H	2.778354798508	-4.154296721175	1.258150426590
H	3.928717203502	-3.328663472172	-1.119520588848
C	-1.270745713035	-1.220612935079	-1.285212502288
C	-2.466613881467	-1.920129693921	-1.435628414029
C	-3.398312115526	-1.906255078626	-0.398361202576
C	-3.115062999514	-1.199698148169	0.768563174237
C	-1.904431327064	-0.520342173811	0.873616651464
F	-4.002124015742	-1.176136165373	1.757234014392
F	-4.544637118495	-2.562429649276	-0.521754764385
F	-2.733489140956	-2.596898510433	-2.546420298539
F	-0.407593084685	-1.266072115475	-2.311767943094
F	-1.677238885037	0.134703888050	2.023261705452
C	2.267657852410	2.472143340105	0.716947719546
H	2.344636524067	1.541157909589	0.101080537995
C	-2.405456145462	2.443044023586	-0.710666962358
H	-2.235666073686	1.430353496200	-1.102438912347
C	0.905597135813	-2.255692801497	2.253008423993
H	0.388684219329	-1.285591493311	2.212847344679
C	2.948021399968	-0.771304781095	-1.939000091169
H	2.187081136989	0.026717850530	-1.891840179674
C	-3.670181986924	2.421372004004	0.137187771135
H	-4.532732520345	2.113376952024	-0.472119782381
H	-3.566637861964	1.718428558623	0.975675320783
H	-3.898181737592	3.417233272697	0.550028654665
C	-2.501506559570	3.398649206002	-1.895573294645
H	-1.562385286059	3.370386398554	-2.464513053196
H	-3.334456595453	3.118320516958	-2.558391955067
H	-2.671480772292	4.433103086675	-1.555130857801
C	2.848614565241	2.168061542898	2.089725720683
H	2.284913406375	1.358451030691	2.575587624538
H	3.904804618431	1.868414063009	2.012754034187

H	2.796618950123	3.057144889784	2.737586756738
C	3.042615391460	3.542054678535	-0.043866159364
H	4.096464531712	3.252159130357	-0.173815845688
H	2.578842402802	3.688666771092	-1.028287111074
H	3.019183294885	4.497517240183	0.503064559161
C	2.735869337571	-1.555646680441	-3.222064197690
H	1.726788994809	-1.988273281259	-3.241755976491
H	2.840554520651	-0.885726618395	-4.087335574368
H	3.476649627459	-2.363291590898	-3.334562711710
C	4.329873103018	-0.145120107881	-1.833385977959
H	4.434244288638	0.422338911855	-0.896486855816
H	5.121107776809	-0.911208748437	-1.859502186584
H	4.498115094841	0.544976145821	-2.672764912810
C	-0.134581182360	-3.357764368583	2.128685292955
H	-0.856300171104	-3.286713379963	2.955180512740
H	-0.687944341175	-3.259162665179	1.183801584189
H	0.325337812815	-4.358274025737	2.164980935160
C	1.719879405755	-2.331404440177	3.534011200700
H	1.054590306044	-2.255863217155	4.406088926748
H	2.267974650409	-3.283786861371	3.613473371142
H	2.448179641559	-1.508555044701	3.584188393742

67

TS4_opt, 1-A (C1): E(UPBE0-D3BJ/def2SVP) = -3256.58465397

G_corr(298K) = 0.465859

E_BS(COSMO-PBE0-D3BJ/def2TZVP) = -3258.74860175 <S**2> = 0.9477

E_T(COSMO-PBE0-D3BJ/def2TZVP) = -3258.74619833 <S**2> = 2.0233

C	4.758474354853	-0.761414879393	0.025000082364
C	4.779990574928	0.592959981581	-0.085474977445
C	2.613594215193	-0.054389570025	0.073177899053
N	3.434639166410	-1.130859693162	0.122636233921
N	3.464499254838	1.000630513293	-0.055810177657
Ni	0.685160923137	0.212734122620	0.001349523159
C	-0.994628414344	1.159097768987	-0.260140022809

N	-1.719547303430	1.319905200672	-1.392861136294
N	-1.707825922810	1.812567067578	0.690553215202
C	-2.864128623681	2.049445983302	-1.156048580547
C	-2.856493417838	2.363364263440	0.166629986260
H	-3.602485713001	2.263580040459	-1.921206766234
H	-3.585445414204	2.900427367272	0.764092568037
H	5.574155356642	-1.478473441205	0.042795877256
H	5.615928535402	1.280161756506	-0.181131200590
C	-0.857194903168	-2.137751093419	-0.349023123133
C	-0.729001150480	-1.798030492106	1.020324386159
C	-2.099781038168	-1.964922421417	-0.974155181695
C	-1.863950843292	-1.375851904859	1.723931873967
C	-3.199252437017	-1.513950986873	-0.282073552262
C	-3.065339678320	-1.161764558971	1.078683439128
F	0.268055533488	-1.759540232142	-1.178528374112
F	-2.236284596919	-2.278142562801	-2.276807489516
F	-4.403031590252	-1.443017678549	-0.860223369739
F	-4.085577894571	-0.551049618295	1.700370665660
F	-1.764725104263	-1.079201830304	3.029274697290
F	0.269730116947	-2.382358141156	1.774575631701
C	-1.282700967879	0.786168421184	-2.686531794223
H	-0.826529391924	-0.186478184976	-2.456111852682
C	-0.215315674883	1.691026703870	-3.284680038759
C	-2.458888125568	0.548685962634	-3.614703021164
H	-3.222560916924	-0.074744665472	-3.129584643942
H	-2.917186259625	1.491670705773	-3.952983643965
H	-2.109630135491	0.012252360512	-4.507724594449
H	-0.612328790962	2.699130957049	-3.483894240075
H	0.158252231058	1.270466372675	-4.229957178148
H	0.630294958367	1.775576362149	-2.585841380655
C	-1.247779080901	1.922095513005	2.076482168363
H	-0.729738953536	0.970718373673	2.272092801836
C	-2.406467338886	2.041874222653	3.048671410262
C	-0.248634734633	3.062185280976	2.207290126198

H	0.599776304227	2.904538559015	1.526935895625
H	-0.716221683593	4.028889523985	1.962446386280
H	0.141274286438	3.115791746172	3.234450670159
H	-2.914093922023	3.015961026376	2.963244666678
H	-2.024368564745	1.959281537537	4.075637198498
H	-3.137585044896	1.238187586051	2.893464318489
C	2.965427244894	-2.514010324118	0.226031480018
H	1.887170909193	-2.424235280501	0.384236818349
C	3.565105221350	-3.200696915496	1.440992277933
C	3.203941556417	-3.258007345856	-1.077022064570
H	2.700919187843	-2.740979376366	-1.905929945275
H	4.277506541166	-3.350599681598	-1.309086067003
H	2.782643944136	-4.271338750466	-1.008018626526
H	4.654598990700	-3.339943270588	1.349442366683
H	3.109518408290	-4.193750410306	1.561930313666
H	3.357829261995	-2.622265900522	2.352697710380
C	2.996512468515	2.376018191299	-0.179709619102
H	1.896532516445	2.269918800884	-0.170892039157
C	3.402420709628	2.975959171929	-1.516196642073
C	3.430511459367	3.214075037221	1.011664411775
H	3.115259014352	2.743931514051	1.954292738107
H	4.524898830033	3.336869281619	1.038271862474
H	2.982295812957	4.216912402493	0.957150728385
H	4.494588343995	3.097727571693	-1.590814921964
H	2.946247143865	3.968980637060	-1.639738617324
H	3.069595564551	2.336371938892	-2.346362191286

107

TS5_opt, 1-A (C1): E(RPBE0-D3BJ/def2SVP) = -4179.16529146

G_corr(298K) = 0.761535

E(COSMO-PBE0-D3BJ/def2TZVP) = -4182.26991407

C	2.075238177095	2.195503051671	3.076207788514
C	1.160562653302	3.114549910552	2.685803520895
C	0.962497795445	1.320293668662	1.295806066667

N	1.945479265998	1.123164317921	2.216305563466
N	0.497423185815	2.566278130777	1.603035809458
Ni	0.293195580202	0.017291882637	-0.076225363297
C	-0.184125287594	-0.535559957268	-1.837558778913
N	0.768826754800	-0.637105421688	-2.809434946190
N	-1.346939429635	-0.794692530824	-2.512966213208
C	0.223887604293	-0.939843094861	-4.045522110544
C	-1.110980050363	-1.032008691055	-3.857005670261
H	0.836865217297	-1.062626413777	-4.933057105566
H	-1.917846772131	-1.241185891419	-4.551448902098
H	2.816540397497	2.207777552721	3.869784144007
H	0.927968918119	4.105964340564	3.063239379357
C	-2.660464615390	-0.896923234033	-1.957086486065
C	-3.374851470176	-2.098939958488	-2.095282200864
C	-3.217326578316	0.217574422027	-1.312885323433
C	-4.642326416902	-2.174204571394	-1.512523391101
C	-4.474202441450	0.078588806987	-0.725148825117
C	-5.196928543733	-1.113951975533	-0.795802584422
H	-5.190660956115	-3.117330249296	-1.581787376859
H	-4.898913366073	0.935556406507	-0.194597294938
C	2.169956950265	-0.424724576876	-2.634937406685
C	2.748588430037	0.696209697688	-3.254238637285
C	2.946384201748	-1.391278141773	-1.971461658104
C	4.136843614648	0.837370703841	-3.199005486981
C	4.330457219835	-1.205114483109	-1.958916659058
C	4.945545317119	-0.109356853709	-2.567312656729
H	4.597308618763	1.707910921795	-3.675100572052
H	4.946117302112	-1.957636848341	-1.459972573062
C	2.891114629874	0.049307766250	2.241570935464
C	2.756060011339	-0.980201629622	3.179455440820
C	3.978641135679	0.128769356810	1.365326250115
C	3.735010074867	-1.973878462378	3.188676048105
C	4.936422367787	-0.886107555139	1.418682728679
C	4.824307547058	-1.951906643062	2.313376649938

H	3.635222066431	-2.799483225474	3.899267258630
H	5.796543068525	-0.835159439774	0.745363074082
C	-0.474754763315	3.319448693374	0.876611670609
C	-0.112906562589	3.806109833426	-0.392044062359
C	-1.700759773854	3.655582468900	1.470239744042
C	-1.020923745537	4.610976472213	-1.076991766913
C	-2.567384184690	4.482688739456	0.745882203676
C	-2.256689493219	4.961704833194	-0.525759080435
H	-0.746712938814	4.988478958450	-2.066328528523
H	-3.524928935289	4.754481187406	1.199726128483
C	-2.490906221832	1.519430644291	-1.264666519377
H	-1.571600535966	1.428890655223	-0.662558224311
H	-2.169157731061	1.836627517965	-2.269170349644
H	-3.116152288455	2.308431966369	-0.831118237143
C	-2.819208034023	-3.303621789788	-2.800153398371
H	-3.028580248871	-3.275925864958	-3.882208114368
H	-1.734014864949	-3.387345537278	-2.666973197879
H	-3.277494977121	-4.215901091210	-2.397677210816
C	-6.500754773101	-1.269852267377	-0.072166085696
H	-7.171242073088	-1.972111671507	-0.588296708696
H	-6.315236114687	-1.671266807998	0.937409290704
H	-7.022083834816	-0.308227128279	0.039370832433
C	2.331380713234	-2.577127122162	-1.301229202976
H	1.548974011915	-3.034440708211	-1.922692265482
H	3.096802676567	-3.332760531481	-1.079706126652
H	1.846128634903	-2.289543253548	-0.352463734332
C	1.914514025797	1.702671107608	-3.993918035045
H	1.758753499838	1.401153096170	-5.042704160983
H	0.919406403265	1.822257824521	-3.544315992065
H	2.411929911577	2.682322705271	-4.006977023080
C	6.434835616867	0.062554654058	-2.507134941737
H	6.952635301736	-0.907230389010	-2.533558020666
H	6.807375745694	0.675464968354	-3.340228258419
H	6.736087888015	0.564915924544	-1.571744483255

C	1.213884068192	3.464752512216	-0.996369297393
H	2.040803483467	3.656516104844	-0.296345381982
H	1.258911857582	2.392491130544	-1.244689355047
H	1.388961597625	4.042634023607	-1.913031014872
C	-2.132866139796	3.148611583929	2.814648571918
H	-1.290651489757	2.935069881919	3.483534451485
H	-2.795295121237	3.876750176359	3.303196966879
H	-2.684245943523	2.202105208678	2.712300010903
C	-3.231755379959	5.800227796274	-1.298250308238
H	-2.718956740344	6.558729653591	-1.907715922264
H	-3.821727249203	5.172992096045	-1.987723693434
H	-3.940725036127	6.313489471231	-0.633305892083
C	1.610806154153	-1.010418319963	4.144243272598
H	0.716836924178	-0.547042443375	3.713126750705
H	1.362385827038	-2.045887858648	4.412444415712
H	1.864577363877	-0.474774578104	5.075047657302
C	4.104737586670	1.271789626720	0.404523540079
H	3.915054329136	2.237479786082	0.897819164571
H	5.107440213172	1.303623384919	-0.039105033119
H	3.377886230547	1.176818688360	-0.417070672086
C	5.833673481536	-3.063238970554	2.319002156509
H	6.045522485961	-3.412632853034	3.340416319201
H	5.460709589993	-3.932046111572	1.750952530464
H	6.782595309909	-2.750948645105	1.859504891616
C	-0.780728604861	-1.193096019544	0.895591254358
C	-1.562157733755	-0.662099542567	1.943627170257
C	-1.310999172375	-2.395709061633	0.371271862808
C	-2.777572874489	-1.193293136825	2.358211694140
F	-1.142138575311	0.417896352665	2.631146996245
C	-2.499022922239	-2.964154025004	0.790993301413
F	-0.639344633906	-3.042863213347	-0.592394150953
C	-3.270674680263	-2.352929636235	1.778275658557
F	-3.486630017814	-0.561723514248	3.294003542316
F	-2.942717303453	-4.082568155419	0.220057877972

F	-4.453828428032	-2.858710003473	2.132168997238
F	0.814926089657	-1.591605917174	1.381567303907

107

TS6_opt, 1-A (C1): E(RPBE0-D3BJ/def2SVP) = -4179.16690944

G_corr(298K) = 0.763981

E(COSMO-PBE0-D3BJ/def2TZVP) = -4182.28064853

Ni	0.074361819871	-0.314430175612	-0.182242844929
N	1.298204647599	-2.548608151748	1.287807280337
N	-0.749709517254	-2.959700793108	0.822096389596
C	0.218262940072	-2.023074592921	0.654690752925
C	0.993076022883	-3.755021471101	1.888368040343
H	1.730670445545	-4.316832752682	2.453384052692
C	-0.303109064160	-4.016244788414	1.593997291178
H	-0.945425486160	-4.854144192172	1.847393648124
C	-0.424610197370	0.632716432602	1.377603563832
C	-1.739336811568	1.118402271569	1.525928204700
C	-2.024733360369	2.308081510706	2.172520190029
C	-0.997150455006	3.160114337233	2.579769818682
F	-2.768283591163	0.353378482932	1.137748767883
F	-3.289005926825	2.702968211471	2.335839927451
F	-1.279379619784	4.348390989043	3.117571873279
C	0.578675446310	1.532573917950	1.775218100916
C	0.319222582102	2.751728939506	2.383333961975
F	1.870169294503	1.174204957906	1.627797000343
F	1.324738081830	3.558040110772	2.727037778885
N	-0.937554355067	2.004794761815	-1.859219955677
N	1.181820843159	1.792472062231	-2.009031467703
C	0.094025815901	1.241887570801	-1.403958943164
C	-0.506124965634	2.998581711085	-2.715688294286
H	-1.196086232039	3.697077377370	-3.178962841100
C	0.837332126024	2.864766415944	-2.813951021103
H	1.578922735249	3.416662547344	-3.384006920247
F	-0.413293491417	-0.721043294860	2.576685515102

C	2.614415113611	-1.991673363964	1.313106976260
C	3.417841790427	-2.169714133231	0.176573003066
C	3.100430942623	-1.386734620055	2.480479598243
C	4.725818268207	-1.689916137487	0.211234872432
C	4.412254236864	-0.904385492336	2.454118613102
C	5.236319227420	-1.039515326562	1.337620337695
H	5.364581406842	-1.838730218481	-0.661938457076
H	4.799856679020	-0.410446375533	3.349628367602
C	6.629403828649	-0.481933745961	1.339792565887
H	7.045022794910	-0.434566087275	2.356449525998
H	7.308482735564	-1.084401341544	0.718579302270
H	6.636650047621	0.544851931346	0.936507648053
C	2.267557197616	-1.271608969025	3.717829691895
H	2.702348648981	-0.532907461157	4.404865974638
H	1.230405328840	-0.987064657677	3.473332284044
H	2.228610024711	-2.236514173185	4.251884164288
C	2.887301381672	-2.885446111310	-1.029446301464
H	2.461839747474	-3.865662201999	-0.762913731089
H	2.079202745348	-2.309643008552	-1.504278363290
H	3.677727265904	-3.038279081638	-1.775632793977
C	-2.062327524348	-2.921682147853	0.260929410744
C	-2.213971150948	-3.290245175627	-1.083625746826
C	-3.159816291621	-2.612428857520	1.078232289328
C	-3.502457621813	-3.311199003406	-1.619742605486
C	-4.426065599407	-2.635772026936	0.488184920762
C	-4.620670406924	-2.981713555640	-0.849941305214
H	-3.634488654872	-3.596609347331	-2.667619637467
H	-5.290288632982	-2.373649824060	1.105467699946
C	-6.002786268874	-3.020175821183	-1.435725435856
H	-5.978721055228	-3.007977386659	-2.534976296368
H	-6.537700617928	-3.933779292115	-1.127727951549
H	-6.605174295341	-2.164345092903	-1.095652681067
C	-2.988885358287	-2.299647149885	2.531506792695
H	-3.860148849367	-1.748279390738	2.909638653045

H	-2.905291957753	-3.231575676210	3.116842926551
H	-2.077672733825	-1.704801610423	2.707844198483
C	-1.023134873444	-3.667073471078	-1.914782622865
H	-0.294716037789	-2.843137315120	-1.973361882201
H	-0.483508127681	-4.521742120466	-1.476617548619
H	-1.322815354410	-3.934435312559	-2.936860696478
C	-2.318095628997	1.797803332861	-1.562720184125
C	-3.016819567214	2.773895088462	-0.841759289512
C	-2.939310174510	0.640219427338	-2.051707135610
C	-4.360885662854	2.524341066378	-0.554474700316
C	-4.281897690410	0.441715552714	-1.742643205588
C	-5.002463131104	1.359944236303	-0.974811567386
H	-4.911487669180	3.255458147061	0.042548418159
H	-4.774267957263	-0.462966459388	-2.105304775673
C	-6.418592011326	1.070344579925	-0.575542575640
H	-6.966058854247	1.987503015020	-0.316491088179
H	-6.968190246710	0.552471244428	-1.375701828203
H	-6.433329989759	0.415536879960	0.312014967403
C	-2.182727211467	-0.347645329347	-2.885242752136
H	-2.862499481298	-1.072078681714	-3.349845783778
H	-1.598094117739	0.150119627726	-3.674105806650
H	-1.472251255104	-0.907218437594	-2.257639255624
C	-2.366219984817	4.048051963107	-0.388222667978
H	-1.292188640105	3.915712709803	-0.202425431670
H	-2.481623781853	4.845262806771	-1.141700561038
H	-2.825878158668	4.402887483666	0.543208894668
C	2.518418428454	1.285324214253	-2.005768807949
C	3.521231327422	1.977340287569	-1.311978875533
C	2.803342496710	0.192890738731	-2.838179442296
C	4.836936499429	1.536856898075	-1.474789956101
C	4.133572986846	-0.206867003046	-2.963733046823
C	5.165683414282	0.460282299182	-2.300421470623
H	5.632559805907	2.068210477492	-0.944933309345
H	4.370418342306	-1.053891122626	-3.613795388997

C	6.596685873858	0.052627054162	-2.495796745581
H	6.677647472031	-0.984633258786	-2.851439982532
H	7.087990498118	0.695549440682	-3.244841613551
H	7.171958043441	0.142246173622	-1.563400383858
C	1.710521627040	-0.497357929063	-3.599241090889
H	2.106294328686	-1.336288885093	-4.186250621571
H	0.932043021449	-0.885951271351	-2.923896234987
H	1.201285786053	0.196799023200	-4.287189198745
C	3.209890617670	3.147935805940	-0.429545840017
H	2.768086336869	2.806838072627	0.517208395364
H	4.123055643220	3.710391554907	-0.194288877543
H	2.490108215767	3.838761594745	-0.891477211144

107

TS7_opt, 1-A (C1): E(RPBE0-D3BJ/def2SVP) = -4179.18860574

G_corr(298K) = 0.762096

E(COSMO-PBE0-D3BJ/def2TZVP) = -4182.29529633

Ni	0.419522209690	-0.328220098056	0.202679807947
N	0.902636401067	1.330894203569	2.619174818973
N	0.869566572600	-0.813914681277	2.947718891673
C	0.417408779675	0.160442454794	2.081078515167
C	1.371411503158	1.097609652827	3.910486954393
H	1.700750452608	1.909795609849	4.552155602103
C	1.344881481118	-0.238543287182	4.119195001615
H	1.650156458378	-0.830929272420	4.976658781393
C	-1.195180266387	0.567513142077	0.022237007658
C	-2.350625494134	-0.045043015731	0.500306402472
C	-3.581084700715	0.593858883920	0.474619266487
C	-3.679466294606	1.882904518545	-0.046170089068
F	-2.321942951075	-1.299330410330	0.940033251364
F	-4.679659293480	-0.015165822628	0.915265220660
F	-4.861522139814	2.487508669108	-0.105188454561
C	-1.325934099888	1.837372623341	-0.504326210625
C	-2.545182357791	2.512151391897	-0.541637900579

F	-0.271342424296	2.468098032930	-1.046886203773
F	-2.632741083556	3.730450964505	-1.073495090808
N	-0.454824731308	-1.015366057029	-2.619392242385
N	1.659048336404	-0.741324742304	-2.514551554890
C	0.540238689364	-0.741981282514	-1.728937470296
C	0.027600449007	-1.181088972018	-3.902049212694
H	-0.632846356672	-1.410818484783	-4.732550111364
C	1.366294105635	-1.004259068048	-3.839129096123
H	2.138785129889	-1.034909227453	-4.601737005505
F	-1.292781517920	0.197018235533	2.707715207078
C	0.924682843092	2.595352566555	1.965819575596
C	2.107381438667	2.964296206429	1.306065304134
C	-0.191585914205	3.450049214848	1.996955460347
C	2.141245361453	4.176737029734	0.615382459092
C	-0.116505640710	4.640816605779	1.269116409152
C	1.026280262785	5.014760395592	0.560635457373
H	3.058998507377	4.465694023835	0.094279318134
H	-0.993700327901	5.294118027062	1.251746504803
C	1.037163271116	6.269411696350	-0.262132171861
H	0.547956788517	7.103048738096	0.263597367146
H	2.060184053573	6.578384886369	-0.520785355323
H	0.485760787411	6.113031280889	-1.204312170099
C	-1.413414666517	3.124068309020	2.797356609851
H	-2.286535901181	3.668927619548	2.411235176966
H	-1.607098193920	2.038574180524	2.800492800394
H	-1.267306271779	3.434274089377	3.846692183434
C	3.303158707573	2.059263387471	1.335244248664
H	3.575802827443	1.777931666879	2.363618055421
H	3.106311993265	1.120068369458	0.795913735046
H	4.172263826783	2.537324191779	0.863808077842
C	0.658612744235	-2.173287427199	2.637378295443
C	1.437698127626	-2.696410894753	1.595770642567
C	-0.339008748586	-2.934531345644	3.263570302257
C	1.219232013103	-4.008295259734	1.175703259228

C	-0.519521664691	-4.246972155955	2.815364421320
C	0.235920284438	-4.799392163711	1.776827001390
H	1.832270928976	-4.425499712385	0.371530138232
H	-1.300416229022	-4.852644276364	3.284789095165
C	-0.031885967281	-6.193738170716	1.288989298848
H	0.866264492137	-6.647092548513	0.844722637514
H	-0.379738331881	-6.847365961503	2.101876251502
H	-0.816333258738	-6.193024999005	0.513152489187
C	-1.207033173119	-2.326553870974	4.318106034151
H	-2.071913567131	-2.968261019873	4.534045096440
H	-0.654515916642	-2.173911558400	5.259572873446
H	-1.539326240843	-1.335740605127	3.958839210083
C	2.505254932816	-1.834386908434	0.993605546918
H	2.169291387547	-1.082386162322	0.232943679073
H	3.025938777776	-1.245900751713	1.760500442482
H	3.238253757469	-2.431668521983	0.439731534520
C	-1.861559752183	-1.123029467433	-2.377071835071
C	-2.692620348410	-0.098870006683	-2.860329266001
C	-2.364958466521	-2.271343115482	-1.758119713734
C	-4.063546970753	-0.232057758707	-2.655046027962
C	-3.748187575294	-2.354175369203	-1.580214496150
C	-4.610294524906	-1.343314678175	-2.004407831892
H	-4.725268279713	0.572036726146	-2.989259112411
H	-4.158560908075	-3.229652760270	-1.070306262929
C	-6.081147137187	-1.406093634514	-1.721740431912
H	-6.674103108407	-1.033868307710	-2.570230437523
H	-6.407586462535	-2.429651152605	-1.490731298319
H	-6.314186412735	-0.775187255509	-0.849074699350
C	-1.457134452216	-3.347680671408	-1.247600339221
H	-2.012718097348	-4.281959239553	-1.089613704615
H	-0.625900928168	-3.550597294992	-1.939545889440
H	-1.018083850429	-3.055949773962	-0.280887361121
C	-2.135924560147	1.105984844639	-3.562192681068
H	-1.182519110616	1.432540890092	-3.125355699819

H	-1.957790081919	0.905248835075	-4.631966435256
H	-2.837758247169	1.948275848581	-3.496275135393
C	3.014377859951	-0.546940481332	-2.106469524908
C	3.509949892634	0.756020387829	-1.966414725262
C	3.833061699939	-1.678294927943	-1.972888864472
C	4.857121607729	0.900067770001	-1.625927913287
C	5.169389649667	-1.480048766171	-1.624565436701
C	5.697422033558	-0.199173908702	-1.436217685015
H	5.261155122197	1.909546060404	-1.510992621255
H	5.817457563939	-2.352184288940	-1.499710514341
C	7.126120617731	-0.009226943973	-1.019650592516
H	7.759808133113	-0.843846824903	-1.351626460064
H	7.543377602292	0.924004408085	-1.424312356185
H	7.205408752066	0.047277326545	0.078848697247
C	3.284818479473	-3.056388349439	-2.202085127634
H	3.951050528340	-3.820797906245	-1.779399952779
H	2.286823070878	-3.174582005325	-1.754248516514
H	3.175969874878	-3.269954051980	-3.277765495441
C	2.627120134319	1.944368984395	-2.181064842459
H	2.058274028304	1.857373596946	-3.119298100184
H	1.884969264453	2.051055739835	-1.376936482317
H	3.216953580113	2.869207525679	-2.217051881436

107

TS8_opt, 1-A (C1): E(RPBE0-D3BJ/def2SVP) = -4179.19091248

G_corr(298K) = 0.761769

E(COSMO-PBE0-D3BJ/def2TZVP) = -4182.29185282

Ni	-0.422650715884	0.218405192180	0.235098288883
N	0.748984804363	-1.204235048082	2.684135200333
N	-1.254025283257	-0.324244294550	2.792628463743
C	-0.060750929500	-0.244251102995	2.050815776692
C	0.161839192464	-1.612167304636	3.884510532087
H	0.696210294214	-2.251588364971	4.581713169604
C	-1.071613154743	-1.077622691431	3.954059830635

H	-1.841414202462	-1.147321142279	4.717705928595
C	1.333140923355	-0.129538447093	-0.277854296839
C	2.413982036112	0.733736270749	-0.126989603477
C	3.668881107102	0.457783943422	-0.668172067925
C	3.881607346829	-0.727867139149	-1.360954364659
F	2.318586959373	1.859963013357	0.570558524005
F	4.674737933461	1.314628556919	-0.505831302948
F	5.084197449982	-1.020949356857	-1.845040942269
C	1.594385840832	-1.314784576201	-0.951155620769
C	2.833389928187	-1.632856088203	-1.495369073851
F	0.631272406620	-2.242292177889	-1.062746635853
F	3.039015357324	-2.799381083482	-2.099891843647
N	-0.424117410208	2.084580044318	-2.084711081448
N	-1.699191995199	0.431880286768	-2.523060843931
C	-0.916595061568	0.941672972586	-1.534095398863
C	-0.893103199483	2.282418168033	-3.368291027736
H	-0.612970910776	3.152621762539	-3.953917054642
C	-1.700597805819	1.232034322945	-3.649426250766
H	-2.277920164543	0.979791600985	-4.534060800543
F	0.516839463332	1.173769772802	2.398027512737
C	1.876929061297	-1.890237033425	2.155207216727
C	1.683693922547	-3.170957686025	1.604786528746
C	3.160182888400	-1.324228417490	2.229297608298
C	2.781760704031	-3.846480934988	1.071785048206
C	4.228023168622	-2.035515146782	1.674070979362
C	4.060142875881	-3.284183414552	1.074272224683
H	2.626942114801	-4.826832762858	0.612157106310
H	5.224239596707	-1.582599278463	1.695267980051
C	5.207168416833	-3.962884225728	0.385366711409
H	6.155633202987	-3.801504954604	0.918768493615
H	5.042034601765	-5.045631242712	0.290092994857
H	5.328676831830	-3.554597196331	-0.631623774324
C	3.402326310678	-0.000768333270	2.891953237047
H	4.179214580084	0.565094077042	2.357376599363

H	2.489502367341	0.605362664466	2.927768561575
H	3.759080685819	-0.153773488572	3.924759586962
C	0.314569494505	-3.777610400694	1.548721707065
H	-0.092279109473	-3.962188816957	2.555235882366
H	-0.382588424643	-3.098778523986	1.040797523820
H	0.330307990817	-4.729224985231	1.000428432684
C	-2.494425711696	0.225155511985	2.387750454220
C	-3.383461710767	-0.582946235827	1.650857214680
C	-2.886314038772	1.501009723621	2.845818340182
C	-4.661633857327	-0.091817514227	1.378268984207
C	-4.157670813496	1.965670047748	2.502911626456
C	-5.062245098626	1.183339928982	1.779973220377
H	-5.363743506973	-0.729953416177	0.835799925381
H	-4.462398112987	2.959660150330	2.844132313392
C	-6.421393451628	1.712039127150	1.426539198605
H	-7.121990440761	0.898978629206	1.187946939498
H	-6.850772892363	2.305773286530	2.246984305956
H	-6.368008266118	2.372479582175	0.544259569863
C	-1.988407128269	2.307865194187	3.733144702673
H	-2.276721232023	3.368401699544	3.725376997993
H	-2.066358030824	1.949598050687	4.773662595961
H	-0.937175950548	2.196732468511	3.435145749824
C	-2.977552510710	-1.956741106024	1.206147313506
H	-2.288513231810	-1.913602579749	0.345481747285
H	-2.452975038984	-2.494618054177	2.008064181806
H	-3.851807656819	-2.540852155835	0.891224451692
C	0.495211543215	2.988817309004	-1.470889748479
C	1.774415259376	3.114729888913	-2.034865112288
C	0.091040016544	3.733071867177	-0.356181385812
C	2.650723919822	4.030973287571	-1.455897148917
C	1.008277758447	4.634936200975	0.184749415224
C	2.288412652158	4.797241611852	-0.345780440084
H	3.661459179693	4.120341085372	-1.863188472406
H	0.713612815402	5.217072551321	1.062150110815

C	3.279149016267	5.718209517040	0.301626096808
H	3.948605634506	6.180232829308	-0.438481648905
H	2.781003393449	6.518672068111	0.866874610168
H	3.910642860139	5.156372361825	1.009881467287
C	-1.254122842300	3.540933532779	0.274731107399
H	-1.515444430536	4.397095846214	0.911024120121
H	-2.050724377966	3.402577141258	-0.470835706697
H	-1.245643205870	2.647280057160	0.919624413572
C	2.218836889132	2.255333053856	-3.183752230656
H	1.831013929909	1.230170148323	-3.088673254435
H	1.881448134561	2.647446779631	-4.157084725389
H	3.315330412903	2.199322031093	-3.212156199534
C	-2.513690195737	-0.734616384651	-2.415878138225
C	-2.021030343176	-1.971638931099	-2.845441045571
C	-3.831739800760	-0.562650214301	-1.964867886408
C	-2.857732526017	-3.082418549054	-2.692057928511
C	-4.635405828925	-1.695998620499	-1.862216038335
C	-4.154829353099	-2.969089128567	-2.190323537264
H	-2.483666114729	-4.064592952436	-2.994441270665
H	-5.667162483053	-1.583904691229	-1.517027521043
C	-5.014096555181	-4.182667247945	-1.991356830148
H	-6.074764936646	-3.963129474257	-2.181958246905
H	-4.708263835631	-5.008826120419	-2.648514804026
H	-4.937299589910	-4.544776279967	-0.952156184704
C	-4.348606211169	0.810355138195	-1.654939789514
H	-5.398299407549	0.773944623348	-1.338996950404
H	-3.769361165374	1.284011103261	-0.848395962389
H	-4.279138742529	1.466983138308	-2.536774380759
C	-0.697966313064	-2.101891973950	-3.538556164270
H	-0.854503676919	-2.127079268672	-4.629988461132
H	-0.027029315849	-1.264013357211	-3.315120373039
H	-0.183832053049	-3.026874073098	-3.250005711850

107

TS9_opt, 1-A (C1): E(UPBE0-D3BJ/def2SVP) = -4179.14902629

G_corr(298K) = 0.755524

E_BS(COSMO-PBE0-D3BJ/def2TZVP) = -4182.26253131 <S**2> = 0.9417

E_T(COSMO-PBE0-D3BJ/def2TZVP) = -4182.24880441 <S**2> = 2.0158

C	-1.095905417197	3.963038563853	-1.828594829412
C	-2.215078689986	3.294215655973	-2.198085044131
C	-1.070669097233	1.954238287165	-0.749646788201
N	-0.416390934605	3.134903252825	-0.952733719416
N	-2.181359230097	2.080612404421	-1.533429428194
Ni	-0.580807612700	0.430228506709	0.262342234184
C	-0.418252270115	-0.963220169841	1.538339247616
N	-1.495724958829	-1.551647222591	2.129593045773
N	0.640723466781	-1.510921631452	2.211662341541
C	-1.128617141241	-2.420889858091	3.143249769917
C	0.223630549071	-2.391031964789	3.196517261961
H	-1.861902271751	-2.962244125236	3.733885429450
H	0.923966580032	-2.899619022645	3.851361879091
H	-0.718649583030	4.942904353012	-2.106664759517
H	-3.021818023676	3.561080543247	-2.874621514326
C	2.103333717867	-0.868519632796	-1.539702102707
C	3.485448638471	-0.678442954722	-1.630550864930
C	1.588939470940	-2.170538855105	-1.533875318429
C	4.334093507393	-1.762378410692	-1.749448754608
C	2.437931807747	-3.255229025692	-1.654454360078
C	3.820284874791	-3.062122418025	-1.699904366716
F	1.318997532561	0.147194263584	-1.033179904702
F	0.260204931765	-2.375967648208	-1.529550534901
F	1.949465610495	-4.495151254877	-1.678329872270
F	4.645660278126	-4.103636148874	-1.562289914321
F	5.650153671825	-1.584185478650	-1.877357195539
F	3.994663475703	0.560883005221	-1.689015026200
C	0.864142363912	3.452416154303	-0.409983000246
C	2.001173077107	3.247803955722	-1.206179982506
C	0.942049550671	3.993647500725	0.877994731347

C	3.243961550241	3.557184998411	-0.654810266816
C	2.208227220439	4.309428502280	1.376725002621
C	3.368961377355	4.085750558155	0.633174410571
H	4.142224628968	3.365914328647	-1.247491569530
H	2.288039895775	4.729452818851	2.383711531549
C	4.724864659761	4.406565271537	1.192423236246
H	5.414593617558	3.556473994353	1.071700687440
H	5.178713381614	5.264010925004	0.669355612597
H	4.673992070489	4.656339577557	2.261541636265
C	-0.297203852377	4.199448309118	1.694959069320
H	-0.062734722199	4.674199788740	2.657105730293
H	-1.029586104055	4.829472037184	1.166305623761
H	-0.796349107189	3.237032962087	1.893543676625
C	1.879770118990	2.732835790924	-2.608513600491
H	1.143754107768	1.920444152205	-2.670637383791
H	1.551891869945	3.531266204202	-3.294518435063
H	2.842311461450	2.346915234673	-2.964811257255
C	-3.156355216259	1.057947521783	-1.727714197576
C	-4.433629450880	1.229838185526	-1.182759270485
C	-2.826180937650	-0.052902953844	-2.520066398678
C	-5.403029575570	0.267984819615	-1.473991766790
C	-3.820094744292	-1.002872894275	-2.752711660969
C	-5.116803613861	-0.853613772327	-2.251870286447
H	-6.407060052346	0.392214798171	-1.061207487297
H	-3.573789583585	-1.880249345965	-3.357740968464
C	-6.166623717022	-1.886340540205	-2.541589882177
H	-7.100489091618	-1.676226920708	-2.001382867984
H	-6.402912873612	-1.925010394711	-3.617173344307
H	-5.824909997676	-2.892859676353	-2.252629707598
C	-1.447524398027	-0.206775056946	-3.081549686845
H	-1.359095465645	-1.122476542953	-3.679350123422
H	-1.171285571187	0.655469952273	-3.709386725347
H	-0.717314786145	-0.256286926897	-2.261452851836
C	-4.740207084026	2.401849617230	-0.298878951899

H	-3.962175565090	2.520408199512	0.470673686200
H	-4.781614387186	3.347577529784	-0.862880605899
H	-5.706771558561	2.267460804582	0.204426935873
C	-2.861024829162	-1.263243048557	1.836230913748
C	-3.455949159117	-0.134860263631	2.421806967847
C	-3.596441512220	-2.182674158530	1.076589709719
C	-4.834204255953	0.022592516553	2.280247234897
C	-4.974805134879	-1.987847381299	0.975076503557
C	-5.614520870440	-0.908289534413	1.587530642008
H	-5.312735331110	0.895576871661	2.733746001015
H	-5.564780649058	-2.699801712400	0.391537126578
C	-7.104905167170	-0.749004664669	1.510416117185
H	-7.589749621123	-1.118598158636	2.429479150049
H	-7.394365549319	0.306947549991	1.397560018815
H	-7.528988785461	-1.313438781106	0.667546013029
C	-2.905852360855	-3.317620208462	0.382275298828
H	-3.602070492128	-3.857713224691	-0.273366359934
H	-2.067633497014	-2.945747453187	-0.228156557712
H	-2.480695227833	-4.040762866717	1.096681808606
C	-2.608290188710	0.877157559003	3.127269111557
H	-2.067509384331	0.438556306448	3.980816912348
H	-1.838780931690	1.241168309979	2.420069485786
H	-3.206669252819	1.724524780339	3.487711788107
C	2.009042877624	-1.183428388710	1.982598012706
C	2.424581949628	0.141650009231	2.189094208314
C	2.913549117930	-2.193504488759	1.615137247123
C	3.752779408038	0.461557441108	1.911119209374
C	4.240761989724	-1.825818673552	1.384561666546
C	4.673046641715	-0.501575418340	1.490445901908
H	4.075306711613	1.498567077061	2.033703707296
H	4.950374541041	-2.596710337803	1.070560702439
C	6.074804083447	-0.115060387716	1.121657867091
H	6.409253993851	0.771718189730	1.679996859888
H	6.785183837701	-0.932927257737	1.309918805947

H	6.129740452329	0.123293597639	0.047517138329
C	2.489606472521	-3.626179732194	1.465934281350
H	3.207621269733	-4.181975910092	0.848907353941
H	2.431768933132	-4.138392237383	2.440325714337
H	1.500661949113	-3.710977514720	0.993050909014
C	1.474581399215	1.172653743390	2.709264935548
H	0.688046644217	1.379680672715	1.957457490183
H	0.952917370987	0.812500486317	3.610102351624
H	1.997252955559	2.106669277402	2.944901482508

References

[S1] a) R. C. Poulten, M. J. Page, A. G. Algarra, J. J. Le Roy, I. Lopez, E. Carter, A. Llobet, S. A. Macgregor, M. F. Mahon, D. M. Murphy, M. Murugesu, M. K. Whittlesey, *J. Am. Chem. Soc.,* 2013, **135**, 13640-13643; b) S. A. Johnson, E. T. Taylor, S. J. Cruise, *Organometallics,* 2009, **28**, 3842-3855.

[S2] A. J. Arduengo, III, S. F. Gamper, J. C. Calabrese, F. Davidson, *J. Am. Chem. Soc.,* 1994, **116**, 4391-4394.

[S3] a) D. H. Grant, *J. Chem. Educ.,* 1995, **72**, 39; b) D. F. Evans, *J. Chem. Soc.,* 1959, 2003-2005; c) S. K. Sur, *J. Magn. Res.* 1989, **82**, 169-173.

[S4] S. Stoll, A. Schweiger, *J. Magn. Reson.,* 2006, **178**, 42-55.

[S5] G. M. Sheldrick, *Acta Cryst. C,* 2015, **71**, 3-8.

[S6] M. J. Frisch, G. W. Trucks, H. B. Schlegel, G. E. Scuseria, M. A. Robb, J. R. Cheeseman, G. Scalmani, V. Barone, G. A. Petersson, H. Nakatsuji, X. Li, M. Caricato, A. V. Marenich, J. Bloino, B. G. Janesko, R. Gomperts, B. Mennucci, H. P. Hratchian, J. V. Ortiz, A. F. Izmaylov, J. L. Sonnenberg, D. Williams-Young, F. Ding, F. Lipparini, F. Egidi, J. Goings, B. Peng, A. Petrone, T. Henderson, D. Ranasinghe, V. G. Zakrzewski, J. Gao, N. Rega, G. Zheng, W. Liang, M. Hada, M. Ehara, K. Toyota, R. Fukuda, J. Hasegawa, M. Ishida, T. Nakajima, Y. Honda, O. Kitao, H. Nakai, T. Vreven, K. Throssell, J. A. Montgomery Jr., J. E. Peralta, F. Ogliaro, M. J. Bearpark, J. J. Heyd, E. N. Brothers, K. N. Kudin, V. N. Staroverov, T. A. Keith, R. Kobayashi, J. Normand, K. Raghavachari, A. P. Rendell, J. C. Burant, S. S. Iyengar, J. Tomasi, M. Cossi, J. M. Millam, M. Klene, C. Adamo, R. Cammi, J. W. Ochterski, R. L. Martin, K. Morokuma, O. Farkas, J. B. Foresman, D. J. Fox, *Gaussian 16 Revision B.01* (Gaussian, Inc., Wallingford CT, 2016).

[S7] a) S. Grimme, J. Antony, S. Ehrlich, H. A. Krieg, *J. Chem. Phys.,* 2010, **132**, 154104; b) E. R. Johnson, A. D. Becke, *J. Chem. Phys.* **2005**, *123*, 024101; c) Becke, A. D.; Johnson, E. R. *J. Chem. Phys.,* 2005, **123**, 154101; d) E. R. Johnson, A. D. Becke, *J. Chem. Phys.,* 2006, **124**, 174104.

[S8] S. Grimme, S. Ehrlich, L. Goerigk, *J. Comp. Chem.,* 2011, **32**, 1456-1465.

[S9] J. P. Perdew, K. Burke, M. Ernzerhof, *Phys. Rev. Lett.,* 1996, **77**, 3865-3868.

[S10] F. Weigend, R. Ahlrichs, *Phys. Chem. Chem. Phys.,* 2005, **7**, 3297-3305.

[S11] F. Weigend, *Phys. Chem. Chem. Phys.,* 2006, **8**, 1057-1065.

[S12] a) J. P. Perdew, M. Ernzerhof, K. Burke, *J. Chem. Phys.,* 1996, **105**, 9982–9985; b) C. Adamo, V. Barone, *J. Chem. Phys.,* 1999, **110**, 6158-6170.

[S13] F. Jensen, *J. Chem. Theory Comput.,* 2015, **11**, 132-138.

[S14] J. Tomasi, B. Mennucci, V. Cammi, *Chem. Rev.,* 2005, **105**, 2999-3094.

[S15] a) A. Klamt, G. Schüürmann, *J. Chem. Soc., Perkin Trans. 2,* 1993, 799-805; b) A. Klamt, *WIREs Comput. Mol. Sci.,* 2011, *1*, 699-709; c) A. Klamt, *WIREs Comput. Mol. Sci.,* 2018, **8**, 1-11.

[S16] T. Soda, Y. Kitagawa, T. Onishi, Y. Takano, Y. Shigeta, H. Nagao, Y. Yoshioka, K. Yamaguchi, *Chem. Phys. Lett.,* 2000, **319**, 223–230.

[S17] a) F. Neese, *Coord. Chem. Rev.,* 2009, **253**, 526-563; b) J. P. Malrieu, R. Caballol, C. J. Calzado, C. de Graaf, N. Guihéry, *Chem. Rev.,* 2014, **114**, 429-492.
